# Expansion of Human-Specific GGC Repeat in Neuronal Intranuclear Inclusion Disease-Related Disorders

**DOI:** 10.1016/j.ajhg.2019.05.013

**Published:** 2019-06-06

**Authors:** Yun Tian, Jun-Ling Wang, Wen Huang, Sheng Zeng, Bin Jiao, Zhen Liu, Zhao Chen, Yujing Li, Ying Wang, Hao-Xuan Min, Xue-Jing Wang, Yong You, Ru-Xu Zhang, Xiao-Yu Chen, Fang Yi, Ya-Fang Zhou, Hong-Yu Long, Chao-Jun Zhou, Xuan Hou, Jun-Pu Wang, Bin Xie, Fan Liang, Zhuan-Yi Yang, Qi-Ying Sun, Emily G. Allen, Andrew Mark Shafik, Ha Eun Kong, Ji-Feng Guo, Xin-Xiang Yan, Zheng-Mao Hu, Kun Xia, Hong Jiang, Hong-Wei Xu, Ran-Hui Duan, Peng Jin, Bei-Sha Tang, Lu Shen

**Affiliations:** 1 Department of Geriatrics, Xiangya Hospital, Central South University, Changsha, Hunan 410008, China; 2 Department of Neurology, Xiangya Hospital, Central South University, Changsha, Hunan 410008, China; 3 National Clinical Research Center for Geriatric Disorders, Xiangya Hospital, Central South University, Changsha, Hunan 410008, China; 4 Center for Medical Genetics, School of Life Sciences, Central South University, Changsha, Hunan 410008, China; 5 Department of Human Genetics, Emory University School of Medicine, Whitehead Research Building, Room 305A, 615 Michael Street, Atlanta, GA 30322, USA; 6 Department of Pathology, Xiangya Hospital, Central South University, Changsha, Hunan 410008, China; 7 GrandOmics Biosciences, Beijing 100000, China; 8 Department of Neurology, The First Affiliated Hospital of Zhengzhou University, Zhengzhou, Henan 450052, China; 9 Department of Neurology, The First Affiliated Hospital of University of South China, Hengyang, Hunan 421000, China; 10 Department of Neurology, The Third Xiangya Hospital, Central South University, Changsha, Hunan 410008, China; 11 Department of Neurosurgery, Xiangya Hospital, Central South University, Changsha, Hunan 410008, China; 12 Hunan Key Laboratory of Medical Genetics, Central South University, Changsha, Hunan 410008, China; 13 Key Laboratory of Hunan Province in Neurodegenerative Disorders, Central South University, Changsha, Hunan 410008, China

**Keywords:** neuronal intranuclear inclusion disease, whole-exome sequencing, linkage analysis, long-read genome sequencing, *NOTCH2NLC*, GGC repeat expansions

## Abstract

Neuronal intranuclear inclusion disease (NIID) is a slowly progressing neurodegenerative disease characterized by eosinophilic intranuclear inclusions in the nervous system and multiple visceral organs. The clinical manifestation of NIID varies widely, and both familial and sporadic cases have been reported. Here we have performed genetic linkage analysis and mapped the disease locus to 1p13.3-q23.1; however, whole-exome sequencing revealed no potential disease-causing mutations. We then performed long-read genome sequencing and identified a large GGC repeat expansion within human-specific *NOTCH2NLC*. Expanded GGC repeats as the cause of NIID was further confirmed in an additional three NIID-affected families as well as five sporadic NIID-affected case subjects. Moreover, given the clinical heterogeneity of NIID, we examined the size of the GGC repeat among 456 families with a variety of neurological conditions with the known pathogenic genes excluded. Surprisingly, GGC repeat expansion was observed in two Alzheimer disease (AD)-affected families and three parkinsonism-affected families, implicating that the GGC repeat expansions in *NOTCH2NLC* could also contribute to the pathogenesis of both AD and PD. Therefore, we suggest defining a term NIID-related disorders (NIIDRD), which will include NIID and other related neurodegenerative diseases caused by the expanded GGC repeat within human-specific *NOTCH2NLC*.

## Introduction

Neuronal intranuclear inclusion disease (NIID [MIM: 603472]) is a rare multisystem neurodegenerative disease characterized by the pathology of eosinophilic intranuclear inclusions in the central, peripheral, and autonomic nervous systems, as well as in the visceral organs.^1^, ^2^ The first NIID-affected case subject was reported in 1968.^3^ However, until 2011, only about 40 NIID-affected case subjects had been described worldwide, which were diagnosed by post-mortem brain biopsy.^1^, ^4^, ^5^, ^6^, ^7^, ^8^, ^9^, ^10^, ^11^, ^12^, ^13^ Because eosinophilic intranuclear inclusions also exist in dermal cells of NIID-affected individuals,^14^, ^15^ skin biopsy has become a useful tool to confirm NIID diagnosis, and the number of reported cases has increased to more than 100 case subjects.^2^

Both familial and sporadic case subjects have been described.^2^ The onset age of NIID varies widely and can be divided into three subgroups according to the age of onset: infant form, juvenile form, and adult form.^1^ The clinical manifestation of adult-onset NIID can vary widely, including dementia,^9^, ^16^ peripheral neuropathy,^5^, ^17^ autonomic dysfunction,^17^, ^18^ cerebellar ataxia,^7^, ^8^ parkinsonism,^4^, ^19^, ^20^ seizure,^21^ stroke-like episodes,^22^ disturbance of consciousness,^21^ and encephalitic episodes.^4^ Dementia is the most prominent symptom in sporadic NIID-affected case subjects. In familial NIID-affected case subjects, based on the initial and main symptoms, a subgrouping of adult-onset NIID-affected case subjects was suggested based on dementia-dominant and limb weakness-dominant phenotypes.^2^ Considering the wide range in distribution of intranuclear inclusions in the nervous system and other organs, it is possible that there are additional phenotypes of NIID. The typical symmetrical high signal seen in corticomedullary junctions using diffusion weighted imaging (DWI) could be a powerful tool for screening NIID-affected case subjects.^2^, ^15^, ^23^, ^24^ In addition, almost all dementia-dominant NIID-affected case subjects and about 40% of limb weakness-dominant NIID-affected case subjects have been seen to have remarkable leukoencephalopathy on fluid-attenuated inversion recovery (FLAIR) images and T2-weighted (T2) images, suggesting that severe white matter hyperintensity may also be an indicator for NIID.^2^, ^15^

The pathogenesis of NIID remains unknown. Immunohistochemically, the intranuclear inclusions are positive for ubiquitin and ubiquitin related proteins, including p62, SUMO1, FUS, MYO6, and OPTN-C proteins,^5^, ^12^, ^13^, ^25^, ^26^ indicating that the ubiquitin-proteasome system in the nucleus may play a role in NIID. In addition, some intranuclear inclusions were found to stain positive for an anti-polyglutamine antibody 1C2^4^, ^13^ and also with an anti-ataxin3 antibody.^11^, ^27^ However, this could be the result of a cross-reaction. No CAG repeat expansions have been observed in NIID.^27^

Here we report the identification of an expanded GGC repeat in *NOTCH2NLC* (MIM: 618025) via long-read genome sequencing (LRS) in a five-generation Chinese Han NIID family, and the repeat expansions were validated by repeat-primed PCR (RP-PCR) and GC-rich PCR (GC-PCR) in three additional NIID-affected families as well as five sporadic NIID-affected case subjects. In addition, we found two Alzheimer disease (AD)-affected families (1.43%) and three parkinsonism-affected families (1.46%), with GGC expansions in *NOTCH2NLC* from a cohort that included 140 AD-affected families, 205 parkinsonism-affected families, 51 spinocerebellar ataxia (SCA)-affected families, 16 peripheral neuropathy-affected families, and 44 motor neuron disease (MND)-affected families. Further, skin biopsies confirmed the typical NIID pathology in these two AD-affected families and three parkinsonism-affected families, indicating that GGC repeat expansions could also contribute to AD and PD phenotypes. Thus, we suggest defining a term of NIID-related disorders (NIIDRD), which is a spectrum of diseases including NIID and other diseases caused by the GGC repeat expansions of *NOTCH2NLC*. The prevalence of NIIDRD is not rare, and screening for expanded GGC repeat within *NOTCH2NLC* should be done not only in dementia-affected and peripheral neuropathy-affected individuals, but also in parkinsonism-affected individuals.

## Material and Methods

### Study Participants

Twenty affected individuals from four NIID-affected families, five sporadic NIID-affected case subjects, and 211 healthy control subjects from mainland China were recruited for this study. All NIID-affected individuals were recruited from the National Clinical Research Center for Geriatric Disorders, Xiangya Hospital, and subjected to a thorough neurological examination by at least two experienced neurologists. We defined familial NIID-affected case subjects as having at least one NIID-affected case subject diagnosed by skin biopsy, and the additional family member(s) had to have either high signal intensity in the corticomedullary junction using DWI or a clinical examination by a neurologist that confirmed the concordance of phenotype with the pathologically diagnosed NIID individual from the same family. All sporadic NIID-affected case subjects presented with typical high intensity in corticomedullary junction in DWI and received a skin biopsy to confirm the diagnosis. All healthy control subjects were recruited from the medical examination center of Xiangya hospital.

Besides NIID cohorts, a cohort of individuals with other neurodegenerative disorders were also recruited, including 140 AD-affected families, 205 parkinsonism-affected families, 51 SCA-affected families, 16 peripheral neuropathy-affected families, and 44 MND-affected families, of which the known pathologic genes had been excluded. All individuals were recruited from the Department of Neurology and National Clinical Research Center for Geriatric Disorders, Xiangya Hospital. All participants were subjected to a thorough neurological examination by at least two experienced neurologists.

This study was approved by the Ethics Committee of Xiangya Hospital of the Central South University in China. Written informed consent was obtained from all individuals.

### Skin Biopsy, Immunohistochemistry, and Electron Microscopy

Skin biopsies were performed after local anesthesia. A 5-mm-diameter biopsy specimen was obtained at 10 cm above the lateral malleolus of the affected individual. For immunohistochemistry, all samples were fixed in 10% formalin and then were embedded in paraffin and sectioned at 6 mm thickness. Sections of all samples were stained by hematoxylin & eosin (H&E) and immunohistochemical analysis was performed. Anti-ubiquitin (3936; Cell Signaling) and anti-p62 (610833; BD Biosciences) antibodies were used for ubiquitin and p62 staining. Images were acquired by deconvolution digital microscope (Axioplan 2; Carl Zeiss).

Samples for electron microscopy were sliced into 1×1×3 mm^3^ size and fixed in 2.5% glutaraldehyde solution with Millonig’s phosphate buffer (pH 7.3). In the following preparations, the samples were incubated in 1% osmium tetroxide, then dehydrated with graded acetone, and embedded with resin. 50–100 nm ultrathin sections were prepared with an ultramicrotome (Leica Microsystems) and a diamond knife. After 3% uranyl acetate and lead nitrate double staining, prepared sections were examined and photographed on a Hitachi HT-7700 electron microscope.

### Linkage Analysis and Whole-Exome Sequencing

**Figure 1:**
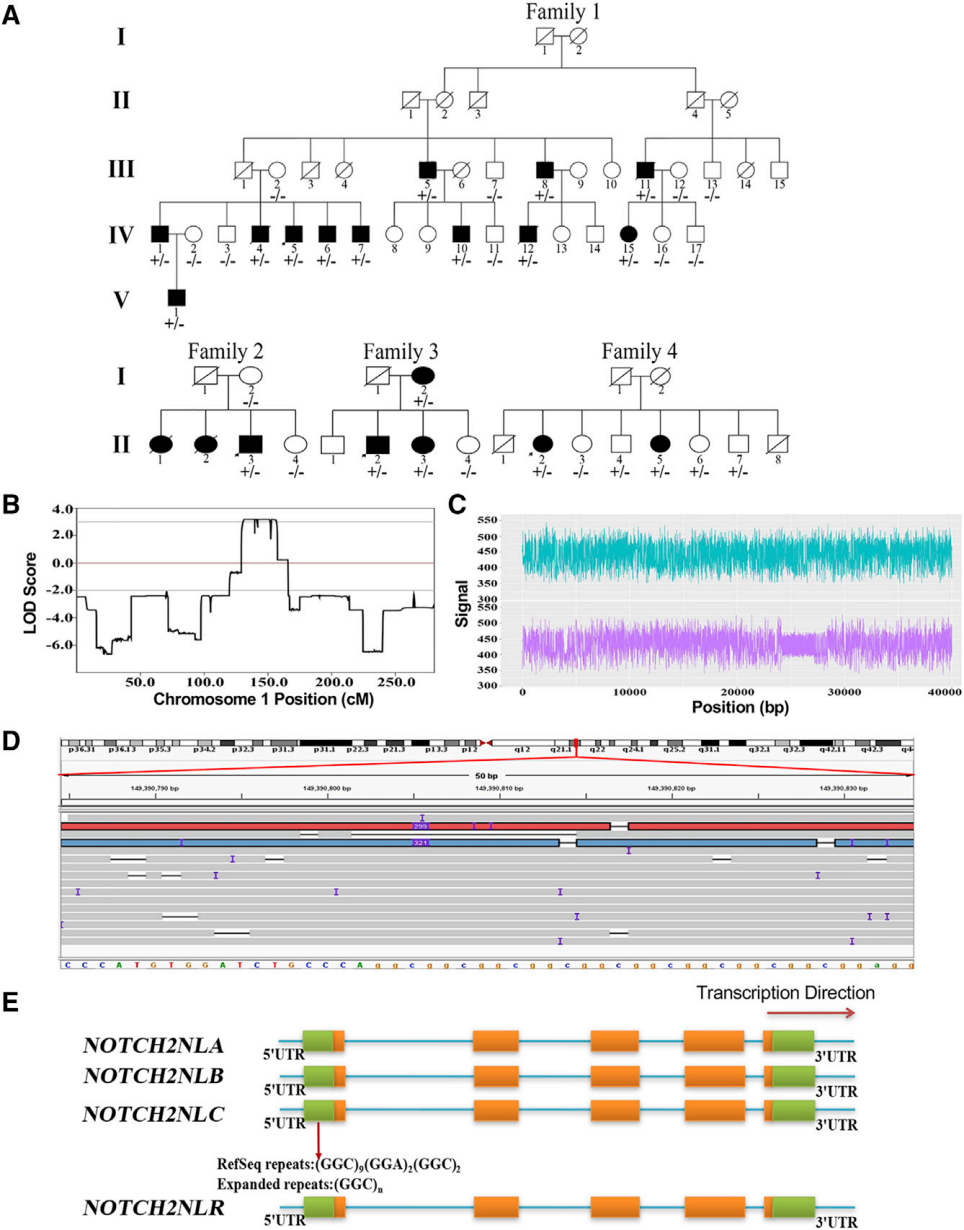
Identification of Expanded GGC Repeat within *NOTCH2NLC* in Neuronal Intranuclear Inclusion Disease (A) Pedigrees of neuronal intranuclear inclusion disease (NIID)-affected families and corresponding individual genotypes. (B) Genetic linkage analysis indicated maximum logarithm of odds (LOD) scores 3.184 in chromosome 1, a 49.8-Mb region at 1p13.3-q23.1 (chr1:109260034-159016186). (C and D) GGC expansions detected by LRS. Nanopore electric signal (C) from subject F1-IV:15 indicated GGC expansions in the lower lane compared to the normal allele in the upper lane. More than ten reads covering the causative region were seen in the Integrative Genomics Viewer for subject F1-IV:15. Two reads were determined to carry the “insertion” variation (chr1:149390803-149390842, hg38 version), corresponding to the GGC triplet expansion in *NOTCH2NLC* (D). (E) Schematic representation of the causal variant in *NOTCH2NLC*: a certain number of GGC triplets exist in 5′ UTR of *NOTCH2NLC* in healthy individuals, and large expanded GGC triplets are present in affected individuals.

Whole-genome SNP scanning on selected members in family 1 (Figure 1A) was performed using Illumina Asian Screening Array with more than 7 million polymorphic loci. Information on SNPs was extracted by the genotyping module of Genomestudio (v.2011.1). Illumina cnvPartition CNV Analysis Plug-in of Genomestudio (v.2011.1) was used for copy number variations (CNVs) analysis. Linkage analysis was performed by the parametric linkage analysis package of MERLIN (v.1.1.2). Whole-exome sequencing (WES) and analysis for the three affected individuals (F1-IV:1, F1-IV:6, and F1-IV:10) and one unaffected individual (F1-IV:3) were used to detect potential causal single nucleotide variants (SNVs) and InDels (insertions and deletions) in the mapping region. Sequencing data were subjected to quality control (QC). This was followed by Burrows-Wheeler Alignment Maximal Exact Matches (BWA-MEM) for read mapping, Samtools for SNV/InDel calling, ANNOVAR for variant annotation, and all potential disease variants were examined in Integrative Genomics Viewer.

### Long-Read Genome Sequencing

Long-read genome sequencing (LRS) on seven affected individuals (F1-IV:7, F1-IV:15, F2-II:3, F4-II:2, F5-II:1, F5-II:4, F9-II:6) and three healthy control subjects was performed on the Oxford Nanopore platform as describe previously.^28^ Combined analysis strategy of RepeatHMM and inScan were used for detection in the target region. Minimap2 and nanopolish calls were used for analysis of DNA methylation.

### PCR Assays and GGC Repeat Size Determination

For the repeat-primed PCR (RP-PCR) assay, a fluorescein (FAM)-labeled gene-specific primer (5′-CCTCAGCCCGATACTCACCAT-3′) and repeat-containing primers (5′-TACCAATACGCATCCCGCGATTTGTCTTA(CGG)_5_-3′) were utilized for identifying the CGG repeat expansion. For the GC-rich PCR (GC-PCR) assay, the fluorescein (FAM)-labeled forward primer (5′-AGCGCCAGGGCCTGAGCCTTTGAAGCAG-3′) and reverse primer (5′-TCGCCCCAGGTGGCAGCCCCGGGCGCCGCGGAC-3′) were utilized for repeat size determination. Seven-deaza-2-deoxy guanosine triphosphate (deaza-dGTP) was used in place of dGTP. PCRs were performed using 50 ng of genomic DNA in a 25 μL reaction mix including 10X Expand Long Template Buffer 1, 2.5 mM MgCl_2_ (Thermo Scientific, Cat#F-510Mg), 2% 2,4-dimethylsulfolane (Sigma, Cat#1003-78-7), 0.2 mM each of deaza-dGTP, dATP, dCTP, and dTTP, 0.2 μM primers (BioSune, Shanghai, China) and 1 U of DNA ploymerase from Expand Long Template PCR System (Roche, Cat#11681842001), using the following thermal conditions: 98°C for 4 min, followed by 30 cycles of 98°C for 45 s, 60°C for 45 s, 72°C for 4 min, and a final extension at 72°C for 7 min. The PCR products were subjected to capillary electrophoresis using the 3500xL Genetic Analyzer for Human Identification (Applied Biosystems). Allele sizes were determined using GeneScan 1000 ROX Size Standard (Applied Biosystems). GGC repeats less than 200 were determined directly by the size of PCR product as determined by ABI 3500xL and compared with the PCR products of known *FMR1* (MIM: 309550) premutation CGG repeat alleles of various sizes. For GGC repeats over 200, the size was determined using an Agilent 2100.

### RNA Extraction and Quantitative RT-PCR

Human postmortem dorsolateral prefrontal cortex (DLPFC) tissue from the subjects of different ages (4, 15, 36, and 60 years old) were obtained from the NIH NeuroBioBank. Total RNA was isolated using Trizol (ThermoFisher). Before reverse transcription, total RNA was treated with ezDNase (Millipore) for 5 min at 37°C to remove contaminated genomic DNA, and then incubated at 55°C for 5 min to inactivate the ezDNase. After ezDNase treatment, total RNA was extracted with phenol: chloroform = 25:24 (pH 5.2) and precipitated with glycogen and ethanol. The reverse transcription was conducted using SuperScript (II) (ThermoFisher, Cat# Cat. No. 18064-022) to generate first strand cDNA. The first strand cDNA served as the template for real-time PCR using FAST-7500 real time PCR equipment (Thermo Fisher). GAPDH TaqMan Gene Expression Assay (Hs02786624_g1) was used as an internal control. NOTCH2NLB primers: Forward (5′-GGGAGATATGAAGGGACGCA-3′); Reverse (5′-GGCACACACCTTCCATTCTC-3′). NOTCH2NLC primers: Forward (5′-CTGACCTTTCAAGATCCTGCTTTCATCCCAGCT-3′); Reverse (5′-AAGTGCCTTACTTTGCGTAGCTGTGTGCTTGGCAGT-3′).

### Statistical Analyses

For methylation analysis, the dispersion parameters were estimated through a shrinkage estimator based on a Bayesian hierarchical model, then a Wald test was performed as describe previously^29^ to identify differentially methylated loci (DML). For the expression data of *NOTCH2NLC* in the blood of both NIID-affected individuals and control subjects, statistical analysis was performed using Student’s t test. Differences with p < 0.05 were considered as being statistically significant.

## Results

### Identification of an Expanded GGC Repeat within Human-Specific *NOTCH2NLC* in a NIID-Affected Family

We recruited a five-generation Chinese NIID-affected family (family 1) with muscle weakness-dominant type (Figure 1A) and performed genetic linkage analysis. Our analysis identified a single peak with a maximum logarithm of odds (LOD) score of 3.184 at 1p13.3-q23.1 (chr1:109260034–159016186) (Figure 1B). To determine the genetic etiology of NIID in this family, we performed whole-exome sequencing (WES) in three affected individuals (F1-IV:1, F1-IV:6, and F1-IV:10) and one unaffected individual (F1-IV:3) (Figure 1A), and we were unable to identify any nonsynonymous or potentially causal variants. We went on to perform low-coverage long-read genome sequencing (LRS) on two other affected individuals (F1-IV:7, F1-IV:15) using the Oxford Nanopore platform. Intriguingly, we observed that one allele of *NOTCH2NLC* had an expanded GGC repeat in both affected individuals (Figures 1C and S1), which WES failed to detect.

The expanded GGC repeat is located at the 5′ end of *NOTCH2NLC*. *NOTCH2NLC* is one of the four NOTCH2 paralogs that are present only in the human genome; the other paralogs are *NOTCH2NLA* (MIM: 618023), *NOTCH2NLB* (MIM: 618024), and *NOTCH2NLR* (MIM: 618026). Previous studies have shown that all four NOTCH2NL paralogs contain the first four exons and introns of NOTCH2 as the result of a partial segmental duplication of the NOTCH2 ancestral gene.^30^ *NOTCH2NLA*, *B*, and *C* are highly similar to each other, with more than 98% sequence identity over NOTCH2NL exons 1–5.^30^ However, only *NOTCH2NLC* contains the GGC repeat in the reference human genome (hg38), (GGC)_9_(GGA)_2_(GGC)_2_, which is expanded in the NIID-affected family that we have examined here (Figures 1D and 1E).

### Expanded GGC Repeat Is Associated with NIID

**Figure 2:**
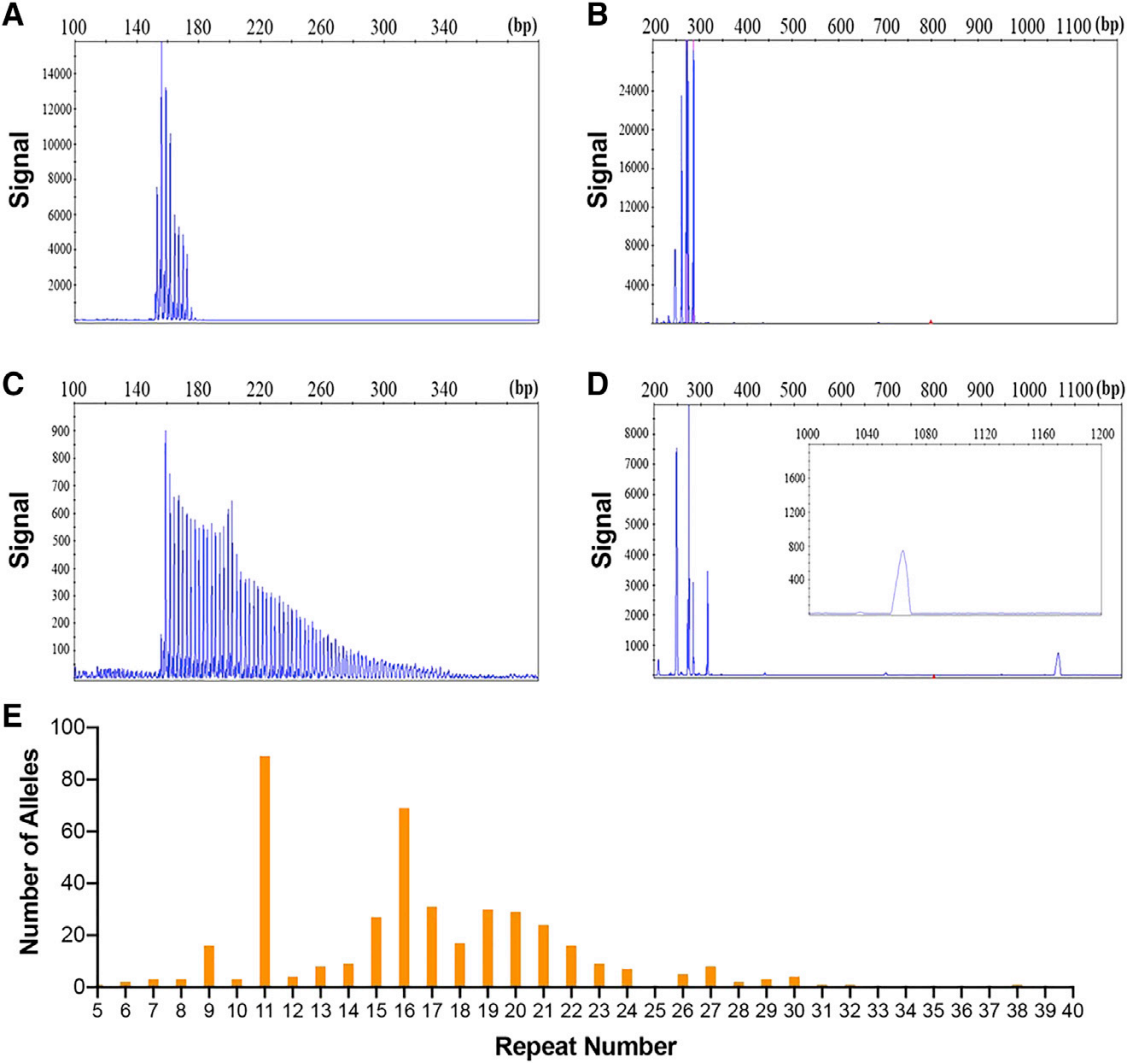
Validation of Expanded GGC Repeats and Variations of GGC Repeat Size among Normal Individuals (A–D) Representative electropherogram of the RP-PCR assay and the GC-PCR assay showed abnormal repeat expansion in affected individuals (C and D) and negative result in control subjects (A and B). (E) Size distribution of GGC repeat among healthy control subjects, which are usually less than 40 repeats.

To confirm our Nanopore sequencing results, we designed primer sets for both repeat-primed PCR (RP-PCR) and GC-rich PCR (GC-PCR) assays to detect both normal and expanded GGC repeats. Among the 211 healthy control subjects that we examined, the GGC repeat sizes range from 5 to 38, with modes at 11 and 16 repeats (Figure 2E). Our RP-PCR analysis confirmed the expanded GGC repeat among the affected family members in family 1 (Figure 1A). All affected individuals in this family showed similar peak patterns using RP-PCR, indicating the presence of expanded GGC repeats (Figure 2C), while unaffected members of the pedigree carried only normal repeats (Figure 2A). We then determined the repeat size using GC-PCR and found that all the affected individuals carry alleles with greater than 100 repeats (Figure 2D). To further confirm the role of GGC repeat expansion in NIID, we examined three additional NIID-affected families (families 2–4) (Figure 1A) and five sporadic NIID-affected case subjects by RP-PCR and GC-PCR assays, some of which were further confirmed by LRS (Figure S1). All affected individuals from both familial and sporadic case subjects carried an expanded GGC repeat larger than 66. Together, these results suggest that GGC repeat expansion is indeed associated with NIID.

However, we did note that the expanded GGC repeat was also observed in a few individuals that were initially thought to be phenotypically normal, such as F1-III:5 and F1-III:8 (Figures S2A and S2B). Upon re-examination of subject F1-III:8, we found that this individual has subclinical peripheral neuropathy and typical eosinophilic intranuclear inclusions (Figures S2C and S2D). Subject F1-III:5 did not complain of limb weakness and refused to receive further examination but presented with tremor and disturbance of consciousness, which are the classic symptoms of NIID. Nevertheless, this indicates the possibility of varying severity for expanded GGC repeat-caused NIID.

### Expanded GGC Repeat Is Associated with Dementia and Parkinsonism

**Figure 3:**
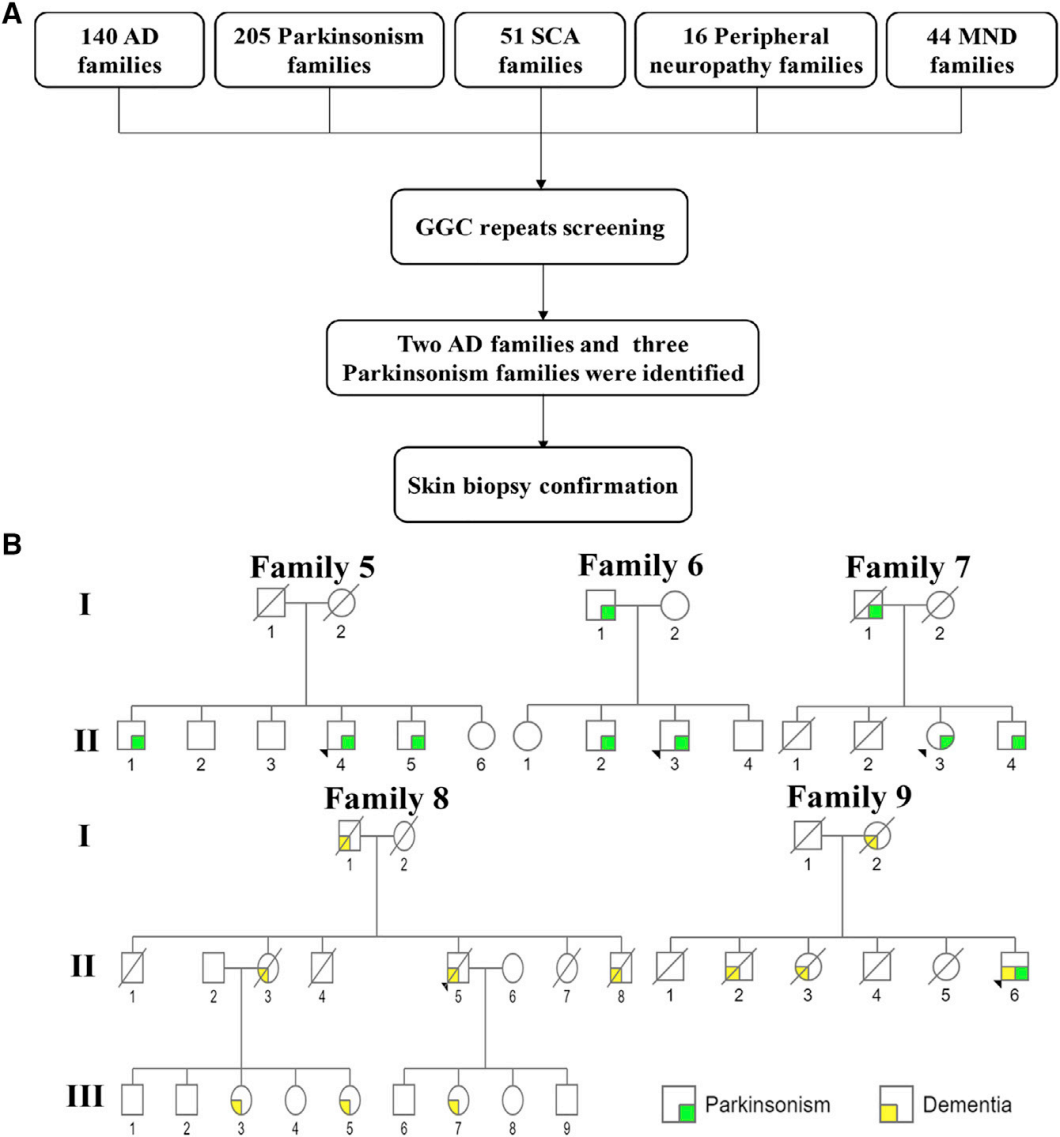
Expanded GGC Repeats Are Associated with Dementia and Parkinsonism (A) Flow chart of GGC repeat expansion screening in a cohort of families with neurodegenerative disorders. (B) Pedigrees of AD-affected and parkinsonism-affected families that carry expanded GGC repeats in *NOTCH2NLC*.

Because of the wide range of clinical manifestations associated with NIID, we further determined the size of the GGC repeat in a cohort of 140 families with AD, 205 families with parkinsonism, 51 families with SCA, 16 families with peripheral neuropathy, and 44 families with MND (Figure 3A). Surprisingly, we found affected individuals from three parkinsonism-affected families (families 5–7) and two AD-affected families (families 8 and 9) that carry the expanded GGC repeat in *NOTCH2NLC* (Figure 3B, Tables 1, S2, and S3). LRS was also performed on three affected individuals (F5-II:1, F5-II:4, F9-II:6), confirming the presence of expanded GGC repeat (Figure S1). Upon reexamination, we confirmed that these individuals display classic PD and AD symptoms, respectively. Intriguingly, their skin biopsies revealed typical eosinophilic, p62-positive, and ubiquitin-positive intranuclear inclusions, the pathological feature associated with classic NIID-affected individuals (Figures S3A–S3H and S3M–S3T). These findings suggest that the expanded GGC repeat could also be associated with dementia and parkinsonism.

**Table 1:** Summary of Clinical Features of NIID

	**Sporadic Case Subjects (n = 5)**	**Familial Case Subjects**			
		**Total (n = 40)**	**Muscle Weakness (n = 15)**	**Parkinsonism (n = 9)**	**Dementia (n = 16)**
Sex ratio (male/female)	2/3	26/14	12/3	8/1	6/10
Average onset age (range)	62.0 (51–69)	50.6 (30–78)	35.6 (30–54)	60.6 (37–78)	58.1 (31–71)
Average disease duration (range)	5.6 (1–14)	12.5 (1–49)	16.6 (3–49)	6.0 (1–15)	12.4 (2–30)

**Clinical Manifestations**					

Dementia	2/5 (40.0%)	14/40 (35.0%)	0/15 (0%)	0/9 (0%)	14/16 (87.5%)
Abnormal behavior	2/5 (40.0%)	15/40 (37.5%)	2/15 (13.3%)	0/9 (0%)	13/16 (81.3%)
Peripheral neuropathy					
Muscle weakness	0/5 (0%)	18/39 (46.2%)	13/15 (86.7%)	1/9 (11.1%)	4/15 (26.7%)
Sensory disturbance	0/5 (0%)	13/37 (35.1%)	4/13 (30.8%)	4/9 (44.4%)	5/15 (33.3%)
Autonomic dysfunction					
Bladder dysfunction	3/5 (60.0%)	22/39 (56.4%)	5/15 (33.3%)	5/9 (55.6%)	12/15 (80.0%)
Miosis	2/5 (40.0%)	5/29 (17.2%)	1/11 (9.1%)	0/8 (0%)	4/10 (40.0%)
Parkinsonism					
Tremor	1/5 (20.0%)	19/40 (47.5%)	11/15 (73.3%)	5/9 (55.6%)	3/16 (18.8%)
Rigidity	1/5 (20.0%)	12/40 (30.0%)	1/15 (6.7%)	9/9 (100.0%)	2/16 (12.5%)
Bradykinesia	1/5 (20.0%)	12/40 (30.0%)	1/15 (6.7%)	9/9 (100.0%)	2/16 (12.5%)
Ataxia	0/5 (0%)	7/40 (17.5%)	2/15 (13.3%)	4/9 (44.4%)	1/16 (6.3%)
Neurological attack					
Disturbance of consciousness	4/5 (80.0%)	7/40 (17.5%)	3/15 (20.0%)	1/9 (11.1%)	3/16 (18.8%)
Stroke-like episode	4/5 (80.0%)	4/40 (10.0%)	1/15 (6.7%)	2/9 (22.2%)	1/16 (6.3%)
Encephalitic episode	3/5 (60.0%)	2/40 (5.0%)	1/15 (6.7%)	0/9 (0%)	1/16 (6.3%)
Brain MRI					
Severe leukoencephalopathy	5/5 (100.0%)	7/20 (35.0%)	1/7 (14.3%)	2/6 (33.3%)	4/7 (57.1%)
DWI U-fiber high signal	5/5 (100.0%)	6/16 (37.5%)	1/3 (33.3%)	1/6 (16.7%)	4/7 (57.1%)
Cognitive function test					
MMSE (<education matched average)	0/2 (0%)	4/21 (19.0%)	0/7 (0%)	0/7 (0%)	4/7 (57.1%)
MoCA (<education matched average)	1/2 (50.0%)	9/16 (56.3%)	3/7 (42.9%)	4/5 (80.0%)	2/4 (50.0%)
Nerve conduction					
MCV slowing	3/3 (100.0%)	19/22 (86.4%)	8/9 (88.9%)	6/6 (100%)	5/7 (71.4%)
CMAP reduction	1/3 (33.3%)	14/22 (63.6%)	7/9 (77.8%)	2/6 (33.3%)	5/7 (71.4%)
SCV slowing	3/3 (100.0%)	15/22 (68.2%)	7/9 (77.8%)	3/6 (50.0%)	5/7 (71.4%)
SNAP reduction	1/3 (33.3%)	13/22 (59.1%)	6/9 (66.7%)	2/6 (33.3%)	5/7 (71.4%)
Skin biopsy	5	14	3	5	6
Average no. of GGC repeats (range)	105 (86–133)	188 (66–517)	272 (118–517)	83 (66–102)	129 (91–268)

### Expanded GGC Repeat and Phenotypic Variability

A total of 40 affected familial individuals with expanded GGC repeats, ranging from 66 to 517 repeats, were analyzed in this study. Only about 37.5% of affected familial individuals presented with the classical NIID radiological findings of symmetrical hyperintense linear lesions in the corticomedullary junction in DWI (Figure S4A) and severe white matter hyperintensity in FLAIR images/T2 weighted image (Figure S4B). However, all skin biopsies revealed eosinophilic, p62-positive, and ubiquitin-positive intranuclear inclusions in dermal cells (Figure S3). Electron microscopy imaging also revealed intranuclear inclusions lacking membranes (Figure S3H). The main clinical manifestations in these affected familial individuals included bladder dysfunction (56.4%), tremor (47.5%), muscle weakness (46.2%), abnormal behavior (37.5%), sensory disturbance (35.1%), dementia (35.0%), rigidity (30.0%), bradykinesia (30.0%), ataxia (17.5%), disturbance of consciousness (17.5%), miosis (17.2%), stroke-like episodes (10.0%), and encephalitic episodes (5.0%) (Table 1). Based on the initial and main clinical manifestations among these affected individuals with expanded repeats, we were able to divide the familial case subjects into three subgroups: muscle weakness-dominant type, parkinsonism-dominant type, and dementia-dominant type (Table 1 and Supplemental Note).

The muscle weakness-dominant type (families 1 and 2) was similar to what has been reported for classic NIID where the main and initial clinical manifestation is muscle weakness. The average onset age was around 36 (Figure 1A, Tables 1 and S1). Muscle weakness usually began in the distal part of lower limbs, then moved up to the throat muscles and face. However, the severity and position of muscle weakness could vary significantly between individuals. Tremor was frequently seen in the early stages of almost all of the affected members. The number of GGC repeats in this subgroup ranged from 118 to 517 repeats.

The parkinsonism-dominant type (families 5–7) presented with parkinsonism as the main clinical manifestation. The onset age was typically around 60 years old (Figure 3B, Tables 1 and S2). The severity of symptoms varied greatly, even between siblings with similar repeat sizes. No dementia was observed in either the muscle weakness or parkinsonism groups. A smaller repeat expansion, 66–102 repeats, was observed in this group.

In the dementia-dominant type (families 3, 4, 8, 9), individual characteristics were similar to the previously described dementia-dominant phenotype in NIID.^2^ The average onset age was 58. Dementia was the most prominent symptom (Figures 1A and 3B, Tables 1 and S3). Abnormal behavior, autonomic dysfunction, disturbance of consciousness, tremor, rigidity, limb weakness, and sensory disturbance were also seen. This subgroup repeat size ranged from 91 to 268 GGC repeats.

### Methylation and Expression at *NOTCH2NLC* Locus

**Figure 4:**
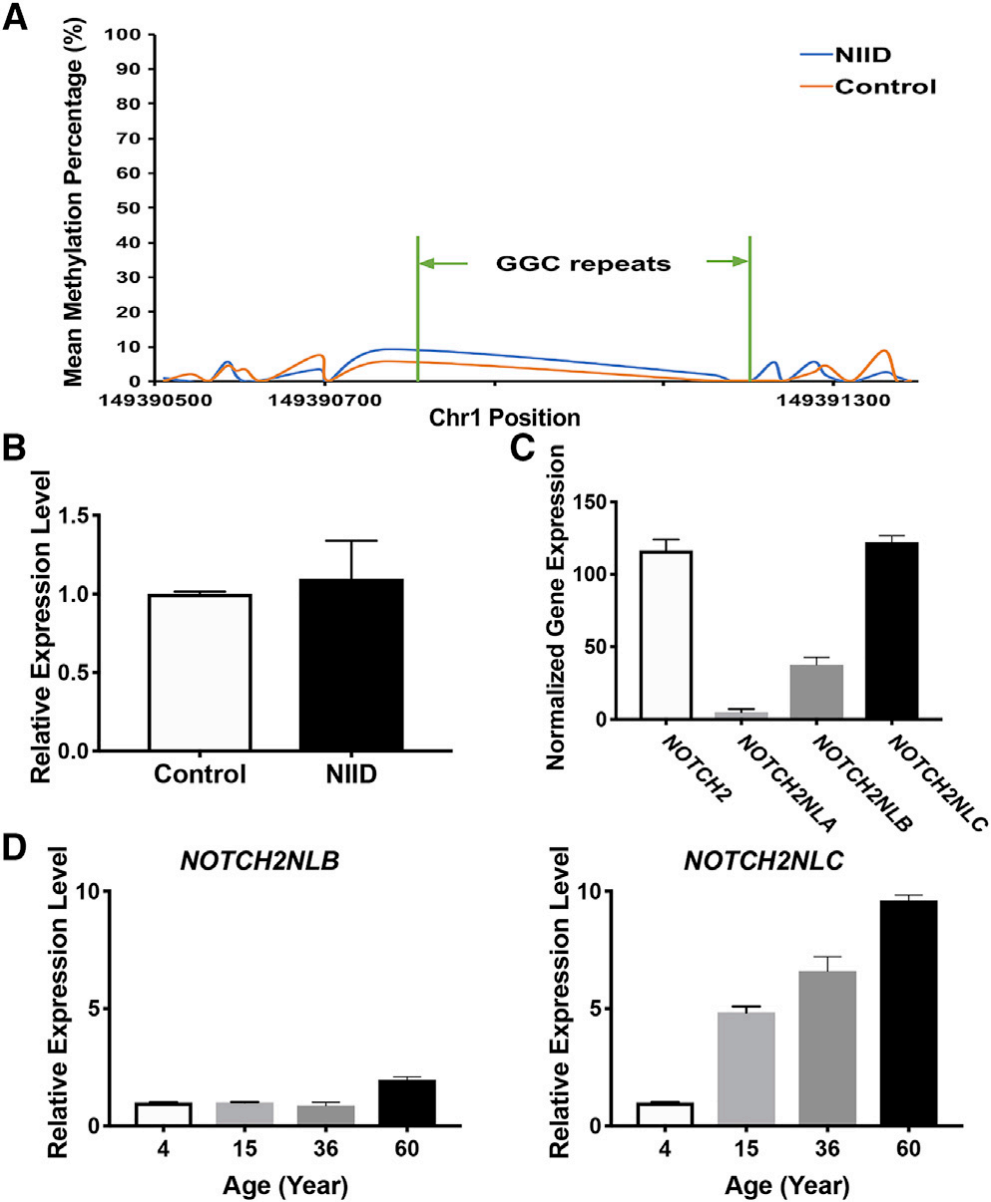
Methylation and Expression at *NOTCH2NLC* Locus (A) Methylation status across expanded GGC repeats region was determined using LRS data from seven affected individuals (F1-IV:7, F1-IV:15, F2-II:3, F4-II:2, F5-II:1, F5-II:4, F9-II:6) and three healthy control subjects, and no significant methylation difference was detected between NIID-affected case subjects and control subjects. Wald test was performed for statistical analysis; ^∗^p < 0.05. (B) *NOTCH2NLC* expression level in both NIID-affected case subjects (NIID) and normal control subjects (Control). The ezDNase-treated total RNA isolated from the blood of both NIID-affected case subjects and control subjects was reversely transcribed into cDNA followed by quantitative PCR. *GAPDH* was used as internal control. Error bars represent the SD; Student’s t test was performed for statistical analysis; ^∗^p < 0.05. p = 0.776 (ns). (C) Expression levels of *NOTCH2* and three *NOTCH2NL* paralogs (*NOTCH2NLA*, *NOTCH2NLB*, and *NOTCH2NLC*) in human adult cortex detected by RNA-seq. Significant differences were observed in the expression levels of these four genes. Shown are the normalized gene expression levels. Error bars represent the SD. (D) Dynamic change of *NOTCH2NLC* expression in human brain during aging. Relative expression levels of both *NOTCH2NLB* (left) and *NOTCH2NLC* (right) in DLPFC region of human brain during aging are shown. The total RNA isolated from the DLPFC of human postmortem brains from 4-, 15-, 36-, and 60-year-old subjects were used for quantitative RT-PCR with *GAPDH* as internal control. Error bars represent the SD.

Previous works have found that more than 200 CGG repeats at the *FMR1* locus can lead to hypermethylation of the CGG repeat and adjacent CpG islands, resulting in transcriptional silencing.^31^ Because many of the expanded repeats that we discovered at the *NOTCH2NLC* locus in NIID-affected case subjects were greater than 200, we examined the methylation status of GGC repeat and adjacent regions by re-analyzing the signals from Nanopore sequencing. The methylation level around the GGC repeats was very low and no significant methylation difference between NIID and control was detected (Figure 4A), which suggests that the expanded GGC repeat at the *NOTCH2NLC* locus is likely unmethylated. Furthermore, we determined the expression of *NOTCH2NLC* in the blood of both NIID-affected case subjects and control subjects, and did not detect significant changes in expression for the NIID-affected case subjects with expanded GGC repeats (Figure 4B), indicating that the expanded GGC repeat does not alter the expression of *NOTCH2NLC* and that the GGC repeat RNA could potentially play a role in the molecular pathogenesis of NIID.

Previous studies have shown that *NOTCH2NLA*, *NOTCH2NLB*, and *NOTCH2NLC* can be transcribed in human ES cells.^30^, ^32^ We analyzed the expression of these three paralogs and *NOTCH2* using the published human brain RNA-seq datasets^33^ and found that *NOTCH2NLC* displays the highest expression in the brain among these three NOTCH2 paralogs (Figure 4C). Given that NIID is a neurodegenerative disorder, we designed specific primers that could distinguish the expression of *NOTCH2NLB* and *NOTCH2NLC* by RT-PCR (*NOTCH2NLA* has very low expression in brain; Figure 4C) and determined the expression dynamics of both genes during human brain aging. Interestingly, while the expression of *NOCTH2NLB* displays only modest changes in human prefrontal cortex during aging, the expression of *NOTCH2NLC* increased significantly with age (Figure 4D). Thus, the increased expression of expanded GGC repeats during aging could contribute to the molecular pathogenesis of NIID.

## Discussion

Neuronal intranuclear inclusion disease (NIID) is a slowly progressing neurodegenerative disease characterized by eosinophilic intranuclear inclusions in the nervous system and multiple visceral organs. The clinical manifestation of NIID varies widely, and both familial and sporadic case subjects have been reported.^2^ Here we report the identification of a GGC repeat expansion at the 5′ end of *NOTCH2NLC* as the genetic cause of NIID. *NOTCH2NLC* is one of the three human-specific *NOTCH2*-related genes (*NOTCH2NLA*, *NOTCH2NLB*, and *NOTCH2NLC*) in 1q21.1, highly expressed in the brain and thought to be involved in the evolutionary expansion of the human brain.^30^, ^32^ Thus, NIID represents a genetic disorder caused by an expanded repeat in a human-specific gene.

Almost 30 years ago, expansion of unstable nucleotide (microsatellite) repeats, notably trinucleotide repeats, was identified as a previously unknown mutational mechanism underlying certain human diseases.^34^, ^35^, ^36^, ^37^ In recent years the development of next-generation sequencing along with the focus on exome capture provided a massive increase in the catalog of genes with mutations in Mendelian disorders;^38^, ^39^, ^40^, ^41^, ^42^, ^43^, ^44^, ^45^, ^46^, ^47^, ^48^, ^49^, ^50^ however, in many instances the causal mutations could not be identified even when genome-wide association studies (GWASs) or genetic linkage analysis identified a specific genetic locus. Recent works, including identification of an unstable hexanucleotide repeat within *C9ORF72* (MIM: 614260) as a frequent cause of frontotemporal dementia (FTD)/amyotrophic lateral sclerosis (ALS)^51^, ^52^ and CTG repeat expansion as the cause of Fuch’s endothelial corneal dystrophy (FECD),^53^ suggest that repeat expansions could contribute to the genetic etiology of these disorders, which could be missed by short-read sequencing. In our initial analyses, we failed to detect any causal mutations using WES despite the fact that we mapped the NIID-causing gene to 1p13.3-q23.1. Our findings presented here further highlight the importance of considering the genetic contribution of unstable repeats in disorders where traditional single nucleotide variants (SNVs) or copy number variants (CNVs) cannot be identified.

In addition to GGC repeats, we did observe different repeat units, such as GGT, through our LRS analyses (Figure S1). This is likely due to the high error rate of LRS itself and interference of sequence from paralogs. With the high error rate of Nanopore sequencing, it was challenging to acquire accurate sequence data within the expanded GGC repeat region, which would require further analyses with more accurate methodology. Despite these technical issues, most reads that we obtained were expanded GGC repeats.

The key features associated with the disorders caused by unstable nucleotide (microsatellite) repeats are genetic instability and anticipation.^31^ In the NIID-affected families reported in our study, a wide range (66–517) of repeat numbers were observed in affected individuals, while control subjects have fewer than 40 repeats. Anticipation was seen in a few families, while unlike fragile X-related disorders, the pioneer locus with expanded CGG/GGC repeats, increased instability of the GGC repeat size over generations was not observed in all families. Therefore, anticipation was not clearly seen in NIID-affected families, and more NIID-affected families need to be studied to explore the genetic pattern over multiple generations. Nevertheless, the number of affected individuals does seem to increase over multiple generations, suggesting a potential genetic anticipation effect. We also observed variable severity in the clinical manifestation among our affected case subjects; however, there is no apparent association between GGC repeat size and the severity or the onset age in NIID. It is possible that additional genetic or environmental factors could contribute to the pathogenesis of NIID. In addition, extensive works on the *FMR1* locus have shown that there is a threshold for the methylation status of the CGG repeat.^31^ Typically, if the CGG repeat is below 200, it is unmethylated, but the majority of expanded CGG repeats over 200 are hypermethylated, leading to transcriptional silencing of *FMR1* and fragile X syndrome.^31^ Thus, it has been hypothesized that loci with more than 200 CGG repeats could be recognized by DNA methyltransferase(s) and induce hypermethylation.^54^ Similar observations have recently been reported in Baratela-Scott syndrome (BSS), which was found due to an expanded GGC repeat in *XYLT1* (MIM: 608124).^55^ However, as reported here, many expanded alleles associated with NIID-affected individuals exceeded 200 repeats but were not methylated. Our finding suggests that in addition to GGC/CGG repeat size, additional factor(s) or sequence(s) could also determine the methylation status of expanded GGC/CGG repeat *in vivo*.

Our analyses of both classic NIID and other individuals with neurological conditions revealed a broadened clinical spectrum of adult-onset NIID. Besides the suggested dementia-dominant type and muscle weakness-dominant type,^2^ we have identified the parkinsonism-dominant type as a clinical presentation in NIID. Among sporadic case subjects, we also identified a paroxysmal disease-dominant type (Supplemental Note, Table S4). Furthermore, GGC repeat expansion was also observed in two AD-affected families (1.43%) and three parkinsonism-affected families (1.46%), implicating that GGC repeat expansions in *NOTCH2NLC* could also contribute to the pathogenesis of both AD and PD. Therefore, we suggest defining a term NIID-related disorders (NIIDRD), which include NIID and other related neurodegenerative diseases caused by the expanded GGC repeat. It is likely that the prevalence of NIIDRD could be higher than initially thought. Given that only about 37% of affected individuals showed typical MRI features of NIID, genetic testing for the expanded GGC repeat could potentially be a more sensitive and accurate screening tool for NIIDRD.

Identification of the GGC repeat expansion as the genetic cause of NIID will help elucidate the molecular pathogenesis in NIID. Our results presented here suggest that the GGC repeat expansion-induced pathogenesis is likely dominant, which suggests that the expression of the expanded GGC repeat could cause the neuronal toxicity associated with NIID and its related disorders. Indeed, a dominant-negative gain-of-function model has been proposed for another neurodegenerative disorder, fragile X-associated tremor/ataxia syndrome (FXTAS [MIM: 300623]), which is also caused by the expanded GGC/CGG repeats.^56^ Intriguingly, it was previously discussed that NIID and FXTAS share many clinical and neuropathological features,^27^, ^58^ which could be well explained by our genetic findings presented here. Two potential mechanisms have been proposed for FXTAS: RNA toxicity and repeat-associated non-AUG (RAN) translation.^59^ The CGG repeat RNA could sequester key RNA binding proteins and prevent them from performing their normal physiological function.^56^ Also, repeat-associated non-AUG (RAN) translation of the CGG repeat into polypeptides, the predominant species being poly-Glycine (FMRpolyG peptides), could contribute to FXTAS pathogenesis.^60^, ^61^ Both *FMR1* mRNA and FMRpolyG peptide have been found in the human postmortem brain inclusions that are characteristic of FXTAS pathology, suggesting that both mechanisms may contribute to the disease.^60^, ^62^ It will be important to explore both mechanisms in NIID pathogenesis in the future. In addition, it could be challenging to establish appropriate model(s) to study NIID since *NOTCH2NLC* is a human-specific gene.

In summary, we have identified an expanded GGC repeat within human-specific *NOTCH2NLC* as the genetic cause of NIIDRD, which could include dementia, parkinsonism, and peripheral neuropathy. Our finding will facilitate more accurate clinical diagnosis in the future and help us better understand the molecular pathogenesis of NIIDRD in general.

## Declaration of Interests

The authors declare no competing interests.

## Web Resources

GenBank, https://www.ncbi.nlm.nih.gov/genbank/
Genomestudio (v.2011.1), https://support.illumina.com/array/array_software/genomestudio/downloads.html
gnomAD Browser, https://gnomad.broadinstitute.org/
IGV, http://www.broadinstitute.org/igv/
inScan, https://github.com/Nextomics/inScan
MERLIN (v.1.1.2), http://csg.sph.umich.edu/abecasis/Merlin/download/
Minimap2, https://github.com/lh3/minimap2
Nanopolish call, https://github.com/jts/nanopolish
OMIM, http://www.omim.org/
RepeatHMM, https://github.com/WGLab/RepeatHMM
UCSC Genome Browser, https://genome.ucsc.edu

## Supplemental Data

Document S1. Supplemental Note (case reports), Figures S1–S4, and Tables S1–S4

Document S2. Article plus Supplemental Data

## Footnotes

Supplemental Data can be found online at https://doi.org/10.1016/j.ajhg.2019.05.013.
