# Supplementary material for: Expansion of Human-Specific GGC Repeat in Neuronal Intranuclear Inclusion Disease-Related Disorders
Source: Am J Hum Genet. 2019 Jun 6;105(1):166–76. doi: 10.1016/j.ajhg.2019.05.013 (PMC6612530; doi:10.1016/j.ajhg.2019.05.013)
Supplement: Document S1. Supplemental Note (case reports), Figures S1–S4, and Tables S1–S4 [file mmc1.pdf]

The American Journal of Human Genetics, Volume *105*

Supplemental Data

Expansion of Human-Specific GGC Repeat in Neuronal Intranuclear Inclusion Disease-Related Disorders

Yun Tian, Jun-Ling Wang, Wen Huang, Sheng Zeng, Bin Jiao, Zhen Liu, Zhao Chen, Yujing Li, Ying Wang, Hao-Xuan Min, Xue-Jing Wang, Yong You, Ru-Xu Zhang, Xiao-Yu Chen, Fang Yi, Ya-Fang Zhou, Hong-Yu Long, Chao-Jun Zhou, Xuan Hou, Jun-Pu Wang, Bin Xie, Fan Liang, Zhuan-Yi Yang, Qi-Ying Sun, Emily G. Allen, Andrew Mark Shafik, Ha Eun Kong, Ji-Feng Guo, Xin-Xiang Yan, Zheng-Mao Hu, Kun Xia, Hong Jiang, Hong-Wei Xu, Ran-Hui Duan, Peng Jin, Bei-Sha Tang, and Lu Shen

Supplemental Note

Case Report

Case F1-IV: 6

A 47-year-old male presented with limb weakness for 14 years. His four brothers also presented with muscle weakness in their early 30s. Muscle weakness began in the distal portion of the lower limbs, then moved up to the throat muscles and face. He routinely felt numbness in his limbs. The neurological examination revealed miosis, muscle atrophy, bilateral hand resting tremor, postural tremor, intention tremor, ataxia and weakness of the four limbs. Mini-Mental State Examination (MMSE) score was 29. Nerve conduction studies showed slowed motor and sensory nerve conduction velocity. Eosinophilic intranuclear inclusions were found in his skin samples, and the diagnosis of NIID was made.

Case F6-II: 3

A 49-year-old male was admitted to the hospital because of progressive slow walking over the last 3 years. The man was diagnosed with Parkinson's disease in 2017, and showed good response with dopaminergic therapy. He also complained about sleep disorders, constipation, and urgency of urination. The neurological examination revealed muscle rigidity and bradykinesia of right upper and lower limb. Unified Parkinson's disease rating scale (UPDRS) motor score was 5. Memory, orientation, and calculation were normal. The Mini-Mental State Examination (MMSE) score was 30, and the Montreal cognitive assessment scale (MoCA) score was 25. Blood chemistry was normal. Further skin biopsy confirmed the diagnosis of NIID.

Case F9-II: 6

An 81-year-old male presented with progressive cognitive decline for 10 years, and was diagnosed with Alzheimer's disease in 2015. His medical history included hypertension, diabetes, and he had to wear a catheter at all times due to urine retention over the past 10 years. His siblings had also been diagnosed with Alzheimer's disease. He had experienced several loss of consciousness episodes that lasted a few minutes each. The neurological examination revealed cognitive impairment, miosis, bilateral hand resting tremor, muscle rigidity, and bradykinesia. His brain DWI images showed typical high signal in the corticomedullary junction, Fluid-attenuated inversion

recovery (FLAIR) images showed severe white matter hyperintensity. CSF examination was normal, including the protein level and cytology. Electroencephalogram showed an increase in slow waves and no epileptiform discharges. He further received a skin biopsy which confirmed the diagnosis of NIID.

Case SP1

A 69-year-old male with left hemiplegia was admitted to the hospital. There was no medical history or family history for him. The neurological examination revealed complete recovery from the left hemiplegia. MMSE score was 27. No typical high signal in acute cerebral infarction was observed in DWI, while symmetrical hyperintense linear lesions was presented in the corticomedullary junction. Nerve conduction studies showed slowed motor and sensory nerve conduction velocity. His skin samples indicated typical NIID pathology.

Figure S1. Summary of Reads Covering Expanded Repeat Region from Oxford Nanopore Platform.

ID	Reads	Insertion Size	Repeat Number	Repeat Pattern	Match Ratio (observed read/pure repeat)
F1-IV:7	read 1	666	222	GGT	77%
	read 2	645	215	GGT	74%
F1-IV:15	read 1	344	114	GGT	88%
	read 2	340	113	GGC	78%
	read 3	295	98	GGC	86%
	read 4	370	123	GGT/GGC	81%/78%
F2-II:3	read 1	561	187	GGC	74%
	read 2	568	189	GGC	71%
	read 3	625	208	GGC	73%
	read 4	628	209	GGC	83%
	read 5	635	211	GGC	76%
F4-II:2	read 1	322	107	GGT/GGC	80%
	read 2	327	109	GGT/GGC	79%/77%
	read 3	384	128	GGC/AGC	87%/83%
	read 4	410	136	GGC	76%
	read 5	357	119	GGC	83%
	read 6	342	114	GGC	84%
F5-II:1	read 1	342	114	GGC	84%
	read 2	263	87	GGC	84%
	read 3	248	82	GGC	87%
F5-II:4	read 1	311	103	GGT/GGC	86%/84%
	read 2	348	116	GGT	88%
F9-II:6	read 1	345	115	GGC	78%
	read 2	396	132	GGC	85%
	read 3	403	134	GGC	78%
	read 4	449	149	AGC	81%
	read 5	356	118	GGC	82%
	read 6	385	128	GGC	77%
	read 7	343	114	GGT/GGC	77%/84%

Raw sequence data of expanded repeat region from Oxford Nanopore platform.

<u>F1-IV:7-read 1</u>

ACCATAAGTGTTTAGAAGTTTGAATTTTATCCTAGGGAGCTAGGGAGAGGTTCT
ATGGGTAGATTTCCTCATCCCAACAGATTGTAAAACTCCCACATCCAATTAGTC
ACACCTCCATTTTTTTTCTGTTTCTTTAACATTGTCTCAGATTCATTCCTTCTGA
AATCTACCTTATTAGTTCAGGTCTTCTTAAATTTGTGCTCTGGACTGTTCACTCA
ACTCTTTGACTGGTCCTGGGCTCCAGTTCACCTCTTGCTCTAAATAATCAATTT
TCTACAGAACTACTGAGCAATCTCCTTCCATCAATCTCAACTCTGTTCAAAAAA
CTTGTTGTGTTTTATAGACCCAAGAGGTAGCTGCTCCCACCCTTTCAGCTTGCT
AGTCTGGCTGTACACTGTCCCAACCCCTTTTCCTATTCTTGACTCCTCCTCTCA
CTGCTAACAGAGACAGAACTCATTTACTTACTCTTTGACTGTAATTTTCGAGCC
ACGAGGGCTCTTTCCGAATGATATGTCCATACAACTATTGTTGTTGTTCTTACTT
TTAGCCTCTTTTTCTTAGCCCACTTGTACCCAGGTTTGACTACACCCCTACTCTC
CTGCCCTGAAGTAACTAATATTCTGGTTATAGTTCTTAATATCTATATCTATACAT
TCTATATATATGCATCCACAGATAATGTAGTATTGTCTTTAAAAAAATATACATGT
ACCGTATACTTTTGTCTCCTCTCTGCATTAGCTTTTTTTCACTATCATTTATTTTT
AAGATTTATCCCTATTGGACATAAATAGCTATAGTCTTTTCAATTACTGTGTTGC
ATTTTACCTTATAAATATGCTCATACATTGTCTATCCATTCCTCTAAGGTGTGGCA
TTTATGTTGTTTTCAAGGTTTTGCTATTACAGTGTTAGATAAATATCCTTAGCTTA
GCTGGTCTCCTTGTCTTATACAAATTTTACGACCTTTCAAGATCCTGCTTTCATC
CCAGCCTCTGGGAAAGCTCTTCCTGAACTTTCAGCCTTGGCTGTGCCAGCAAA
GTCTGGCATCAAAGTTGCTTGCATCTTTGGTATCATCCCACTGCTAAAGCACAA
CAGCTACTAAAGTAAGGCACTTTAAGAAATATCTGCCACACGGATGAATGTGA
ATGAATGAATGGGGCAGTACCACTCGTGATGGCTCTATTCCTTTTAATGGACTA
TGCCTGGGCCTACTTCCTTCTTTGGATGTTAATCATCCACTCCAGAGCATTCTCT
AGCTAGCAAAGCAGTGTGGATGTGGGTGTAGGTGGCGATGCCAGACTCACTG
GATGAGGACCGCCTCCCTGCAAGTCACGATCCCGGCAACTCCAGTGCTTGAA
GTTCAGAGTCCTACCCCACCCCCCTCACCTCCACGCCCTTCTGCACTGGTCGT
TTTTGAAGGAGCCGCTTGGCCCTGATCGAGTTAAGGCTGCTGGAGAAGGATGC
GGACGGGCCAGTGACTCGTAGTAGATCCTCCGCGCGAGCTCGGGTCGGCGCT

TCTTCCTGCGGGAAACCCTGGTGCCCAAGGTGGCGGGGGCTGAGGCTGCGGC
GACAGTGGGTAACAGGTGGGAGGAGTGGCTGAGGGAAGGATTGGTCGAGGA
TGCTTCTGCTGCACACCTGAGAAAGTTTCACAAAACTTCGGGCGGCGGCTGG
AGGCGGCGGCCGAGGAGCGCGGACCTGGGGCGCGGGGAGTCGAGGCATTTG
CGCTTCTGTAAGCTGACTGTGTTAGGGCTTGAGTTTTTGAAGCAGGAGGAGGG
GAGGAGAGAGGGGTTTCACGGGGACCTTCCCATGTGGATTCAAGCCCAGGTG
GCGGTGGCGGGGCAGTGGCGGTGGTGGCGGTGGTGGTGGCGGTGGCGGTGG
CGGTGGTGGTGGTGGTGGTGGTGGTGGTGGTGGTGGTGGTGGGTGGTGGTGG
TGTGGGTGGTGGCGGGTGGTGGTGGCGGTGGTGGTGGCGGTGGTGGTGGCGG
TGGCGGTGGCGGCGGCAGGTGGTGGTGGCGGGTGGGTGGTGGTGGTGGTGG
GTGGTGGTGGTGGTGGTGGTGGTGGTGGTGGTGGTGGAGGTGGTGGGTGAGG
GAGAGGTGGTGGTGGAGGGAGGTGGGTGGTGGAGGGAGGTGGTGGTGGAGG
AGTGTGGTGGTGGAGGAGGTGGGTGGTGGAGGGAGGTGGTGGTGGAGGGAG
GTGGTGGTGGAGGGAGGTGGTGGTGGAGGAGGTGGTGGGTGAGGAGGTGGG
TGGTGGGAGGGAGGTGGTGGTGGAGGAGGTGGTGGTGGTGAGGTGGTGGTG
AGAGGGAGGTGGTGGTGGAGGGAGGTGGTGGTGGAGGAGGTGGTGGTGGAG
GAGGTGGCGGTGGAGGAGGTGGTGGTGAGGGAGGTGGTGGTGGAGGAGGTG
GTGGTGGAGGAGGTGGTGGTGGAGAGGTGGGTGGTGGAGGAGGGTGGTGGC
GGGAGGAGGTGGCGGCGGAGGAGGCGGCGGCGGGAGGAGGCGGCGGC

F1-IV:7-read 2

ATTTTGACTACGTTTATTTTTGAAAAGGGTTTCCAGCATTTCAATGTGACTATCA
CTAATGATACTTGCTTTTGCCTAAAGTGCTTGTTAATATGCAGTTTTGGAATTCT
GGCAGGTCTATGCCCATACTGACTCTTCAATTTCTGCTTAGTGTTGTTTACATCT
CTAGTTCAACACCTATTATGTGATAGACACTGGGATTAATAGGCTGTGGTCTTT
GCCCATGAGGACCCTCAAAACTTAGTTGAGAACACAGGAAATACAAAGAGTC
CTGTGTGAAAAATAAGCATTGGTGTTAAGATAAAGAAGCGGGGCAGTGGAAG
ACAGTGGAGGGATCTCTGTCTGTTAGAGGGAGGAATTTATTGCAGTGTCGGCA
AAGCCTTCTTTGGACTGAAGGATGAATTATGATTTAAAGTTCATTAAGAGGGA
GGAGGGAAGGGACTACAGGAGTCATTCCAAGCCAAAGGAACAAAAGATACA
GGGACAGAAACAGCAAGATATGTTTGGGGAATCTCTGGGTTAGCTAATACTGT

TATTGTGTAAGTTCACAACAGGGGCAGGGAGGACAGGAGATGGGGCCCAATG
ACTAGCCTTTGTATGTGCCCATGTTTAGGTTTGAATTTATCCTATAGGAGTCAGG
AGAGGTTCTATGGGTAGGATTCCCATCCCAACAGGATTGTAAACTCCTGGCAC
ATCCAATTAGTTTACACCTCCATTTTTTTTCTCATTTCTTTAACATTGTCTCAGA
TTCATCTTCTGAAATTCACCTTATTAGTTCAGGTCTTTAAATTTGTGCCTGGACT
GTTCACCAACTCGATCGGTCCTGGGGCTCCAGTTCACCTTGCCCCAACAATCA
ATTTTTCTACAGAACTATCAGGGACAATCTTCCTTTCCATCAATCTCAACTCTGT
TCAAAAACTTTCAATAGTTCTTATGTGACCTAAGAGGTAGGCTGCCTCCCACCC
TTTCAGCTTGGGCTATCTGTTGCCGTATAATTTGTCCCAAATTTCTTTCCGGTTC
TTGACTCCTCTTCTCACTGCTAACAGAGACAGAACTATTTACTTACTCTTTGAC
TGTACCTGCTTTCACTTAGGCTCTTCCTGAATGATATGTCCATACAACTATTGTT
GTTGTTCCATTTTTAGCTCTTTTCTTAGCCCACTGTACCCAGGTTTGATCACACC
CCCTACTCTGCCCTGAAGTAACCACATTCTGGTTATAGTCTTTAATATCTATATTT
ATCTATATATGCATCCACATGGCTAATGTGTAGTATTGTCTTGAAAAAATATACAT
AAATGGTATAATTTTTGTCTCCCTTACATTAGTTTTCACTATCATTTAGTTTTAAG
ATTTATCCTATTGACATAAATAGCTATAGTCTTTTCAATTACTGTGTTGCATTTCA
TTTTATAAATATACCACATTGTCTATCCATTCCTCTAAGGGTGGGTATTTATGTTG
TTTTTCAAGGTTTTACATGGTGTTACAATCTAATATCCTTAGCTTAGCTGGTCTC
CTTGTCTTATGCTACAAATTTTACTGACCTTTCAAGATCCTGCTTTTCATCCCAG
CTCCTTCGGGGAAAGCCTTCCTGAATCCCAGCCCACAGCGCAAAAGTCTTGGT
ACTAAAGCTGCTTGTGCATCTTGGTATCATCCTACTGCTACAATAACAGCTACT
TAGCAAGGCACTTAAGAAATATTCGCCACACGGATGAACAAATGAATGAATGA
ATGGGGCAGCACCACCCGTGATGGCTCTCTATTCCTTTTAATGGACTTTATGCC
TTGGCCCACTTCTTTCCTTCTTTGACAGCACTGGTCCTGTACTCCAGAGCATTC
CTCTAGCTAGCAAAGCAGTGTGTGGATGTGTGGGGTGTAGGTGGCGATACAGA
CCTCACTGGATGAGGGACCGCCTCTCCTCTGCAAGTTCACGATCCCGGCAACC
CCAGTGCTTCAGTTCAGAGTCCCACCTCACCCCTCACCTCCACGCCCCTTTCT
GCACTGGTCAAGCCAGAGCCGCTGCAGCCCCATCGAGTTAAGGCCATGGAGA
AGGATGCGACGGGGCCAGTGACCTGTAGATCCCTCCGCGCGGAGCCTGGGCT
GGCGCTTCTTCCTGCGGGAAACCCTGGTGCCCAAGGCGGCGGGGCCGAGCCG

CGGCGACAGGCGGGGCTTGCGGTGGAGGAGGCGGCTGAGGTGAAGGACACA
CGAGGCTGCTGCACACCTGAGAAAGCCAGCCAAACTTCGGGCGGCGGTCGGT
GGCGGCTGAGAGGGCGGACCGGGGCGCGGGGAGTCGAGGCATTTGCGCTTTC
CAAAGCCTGGACCGTAGCCAGGCCTGAGCTTGAAGCAGGAGAGGGAGAGAG
AGTGGCCTTTCTATCGGCCTTCCTATGTGGATTCGTCGGTGGTGGCGGCGGGTG
GCGGCGGTGGCGGTGGCGGCGGTGGCGGCGGTGGCGGTGGTGGCGGTGGTG
GTGGTGGTGGCGGTGGTGGTGGTGGTGGTGGGTGGTGGTGGTGGCGTGGCAG
GCGTGGTGGTGGTGGTGGTGGGTGGTGGCGGCGGGTGGTGGTGGTGGCGGGT
GGCGGTGGTGGTGGTGGTGTGGTGGTGGCGGTGGTGTGGCGGCGGTGGAGGG
GCGGGTGGTGTGGAGGTGGGTGTGGTGGAGGAGGTGGTGGTGGAGGTGGTG
GTGGAGGAGGGTGGTGGAGGGGTGGTGGAGGGAGGTGGTGGTGGGAGGGGA
GGTGGTGGTGGGAGGGAGGTGGGTGGTGGAGGAGGTGGTGGTGGAGGAGGT
GGTGGTGGAGAGGTGGTGGTGGAGGGAGGTGGTGGTGGAGGGAGGGGTGGT
GGTGGAGGAGGTGGCGGGTGGAGGGAGTGGTGGTGAGGGAGGTGGTGGTGG
AGGGAGGTGGCGGGTGGAGGAGGTGGTGGCGGGAGGAGGTGGCGGTGGAGG
AGGTGGCGGCGGGAGGGAGGTGGCGGGTGGTGAGGCGGTGGCCGGAGGAGG
TGGCGGCGGAGGAGGCGGCGGCGGAGGAGGGCGCGGCGGAGGAGGCGGCG
GCGGAGGAGGCGGC

F1-IV:15-read 1

TGATAGACACTGGGGATTAATAGGCTGTGGTCTTTGCTCCATGAGGACCCCTTA
AACTTAGTTGAGAACACAGAAATGCCAAAGAGTCCTGTGTGAAAAATAAGTAT
TGGTGTTAAGATGGAAGAAGTTTGGTGGAAGACAGTGGAGGGACTGTCTGTT
GGAGGGAGGAATTTATTGTGTCGGCAAAGCCTTCTTTTGGACTGAAGGATGAA
TATGATTAGAAGTTCATTAAGAGGAGGAGGGAGGGGACTTACAGACATTCAAG
CCAAAGGAACAAAAGATACAGGACAGAAACAGCGCAAGATATGTTTGGGGGA
ATCTCGTTAGCCAATACTGTTACGTGAAGTTCACAACAGGGTAGGAGGACAGG
AGATGGGGCCCAATGACGCAAAAAAGGCCTTGTATGTACCATGTTTGAAGTTT
GAATTTATCCATAGAGTCAGGGGAGAGACTACAGTAGGATTTCCATCCCAATGT
AGGATTGAAACCCCACATCCAGTCACACCTCCATTTTTTTTCTTTCTGCTTTTT
AACATTGTCTCAGATTCATTCTCCTTCTGGAAATCTACTCATAGTTCAGGTCTTC

TTAAATTTGTGCCTGACTGTTCACCAACTTGACTGGTCCTTAGCTCCAGTTCAC
CTCTTGCTCCCCAACAATCAATTTTCACAGAACTATCAGAGCAATCTTCCCTTT
CCATCAATCAACTCTGTTCAAAAACTTTCAATAGTTTTATGACCTAAGAGGCTT
CCTCACCCTTTCAGCTTCTGCCATTCAAGGCCGTGTACTTTGTCCCAACCCCTT
CCGTCCTTGGACTCCTCTCACTGCTAATGAGATACAGAACTCATTTACTTACTC
TTTGACTGTACCTTTGCTTTCACGAGGGCTCTTTCCTGGAATGATATGTCCATAC
ACAACTATTGTTGTTGTTCTTAGCTTTCAGTCTTTTCTTAGCCTCACTGTACCCA
GGTTTGACCCACACCCCGCCTCCTGCCCTGAAGTAATTACTTATTCCAAGTTAT
AGTCTCTAATATCTATATTCATATTCATATATATAACCAAATAGAGTACGTAGTATT
GTCTTGAAAAAATATACATAAATGGTATACTTTGTCTCCCTCAAGCATTAGTTTT
TTCACTATCATTTAGTTTTTAAGATTTATCTTATTGAGGCTAAATAGCTATAGTCT
TTTCACTCATTGTGTGTTGTGTATTTCATTTTATAAATATACCATGTTAATATTCAT
CCATTCCTCTAAGGGTGGGTAGTACTTGTTACTTTTCAAGGTTTTGCTATTACA
GTAACCAAATATCCTTTAGCTTAGCTGGTCTCCTTGTCTTATACAAATTTTTTAC
TGACCTTTCAAGATCCTGCTTATCCCAGCTCCTCTGGGAAAGCCTTCCCTGAAT
CCCCAGCCCACAGCGGCAAAGTCTGGTACTCAAAGTTGCTTGTGCACTTTGGT
GTCATCCCACTGCTACACACAGCTACGCAAAGCAAGGCACTTACATGATGATA
GCCCAAATGAATGAATGAATGGGGCAGCACTACTCCGTGATGGCTCTATTCCTT
TTTAATGGACTTGGCGCCTTGGCCCACTTTCCTTCTTTGACAGCACTGGTCCTA
CCAGAGCACATTCTCTAGCTAGCAAAGGTGGATGTGTGGTGTAGGTGGCGATA
CAGACCTCACTGGATGAGAACGCCTCCCTGCAAGTTCACGATCCCGGCAACCT
GTGCTTGAAGTTCAGGGAGTCCTACCCCACCCCTCACCTCCACGCCCTTCTGC
ACTGGTCATTAAAGGAGCCGCTGCAGCCCTGATCGAGTTAAGGCTGCTGGAG
AAGGATGCGGACGGGCCAGTGACTGTAGTAGATCCCTCTGCGCGGAGCCTGG
CCGCGCTTTCTTCCTGCGGGAAACCCCAGGCTCAAGGCAGCTGAGGCTCGCG
GCGCGACAGGGCGGGGGCTTATGGGAGGAGGCGGCTGAGGCGGAAAGACAC
GAGGTTGCTTGCTGCACACTGAGAAAGTTTCAGCCAAATCTCATGGCGGGTTG
AGGCGGCGGCCGAGGAGCGGCGGGACTCTGGGGCGCGGGGGGAGTTGAGGG
CATTTGCGCTTGCGTTGGACCGTAGCGCCAGGGCTCTGAGCTTTGAAGCAGGG
GAGGGAGGGGAGAGAGGCCTTTCTTATCGGGACCCTTCCCATGTGGGATCTGC

CCAGGCGGCGGGCGGTGGCAGTGGGTGGTGGCGGGTGGTGGTGGTGGT
GGTGGTGGTGGTGGTGGTGGGTGGTGGTGGTGGTGGTGGTGGTGGTGGT
GGTGGTGGTGGTGGTGGTGGTGGTGGTGTGGTGGTGGTGGTGGTGGTGG
TGGTGGTGGGTGGTGGTGGGTGGTGGTGGTGGTGGTGGTGGTGGTGGTG
GTGGTGGTGGTGGTGGTGGTGTGGTGGTGGTGGTGGTGGTGGTGGTGGG
TGGTGAGGGAGGTGGTGGTGGAGAGGTGTGGTGGTGGTGGTGGGTGGTGGTG
GAGGGAGGTGGTGGTGGAGGGAGGTGGTGTGGAGGAGGC

<u>F1-IV:15-read 2</u>

TTAGTGAGTAAAGAGGAAGATCAGACTGGTAACACTGCTTGGTGAAACAACT
GAAAAATAATGTTATCTTTCTCTGAACATACTTACAAGACAAATTTAAAAATATT
TTGACTACGTTATTTTTTGAAAAAGGGTTTCCAGTATTTCAATGTGACTACTTTC
ACTAATGATGCCTTTTCAAAGTTAACATTAACATGCAGTTTTGGAATTTTCATAG
ATCTACCTTTATATACTGACTTGTACTTGCTTAGTGTTGTTTTACATTCTAGTTCA
ACATGTTATGTGATGAACACTGAGGTTAATGGCTGTGGTCTTTGCCCATGAGGG
ACCTCAAAACTTAGTTGAGAACACAGAAATACAAAAGAGTCCTGTGTGAAAA
TAAGTATTGGTGTTAAGATAAAGAAGCTGACAGTGGAAGACAGTGGAGGGAC
TGTCTGTTAGAGGGAGAATTGTTGGGTGTCGGCAAAGCCCTTCTTTGGACAGA
GATGGGAGTGTGATTAGAAGTTCATTAAGAGGAGGAGGAAGGGACTACAGTC
ATTTCAAGCCAAAAGGAACAAAAGATACAGGGACAGAAACAGCAAAGATATG
TTTGGGGAATCTCAAGTTAATACTGTTGTTGTGTGGGAAGTTCACAACAGGGG
CAGGAGGAGGACAGGAGATGGGGCCCAATGACGAAAAGGCCTTGTATGTACC
ATGTTTGGGGGTTGAATTTTATCCTGGAGTCAGGGAGGTTCTGGGTGGGATTTC
CCATCCCAACAGGATTGTAAACTCCCACATCCAATTAGTCCCTCATTTTTTCTG
TTCCTTTAACGTGTCTCAGATTCATCCCTTCTAAATCTACCTTATTGAGTTTCAA
GGTCTTCTTAAATTTATTGCCTGGACTGTTCACCGCCTCTTGACTGGTCCTGGG
GCTCCAGTTCACCTCTTGCTCCCAACAATCAATTTTCTACAGAACTATCAGAGC
AATCTTCCTTTCCATCAATCTCAACTCTGTTCAAAACTTTCCAATAGTTTTTTAT
GCTTGAGCTAAGAGGTAGCTTCTCCCACCCTTTCAGCTTGCCATTCGAGGCTG
CATAATTTAGTCCCAACCCCTTTCGTCAGCAGCTCCTCTCCTGCTAACAGAGAC
CATTCATTACTTCTTTTGACTGTACCTTTGCTTTCCACGAGGGCTCTTTCTGGA

ATGATATGTCCATACAACTATTGTTGTTGTTCTTACTTTTAGCTCTTTTTCTGCTT
AGCCCACTTGTGCCCAGGTTCAGTCACCCCCCTCTCCCTGCCTGGAAGTAACT
ACTATTCTGGTTATAGTCTACTTAATATCTATATCTATATCTATATATATGCATCCAC
AGATAATGTGTGATATTGTCTTGAAAAAAATATACATAAATGGTATACTTTGTCT
CCTGCGATTAGTTTTTCACTATCTTTAGTTTTAAGATTTATCCCTATTGACATAAA
TAGCTATAGTCTTTTCAATTACTGTGTTGCATTTCATTTATAAATATACCACATAC
ATTATTCATCCATTCCTCTAAAGGTGGGCATTTATGTTGTTTTCAGGTTTTGCTAT
TACAATATTTGTGAATATCCTTAGCAGCTGGTCTCCCTTGTCTTATACAAATTTT
ACTGACCTTTCAAGATCCTGCTTTCATCAACTCCTCTGGGAAAGCCTTCCCTG
AATCCCCCAGCCCACAGCAAGTCTTGGTACATCAAAGTGCTTGTGCATCTTTG
GGTATCATCACTGCCAAGCACACAGCTCGCAAAGTAAGAGCATAGAAATATTC
GCTTCACGGATGAACAAATGAATGAATGAATGGGGCCTTCCCACTCCATCGTG
GCTTATTCCTTTTGCAATGGACTTTGGCGCACCTTGGCCCCACTTTCCTTCTTT
GACAGCACTGGTCCTACTCAGAGCATTCTCCTTTAGCAAAGCAGTGTGTGGAT
GTGTGGGTGTGGGTGGCGATACAGACCTCTTTGGATGAGGACCGCCTCTCCCC
TGCAAGTTCACGATCAGCAAACTCTCAGTGCTTGAAGTTCAGAGTCCTGCCCA
CCCCTCACCTCCACGCCCTTCTGCACTGGTCAAGCCCAGCGAGCCGCTGCAGC
CTGATCGAGTTAAGGCTGCTGGAGAAAGGGTGCGGACGGGGGCCAGAGAGA
GTGGCTCGTAATGGGTCCCTCCGCGCGGAGCTCCGGGCCGGCGCTTCTTCCTG
CTGAAACCCTGGGTGCTGCCCAAGGCGGCAGGGGCCGAGGCCGCGGCGACT
GTGGAGGGCAGGGGCTTGCGGTGGGAGGAGGCGGCTGAGGCGGAAGGACAC
ACGAGGCTGCTTCCTTTGCACACCGAAAGTTTCAAGCCAAACTGGGCGGCGG
CTGGGGCGGCAGCCGAGGGCGGCAGCTCGGGGGCGCGGGGAGTCAGGCATT
GCGCCTGTGCTTCGGACCGTAGCGCCAGGCCTGAGCCTTTGAAAGCATGGGA
GGAGGGAGGAGGTGGGAATCCTCTATCAGGACCCCCTCCCATGTGGATCTGCC
CAGGCAAAGGCAAAGGCGGCGGCGGCGGCGGCGGCGGCAGCGGCGGC
GGCGGCGGCGGCAACTGGCGGCTGGCAACAACGGCAACAGCGGCAGCGGCG
GCAGCGGCGGCGGCGGCGGCGGCGGCGGCGGCGGCGGCGGCGGCGGCG
GCGGCACGGCGACGCTGGCAACGGCACGGCTGGCTGGCAACGGCGGCGGCA
GCAGCACGGCTGGCGGCGGCGGCGGCGGCGGCGGCGGCGGCGGCGGCAAGG

AGGAGGCGGCAGCGGAGGCAGCGGAGGAGGCGGCAGCGGAGGAGGCGGCA
GCGGAGGGCAGCAGCGGAGGGGAGGCAGCGGCGGAGGAGGCGGC

F1-IV:15-read 3

CTGGGATTATGACGCAAAGCGCCCGCCTGGCCTACATTTATTTAATAATGTATGC
TTCTAGTCGCTTCTAGGTTGCTCTCTTTGGGGATAACTATCTTATATTCTTCTACA
GATATGTTTTAATAGTGACAAAACTGCTTTACGGTGATTTTAAGAAAATTTGTAT
GCAGCCAGTACTATATGAATTATCTCTGGTGAGTAAAGAGGAAGATCGAACTG
GTAACACTGTGGGTAACAACTGAAAAATGTTATCTTTCTCAAGAACATACTTG
CTGAACAAATTCAAAAAATATTTTGACTCGTTATTTTTGAAAAAGGGTTTCTGA
CAATATATTTCAAATGCGACTACTTTCACTAATGATGCCCCTTTTAAAGTGCTTG
GTAACATGCGGTTTTGGAATTTCCTTAGGTCTATGCCCTTGACTTGTGTACTTG
CTTTTGATGTTGTTTACATTCTAGTTCAACATCTATTATGTGATAGACACTGGGA
TTAATAGGCTGGCGATTCTTTTACCCATGAGGACCTCAAACTTGATTGAGAACA
CAGAAATACAAAAGGAATCCTGGCCGAAAATAAGTATTATTGTTGGAATAAAG
CGGCAATGGAAGACAGTGGAGGGACTGTCTGTTAGAGGAATTATTGTGGTGTC
GGCAAGCCTTCTTTGATGAGGATAATTCGTTTAGAAGTTCTGTTAAGGGGAGG
AGGGAAGGGGACAAGGTCATTGAAGCCAAAGGAATAAAAGATACAGGGACA
GAAACAACAGCAAGATATGTTTAGGGGGAATCTCAGTGTAATACTGTTATTGTG
TGAAGTTCACAACAGGGGCAGGGAGATGGGGCCTTAATGTGAAAAGGCCTTG
TATGTACCATGTTTAAGTTTGAATTTATCTAGGGGAGTCAGGAGAGGAGTTCTA
TGGTGGATTTCCCATCCAACAGGATTAGCAAACTCCCCACATCCAATTAGTCCA
CCTCCTTTTTTTCTGTTTCTTTAACATTGTCTCAGATTCATTCCTTCTGAAATCTA
CCTTATTAGTTCAGGTCTTCTTAAATTTGTACTGGACTGTTCTGTACTCTTGGCT
TTGGTCCTGGGGCTCCAGTTCACCTCTTGCTCCCAACAATCAGTACAGAGACT
ATCAGAGCATCTTCCTTTCCGTAATCTCAACTCTGTTTTCAAAACTTTCCAATA
GTTTATGTGACCTAAGAGGTAGCTGCCTCCCCACCCTTTCAACGCATTCAAGG
CTGCATAATTTAGTCCCAACCCCTTTCAGGTCCTTGACTCACTGTAACAGAGAC
AGAACTCCCATTTGCCTCTTTTGACTGCTTTCTTTGCTTTCGAGGGGCTCTTTC
CTGGAATGATATGTCCATACAAGCTACTGCTGTTGTTCTTACTTTTAGCTCTTTC
TTAGCCCACTGCCAGGTTTGATCGTCACTACTCACCCTGGAAGTAGCCTATTAT

TCCTGGGTTATAGTCTCTTAGCCTTCTATATCTATATCTATATATATATGCATCCCC
AGATAATGTATTGTCTTGAAAAATATACATAAATGGTATACTTTGTCTCCCTCTAT
AAGTTAGTTTTTTCCACTGTCATTTAGTTTTAAGATTTATCCTATTGACATAAATA
GCTATAGTCTTTTCAATTACTGTGTTGCATTTCATTTTATAAATATACATGCAGTA
TTCATCCATTCCTCTAGGGTGCTGAGGCGTATGTATTTGTTTTCAAGGGTTTTGC
TGTACAGTGTTAGCGAATATCCTTAACTAGCTAGTCCTCCTTGTCTTATACAAAT
TTGCAACTTTCAAGATCTGCTTTCATCAACGCTCTATGGAAAGCCTTCCCTGAA
TCCCCAGCCCGGCGGCAAGTCTTGGTACTCAAAGTTGCTTATGCATCTTTGGTA
TCATCCACTACCAAGCACACAGCTGCGCGAAATGTAAAATATTCGCTACGGAT
GAATTGAAATGAATGAATGAATGGGGCAGCACCGCCGTGATGGCTCTATTATTC
CTTTTTAATGGACTTGCGTACTGTCTTTGACAGCTTCTGGTCCTACTCAGAGCA
TTCCTCTAGCTAACAAAGCAGTGTGTGTGGATGTGTGTGTGCCGTTAGCGGGT
GGCGTCTGAACCTCACTGGACCGCCTCCTGCAGTTCGATCCCGGCAAACTCCA
GTGGCAGAGTTCGAGTCCTACCCCCCTCACCTCCACGCCCCTTCTGCACTGGT
CAGGCAGCGAGCCGCTGCAGCCCTGATCCAGTTAGGCTGCCTGCTGGAAGGA
CGGACAGGCCAGTGACTCGTGATCCCTCGCGCGCGGAGCTCGGGCCCGGC
ATATTTCCTGCGGGAAACCCCTGGGTGCCTGCCCAAGGCGGCGGCGGGGCCG
AGGCCGCAACATTGCCAGTAAGGCAGGGGCTTTGCGGTGGGAGGCGGCTGGC
CTTGGGTTACGAGGGCTGCTAAGCTGCACACGCGAAAGTTTCAGCAGGCGGC
GGCTGGAGGCGGCGACCGAGGGAGCGGCAGACTCAGGGGCGCAGGGGAGTC
GAGGGCATTTGCGCCTGTGCTGGACCATGGCGCGCAGGACTGGGCAACGCCA
GGAGGAGGAGAGTGGGGCTCTATCAGGTTATGGATCTGCCAGGCGGCGGCGC
GGCGGCGCGCTGACGGCGGCGGCGGCGGCGGCGGCGGCGGCGGCG
GCGGCGGCGGCGGCGGCGGCGGCGGCGGCGGCGGCGGCGGCGGCG
GCGGCGGCGGCGGCGGCGGCGGCGGCAGCGGCGGCGGCAACGCGGCGG
CGGCGGCAGCGGCTGGCTGGCGGCAGCAACGGCGGCAGCGGCGGCGGC
GGCGGAGGAGGCGGCGGCGGAGGGCGGCGGCGGAGGCGGCGGCGGCGGAG
GCGGCGGCGGAGGCGGCGGCAGGTGGCGGC

F1-IV:15-read 4

AGTTACGTATTGGTTATTTTATCCTATAGGAGTCAGAGAGGTTCTATGGGTAGG

ATTTCCCATCCCAACAGGATTGTAAACTCCACATCCAATTAGTCACACCTCCAT
TTTTTTTTTTCTGTTTCTTTAACATTGTCTCAGATTCATTCCTTCTGAAATCTACC
TTATTAGTTCAGGTCTTTCAATTTGTGCCTGGACTGTTCACCAATTTGACTGGT
CCTGGGGCTCCAGTTCACCTCTGCTCCAACAATCAATTTTCTACAGAACCATCA
GAGCAATCTTCCTTTCCATCAATCTCAACTCTGTCTAAAAACTTTCAATAGTTT
TATGTGACCTAAGAGGTAGAAGCTGCCTCCCACCCTTTCAGCTTGCCATTCAA
GGCTGCACACTCTGTCCCAACTCTTTCCATTCCTTGACCTCTCACTGCCACCAG
AGACAGAACTCCATTTACTTACTCTTTTGACTGTACCTTTTGCTTTCACGGTGG
CTCTTCGAATGATATATTCATACAACTATTATGCTGTTGTTCTTACTTTTACTCTT
TTCAGCCTCCTAATTTGTACCTATAGGTTTGATCACAATTTTACCTCCTGCCCTA
GAAGTAACTAATGTTCTGCTATAGTCTCTTAACATTCTATATCTATATCTATATATG
CTTAATTCACAGATAATGGTATTGTCTTGAAAAAATATACATAAATTTGTATACT
TTTTAGTTCCCTCAAGGACATTAGTTTTTTTCATCATCATTTAGTTTTTAAGATT
TATCCTTATTGACATAAATAGCTATAGTCTTTTTCAATTACGTTGCATTTCATTTC
CGAGTAAATATACTCCACATTATTCATCCATTCCTCTATGTGGCATTTATGTTGTT
TTCAAGTGTTTTTGCTATTACAGTGTTACAAACGAATATCCTTAGCTTAGCTGGT
CTCTTGTTCTATACAAATTTTACTGACCTTTCAAGATCCTGCTTTCATCCCAGCT
CCTCTGGGAAAGCTTTCCCTGAATCCCCCAGCCCACAGCGCAAAGTTCACTCA
AAGTTGCTTGTGCAATCTTTGCATCATCCCACTGCTAAGCACACAGCCACGCC
AGCAAGGCACTTAAGAAATATCGCCAATGGATGAAATCAAAAATGAATGAATG
AATGGGCAGCACCACCCGTGATGGCTCTATTCCTTTTTAATGGACTTTCATGTT
TTGGGCTCACTCTTCTCTCTTGACAGCACTGGTCCTACTCCAGCATTCCTCTAG
CTAGCAAAGCAGTGTGTGATGTGTGGGTGTAGGTGGCGACAAGAGACCACTG
GATGAGAATCGCCTCCTCCTGCAAGTTCACGATCCCGCAACTCCAGTGCTTAG
TTCAGGAGTCCTACCCCACCCCTCACCTCCACGCCCTTCTGCACTGGTCAGCT
AGCGAGCCGCTGCAGCCCTGATCGAGTTAAGGCTGCTGGAGAAGGATGCGGA
TTTGTGCCGCATTCCGTAGACCTCCGCGGAGCTCGGGCCGGCGCTTCTTCCTG
CGGGAAACCCCTGCGCTCCAAGGCGGCGTGGGGGGCTGAGGCTGCGGCGAC
AGTGGGGCGGGGGCCGGTGGTGGGAGGAGGCGGCTGAGGCGGGAAGGGATA
CACGAGGTTGAAAGCTTGCTGCACACCTGAGAAAGTTTAGCTAAATCTGGCG

GTGGCTGAGGTGGCGCGAGGAGCGGCGGACGGGGCGCGGGGAGTCGAGGTG
ATGGTGCCAAAGCTGACTGGGGCGCTGTGCCTGGAGCTTGAAGGCAGAGGAG
AGAGAGAAAGCGGGTTTATCGGGACTTTCTATGGGATTTGCCGCAGGGGTGTG
GTGGTGGTGGTGTGGTGGTGGTGGTGGTGGCGGTGGTGGTGGCGGTGGTGGT
GGTGGCGGTGGTGGTGGTGGTGGTGGCGGGTGGTGGTGGTGGTGGTGGTGTG
CGGTGGTGGTGGTGGCGGCGGTGGTGGTGGCGGTGGTGGCGGTGGCGTGGTG
GCGGTGGTGGTGCGGTGGCGGTGGTGGTGGTGGTGGTGGTGGTGGTGGTGGT
GGTGGTGGCGGTGGCGGGTGGCGGTGGCGGTGGCGGTGGCGGTGGCGGCGG
CGGCGGTGGAGGAGGCGGCGGTGGAGGAGGTGGTGGCGGAGGAGGCGGTGG
AGGAGGCGGCGGCGGAGGAGGCGGCGGCGGTGAGGCGGCGGCGGAGGAGG
CGGC

F2-II:3-read 1

CAGTGATTTTTAAAATTGTATGCAGCCAAGTCACCCTCGTTATCTTTAGTGAGT
AAGGAAGATCGAACTGAAGTAGCTGCTGTGGGTAACTGAAGAAGCAATGTTA
TCTTTCTCTGGTATACTTGCACAGACAAATTCAAAAATATTTGACTACGTTATTT
TGAAAAGGGTTTCCAGTATTTCAATGTGACTACTTTCACTAATGATACTTGCTT
ACCTAAAGTGCTTGTTAACATGCAGTTTTGGAATTTCATGATCTGGCTTACATA
CAACATACTTGCTTAGTGTTGTTTTTACATTCTAGTTCAACATCTATTATGTGATA
GACACTGGGGATTAATAGGCTGTGGTCTTTGCCCATGAGGGACCTCAAAACTT
AGTTGAGAACACAGAAATACAAAGAGTCCTGTGTGAAAATAAGTAGTGGTGT
TAAGATAAAGAAAGCGGCAGTGGAGACGGTGGAGGGACTGTCTGTTGGAGG
AGGAATTTATTGTGGTGTGGCCAAGCCTTCTTTGGACTGAGGATGAATTATGAT
TAGAAGTTCATTAAGAGGAGGAGGGAAGGGGACTACAGAGTCATTCCAAGCC
AAGGAACAAAGATACAGGGACAGAAACAGCAGATATGTTTGGGAGATCTCAA
GTTAGCCAATACTGTTATTGTGTGAGATTCACAACAGGGGCAGGGAGGACAGG
AGATGGGGCCCAATGGCGAAAAGGCCTTGTATGTACCATGTTTAGGAACAGAT
TACATCCTATGAGGTCAGGGAGAGGTTCTATGGGTAGGATTTCCCATCCCAACA
GTTGTAAACTCCCATCCAATTAGTCACCTCCCATTTTTTCTGTTTCTTTTTAACA
TCATAATTCATTCCTTCAAATCTACCTTGGCTGATTCAGGTCTTCCAAATTTGTT
ACTGGACTGTTCACCAACTCTTGACTGGTCCTGGGGCTCCAGTTCACCTCTTG

CTCCCAACAATCAATTTTCTGCAAGAACTATCAGAGCAATCTTCTTTCCATCAA
TCTCAACTCTGTTCAAAACTTTCAATAGTTTTATGTGACCTAAAGAGTGGCACA
CGCCCACCCTTTCAGCTTGCCATTCAAGGCTGCATAATTTGGTCCCAACCCTTT
CCGTCCTTGACTCCTCACTGCTAACGAGACCAGAACTCATTTACTTACTCTTTT
GACTGTGCCTTTAGCTTTCGAGGCTCTTTCCTGGATGATGTATCCATACAACTAT
TGTTGTTGTTCTTACCAGCTCTTTTCTTAGCCCCGCCTTTTAGGTTGGTCTTTTC
TCCTGCCTGGAAGTAACTACTATTCTGGTTATAGTCTCTTAATATCTATATCTATA
TCTATATATATGCATCCACAGATAATGTAGTATTGTCCTTGAAAAAAAAACATAA
ATGGTATGCTTTGTCTCCTCTGCGATTGGTTTTTTCCTATCATTGGTTTTAAGATT
TATCCTAATAGCGTAAATAACATAGTCTTTTTCAATTGCGTGATTTGCATTTCAA
AACTTGCAATACTACATATTATTCATCCATTCCTCTAAGGGTGGTATTTATGTTGT
TTTTCAAGGTTTTACTGTACAATGTTACAACGAATATCCTTAAGCAAGCTGGTC
TCCTTGTCTTATACAAATTTTGCAGCCTTTCAAGATCCTGCTTTCTGTTAGCTTC
TACAGGAAGCCTTCCTGAATCCCCAGCCCACAGCAGCAAAGTCTTGGCCTCA
GGTGCTTGTGCCTCTTTGGTATAATCCTGTAAACACACAGCTACATAAAGTAAG
GCACTTAAAATATTCCTTCCACGGATGAACAAATGAATGAATGAATGGGGCAG
CACCACTCCGTGATGGCTCTATTCCTTTTAATGGACTTTGGCGCCTTGGCCCAC
TTTCCTTCTTTGTGCTGGTCCTACTCAGAGCATTCCTCTAGCTAGCAAAGCAGT
GTGTGGATGGTGTAGGTGGCGATACAGACCTCACTGGATGAGGACCGCCTCTC
CCTGCAAGTTCACGATCGGCAACTCCAGTGCTTGAAGTTCAGGTCTACCCCAC
CCCTCACCTCCACCCCTTCTCTGCACTGGTCAAGCCAGCGAGCCGCTGCAGCC
TGATCGAGTTAAGGCTGCTGGAAAGGATGCGGACAGGGCCGGTAATCATGTCG
ATCCTCCGCATGGGCTGGGCCGGCGCTTCTTCCTGCAGAAACCCCTGGGTGCC
CAGGCGGCGGGGCCGAGGCCGCGGCGACAATGAGCAGGGCTTGCGGTGGGA
GGAGGCGGCTGAGGCGGAAGGACACACGAGGGGCTGCTTGCTGCACCCGAG
AAAGTTTCAGCCAAACTTCGGGCGGCGCAGGGCGGCGGCCGAGGGCGGCAC
GGACTCCGGGGCGCGGGGGTCAGGGCAGCTGCGCCTGTGCTTCGGACCGTAG
CGCCAGGGCCTGAGCACAAACGGGAGGGAGGAAGGTGGGGCTCCTCTATCAG
GACCCCACCTCCCATGTGGATCTGCCCAGGCGGCGGCGGCTTTGGCGGCAGC
GGCGGCGGCGGCGGCGGCAGCGGCGGCGGCGGCGGCGGCAGCGGC

AACGGCGGCGGCGGCAGCGGCACGGCAGCGGCGGCGGCGGCGGCGGCG
GCGGCGGCAGCGGCGGCGGCGGCGGCGGCGGCGGCGGCGGCAGCGGCGGCG
GCGGCGGCAACGGCAGCAGCGGCGGCAACGGCAGAGAAGGCAACGGCG
GAGGAGGCGGCGGCGGAGGCGGCGGCGGAGGGCGGCGGCGGAGGCGGCTT
GGCGGAGGAGGCGGCAGCGGAGGAGGCGGCGGCGGAGGAGGCGCGGCGGC
GGGAGGCGGCGGCGGAGGGCGGCAGCGGAGGGAGGCGGCGGCGGAGGCCC
CGGCAGCGGAGAGGCTGGCGGCGGAGGAGGCGGCGGCGGAGGAGGCGGCG
GCGGAGGAGGCGGCGGCGGAGGGGCGGCAGCGGAGGAGGCAACGGCGGAG
GAGCGGCAGCGGAGGAGGGGCGGCGGCGGAGGAGGAGCGGCGGCGGAGGG
AGGCGGCGGCGGAGGCGGCGGCGGAGGAGGCGGC

F2-II:3-read 2

GTTAAGATAAAGCGGCAGTGGAAGACAGTGGAGGGACTGTCTGTTAGAGGAG
AATTTATTGTGGTGTCGGCCAAAGCCTTCCTTTGGACTGAAGGATGAATTATGA
TTAGAGTTCATTAAGAGGAGGAGGGACTGGAGGTGTTCAAGCAAAGGAACAA
AGATACAGGGACAGAAACAGCAAAGATATGTTTTGGGGGAATCTCAGTTAGCC
AATACTGTTGTGTGTGAAGTTCACAACAGGGGCAGGGAGGACAGAGATGGGG
CCCAATAGTGAAAAGAGCCTTGTATGTACCATGTTGAAGTTTGAATTTATCCTA
TAGGAGTCAGGAAGTTCTATGGGTAGGATTTCCCATCCCATGTGGGTTATAGCT
CCATCCAATTAATTCACCTCCATTTTTTCTGTTTCTTTAACATTCTCCAGATTCC
ATTCCTTCAAATCTACCTTATTAGTTCAGGTCTTCTTAAATTTGTGCCTGAGACT
GTTCACCCAACTCTTGACTGGTCCTGGGGCTCCAGTTCACCTCTTGCTCCCAA
CAATCCAATTTTCTACAGAACTATCAAGAGCAATCTTCCTTTCCATCGTCAACT
CTGTTCAAAAACTTTCCAATAGTTTTATGTGACCTAAGAGGTGTGCCTCCCCAC
CCTTTCAGCTTGCCATTCAAGGCTGCAATTTTAGTCCCAACCCCTTTCAGTCTT
GACTCCTCTCACTAACAGAGACAGAACTCATTTCACCTCTTTTGACTGTACCTT
TGCTTTCACGAGGCTCTTTCCTGGAATGATATGTCCATACAATATTTTGTTGTTC
TTACTTTTAGCTCTTTTAGCCCACTTATTGCGGGACTTTGATCCACACCCCCTAC
TCTCCTGCCCTAGAGTAACTACTATTCTGGTTATAGTCTCTTTAATATCTATATCT
ATATCTATATGTAGCCATCCACAGATAATGTGTAGTATTATCTTGAAAAAAATATA
CATAAATGGTATACTTTGTCCCCTCTGCGATTAGTTTTCACTTATCATTTAGTTTT

AAATTTATCCCTATTGACATAAATAGCTATAGTCTTTTCAATTGCTGTGTTGCATT
TTATTTATAAATATACCATACATTATTCATCCATTCCTCTAAGGGTGAGCATTATAT
TTTCAAAGGTTTTTATATTACAGTGTTACAACGTTATCCTTAGCTTAGCTGGTCT
CCTTGTCTTACTGAATTTTACTGACCTTTCAAGAGTCTGCTTTCATCCCAGCTC
CTCTGGAAGCTTCCCTGAATCCCCCACAGCCTGACGGCAAAAGTCTTGGTACT
CAAAGTTGCTTGACCGTCTTTGTTGTCATCCACTGCCAAGCACACAGCTTTCAT
AAAGTAAGGCACTTAAAATATTCGCTACACGGATGAACAAATGAATGAATAGT
GAACTGCATTTTTCCTCCGTGATGACTCCTATTATTCCTTTTAATGGACTTTGGC
ATGTGGCCCCACTTTCCTTCTTGAGCACTGGTCCCTACTCCAGAGCATTTGTTC
CTCCTAGCTAGCAAAGCAGTGTGTGGATGTGTGTGTGGGGTGTAGGTGGCAAT
CCTGAACCTCACTGGATGAGGACCGCCTCTCCTGCAAGTTCACGATCTGGCAA
ACTCCAGTGCTTGAGTTCAGGTCTACCCCACCCCTCACCTCCACGCCCCTTCT
GCACAGGTCCAGCAGGCCGCTGCAGCCCCTGATCAGAGTTAAGGCTGCTGGA
GAAGGATGCGGACAGGGGCCAGTGACTCGTAGTAGATCCCTCCGCGCGGAGC
TCGGGCCGGCGCTTCTTCCTGCAGGAAGCCCCTGGGTGCCCAAGGCGGCGGG
GCCGAGGGCCACTGCAACGACGAGTGGGGCAGGGCTTGCGGTGGGAGGAGG
CGGCTGAGCGGAAGGACACACGAGGGCTGCTTCGCTGCACACCGAGAAAGT
TCCGGCTGGAAACTTGGGCGGCGGCTGGGCGGCGGCCAAGACGGCGGACTC
AGGGGCGCAGGGAGTCGAGGCATTTGCGCCTGTGCTTTGGACCCGCCGCGCC
AGGGCCTGAGCCTTTGAAGCAGGAGGAGGAGAGAGTGGGGCTCCTCTTCGGG
CCCCTCCCCATGTGATCTGCCCAGGCGGCGGCGGCTGGCAGCGGCAACTGGC
AGCGGCGGCGGCAGCGGCAGCAGCAACGGCAACGGCACGGCAGCAGCGGCG
GCGGCGGCGGCGGCAGCAGCAGCAGCAGCAGCAGCGGCAGCGGCACCGGCA
ACGGCAGCAACAACGGCAACAACAGCAACGGCAACGGCAGCGGCGGCAGCA
GCAACAGCAACGGCAGCAGCAGCAGCGGCAGCAGCAGCAGCGGGAGAGGC
AACGGCGGAGGAGCAACGTGGAGGAGGCGGCGGCGGGAGGAGGCAACAGC
GGAGGAGGCGGCGGCGGAGGGCGGCGGCGGAGGAGGCGGCGGCGGAGGAG
GCGGCGGCGGAGGTGGCGGCGGAGGAAAGCGGCGGCGGAGAGGCGGCGGA
GGGAGCCGGCGGCGGAGGAGGCGGCGGCGGAGGAACGGCAGCGGAGGAGG
CGGCGGCGGAGGAGGGCTGACGGCGGAGGGCTGGCAGCGGAGGAGGCGGC

GGCGGAGGAGGCGGCAGCGGAGGAGGCGGCAGCGGAGGAGGCGGCAGCGG
AGAGCAGCAGCGGAAGGAGGCGCGAGCGGAGGCGGC

F2-II:3-read 3

GTTATCTTTAGTAGTGAAGAGGAAGATCGAACTGTGGTAACACTGTGCCCAGG
TGAAACATGAAAAATAATATTATCTTTTCTCTAAGACATACTTTTGAGACAAAA
TTCCAAAAAATATTTTGACTACGTTGTTTTGAAAGGGTTCCAAGTATTTTCAAG
TGACTACTTTCCTATGATACTTGCCCACTAAAAAGTACTTAGCATTAACATGCA
GTTTTGGAATTTCCTTAGATTCTACCCATGCAAGCTTGCCTTGAGTGTTTGTTTT
CTGGTTCCTTTATCTATTATGTGAAGTGAACACTAAGTTATCGACTGCATGGTCT
TCCTTGTAGGGACCTCAAAAACTTAGTTGAACACAGAAATACAAAGAAGTCCT
GTGAAAAATAAGTAGCCAATTTTAAGATAAAAAAGCGGCAGTAGAAGACAGT
GGAAGGGACTATCTGTTAGAGGAGAATTTGTTGTGGTGTCATGCACTTCTTTG
GACTGAGGATGAATTATGATTAGAAATTCATTAAGAGGAGGAGGAAGGGGACT
ACAGGGGTCATTTCCAAGCCAAAGACAAAAGATGAGGGACAGAAGCAACAA
GACAGATATGTTTTGGGGGAATCTCAAGTTAATAATACTGTTGTGTGAAGTTCA
CAACAGAGCAGGAGGGACAGGAGATGGAGACTAATAAGCGAAAAAGGCCTT
GTATGTTACCATGTTTAAGTTTAGATTTTATCTATAGGAGTCAGGGAGGGAGTTC
TATGGGTGGGATTTCCATCCCAACAGGTTGTAAACTCCCACGTCAATTAGTCCA
CCTCCATTTTTTTCTGTTTTACTTTAACATTGTCTCAGATTTGTTCCAGAATCTAT
TGGTTCAGACTTTGCTTGGTTTGTGCTGGACTGTTCCACTAACCTCTTGACTGG
TCCTGGGGCTCCAGTTTACCTCTTGCTCCCAACAATCAATTTTTCTACAGAACT
ATGAGCATTCTTTGCTTTCCTCAATCTCAACTCTGTTCAAAACTTTCGGACAGA
TTTTATGTGACCTAAAGAGGTAGCTGCCTCCCACCCTTTCAACACCATTCAAGG
CTGCATAATTTGAATTCTTACTTTCAGTCACCAGCTCCTCTCACTGTAACAGAG
ACAGAACTCATTTACTTACTCTTTGGCCTTTACTTTCAGGGGCTACTCCTGGGA
ATGAAATATATATTTGACTGACGACTGTGTTCTGCACTAGCTCTGCCGCCCACC
CAGCCACCCAGGTTTGATCACACCCCCTCTCACCCCTGGAAGTAGCTTGCAAC
CTGGTTATAGTCTCTTAATATCTATCTATATCTCTCCTACTTGCATCACAGATGAC
ATATAAATGTGTCTTGAAAAAAATATACATAAATGGTATACTTTGGCTCTCCCTC
TAGATTAGTTTTCTATCATTTGGATTTTTTAAGATTTATCCCTATGACATAAATAA

GCTATAGTCTTTTCCAATTACTATATTTACATTCATTTATAAATATACATACATTAT
TCATCCATTCCTCTAAGGGTGCAGGCATTTATGTTACTTTCAGGTTTTGCTATTA
CAGTGTTACAAGATATCCTTAAGCAAGCTGGTCTCTCCTTGTCTTATACAAATTT
TACTGACATTTCAAGACCACACTTTCAGCCCGGCTCCTGGGGAAAGCCTTCCC
TAATCCCCAGCCCACAGCCAGCCAGTGCGACCAGGGTTGCTTGCCACATCTGG
TATCATCCCTACCAAGCACACAGCCTCTTGTAAAGTAAGGCACTTAAGAAATAT
TTCGCTTCACGGATGAACAAATGAATGAATGAATGGGGAATGGCTACTCCGTG
ATGGCTCTGTTCATACTTGTCTGCAGACTTTGGCGCCTTATTTCTCCTTCTTTG
ACCTTTGGTCCTACTCAGAGCATTCCTCTAGCCCTAGCAAAGCAGTGTGTGGA
TGTGTGGGTGTGGGTGGCAGTGAACCTCACTGGATGAGGACCGCCTCTCCCTG
CAAGTTCTACAGTCAGCAAACTCCAGTGCTTGAAGTTCAGAGTCTACCCCACC
CCCTCCACCTCCACGCCCCTTCTGCTGAGTCAACCCAACCCAGCCACTGCAGC
CTGATCAGTTAGGCTGCTGGAGAAGGATGCCAGACAGGGCCAGTGACTCATG
TCTAGATCCCTCCACCGACCAGGCCCACTTCTTCCTACAGGGAAGCTTCGATTT
TTAAAGCCATGGGGCCGAGGCCACCCGGCGACAGTGGGGGCAGGGCTTGCAG
TGGGAGGAGGCGGCTGAGGCGGAAGGACACACGAAGACTTCGCTGCACCGA
GAAAGTTCAGCCAAACTGGGCGGCAGCTAGAATAGCAGCCGGGGAACTGGCG
GACTCCGGGACGCAGGGGTCAGGCATTTTGCGCCTGTACCTTTGGACCATGCG
CCAGGGCCTGGCCTTTAGAAACCAGGAGGAGGGGGAGGGGAGGAAATCGGG
CTCCTATCGGGACCCCTCCCCCATGTGGATCTGCCCAGCAGGCGGCGGCGGCG
GCGGCGGCGGCGGCGGCGGCTTTGACGGCAGCAGCGGCAGCGGCGGCGGCG
GCGGCGGCTGACCCGGCAACGGCAACGGCAGCAGCGGCGGCGAAAAGCGGC
GGCGGCAACGGCAACACGACGGCGGCGGCGGCGGCGGCAGCGGCGGCGGCG
GCGGCGGCGGCAACGGCAGCAGCGGCAACGGCGGCAGCAGCTGCTAACAAC
CCAACAGCAGCAACAGCGGCGGCGGCGAAGCAGCGGCAGCGGAGGAAGGG
GCGGCAACGGAGGGCGGCGGCGGAGGAGAGCGGCAGCAGAGGAAGCCTGG
CGGCGGAGGAGGGCGGCGGCGGAGAGCGGCTGTAGAGAGGCAGCAGCGGAG
GAGGCAGCAGCGGAGGAGAAACTGGCATGGCGGCGACGGAGGGCAGCTTGA
CGGAGGAGGCAGCGACGGAAGGCAGCAGCAGAGGAGAGCAGCAGCAGGAG
GTAACGAGGAGGCGAAAGCGGCGGAAGGCGGCGGCGGAGGAGGCGGCGGC

AGAGGAGAGCGGCAGCGGAGGCGGCGGCGGAGGAAGGGACGGCAGCCGGA
GGAGGCGGCAGCGGAGGAATGGCAACGGCGGAGGAGGCGGCAGCAGAAGA
GGCGGC

F2-II:3-read 4

GTCACACCTCCATTTTTTTTTTTCTGTTTCTTTAACATTGTCTCAGATTCATTCCT
TCTGAAATCTACCTTATTAGTTCAGGGTCTTCTTAAATTTCCAGTTTGGACTGTT
CACTAACTCTGACTGGTCCTAGCTCCAGTTCACCCTCTTGCCCCAACAATCAAT
TTTCTACAGAACTATCAGAGCAATCTTCTTTCCCCATCAATCTCAACCTGTTCA
AAAACTTTCAATAGTTTTATGACCTAAGAGGTAGCTGCCTCCCCCACCCTTCAG
CTTGCCATTCAAGGCCGTATAATTTGGTCCCAACCCCTTTCCGTCATTCTTGAC
TCTCCTCACTGCTAACAGAGACAGAACTCATTTACTTACCTTTGGATCGTACCT
TTGCTTTCACGTAGGGCTCTTTCCCTGGAATGATATGTCCATACAACTATTGTTG
CTGTTGTTCTTACTTTTAGCCTTTCAGCCCACTTGTACCCAGGTTTGATCACAC
CCCTACCTCCTGCCTCCCAAAGCAATCACTATTCTGGTTATAGTCTCTTAATATC
TATATCTATATCTATATATATGCATCCACAGATAATGTGTAGTATTGTCTTGAAAA
ATATACACAAATGGTATACTTCTGTTCTCTCATGTATTAGTTTTTTCACTATCATT
TAGTTTTATGATTTATCCTTATTGACATACAAATAGCTATAGTCTTTTTCAATTAC
TGTGTTGCATTTCATTTTATAAAGGAGTACTATACATTATTCATCCATTCTCTAAG
GGTGGGTATTTATGTTGTTTTCAAGGTTTTGCTATTACAGTGTTACAAATTTAAT
ATCCTTAGCTTAGCTGGTCTCCTGTCTTATGTTAAATTTTGCATTGACCTTTCAA
GATCCTGCTTTCATCCCAGCTCCTCTGGGAAAGCCTTCCCTGAATCCCCCAGCC
CACAGCGCAAAGTCTTTGGTACTAAAGTTGCTTGTGCATCTTTGGTATCATCCC
ACTGCTGCCAACACAGCCACGCAGTAAGGCACTTAAGAAATATTCGCCACACG
GATGAACAAATGAATGAATGAATGGGGTGCACCACCGTGATGGCTCTATTCCT
TTTTAATGGACTTATGCCTTGGCCCACTTCTTCTTTGACAGCACTGTCCTACCC
AGAGCATTCCTCTAGCTAGCAAAGCAGTGTGTGGATGTGTGGGTGTAGGTGGC
GATACAGACTCACTGGATATGAGGGACCGCCTCTCCCGGTAAGTTCACGATCC
CGGCAACCCAGTGCTTGAAGTCTAGAGTCCTATCTTCACCTCACCTCCACGCC
ACCTTCTGCACTGGCTGGGTTAGGTGAGCCGCTGCAGCCCTGATCGAGTTAAG
GCTGCTGGAGAAGGGATGCGGATTGCAGCCAGTGACTCGTAGTAGATCCCTCC

GCGAGCCTGGGCCGGCGCTTTCTTCCCGTGGGAACCCCTGGTGCCCAAGGCG
GCGGGGCTGAGGCCGCGGCGACAGTGTGTGGGCGGGCTTGCGGTGGAGGAG
GCGGCTGAGGCGGAAGGATACGAGGTTGCTTCGCTGCACACCTGAGAAAGTT
TCAGCCAAACTTCTGGGCGGCGGCTGAGGCGGCGGCTGAGGAGCGGCGTGG
ACTGGGGCGGGGAGTCGAGGCATTTGCGCCTGTGCTTCGGACCGTAGCGCCA
GGGCCTGGAGCCTTTGGGGCAGGAGGTGAGGGGAGGGAGAGGCCTCTATCGG
GACCCTCCCCATGTGGATCTGCCCAGGCGGCGGCGGCGGCGGCGGCGGCGGC
GGCGGCGGCGGCGGCGGCGGCGGCGGCGGCGGTGGCGTGGCGGCGGCG
GCGGCGGCGGCGGCGGCGGCGGCGGCGGCGGCGGCGGCGGCGCAAGGGGCA
AGGCGGCGGTGGCGGCGGTGGCGGCGGGCGGCGGCGGGTGGCGGCGGCGGC
GGCGGCGGTGGCGGCGGCGGCGGCGGCGGCGGCGGTGGCATGGCGGGTGGC
GGGGTGGCGGCGGCGGCGGTGGCGGCGGGTGGAGGAGGTGGCGGTGGAGGA
GGGGCGGCGGCGGGAGGGAGGTGGCGGTGGAGGAGGGTGGTGGTGGAGGA
GGTGGTGGCGAGGGAGGCGGTGGCGGAGGAGGTGGCGGTGGAGGAGGTGGG
TGGTGGAGGAGGGTGGTGGCGGAGGAGGCGGTGGCGGAGGAGGTGGCGGTG
GAGGGAGGTGGCGGTGGAGGAGGCGGCGGAGGAGGTGGCGGGCGGAGGAG
GTGGCGGTGGAGGAGGGGTGGCGGGTGAGGAGGCGGTGGTGGAGGAGGCGG
CGGCGGAGGAGGCGGCGGCGGAGGAGGCGGCGGGTGGAGGAGGCGGCGGC
GGAGGAGGTGGTGAAGTGAGGAGGCGGCGGCGGAGGAGGCGGC

F2-II:3-read 5

GGAAGGACTACAGGAGTCATTCCAAGCCAAAGGAACAAAAAGATACAGGGA
CAGAAACAGCAAGATATGTTTGGGGGAATCTCAAGTTAGCTAATACTGTTATTG
TGTGAAGTCTACAACAGGCAGGGAGGACAGAGATGGGCCCAATGACTAAAAG
GCCTTGTGGCTGTACCAGTTTAGAAGTTTGAATTTTATCCTATAGGAGTCAGGG
AGAGGTTCTATGGGTAGGATTTCCCATCCCAACAGGATTGTAAACTCCTCACAT
CCAATTAGTCACACTCATTTTTTTTTTTTTCTGTTTCTTTAACATTGTCTCAGAT
TCATTCTTCTGAAATCTACCTTATTAGTTCAGGTCTTCAAATTTGTGCCTGACTG
TTCACCAACTCTTGACTGGTCCTGGGCTCCAGTTCACCTCTTGCTCCTAACAAT
CAATTTCTACAGAACTATCAGAGCAATCTTTCCTTTCCATCAATCTCAACTCTG
TTCAAAAAAATCTCACAGTTTTATGACCTAAGAGCAGTAGCTGCCTCCCACCC

TTTTCAGCTTGCCATTCATACGTATAATTTGTCCCAACCCCTTCATTCTTGACTC
TCCTCACTGCCAATAGAGACAGAACTCATTTACTTACTCTTTGACTGTACCTTT
GCTTTCACGAGGGCTCTTTCCGGAATGATATGTCCATACAACTATTGTTGTTGTT
CTTACTTTATTTTTCAGCCCACTTGTACCCAGGTTTGATCACACCCCTACTCTCC
TGCCCCCAAAGAATTGCTACTTATTCTGGTTATAGTCTCTTAATATCTATATCTAT
ATCTATATATATGCAATTCACAGATAATGTGTAGTATTGTCTTGAAAAATATACAC
AAATGGTATACTTTGTCTCCCTCTCATGTATTAGTTTTTCACTATCATTTAGTTTT
TAAGATTTATCCCTTGGCTGACATAAATAGCTATAGTCTTTTTCAATTACTGTGT
TGCATTTTCATTTTATAAATATACCACACAGGTCTATCCATTCCTCTAAGGGTGG
GCATTTATGTTGTTTTCAAGGTTTTACATACAGTGTTACAACGTAATATCCTTAG
CTTAGCTGGTCTCTGTCTTATACAAATTTTACTGACCTTTCAAGATCCTGCTTTC
ATCCCAGCTCTGGGAAAGCCTTCCCTGAATCCCCAGCCCACAGCAAGTCTTGG
TACTCAAAGTTGCTTGTGCATCTTGGTATCATCCCCACTGCTACACACAGCTAC
GCAAAGTAAGGCACTTAAGAAATATTCGCTACACGGATGAACAAATGAATGAA
TGAATGGGGCAGTACCACCTGTGACATTTATCTTTTAATGGACTTTAAGTGCCT
TGGCTCTTTGACAGCACTGGTCTCACTCCAGAGCATTCCTCTAGCTAGCAAAG
CAGTGTGTGGATGTGTGGGTGTAGGTGGCGATACAGACCTCACTGGATGAGGA
CCGCCTCCTGCAAGCTCCACGATCCCGGCAACCCAGTGCTTATAGTTCAGATC
CTACCCCACCCCCTCACCTCCACGCCCCCTTCTGCACTGGCCGGGTTTAGCGA
GCCGCTGCAGCCCTGATCGAGTTAAGGCTGCTGGAGGCGATGCGGACGGGGC
CAGTGACTCGTAGTAGATCCTTCCGCGCGGAGCTCGGGCTGGCGCTTCTTCCT
GCGGGAAACCCCCTGCGCCCAAGGCGGCGGGGCTGAGGCCGCGGCGACAGT
GGGGCGGGGCTTGCGGTGGAGGAGGCGGCTGAGGCGGAAGGACACGAGGTT
GCTTCGCTGCACACCTGAGAAAGTTTCAGCCAAACTTCGGGCGGCGGCTGAG
GCGCGGCGGCCGAGGAGCGGCGGACTCAGCGCGGGGAGTCGAGGCATTTGC
GCCTGTGCTTCGACCGTAGCGCCAGGGCCTGGAGCCTTGAAGCAGGAGGAGG
GGAGGAGAGAGGGGCCTCTATCGGGACCCTCCCCATGTGGATCTGCCCAGGC
GGCGGCGGCGGCGGCGGCGGCGGCGGCGGCGGCGGCGGTGGTGGCGGT
GCGGGCGGGGTAGTAGTGCAGGTGGCGGCGGCGGCGGCGGGCGGGTGG
TGGCGGCGGCGGCGGTGGCGGCGGTGGCGGCGGTGGGTGGTGCGG

CGGCGGCGGTGGCGGGTGGCGGTGGCGGTGGCGGCGGTGGCGGGCGGTGGC
GGCGGTGGTGGCGGCGGCGGCGGCGGCGGTGGCGGTGGCGGTGGCGGTGGC
GGTGGTGGCGGCGGGAGGAGGTGGCGGCGGGAGGAGGCGGGCGTGGCGGAG
GGGAGGGTGGCGGCGGAGGGAGGTGGCGGCGGAGGTGGCGGTGGAGGGAG
GGTGGCGGCGGGAGGAGGCGGCGGCGGAGGAGGCGGCGGGTGGAGGAGGC
GGCGGCGGAGGGAGGGCGGCGGCGGAGGAGGCGGCGGCGGAGGAGGCGGT
GGCGGAGGAGGCGGCGGCGGAGGAGGCGGCGGTGGAGGAGGCGGCGGCGG
AGGAGGCGGCGGCGGAGGAGGCGGCGGCGGAGGAGGCGGTGGCGGCGGAG
GAGGCGGCGGTGGAGGAGGTGGCGGCGGAGGAGGCGGCGGCGGAGGAAGG
AGGAGGCGGCGGGCGGAGGAGGCGGC

F4-II:2-read 1

GTGGTGTCGGGCAAAGCCCTCTCTTTGACTGGAAGGGATGAATTATGATTTTTC
AGGAAGTTCATTAAGAGGAGGAGGAAGGGGACTACAGGAGTCATTCCAAGCC
AAAGGAACAAAAGATACAGGACAGAAACAGCAAGATATGTTTGGGGGGGAA
CTCAAGTTAGCTAATACTGTTATTAAAAAGAAGTCTACAACAGGGGCAGGGAG
GACAGGAGATGGGGCCCAATGACTAAAGCGGCCTTGTATGTACCATGTTTAGA
AGTTTGAATTTTATCCTATAGAGTCAGAGAGGTTCATGGGTGGATTTCCCATCC
CAACAGGATTGTAAACTCCCACATCCAATTAGTCACACCTCCATTTTTTTTTC
TGTTTCTTAACATTGTCTCAGATTCATTCCCTTCTGAAATCTACCTTATTAGTTC
AGGTCTTCTTAAATTTGTGCCTGGACTGTTCACTCAACTCTTGACTGGTCCTAA
ATCTAGTTCACCCCTTGCTCCCAACAATCAATTTTCTACAGAACTATCAGAGCA
ATCTTCCCTTTCCATCAATCTCAACTCTGTCTAAAAAACTTTCAATAGTTTTATG
TGACTTAAGAGGTAGCTGCCTCCCCACCCTTTCAGCTTCTTGCCATTCAAGGC
CGTGAAGCAATTTGGTCCCAACCCCTTCTTCCGGCCCTTGACTCTCTCACTGCT
AACAGAGACAGAACTCATATTTACTTACTCTTTTGACTTTCCGTACCTTTGCTT
TCACGAGGCCCTTCCTTGGAATGATATGTCCATACAACCATTGTTGTTGTTCTTA
CTTTAGCTCTTTTCTTAGCCCACTTGTACCCAGGTCGCGATCACACCCCTACTC
TCCTGCTCTGAAGTAACTACTATTCTGGCGATAGTCTCAATATCTATATCTATATC
TATATATGCATCCACAGATAATGTGTAGTATTGTCTTGAAAAAAATATACATAAA
TGGTATACTTGTCTCCCTTCACATTAGTTTTTTTCATCACTATCATTTGTTTTTTA

AGATTTATCCTTATTGACATAAATAGCTATAGTCTTTTTCAATTACTGTGTGTTGC
ATTTCATTTTATAAATATACTATACATTATTCATCCATTCCTCTCATATAAGTGGGC
ATTTATGTTGTTTTCAAGGTTTTGCTAGCTACAGTAAGTTACAACTAATATCCTT
AGCTTAGCTGGTCTCCTTTTGTCTTATACAAATTTTACTGACCTTCAAGATCCTG
CTTTCAGTCCTGGCTCCTCTGGAAAGCCTTCCCCTGAATCCCCCAGCCCACAA
AGTTCAGTACCAAAGTTGCTTGTGCATCTGGTATCATCCCACCGCGTTGCCATT
AATAGCCACGCAAAGTAAGGCATTTAAGAAATATTCTCACATGGATGAACAAA
TGAATGAATGAATGGGGGCAGCACCACCCGTGATGAAGCTCTATTCCCTTTTTA
ATGGACTTTATGCCTTGGCCCACTTTCCTCTTTGACAGCACTGGTCCTACTCAG
GAGCATTCTCTCTAGCTAGCAAAGCAGTGTGTGGATGTGTGTGTAGGTGGCGA
TACAGACCACTGGATGAGGACCGCCTCCCCTGCAAGTTCACGATCCTGGCAAC
CCAGTGCTTCAGTTCAGAGTCCTACCCACCCCCCCACCTCCACGCCCCTTCTG
CACCGGTCGCTTGCGGAGCCGCTGCAGCCTCTGATCGAGTTAAGGCTGCTGAG
AAGGATGCGGACGGCCAGTGACTCGTAGCAGATCCCTCCGCGCGGAGCTCAC
GGCGCTCTTTCCTGCGGGGAAACCCCTGGGTGCCCAAGGGCGGCGGCTGAGG
CCGCGGCGACAGTGGGCGGGGCTTGCGGTGGGAGGAGGCGGCTGAGGCGGA
AGGACACACGAGGCTGCTTCGCTGCACACCTGAGAAAGTCAGAAACTGGCG
GCGGCGAGGTGGCGTGCCGGTGGAGCGTGGACTTTGTGCGGGGGAGTCGAG
GTATTTGCCTGTGCTTTCGGGAAGTGCTGTGGCTTGAGCTTGAAGCAGGAGGA
GGGGAGGAGGGTTTTCTATCGGGACCCCTATGTGGATCGCCCAGGTGGTGGTG
CGGTGGCGGTGGTGGCGGCGGTGGCGGCGGCGGGTGGTGGCGGGTGGTGGT
GGTGGTGGTGGTGGTGGTGGTGGTGGTGGTGGTGGTGGTGGTGGTGGTGGTG
GTGGTGGTGGCGGTGGTGGTGTGTGGTGGGTGGTGGTGGTGGTGGTGGTGGT
GGTGGTGGTGGTGGTGGTGGCGGGTGGCGGTGGTGGTGGTGGCAGGCGGGTG
TGGTGGTGGTGTGGTGCGGTGGTGGTGGCGGGGCGGTGGCGGTGGCGGTGGC
GGTGGTAGTGGCGGCGGCGGCGGGTGGCGGTGGCGGCGGGCGGCGGCGGCG
GC

F4-II:2-read 2

GGGCAGATTCTATCCCAACAGGATTGTAAACTCCCACATCCACCCTAGTCACA
CCTCCATTTTTTTCTGTTTCTTTAACATTGTCTCAGATTCATTCCTTCTGAAATCT

ACCTTATTAGTTCAGGTCTTTAAATTTGTGCCTGACTGTTCACCAACTTGACTG
GTCCTTGGGGCTCCAAAGTTCACCTCTGTTGCTCCCCAACAATCAACTCTTTCT
ACAGAACTATCAGAGCAATCTTCTCCTTCCAGCATCAATCTCAACTCTGTTTCA
AAACTTTCATTAGTTTTATGTGACCTAAGAGGTAGCTGCCTCACCCTTTCAGCT
TCGCTATCCAAGGTTGCATAATTTGGTCCCCAACCCCTTCCGCCCTTGACTCCT
CACTGCTAACAGAGACAGAACTCATTTACTTACTCTTTGACTGTACCTTTGTTT
ACGAGGCTCTTTTCCTGAATGATATGTCCATACAACTATTGTTGTTGTTCTTACT
TTTAGCCTTTTCTGAAATTACTTGTACCCAGGTTTGATCACACCCCCTACCTCCT
GCTCCTGGAAGTAACTACTATTCTGGTTATAGTTCTTACTATCTATATCTATATCT
ATATATGCATCCACAGATAATGTGTAGTATTGTCTTGAAAAAATATACATAAATG
GTATACTTTATTCCCTCTGTATTAGTTTTTTCACTATCATTTAGTTTTTTAAGATTT
ATCCCTATTGGATATAAATAGCTATAGTCTTTTCAATTACTGTGTTGCATTTCATT
TCATAAATATACCATACATTATTCATCCATTCCTCTAAGGGTGGGCATTTATGTTG
TTTTCTAAGGTTTTGCTATTACAGTTACAACTAATGTTCCTTAGCTTAGCTGGTC
TCCTTGTCTTATACAAATTTTACTGACCTTTCAAGATCCTGCTTTCATCCCAGCT
CCTCTGGGAAAGCCTTCCTGAATCCCAGCCCACAGCGGCAGTAAAAGTCTTG
GTACTGTGCTGCTTGCATCTTTTGGTATCATCCTAATGTTAAGCAATACGCTACG
CAAAGTAAGCGTTAATTTAAGAAATATTCACACATCGGATGAACAAATGAAGA
ATGAATGGGGGCAGCACCACCCGTGATGGCTCTATCTTTTAATGACTTTATGCC
TTGGCCCACTTTCCTTTTGACAGTACTGTCCTACTCCAGAGTATCTCTAGCTAG
CAAAGCAGTGTGTGTGGATGTGTGGGTGAGGTATATACAGACCTCATTACGAG
GACCGCCTCCCTGTAAGTTCACGATCCCGGCAACCCAGTGCTTGAAGTTCAGA
GTCCACCCCACCCCTCACCTCCACGCCCTTCTGTACCATCAAGCAGAGCCGCT
GCAGCCCTGATCGAGTTAAGGCTGCTGGAGAAGGATGCGGACGGGCCAGTGA
CTGTAGTAGATCCCTCTGCGCGGAGCTCGCCGCGCTTTCTTCCCGCAGGGAAA
CCCCTTGGTGCCCAAGGTAAGGGGCTGGAGCGTGGCGACAGGGAAGGGGCTT
GCGGTGGGAGGAGGCGGCTGGAGGCGGAAGGACATGAGGTTGCCGCTGCAC
CCGAGAAAGTTTCAGCCAAACTTCGGGCGGTGGTTGAGGCGGCGGGCCGAGG
AGTGCGACTTCGGGGCGCGGGAGTCGAGGATGGTGCCTGTGGGCTGGACTGT
AGCGCCAGGGCCTGGAGCTTTTGAAGCAGGAGGAGGGGAGGAGAGAGGGTT

TCTATCGGGACCCTTCCCTATGGATTCGTTTCAGGTGGCGGCGGTGGCGGTGC
GGTGGTGGCGGTGGTGGTGGCGGTGGTGGCGGCGGCGGTGGTGGCG
GTGGTGGCGGGTGGTGGCGGCGGTGGTGGTGGCGGCGGCGGTGCGGCGGCG
GCGGTGGCGGCGGCGGTGGTGGTGGCGGTGTGGCGGGTGGCGGTGGCGGCG
GTGGTGGTGGTGGCGGGCGGTGGCGGCGGCGGCGGCGGTATGGTGCGGC
GGCGGCGGCGGCGGTGGCGGCGGCGGCGGGTGGTAAGTGCGCGCGCGC
GGTGGCGCGGCGGCGGCGGCGGCGGCGGCGGCGGCGCAGGCGGCGGC

<u>F4-II:2-read 3</u>

GACTACAGGGGTGTTCCAACCAAAGAGACAAAGATGCAAGACAGAAACAAC
CAGTTATGTTTGGGGAATCTCAGTTAGCTGATACTGTTGTTGACAGTTAAACAG
GGCAAGGAGGACAGGAGATGGGCCCCAATGAAACCTTATATATGCCATGTTTA
GAAGTTTAGATTTTATCCTGTAGAGGTCGAGGGAGAGGGTTGTGGTTCCCACA
TCCCAACAGGTTATAAGCTCCACGTCAATTGATCACACCTCCATTTTTTTCTATT
TCTTAACATTGCTCCTCAGATTCATTCCTTCTAAAATCCCTACCTTATTAGTTCA
GATCTTCTTAAATTTATTGCCTGGACTGTTCACCCAACTCTTCAGCTGGTCCCT
GGGGGCTCCAGTTCCTGCCTCCCCCAACAATCAATTTCTACAGAACTATCAGA
GCAAATCTTCCACTCCATCAATCTCAACTCTGTTCAAAAACTTTCCAATGGTTT
ATACTTGACCTAAGAGTGGCTACCTCCCACCTTTCAGCTTTGCCATTCAAGGCT
GCATAATTTTGGTCCCAACCCCTTTCCGTCACTTGACTCCTCCTGCTAACAGAG
ACAGAACTCATTTCGCCTCTTTTGAGCTGTACCTTTGCTTTCTGGGGCTCTTTC
CTGGAATGATCATGTCCTTCAACTATTTATTTGTTGTTCTTACTTTTTAGCTCTTT
TCTTGGCTGCTTGCTGATTTTAATCACCCCCTCTCCTGCCCTGGGAGTAACTGC
TATTCTAGTTATAGTCTCTTTTAATATCTATATCTATATCTATATATAGCCGTCCAC
AGATAATGTGTAGTATTATCTTGAAAAAAATATACATAAATACTTGTATACTTTGT
CTCCCTCTAGATTAGTTTTTTCCATATCATTTTGTAGTTTTTTAAGATTTATCCTAT
TATTGACATGAAATAGCTATAGTCTTTTTCAATTCTGTGTGCATTTCATTTTATAA
ATATACACAGCATTATTCATCCATTCCTCTAAGGGGTGAGGCATTTATGTTATTT
CAAAGTTTTGCTGACTTACAGTGTTACAAAAATATCTTCCAACAACTGGTCTCC
TTGTCTTATGCAAATTTTACTGTTATACCAAAGGATCCTGCTTTCATCCAAGCTC
CTCTAGGAAAGTAACCCTAAGATCCCCCAATAATGACAAGTCTTAGTATAAAGT

TGGCAGCCATCTTTAGTATCCATCACTACCAAACACAGCTACACCAAATGAGC
ACTTAAGAAATATTCACTACAGATGAACAAATAGATGAATAGATGGGGCAGCA
CCATCCGTGATAAATATTCCTTTTTAATGGACTTTGGCGCCTTGGCCCTTTCATG
TGACAGCACTGGTCCTACTCAGACATTCCTCTAGCTAACAAATGGTGGCCAAC
TGGATGTGTGGGTGTAGTGGCGTCTGAACCTCACTGGATGAGGACCATACCTC
TCCTGCAAGTTCCACGATCCAGCAGCTCAGTGGCCGGGATTCAGAGTCCTGCC
CACCCTGCCTCCACGCCCCTTCTGCACTGGTCAAGCCCAGCAGGCCACTGCA
GCCTGATCGAGTTAAGGCTGTACTGGAAAGGATGCGGACAGGGCCAGTGACT
CGTAGTAGATCCTCACTTTGGAGCTCAGGCAGCCTTCTTCCTGCAGGGAAACC
CCTGGGTGCCCAAGGAGGCAGCGGGGCCGGGCCACGCAACGACAATCAGGA
AGACAGGGCTTGCAGTGGGAGGGAGGCGGCTGGGGCGGGGAAGGGACTACA
CGGGCTGCTTCGCTGCACACCGAGAGAGATTTCAGCCAAACTTCCGGGCGGC
AGCTGGGGCAGCAGCCGAGGCCGGCACAGACTCAGGGCATGAGGAGTCAAG
GGCATTGCCTGGCTGGTTCCCGCCATGGCGCAGGGCCTGAGCCTTTGAAGTAG
GGAGGAGGAGATGGGGCTCCTCTATCAGGACCCCACCATGTGGATCTGCCCAG
GCGGCGGCGGCGGCGGCAGCAGCGGCAGCAGCGGCGGCTGGCGGCGGCGGC
AGCGGCGGCGGCTAACGGCAACAGCACCAGCAACAACGGCGGCGGCGGCGG
CGGCAGCAGCAGCAGCAGCAGCGAGCAGCAGCAACGGCAGCAACAGCAGCA
GCAGCAGCGGCGGCAGCAGCAACAGCAACAGCAGCAACAGCAACAACAGCA
ACCGGCAACAGCAACAGCAACAACAACAGCAACAGCAGCAGCAGCAGCAAG
CAGCAGCAGCAGCGGCAGCAGCGGCGGCAGCAGCAGCAGCAGCAGCAGCAG
CAGCAGCAGCTTGTGGCAGCAGCGGCGGCGGCAGCAGCAGCAGCAGCAGCA
GCAGCAGCAGCAGCAGCAGCAGCGGC

F4-II:2-read 4

GAAGACAGTGGAGGACTATATATTAGAGGAGAATTTATTGTGGTGTGACTGAC
CTTCTCTTTGGACTGAGAAAAATTGAATTATGATTAAGTTCATTAGAGGAGGAG
GGAAGGGGACTACAGAGTCATTCAAGCCAAAGACAAAAGATACAGGGACAG
AAACAGCAAGATATGTTTGGGGGAATCTCAGGATTGGCAATACTACATTATTGT
GAAGTTCACAACAGGGGCAGGAGGACGGAGATGCAGAATCTCAGTTCTATGG
GTGAAGTTTCCATCCCAACAGGATGTAAACTCCCATCCAATTAGTCACCTCATT

TTTTTCTGTTTCTTTAACATTGTCTAGATTCATTCCTTCCCAAAATCTGTAATAGT
TCAGGTCTTCTTAAAATTTTGCCTGGACATTCACCAACTCTTGACGCCTGATTT
TCCAAGACCAGTTGCTCTTGCTCCCAGCAATCAATTTTCTACAGAACTATCAGA
GCAATCTTCTTTATCAAGTCTCAACTCTGTTCAACTTTCAATAGTTTTATGTGAC
CTAAGTGGCTGCCTCCCCCACCCTTTCACGCTTTGCAATTCAAGGCTGCATGA
ATTTGATTATTGCCTAGGTCCTTGACTCCTCTCACTGCTAACAGAGACAGAAGC
TCATTTGCCTCTTGACTGTTACCTTTTTGCTTTCCACGAGGCTCTTTCCTGGAAT
GATATGTCCATACAAGCATTATTTATTTCTTGCCTGCTCTTTTCTTATTTTTGTAC
CCAGGTTTGATCCACCCCCTACTCTACATGGAGTAACACTATTCTGGTTATAGT
CTCTTATTTATCATATCATATCTATATATATATACATCCACAGATAATGTAGTATTAT
CTTGAAAAAAAATATACATAAATGGTATCTTGTCTCACTATAGATTAGTTTTCAC
TTCTATAAATTTAGTTTTAAGATTTGTCTAATACAGAGCATAAATAGCTATAGTCT
TTCTCAATTACTGTGTTGCATTCATTTATAATATACCATACATTGTTAATGCATTC
CTCTAGAGGTGGGGCATTTATGTTGTTTTCAAGTTTTGCTATTACAGTGTTACA
ACGAATATCCCTTATAAATAGCTGGTCTCCTTGTCTTATACAAATTTTACCCCGA
CTTTCGAGATCCTGCTTTCATCCCAGCTCCTCTGGGGAAAGCCTTCCCTAGATC
CCTGACTGGCAGCAAAGTCTCCTCAAAAATTTGCTTGCATGCATCTTTGGTATC
ATCCACTATAATACAGCTGCATAAAGTGAGCCACTTAGAAATATTCTTCCACGG
ATGAACAAATGAATAGATGAATGGGCCAGCACCACTCCGTGATGGCTCTATTC
CTTTTTAATGGACTTTGGCGCCTTGGCCCACTTTCCTTCTTTGACAACTGGTCC
TACTCCAGGCATTCCTCTAGCTAGCAAAGCAGTGTGGATGTGTGTGGGTGTGG
GTGGCGATACGAACCTCACTGGATGAGGGACCGCCTCCTGCAGGTTCACGATC
CCAAACTCCAGTGCTTGAAGTTCAGGGTCCTACCCCACCCCTCTCCGCCCCTT
CTACTTTGTGGTCAAGCCAGCAGGCCGCTGCAGCCCTGATCGAGTTAAGGCTG
CTGGAGAAGGATGTTGGACAGGGCAATGACTCGTAGTAGATCCCTCGCGCGG
GCTCGGGCCGGCGCTTCTTCCTGCAGGAAACCCCCTGGGTGCCCAAGGCAGG
GGCCGAGGGCCACTTGACGACAGTGGGAAGACAGGCTTACCTGCAGTGGGA
GGGAGGCGGCTGAGCTGAAGGACACACAGGAAAGCTGCTTCGCTACACTGA
GAAAGTTTCAGCCAAACTTCGGGCGGCGGCTGGGGCGGCAGCCGAAGACGG
CGGACTCAGGGCGCAGGGGAGTCGAGGCATTGCGCCTGTCGCTTCGGACCGT

AGCGCGGGGCTGGGCGCTGGAAGGCAGGAGGAGGAGGAGAGTGGGGCTCCT
TATCGGGACCCCCTCCCCGTGGATCTGCCCAGGCGGCGGCGGCGGCGGCGGC
GGCGGCGGCGGCAGCGGCGGCGGCGGCGGCGGCGGCGGCGGCAGCGGCGGC
GGCGGCAGCGGCAACGGCAGCAGCTGGCCCGGCAGCGGCAGCGGCGGCGGC
GGCGGCAGCGGTGGCTGGCGGTGGTGGCGCGGCGGCCCGGCAACGGCAACG
GCGGCAGCGAAGGCGGCGGCGGCAGCAGCGGCGGCGGCGGCGGCGGCA
ACGGCAGCAGCGGCGGCAGCGGCGGCGGCGGCTGCGGCGGCGGCGGCGGCG
GCAACGACAGCAGCGGCGGCGGCGGCAACGGCAGCGGCGGCGGCAGCGGCA
GCGGCAACGGCAGCAACAGCAACAGCAGCAGCAGCAGCGGCAGCGGCAGCG
GCAGCGGCAGCGGCGGCGGCAGCGGCGGCGGC

<u>F4-II:2-read 5</u>

GAGTCAGGGAGAGGGTTCTATGGGTTGCAGTTCCCATCCCAACAGGATTATAC
AAGCTCCCACATCAATTAGTCCACCTCCATTTTTTTTTCTGTTTCTTTAACATT
GTCTCAGATTCATTCAACTTCTAAATCTACCTTATTAGTTCAAGTCTTCTTAAAT
TTGTACTGGACTGTTCACCAACTCTTGACTGGTCTGGGGCTCCAGTTCACCTC
TTGTAACAATCAATTTTCTACAGAACTATCAGGTAGTCTTTGCTTTCCATCAATC
TCAGCTCTGTTCAAAAACTTTCCAATAGTTTATGTGACCTAAGAGGTAGCTGCC
TCCCCACTTACTTTCAGCTTGCCATTCAAAGGCTTGCATAATTTGTGATCCCAC
AACCCCTTTTCCGTCCTTGACTCCTCTCCTGCTAACAGAGACAGAACTCATTTT
ACTTACTCTTTTGACTGTACCTTTGCTTTCCCGAGGCTCTTTCCTGGAATGATAT
GTCCAAAAGCCCCCAACTGTATTGTTATTTCTTACTTTAGCTCTTTTCTTAGCCC
ACTTGTACCCAGGGTTTGATCCACCCCTACTCTCCTGCCTGGGAGTAACTACTA
TTCTGGTTATAGTCTCTTAGCCTCTATATCTATGTCTATATGTGCATCCACAGATA
ATGTGTAGTATTGTCTTGAAAAAAATATACATAAATGGTATGCTGTCTCCTAGAT
TAGTTTTTCACTATCATTTTGGTTTTTAAGATTTATCCTATTGACATAAATAAGCT
ATAGTCTTTTCAATTACTGTGTTGCATTTCATTTTACTAAATATACCATACATTAT
TCATCCATTCCTCTAAGGGTGGGCGTTGCAGCAACATTTTCAAGGTTTTTACTA
TTACAGTGTTGTGAATATCCTTAAAAACAGCTTTTGAGTCTCCTTGTCTTATACA
AATTTGCCCCAGCCTTTCGAATCCTGCTTTCATCCCAACTCCTCTGGAGAAGCG
CTTCCTGGTCCCCCAGCCCACAATGACAAAATCTTGGTACTCCAAAAGTGTAT

GCATCTTTGGTATCATCACTGCCAAGCTTACACAGCTACATAAGTAGGCAGCAA
AATATTCGCTACACGGATGAACAAATGAATGAATGAATGGGCAGCACCACTCA
CGTGATGGCTCTATTCCTTTTAATGGACTTTGGCGCCTTGGCCCACTTTCCTTCT
TGACAGCACTGGTCCTACTCCAGAGCATTCCTCTAGCTAACTTCGAAAGCAGT
GTGTGGATGTGTGGCGAATTGTAGGTGGCGATACAGACCTCACTGGATGGGGA
CCGCCTCTCCTGCAAGTTCACGATCGAAGAACTCCAGTGGCAGGTTCGAGTCC
TCCACCCCTCACCTCCGCCCCTTCTGCACTGGGTGAAGCTGGCGAAAGGCTGC
TGCAGCCTGATCCGATTTAAGGCTGCTGGGAGGAAGGGATGCGGACGGGGCC
AGTGACTCGTAGTAGATCCCTCCGCGCGGAGTCGGGCCAGCGCTTCTTCCTGT
GAGAAACCCCTGGGTGCCCAAAGGCAGCAAAGACCCGAGGGCCGCGGCTCA
ATGGGACAGGGCTTGCGGTGGGAGGGAAGCGGCTGAGACGGAAGGGACCCC
ACACGAGGCTGGCCTTCACTGCCGGAAGGTTCAATGAAACTTCCGGGCGGCG
GCTGACGGCAGCCGAGGGCGGCGGCGCGGACTCAGGGGCGCGAGGTCGGGC
ATTTGCGCCTGTGCTTCGGACCGTAGCGCCAGGGCTTCTGAGCCTTTGGAAGC
AGGAGGAGGAGAGAGTGAGAGGGCTCCTCTATCAGGACCCCCCTCCCCATGT
GGATCACAGGCGGCGGCGGCGGCATGCAGCAGCGTGGCAGCAGCAGCAGCG
CGGCAGCCAGCGGCAGCAGCAGCGGCGGCGGCAGCTGGCAGCGGCAGC
GGCGGCGGCGGCAGCGGCGGCGGCGGCGGCGGCGGCGGCGGCGGCAGCAGC
GGCGGCGGCGGCGGCGGCGGCAACGGCGGCAACGGCAGCGGCGGCAGCAGC
GGCGGCGCGGCGGCGGCGGCGACGGCGGCGGCGGCGGCGGCGGCGGCGGCG
GGCGGCTGGCGGCGGCACGGCGGCGGCACGGCGGCAACGGCAGCGGCAGCA
GCAGCGGCAGCAGCAGCAACGGCAGCAACAGCAACGGCAGCAGCAGCAGCA
GCGGCAGC

F4-II:2-read 6

GGCAGGTCTATGCCTATACTGACTTGTACTTGCTTAGTGTTGTTTACATTCTAGT
TCAACATCTATTGCGGATAGGACACTCGGGGGATTAATAGGCTGTGGTCTCTTG
CCCACAGAGACCCCGTTAAAACTTAGTTGAGAAACACAGGAAACAAAGAGTC
CTGTGTGAAAAATAAGTATTGGTGTTAAGATGCAGTGAAGACAGTGGAGGGA
CTGTCTGTTAGAGGAGGAATTTATTGTGGTGTCGGCAAAGCTCTTGGATTGAA
GGATGAATTATGATTAGAAGTTCATTACAGAGGAGGAGGGAAGGGGACTACAG

GAGTCATTCTGAAGCCAAAGGAACAAAAGATACAGGGACAGAAATAGCAAGA
TGTTTGGGGGAATCTCGTTACTACTGTTATTGTGTGAAGTTCACAACAGGGGTA
TAGGGAGGACAGGATGGGGTCCAATGACTAAAAGGTCTTGTATGTACCATGTT
TAGAAGTTTGAATTTTATCCTATAGGGAGCTAGGAGAGGTTCTATGGGTAGGAT
TTCCATCCCAACAGGATTGTAAACCCCACATCCAATTAGTTTCACCTCCATTTT
TTTTTCTGTTTCTTTAACATTGTCTCAGATTCATTCCTTCTGAAATCTACCTTAC
GAATTCAGGGTCTTCTTAAATTTGTGCCTGGACTTCACTAACTCTTGACTGGTC
CTGGGGGGCTCCAGTTCACCTCTTGCTCCCAACAATCAATTTTCACAGAACTAT
CAGAGCAATCTTCCTTCCATCAATCTCAACTCTGTTCAAAATCTCAATAGTTTT
ATGTGACCTAAGAGGTAGCTGCCTCCCACCCTTTCAGCTCTGCCATTCAAGGC
TGCATAATTTGGTCCCACCCCTTCCGGCCCTGACTCACTGCTAATAGAGACAGA
ACTTCATTTACTTACTCTTTTGACTGTACCTTTGCTTTTACTTAGGGCTCTTCCT
GGAATGATATGTCCATACAACTATTGTTACATTTTACTTTAGCTCTTTTCTTAGCT
CATTTGTACCCAGGTTTGATCACAATTCCCCCTACTCTCCTGCTCCTGAAGTAA
CTACATTATTCTTATTATAGCTTTTAGTTATTATATTAGGTTATAGGTGTACCATGG
AGCATGTGTAGTATTGTCTTGAAAAAAATATACATAAATGGTATACTTTTGTCTC
CCTCACATTAGTTTTTTCTTACTATCATTTAGTTTTTTAAGATTTATCCCCATTGG
AGTGTAAATAGCTATAGTCTTTTCAATTACTGTGTTGTACTTCCTCATTTTTATAA
ATATACCATACATTATTATCCATTCCTCTAAGGGTGGGCATTTATGTTGTTTCTCA
AGGTTTTGCTATTACAGTGTGTTACAACGACATCCTTAGCTTAGCTGGTCTCCT
TGTCTTATACAAATTTTACTGACCTTTCAAGATCCTGCTTATCCCAGCCCTCTGG
AAAGCCTTCCCTGAATCCCCCAGCCCACAGCGCCAGCGGGTAAAGTCTGGTA
CTCTGTGCTGCTTGTGCATCTTTGGTATCATCCCACTGCTAAGCACACACAGCT
ACGCAAAGTAAGGCACTTAAGAAAATATTCGCTACACGGATGAACAAATGAAT
GAATGAATGAAGCAGTACCACTCCGTGATGGCTCTATCTTTAATGGACTTTGGC
GCCTTTGGCCCACTTTCTCCTTCTTGACAGCACTGGTCCTACTCCAGAGCATTC
CTCTAGCTAGCAAAAGCAGTGTGTGGATGTGTGGGTGTAGGGTGGCGATACAG
ACCTCACTTCGGATGAGGACCGCCTCCCTGCAAGTTCACGATCCCGGCAACTC
CAGCGCTTGAAGTTCAGGAGGTTCCACCCCCACCCCCTCACCCACGCCCCTTC
TGCACTGGTCAAGCCATGAGCCGCTGCAGGCCCCTGATCGAGTTAAGGCTGCT

GGAGAAGGATGCGGACGGGGCCGCGACTTCGTAGTAGATCCCTTCCGCGCGA
GCTCGGGCCGGCGCTCTTCCCGTGGAAACCCCTGGGTGCCCAAGGCGGCGTG
CCGAGGCTGCGGTGGCGACAGTGGGGCGGGGCTTGCGGTGGGAGGAGGTGG
CTGAGGCGAAGGACACACGAGGTTGCTTCGCTGCACACCTGAGAAAGTTTCA
GCCAAACTTGGGCGGTGGTGGCGGCTGAGGCGGCGGCTGAGGAGCGGCGGG
ACTCAAGGGGGAGTCGAGGTATTTGCGCCTCGTGCTTCGGACCGTAGCGCTAG
GCCTGAGCCTTTGAAGCAGGAGGGAGGGGAGGAGAGAGTGGGGCTCTCTATC
GGGACTTTCTCATGTGGATTCGCCCAGGTGGCGGTGGCGGGTGGGCGGCGGC
GGTGGCGGCGGCGGGTGGTGGCGGTGAAAAGTGGCGGCGGCGGCGGTGGCG
TGGCGGCGGCGGCGGCGGCGGTGGCGGCGGCGGCGGCGGCGGCGGTGGTGG
CGGCGGTGGCGGTGGCGGCGGCGGTGGTGGCGGTGGCGGCGGGCGGTGGCG
GTGGCGGCGGCGGCGGTGGCGGCGGGCGGCGGCAGTGGTGGCGGCGGTGGC
GGTGGCGGTGGCGGCGGCGGTGGCGGCGGCGGCGGCGGCGGCGGCGGTGGT
GGCGGCGGCGGCGGCGGCGGCGGCGGCGGCGGCGGTGGCGGCGGCGGCGGC
GGGTGGCGGC

F5-II:1-read 1

GGCAGGTCTATGCCTATACTGACTTGTACTTGCTTAGTGTTGTTTACATTCTAGT
TCAACATCTATTGCGGATAGGACACTCGGGGGATTAATAGGCTGTGGTCTCTTG
CCCACAGAGACCCCGTTAAAACTTAGTTGAGAAACACAGGAAACAAAGAGTC
CTGTGTGAAAAATAAGTATTGGTGTTAAGATGCAGTGAAGACAGTGGAGGGA
CTGTCTGTTAGAGGAGGAATTTATTGTGGTGTCGGCAAAGCTCTTGGATTGAA
GGATGAATTATGATTAGAAGTTCATTACAGAGGAGGAGGGAAGGGGACTACAG
GAGTCATTCTGAAGCCAAAGGAACAAAAGATACAGGGACAGAAATAGCAAGA
TGTTTGGGGGAATCTCGTTACTACTGTTATTGTGTGAAGTTCACAACAGGGGTA
TAGGGAGGACAGGATGGGGTCCAATGACTAAAAGGTCTTGTATGTACCATGTT
TAGAAGTTTGAATTTTATCCTATAGGGAGCTAGGAGAGGTTCTATGGGTAGGAT
TTCCATCCCAACAGGATTGTAAACCCCACATCCAATTAGTTTCACCTCCATTTT
TTTTTCTGTTTCTTTAACATTGTCTCAGATTCATTCCTTCTGAAATCTACCTTAC
GAATTCAGGGTCTTCTTAAATTTGTGCCTGGACTTCACTAACTCTTGACTGGTC
CTGGGGGGCTCCAGTTCACCTCTTGCTCCCAACAATCAATTTTCACAGAACTAT

CAGAGCAATCTTCCTTCCATCAATCTCAACTCTGTTCAAAATCTCAATAGTTTT
ATGTGACCTAAGAGGTAGCTGCCTCCCACCCTTTCAGCTCTGCCATTCAAGGC
TGCATAATTTGGTCCCACCCCTTCCGGCCCTGACTCACTGCTAATAGAGACAGA
ACTTCATTTACTTACTCTTTTGACTGTACCTTTGCTTTTACTTAGGGCTCTTCCT
GGAATGATATGTCCATACAACTATTGTTACATTTTACTTTAGCTCTTTTCTTAGCT
CATTTGTACCCAGGTTTGATCACAATTCCCCCTACTCTCCTGCTCCTGAAGTAA
CTACATTATTCTTATTATAGCTTTTAGTTATTATATTAGGTTATAGGTGTACCATGG
AGCATGTGTAGTATTGTCTTGAAAAAAATATACATAAATGGTATACTTTTGTCTC
CCTCACATTAGTTTTTTCTTACTATCATTTAGTTTTTTAAGATTTATCCCCATTGG
AGTGTAAATAGCTATAGTCTTTTCAATTACTGTGTTGTACTTCCTCATTTTTATAA
ATATACCATACATTATTATCCATTCCTCTAAGGGTGGGCATTTATGTTGTTTCTCA
AGGTTTTGCTATTACAGTGTGTTACAACGACATCCTTAGCTTAGCTGGTCTCCT
TGTCTTATACAAATTTTACTGACCTTTCAAGATCCTGCTTATCCCAGCCCTCTGG
AAAGCCTTCCCTGAATCCCCCAGCCCACAGCGCCAGCGGGTAAAGTCTGGTA
CTCTGTGCTGCTTGTGCATCTTTGGTATCATCCCACTGCTAAGCACACACAGCT
ACGCAAAGTAAGGCACTTAAGAAAATATTCGCTACACGGATGAACAAATGAAT
GAATGAATGAAGCAGTACCACTCCGTGATGGCTCTATCTTTAATGGACTTTGGC
GCCTTTGGCCCACTTTCTCCTTCTTGACAGCACTGGTCCTACTCCAGAGCATTC
CTCTAGCTAGCAAAAGCAGTGTGTGGATGTGTGGGTGTAGGGTGGCGATACAG
ACCTCACTTCGGATGAGGACCGCCTCCCTGCAAGTTCACGATCCCGGCAACTC
CAGCGCTTGAAGTTCAGGAGGTTCCACCCCCACCCCCTCACCCACGCCCCTTC
TGCACTGGTCAAGCCATGAGCCGCTGCAGGCCCCTGATCGAGTTAAGGCTGCT
GGAGAAGGATGCGGACGGGGCCGCGACTTCGTAGTAGATCCCTTCCGCGCGA
GCTCGGGCCGGCGCTCTTCCCGTGGAAACCCCTGGGTGCCCAAGGCGGCGTG
CCGAGGCTGCGGTGGCGACAGTGGGGCGGGGCTTGCGGTGGGAGGAGGTGG
CTGAGGCGAAGGACACACGAGGTTGCTTCGCTGCACACCTGAGAAAGTTTCA
GCCAAACTTGGGCGGTGGTGGCGGCTGAGGCGGCGGCTGAGGAGCGGCGGG
ACTCAAGGGGGAGTCGAGGTATTTGCGCCTCGTGCTTCGGACCGTAGCGCTAG
GCCTGAGCCTTTGAAGCAGGAGGGAGGGGAGGAGAGAGTGGGGCTCTCTATC
GGGACTTTCTCATGTGGATTCGCCCAGGTGGCGGTGGCGGGTGGCGGCGGC

GGTGGCGGCGGCGGGTGGTGGCGGTGAAAAGTGGCGGCGGCGGCGGTGGCG
TGGCGGCGGCGGCGGCGGCGGTGGCGGCGGCGGCGGCGGCGGCGGTGGTGG
CGGCGGTGGCGGTGGCGGCGGCGGTGGTGGCGGTGGCGGCGGGCGGTGGCG
GTGGCGGCGGCGGCGGTGGCGGCGGGCGGCGGCAGTGGTGGCGGCGGTGGC
GGTGGCGGTGGCGGCGGCGGTGGCGGCGGCGGCGGCGGCGGCGGCGGTGGT
GGCGGCGGCGGCGGCGGCGGCGGCGGCGGCGGCGGCGGTGGCGGCGGCGGCGGC
GGGTGGCGGC

F5-II:1-read 2

GGGGACTACAGGAGCTGGTCTCCAAGCCAAAGGAAACAAAAAAGATAGGGA
CAGAAACAGCAAGATATGTTTGGGGAATCTCAAGTTAGCTAATACTGTTATTGT
GTGAAGTTCAAATAGGGTAGGGAGCAGGTGAGATGTGGGGCCCAATGACGAA
AAGGCCTTGTATGTACCATGTTTAGAAGTTTTGGAATTTTATCCTATAGGAGTCA
GGGAGAGGTTCTATGGGTAGGATTTCATCCCAACAGGATTGTAAACTCCCAGC
ATCCAATTAGTCACACCTCCATTTTTTTTTCTGTTTCTTTAACATTGTCTCAGA
CTATTCCTTCTGGAAATCTACCTTATTATTAGTTCAGGTCTTCTTTAAATTTGTGC
CTGGACTGTTCACCAACTTGACTGGTCCTGGGGCTCCAGTTCACCTCAAGTTC
TTAACAATCAATTAATTTTTCTACAGAACTATCAGAGCAATCTTCCTTCCATCAA
TCTCAACTTGTTCAAAAAACTTTCAATAGTTTTATGTGACCTAAGAGTAGCTGC
CTCCTCACCCCTTTCAGCTTGCCATTCAAGGCTGCATAATTTGTCCCAACCCCT
TTCCGTCCTTCTTGACTCCTCTCACTGCTAACAGAGACAGAACTCATTTACTTA
CTTCTTTGACTGTACCCCTTGCTTCTCACGAGGGCTCTCCTTCCTAAATGATATG
TCCATACAACTATTGTTGTTGTTCTTACTTTTAGCTCTTTTCTTAGCTCCACTTGT
ACCCAGGTTTGATCACACCCCTACTCTCTCTGCCCTGAAGCACCATTATTCTGT
TATAGTCTCTTAATATCTATATCTATATTCATATATATGCATCCACAGATAATGTGT
GGTATTGTCTTGAAAAAATACATAAATGGTATACTTTGGTCTCCCTCTTATTAGT
TTTTTCACTATCATTTAGTTTTTAAGATTTATCCCTTATTGGACATAAATAGCTAT
AGTCTTTTTCACTACTGTGTTGCATTTCATTTTATAAATATGTACCATACATTATT
CATTCATTCCTCTAAGGGTGGGCATTTATGTTGTTTCTGCTGTTTCTGTTATTAC
AAAAAGTTACAACTAATATCCTTAGCTAGTTGGTCTCTTGTCTTATGGGCCAAA
TTTTTACTGACCTTTCAAGATCCTGCTTTCATCCCAGCAGCTCCTCTGGGAAAA

GCCTTCCCTGAATCCCCAGCCCACAGCTGCAAAAGTCTTGGTACTCAAAGTTG
CTTTGTGCATCTTGTATCATCCCACTGCTACACACAATAGCCACGCAGAAAGTA
AGGCACTTAAGAAATATTCGCCACGGATGAACAAATGAATGAATGAATGGGGC
ATACCACTCCGTGATGGCTCTATTCCTTTTAATGGACTTCATGCCTTGGCCCACT
TCTTCTCCTTCTTTTTGACAGCACTGGTCCTACTCCAGACATTCCTCTAGCTAG
CAAAGCAGTGTGTGGATGGGGTGGGGGTGTAGGGTGATACAGACCTCACTGA
CGAGGACCGCCTCCCTGCAAGTTCACGATCCCGGCAACCCAGTGCTTGAAGC
TCAGAGTCCTACCTCAATTCCCCCTCACCTCCACGCCCTTCTGCACTGGTCGAC
AGTGAGCCGCAGCCCCTGATCGAGTTAAGGCTGCTGGAGAAGGATGCGGACG
GGGCCAGTGACTCGTAGTAGATCCCTCCGCGCGGAGCTCGGGCCGGCGCTTCT
TCCTGCGTGGAAACCCTGGTGCCCAAGGCGGCGGAAGCCGAGGCCGCGGCG
ACAGTGGGGCGGGGCTTGCGGTGGGAGGAGGCGCTGAGGCGGAAGGACACA
CGAGGCTGCTTCTTGCCGCAATACCACCCGAGAAAGTTTCAGCCAAACTTCGG
GCGGCGGCTGAGGCGGCGGCCGAGGAGCGGCGGACTTTTCGGGGCGCGGGG
AGCGAGGCATTTGGTGCCTGTGCTGGACCGTGCGCCTGTGCTCTGAGCTTTGG
GGCAGGAGGGGAGGGAGAGAGTGGGCTTCCTTATCGGGACCCCTCCCTATGT
GGACTCGCTTCCAGGCGGCGGCGGTGGCGGCGGCGGCGGCGGTGGCAAGGT
GGTGGCGGTGGCGGCGGCGGCGGCGGCGGTGGTGGCGGCGGCGGCGGGCGG
CGGTGGCGGCGGTGGCGTGGCGGCGGCGGCGGCGGCGGCGGCGTGGCGGCG
GCGGCGGCGGCGGCGGCGGTGGCGGCGGCGGCGGCGGCGGCGGCGGCGGTG
GCGGCGGTGGCGGTGGCGTGGCGGCGGCGGCGGCGGCGGCGGCGCGGCGGC
GGCGGCGGCGGCGGCGGCGGC

F5-II:1-read 3

GTGGAGGGACTGTCTCTGTTATTAGAGGGAGGAATTTATTATTGTGGTGTCGGC
CGTGGTCTTTGGACTGAAGGATAGTTATGGTAGAAGTTCATTAGAGGAGGAGG
GAAGGGGACTACAGGAGGGTCATTCAGGCAAAGGAACAAGATACAGGGACC
CAGAAACAGCAAGATGTGTGGGGGAATCTCAAGTTAGCCAATACTGTTATTGT
GTGAAGTTCACAACAGGGCAGGGAGGAGAGGACAGGAGATGGGGCCCAATG
TGAAAAGACCTTGTATGTACCATGATTTGAATTTTTATCCTATAGGAGTCAGGG
AGAGGTTCTATGGGTAGGTTTACATCCCAACAGGATTGTAAACTCCCATCCAAT

TATAGTCACACCTCCATTTTTTTTCATTTCTTTAACATTGCATCTCAGATTCATTC
CTTCTGAAATCTACCTTATTAGTTCAGGTCTGTAGGTTGTGCTGGACTATTTCAT
AACTCTTGACTGGTCCTGGGCTCCAGTTCACCTCTTACTCCAACAATCAATTTT
CTACAGAACTATCAGAGCAATCTTCCTTTCCATCAATCTCAACTCTGTTCAAAA
ACTTTCAATAGTTTATGTGACCCTAAGAGGTAGCTGCCTCCCCACCTTTCAGCT
TGCCATTTCAAGGCTGCATAATTTGGTCCCAACCCCCTTTCCGTCCTTGACTCC
TCTCCTGCTAACAGAGACAGAACTCATTTTACTTACTCTTTTGACTGTACACCT
TTGCTTTCCACGAGGGCTCTTTCCTGGAATGATATGTCCATACAACTGTGTGTT
GTTGTTCTTGCCTGGCTCTTTCTTGGCCCACTTGTACCCAGGTTTGATCACCCC
TACTCTCCTGCCCTGGGTAACTGCTATTCAGTTATAGATCTTAGCTATCACCATC
TATATCTCTATATATAACCATCTTTAGATAATGTGTAGTATTGTCTTGAAAAAAAT
ATACATAAATGGTATACTTTGTCTCCCTCTCTGCGATTAGTTTTTTTCACTATCA
TTTTAGTTTTAAGATTTATCCCTATTGACATAAATAGCTGGTCTTCCAATTACTGT
GTTTTTCATTTTATAAATATACCATACATTGTTACATCCATTCCTCTAAGAGGTGG
GCATTTATATTTATTTTCAAAGGTTTGCTATTACAGTGTTACAACGAATATCCTTA
GCTTAGCTGGTCTCCTTGTCTTGCCTAAATTTTACCCCACTGACCTTTCAGATC
CTGCTTTCATCCCAGCTCCTCTGGGAAAGCCTTCCCTGAATCCCCCAGCCCAC
AGCAGCAAAGTCTCTTGGTGCTGCTCAAGTTGCTTGTGCATCTTTGGTATCATC
CCTGCCAAGCACACAGCTACATAAAGTGTTACTTAAAATATTAGCCGTTGACA
AATGAATGAATGGGGCAGCACCACTCCGTGATGGCTCTATTCCTTTTGGCTTTG
GCGCCTTGGCCCACTTTCCTTCTTTGACAGCACTGGTCCCTACTCCAGAGCATT
CCTCTAGCTAGCAAAGCAGTGTGTGGATGTGTGTGGGTGTGGGTGGCGATATA
CAGACCTCACTGGATGAGGTGCCTCTGCAAGTTCACGATCCAGCCCAAACTCC
AGTGCTTGAGTTCAGATTACACCCACCCCCTCACCTCCACGCCTTCTGCACTG
GTCCAAGCCAGCGAGCCGCTGCAGCCTAGTCGAGTTAAGGCTGCTGGAGAAG
AAGGATGCACGGACAGGGCCAGTGGCTCGTAGTAGTCCGCGCGGGAGCTCGG
GCGGCACTTCTTCCTGCAGGAAACCCTGGGTTGCCCAAGGCGGCGGGGCCGA
GGCCACGCGGCAATGGGGCAGACTTGCGGTGGGAGGAGGCGGCTGAGGCGG
AAGGACACGAGGCTGCTGGCTGCACCCGAAAGTTTCAGCCAAACCGGGCGG
CGGCTGGGCGGCGGCCGAAGACGAGCTCAGGGCGCGGGGAGTCGAGGCATG

CCTGCGCTGCGGACCGTAGCGCCAGGGCCCTGAGCCTTTGAAGCCGCAGGAG
GAGGTGAGACGTCTCGGTTCCTCATGTGGATCTGCCCAGGCGGCGGCGGCGG
CGGCGGCGGCGGCGGCAGCGGCGGCAGCGGCGGCGGCAACGGCGCGGCGGC
GGCAGCGGCGGCGGCGGCAGCGGCGGCGGCGGCGGCGGCGGCGGCGGCGGC
AGCGGCGGCGGCGGCGGCGGCGGCGGCAGCGGCAGCAACGACGGCAACGGC
AGCAGCAGCAGCAGCGGCAGCAGCGGCGGCAGCAGCGGCAACGGCGATGGC
TGCAACGGCAGCAGCGGCGGCGGCGGCGGC

<u>F5-II:4-read 1</u>

GAAGACAGGAGGGACTGGTCTGTTAGAGGAGGAATTTATTGTGGTGTCGGCA
AAGCCTTCTTTGACTGAAGGATGAATTATGATTGTAGTTCATTAAGAGGAGGA
GGGAAGGGGACTACAGGAGTCATTCCAAGCCAAAGGAACAAAAGTATTACAG
GAGAAACAGCAAGATATGTTTGGGGGAACTTGCCAGCCAATACTGTTATTGTG
TGAAGTCTGGCTACCAGGCAGGAGGACACAGAGATGTAGTCTAATGACTTAA
AAGCAAGGCCTTGTATAAAAATTCCATGTTTAGAAGTTTGAATTTTATCCCATA
GGAGCTGTGAGAGGTTCTATGGGTAGGATTTCCCATCCCAACAACAGGATTGT
AAACTCCCACATCCAAGAATTAGTCACACCCCCATTTTTTTTTTTCTTGCTTTT
AACATTGTCTCAGATTCATTCCTTCTGAAATCTACCTTATTAGTTCAGGCTCAAT
TTGTGCCTGGACTGTTCACTCAACTCTTGACTGGTCCTAACGCAGTTCACCTCT
TTGCTCCCAACAATCAATTTTCTACAGAACTATCAGAGCAATCTTCCTTTCCAT
CAATCTCAACTCTGTCTGGGCAACTTTCAATAGTTTTATGTGACCAAGAGGTG
AAGCGTTTCACCCTTTCAGCTTAAGCATTCATATGCTGCATAATTTGTCCCAAC
CCTTCTCGTCCTTGACTCTCATTGCTAACAGAGACAGAACTCATTTATTTACTC
TTGATTGTACCTTTGTTTACGAGGGCTCTTTCCCTGAATGATATGTCCACAACTA
TTGTTGTTGTTCTTACTTTTAGCTCTTAGCCCACTTGTACCCAGGTTTGATCACA
CCCCCCACTCTCCTGCCCTGGAAGTAACTACTATTCTGGTTATAGTCTTCAATAT
CTATATCTATATCTATATATATGCATCCACAGATAATGTGTAGTATTGTCTTGAAA
AACATAAATGGTATACTTTGTCTTCATGTATTAGTTTTTTTTCACTATCATTTAGT
TTTTAAGATTTTATCCCTATTGACATAAATAGCGGCGTCTTTTCATAATTACTGTG
TTGCATTTCATTTTATAAACATACCACATTATCTATCCATTCTCTAAGGGTGGCAT
TTATGTTGTTTTCTGGCTGTTTTGCTATTACAGGTGTTAGCAAACGAATATCCAG

TTTAGCTGGTCTCCTTTGTTCATGCCAAATTTTACTAATCTTTCAAGATCCTGCC
ATCCCAGCCTCTGGGAAAGCCTTCCCTGAATCCCAGCCCAGCGCAAAGTCTTG
GTACTCAGTTGCTGGCAGTGCATCTTTTGGTATCATCCCACTTGCTACACAGCG
CACACAAAAGTAAGGCACTTAAAGAAACCACGGATGAACAAATGAAGAATGA
ATGGAAGCAGCACAACGGGTGATGGCTCTTATTCCTTTTAATGGACTTTGGCG
CTCTTGGCCCAATTTCCTTCTTTTGACAGCACTGGTCCTACTCCAGAGCACATT
CTCTAGCTAGCAAAGCAGTGTGTGGATGTGTGTAGGTGGCGATACAGACCTCA
CTGATGAGATCTGCCTCTCCCTGCAAGTCTACGATCCAACCCAGTGCCAGTTC
AGAGTCCCACCCACCCCCACCCACGCTCCCTTCTGCACTGGTCGCTTTCAGCG
AGGGGGCGCAGCCCCTGATCGAGTTAAGGCTGCTGGAGAAGGATGCGGACGG
GGCCAGGACTGTAGTAGATCCTCCGCGTGCGGAGCTCGGGCCGGCGCTTTTTC
CTGCGGGAAACCCCTGGTGCCCAAGGCGGAAGCTGAGCGCCGCGGCGACAG
GGCGGGGCTTGCGGTGGAGGAGGCGGCTGAGGCGGAAGGACACACGAGGCT
GCTTCGCTGCACAATTCCGAGAAAAGTTTCAGCCAAACTTCGGGCGGCGGCC
GAGAGGCGGCGGCCGAGGAGCGGCGGACTCTGGGGCGCGGGGGAGTCGAGG
CATTTAGGTGCCTGTAAGCTTGACCGTAGCGCCAGCCTGAGCTTTGAAGCAGG
AGGAGAGTGGGGCCTCTACTGGGACCCCCATGTGGATCTGCCCAGGCGGTGG
TGGTGGTGGTGGTGGGTGGGTGGTGGTGGTGGGTGGTGGTGGTGGTGGTGGT
GGTGGTGGTGGTGGGTGGTGGTGGTGGTGGTGGGTGGGTGGGTGGTGGTGGT
GGTGGTGGTGGTGGGTGGTGGTGGTGGTGGTGGTGGTGGTGGTGTGGCGGGT
GGTGGTGGGGTGGTGGCGGCGGTGGTGGTGTGGCGGTGGCGGTGGCGGCGGT
GGCGGTGGCGGCGGCGGTGGCGGTGGCGGCGGGTGGCGGCGGCGGCGGGCG
GCGGCGGCGGCGGCGGCGGCGGCGGCGGCGGCGGCGGCGGT

F5-II:4-read 2

GGATGGTAGATGGGGGCCCAATGACTTAAAAGGCCTTTGTATGTACTATGTTTA
GAAGTTTGAATTTTATCCTATAGAGTCAGAGAGTTCTATGGGTAGGATTTCCCA
TCCCAACAACAGGATTGTAAACCCCACATCCAATTAGTCACACCTCCATTTTTT
TTTTCTGTTTCTTTAACATTGTCTCAGATTCATTCCTTCTGAAATCTACCTTATTA
GTTCAGGTCTTCTTAAATTTGTGCCTGGACTGTTCACCAACTCTTTGACTGGTC
CTGGGGCTCCAGTTCACCTTGCTCCCAACAATCAATTTTCTACAGAACTATCAG

AGCAATCTTCCTTTCCATCAATCTCAACTCTGTTCAAAAACTTTCAATAGTTTT
ATGCACCAAGAGGTAGCTGCCTCCCCCACCCCTTCAGCTTGGCTATCTCAAGG
CTGCATAATTTGTCCCAACTCCCCTTCCGGCCCTGACTCCTCTCACTGCTAACA
GAGACAGAACTCATTTACTTACTTCTTTTGACTGTACCTTTGCTTTCACGTAGG
GCTCTTTCCTGGAATGATATGTCCATACAACTATTGTTGTTGTTCTTACTTTTAG
CTCTCTTTCTTAGCCCACTTGTACCCAGGTTTGATCACACCCCCTACTCTCCTG
CCCTGGAAGTAACTACTATTCTGGTTATAGTCTCTTTAATATCTATATCTATATCT
ATATATATGCATCCACAGATAATGTGTAGTATTGTTCAAAAAAAACATACTACAA
ATGGTATACTTTTGTCTCCTCTTATGCATTAGTTTCTTCACTATCATTAGTTTTAA
GATTTATCCCTATTGGAGTACAACAGCTATAGTCTTTTCAATTACTGTGTTGCAT
TTCATTTTATAAATATATCTATACATTATTCATCCATTCCTCTAAGGGTGGGCATTT
ATGTTGTTTTCAAGGTTTTGCTATTACAGTGTGTTACAACTAATATCCTTAGCTT
AGCTGGTCTCCTTGTCTTATACAAATTTTACTGACCTTTCAAGATCCTGGCTTAT
CCCAGCTCCTCTGGGAAAGCTCTCTTCCCTGAACTTCAGCCTAAGCGGTAAAG
TCTTTGGTACTCAAAGTTGCTTTGTGCATCTTGCATCATCCCACTGCTACACAC
AGCTACGCAGCAAGGCACTTAAGAAATATTCGCTACACGGGATGAACAAATGA
ATGAATGAATGGGGCAGCACCACCTGTGATGGCTTCTATTCCTTTTAATGGACT
TTTGGCGCCTTGGCCCATTTTCCTCTTTGACAGCACTGTCCTACTCCAGAGCAT
TCCTCTAGCTAGCAAAGCAGTGTGTGGATGTGTGGGTGTAGGTGGCGATACAG
ACTCATCCTCGGATGAGGACCGCCTCTCCCTGCAAGCTCACGATCCCGCAAAC
TCCAGTGCTTGAAGTTCAGAGAGTCCACTCCCCACCCCCTCACCTCCACGCCC
TTCTGCACTGGCTGCTTGCTGTGAGCCGCTGCAGCCCTGATCGAGTTAAGGCT
GCTGGAGAAGGATGTGATCAGCCAGTGACTCGTAGTAGATCCCTCCGCGCGGA
GCTCGCCGGCGCTTCTTCCTGCGGGAAACCCTGTGCCCAAGGCGAGGAAGGA
GGCCGCGGCGACAGGGGCGGGGCTTGCGGTGGAGGAGTAAGGCTGAGGCGG
AAGGACACACGAGGTTGCTTCTGCTGCACCTGAGAAAGTTTCGCGCCAAACT
TCGGGCGGCGGTTGAGGCGGCGGCCGAGGAGCGGCGGACTGGGGCGCGGGG
GAGTTGAGGCATTGCGCCTGTGCTTCGACCAAAGTGCCAGGGCCTGAGCTTTG
AAGCAGGAGGGAGGGGAGGAGAGAGAGGTTTCTCTATTGGACCCTCCCATGT
GGATTCGGGTTTGTGTGGCGGTGGCGGTGGCGGTGGTGGCGGGTGGTGGCGG

TGGTGGTGGTGGTGGTGGTAGTGGTGGGTGTGGTGGTGGTGGTGGTGGT

GGTGGTGGTGTGGTGGTGGTGGTGGTGGTGGTGGTGGTGGTGGTGTGGT

GGTGGTGGTGGTGGTGGTGGTGGTGGGTGGTGGTGGGTGGTGGTGGTGG

TGGTGGGTGGTGGTGGTGGTGGTGGTGGTGGTGGTGGTGGTGGTGGTGG

TGGTGGTGGTGGGTGGTGGTGGTGGTGGTGGTGGGTGGGTGGTGGTGGT

GTGGTGGCGGGTGGGTGGTGGTGGTGGGTGGTGGTGTGGTGGTGGTGGT

F9-II:6-read 1

GGTAATAGTCTTTGCCCATGAGGACCTCAAAACTTAATTGAGAACACAGAAAT

ACCAAAAAGTCCTGTGTGAAAAATAAGTATTGGTGTTAAGATAAAGAAAGCTG

ACAGTGGAAGACAGTGGAGGACATCTGTGGGAGGAGGAATTTATTGTGGTGT

AAGCAAAACCTTCTTTGGACTGAAGGATGAATTATGATTAGAAGTTCCATTAA

GAGGAGGAGGAAGGGGACTACAGTCATTCAAACCAAAGGAACAAAAGATAC

AGGGACAAAAACAGCAAGATATGTTTGGGGAATCTCAAATTAGCCAATACTGT

TATTGTGTAGAAGTTCTAACAGGGGCAGGAGGACAGGAGATAGGACCCCAATA

GCCAGAAAAAAGGCCTTGTATGTACCATGTTTAGAAGTTTGAATTTTATCCTAT

AGGAGTCAGGGAGGTTCTATGGGTGGGATTTTCCCATCCCAACAGGATTGCTT

AACTCCACATCAATTAGTACACCTCCATTTTTTTTTGTTTCTTTAACATTGTCTC

AGATTCATTCCTTCTGAAATCTACCTTATTAGTTCAGGTCTTCTTAGAATTTGTA

CCTGGACTGTTCCACCAACTCTTGAGCTGGTCCTGGGGCTCCAGTTTACTCTG

CTCCCAACAATCAATTTTACCTACCAAGAACTATCAGGCAATCTTCCTTCCATC

AATCTCAACTCTGTTCAAAAACTTTCAATGGTTTATGTGACCCTGAAGTGTTGC

CTCCCCACCCTTTCAGCTTACCATTCAAGGCTGCATAATTTGGTCCCAACCCCC

CTTTCCGTCCTTGACTCCTCTCACTGCTAACAGACAGAACTCATTTGCCTCTTT

TGACTGTACCTTTGCTTTCACGAGGGGCTCTTTCTGGAAATGATATGTCAGCAC

AACTATGTTGTTGTTCTTATAGCTCTTTTCTTGTACTTGTACCCAATTTGATCAC

CCTACTCTCCTCACCCTGGAAATGCTACTATTCTGGTTAACAGTCTCTTATATCT

ATATCTATATCATATATACCATCCACAGATAATGTGTAGTATTGTCTTGAAAATAT

ACATAAATAAAAGGCATACTTTTATCTCTCTCCTAGATTTAGTTTTTCACTATCAT

TTGGTTTCAGATTTATCCCTATTGACATAAATAAGCTATAGTCTTTTTCAATTACT

GTGTTGCATTTTCATTTTTCAAATATACCATACATTATTCATCCATTCCTCTAAGG

GTGGGCATTTTATGTTGTTTTCAGGGTTTTGCTATTACAGTGTTACAACTTGAAT
ATCCTTAGCTTAGCTGGTCTCCTTGTCTTATACAAATTTTACTGACCTTTTCAAA
GATCCTGCTTTTCATCCAGCTCCTCTGGGAAGCCCTTCCCTAGATCCCTGACCC
ACAGCTGACAGTACTCAAAGTTGGCACGGCATCTTTGGTATCATCCTGCCAAG
CACACAGCCTACTACGCAAAGTAGGCGCAGAAATATTCCTTCACGGATGAACA
AATGAAGAAATGAATGAATGGGGCAGCACCCTTCCGTGATGGCTCTATTCCTTT
TTAATGGACTTTGGCGCCTTGGCCCACTTTCCTTCTTGACAACTGGTCCTGCTC
AGAGCATTCCTCTAGCTAACAAAGCGGTGTGTGGATGTGTGGGTGTAGGTGGC
GATACAGACCTCACTGGATGAACCGCCTTACTCTCCCTGCAGTTCGATCGGCA
GAGCTCCATTGCGCGGGGACCAGAATTCTACTTGCCCCCTACTCCACCCTTCT
GCACTGGTCAAGCCAGCGAGCCCGCTGCAGCCCACAGTAGTTGAGGCTGCTG
GGAGAAGGATGCGGACAGGGGTTGATGACTCGTGGTAGATCCCTCCGCGCGC
GGAGCTCGGGCAGCGCTTCTTCTGGGAAGCCCCTAGGTGCCCAAAGCGGCGG
GGCCGAGGCCGCGGCATTTCAATCAGGGCAGGGCTTTGCGGTGGGAGGGAGG
CGGCTGAGCGGAAGGACACACAGGGCTGCTTCGCTGCACACCGGTTCAAAGC
CAGAGCTTCAGGCGGCAGCTGAGGCGGCAGCCAGGGGCGTGACTCCAGGGC
GCAGGAGTCGGGCATTGCGCCTGTGCTTCGGACCGTAGCGCCAGGGCTAGGC
CTTTGAAGGCAGGAGGAGGAGAATTAGGGCTCCTATCGGGACCCCCCTCCCAT
GGATCTGCCCAGGCGGCGGCGGCGGCGGCGGCAGCGGCAGCAGCAGCAGCA
GCGGCGGCGGCACGGCAGCAACAGCAGCAACGGCTTGGCGAAGCGGCGGCG
GCAGCGGCGGCGGCGGCGGCGGCGGCGGCGGCGGCGACGGCAACAACGGCA
GCAGCAGCAGCGGCGGCGGCGGCGGCGGCGGCAGCAGCGGCGGCAGCAGCA
ACGGCTGGCAACGGCAACAGCAGCAGCAGCAGCGGCAGCGGCGGCGGCAGC
GGCGGCGGCAGCGGCAGCAGCGGCAGCGGCAGCGGCGGCAGCGGCGGCAGC
GGCAACGGCGGCAGCTGGCAGCGGCGGCAGCGGCGGCAGAGGAAGGCGGC

F9-II:6-read 2

GGCCTTTGTACTATGTTTAAGTTTGAATTTATCCTATGAGTCAGGGAGGTTCTAT
GGGTAGGATTCCCATCCCAACAGGATTGTAAGCTCCCACATCAATTAGTCACAC
CTCCATTTTTTTTCTGTTTCTTTAACATTGTCTCAAGGTTGTTCCTTCTAATCTAC
CTTATTAGTTCAGGTCTTCTTAAATTTGTGCCTGGACTGTTCACCAACCTCTTG

ACGGTCCTGGGAGCCAGTTTCACCTCTTGCTCCCAACAATCCAATTTTCTACA
GAACTATCAGAGCAATCTTCTTTCCATCAATCTCAACTCTGTTCAAAACTTTCA
ATAGTTTATATGACCCTAAGAGGTAGCTGCCTCCCCACCCCTTTCAGCTTGCCA
TTCAGGCTGCATGTAATTTGGTCCCAACCCCCTTTCCGTCCTTGACTCCTCTCA
CTGCTAACCAGAGACAAGAACTCATTGCCTCTTTTGACTGTACCTTTGCTGCTT
TCTGAGGCTCTTTCCTGGAATGATATGTCACGCTGTGTTGTTGTTCACTGGCTC
TTTTACTTAGCCCACTTGCCCAGGTTTGATCACACCCCTACTCTCTCCTGCCCT
GGAAGTAACTACTATTCTAGTTTCAGTCTCTTAATATCTATATCTATGTCTATATAT
ATGCATCTACAGATAATGTGTAGTATTGTCTTAGAAAAAAATATACATAAATGGT
ATACTGTCTCCTGCGATTAGTTTTTTTCACTGTATTTGTAGTTTTTAGATTTATCC
CTGACATAATGTGCAAATTACTTTTTCCATTACTGTTGCATTTCATTTTATAAATA
TACCATACATTATTCATCCATTCTACTCTAAGGGTGGGCATTTATGTTGTTTTCA
GGTTTTATTACAGTGTTATGAATATCCTTAGCTTAAGCTGGTCTCCTTGTCTTATA
CAAATTTTACTGACCTTTCAGATCCTGCTTCATCCAGCTCCTCTGGGGCTTCCC
TAAATCCCCAAACACAGCAGCAAAGTCTTGGTACTCAAAGTTGCTTGCATCTT
TGGTATCATCCACTGCAAGCACACAACTCACATAAAGTAAGGCACTTAAATATT
ATACGGATGAACAAATGAATGAATGAATGGGCAGCACCACTCCGTGATGACCA
TTGCTTTAATGGACTGGCGCCTTGGCCCACTTTCCCCTTGCGACTGGCAGCAC
TGGTCCTACTCCAGAGCAGCCCTCTAGCTAGCAAAGCAGTGTGTGGATGTGTG
GGTGTAGGTGGCGATACAGACCTCACTGGATGAGGACCCACTTACTCTCCCTG
CAAGTTCGATCAGCAGCAGAGAGCTCAGTGCTTGAAGTTCAGGTGCACCCCT
GCCTCCACGCCCCTTCTGCACTGGTCAAGCCAGCAAACCGCTGCAGCCCTGAT
CGAGAGTTAAGGCTGCTGGAGAAGGATGCGGACAGGGCCAGTGACTCAGTAG
TGAATCCCTCCGCGCCCAGACTCGGGCCGGCACTTCTTCCTGCAGGGAAACCC
TGGGTGCCCCAAGGCAGGCAGGGGCCAGGCCGCGGCGACAGTGGGGCGGGA
GCTTGCAGTGGGAGGGAGGCGGCTGAGGCCCGGAAGGACCACAGGCTGCTT
CGCTGCACACCCGAGAAAGTTTCAACCAAACTTCGGGCGGCTGGGCGGCGGC
CGAGGAGCGGCGCTTTCAGACTGGGGGCACGCAGGGGAGTCAAGGCATTGC
GCCTATGCCTTCGGACCGTAAGCGCCAGGCCTGAGCCTTTGAAGCAGGGAGG
AGGGAGGAGAGTGGGGCTCCTCTATCAGGACCCCTCCCATGTGGATCTGCCCA

GGCGGCGGCGGCGGCGGCGGCGGCGGCAGCAGCAGCGGCGGCGGCGGCGGC
AACGGCGCGGCAGCGGCGGCGGCGGCGGCGGCGGCGGCAGCAGCGGCGGCG
GCGGCGGCGGCGGCGGCGGCGGCGGCGGCGGCGGCGGCGGCGGCGGCGGCG
GCGGCAGCGGCAGCGGCGGCGGCGGCGGCGGCAGCAGCGGCGGCGGCGGCG
GCAACGGCAGCAGCGGCGGCGGCGGCCCGGCGGCGGCGGCAACGGCGGCGC
GGCAGCGGCGGCGGCGGCGGCGGCGGCAGCGGCAGCGGCGGCGGCAGCGGC
GGCAGCGGCGGCAGCGGCGGCGGCGGCGGCGGCGGCGGCGGCAACGGCAGC
GGCGGCGGCAGCGGCAACGGCGGCAGCAGAAGAGGCAGC

F9-II:6-read 3

GGATTCAGAGGATGAATTATGATTAAGTTCATTAAGAGGGAGGAGGAAGGGGA
CTACGAGTCATTCCAAGCCGAACAAAAGATACAGGGACAGAAACAGCAAGAT
ATGTTTGGGGGAATCTCAAGTTATGGCCAATACTATTGTGTGTGTGAAGTTCAC
AACAGAACATAGGAGGACAGGAGATACAGAGACCCAATGGCGAAAAGGCTG
TATGTACCATGTTTGATTTGAATTTTTATCTATGGAGTCGGGAGAGAGGTTCTAT
GGGTAGGATTCCCATCCAACAGGTTGTAAACTCCCACATCCAATTAGTCACAC
CTCCATTTTTTTTTCTGTTTTCTTTAACATTGTCTCAATTCATTCTTTAATCTACC
TTATGGATTTCAGGTCTTCTTTAAATTTGTTACCTGGACTGTTCTGCCTTTCCAG
CTAGTCACAAACCAGTTTCTTTCCCTCTTGCTCTAACAATCAATTTTTCTACAG
AACTATCAGAACAATCTTCCTTTCCATCAATCTCTCGCTCTGTTCAAAGCTTTC
AATAGTTTTATGTGACCCTAAAATTTAGCTGCCTCCCCACCTTTCAGCTTGCTG
CCATTCAAGCTGTAATTTAGTGCAACCTTTTACCATGTAGCTCTCTCTCTTAACA
GAGACAGAGACTCATTTACTTTTACTCACGGCTGTACCTTTGCTTTCAGGGCTC
TTTCTGGAATGATATGTCCATAACTATTGTTGTTTATTTCTTACTTTTAGCTCTTT
TCTTAGCCACTTGTACCCAGGTTTGATCCACCCCTACTCTCCTGCCTGGAAGTA
ACTACTATTCTGACATAGTCTTAATATCTATATCTATATCTATATATGCATCCACAG
ATAAATGTGTGATATTGTCTTGAAAAAATATACATAAATGGTATGCTTTGTCTCC
CTCTCGTTAGTTTTTCACTTTATCATTTGGTTTTAAGTTTATCTAACAATATAAAT
AATATAGTCTTTCAATTACTGTGTTACATTTCATTTTTATAAATACCATACATTATT
CATCCATTCCTCTAAGTGTGACATTTATGTTGTTTCAGGTTTGCTATTACAATTAT
TTGTGAATATAGCTTAAGCGGTCTCATGTCTTGCTCTAAATTTACTAGCCTTTCA

GGGTCCTGTTTCATCCAGCTCCTCTGGGAAAGCCTTCCTGAATCCCCCAGCCC
ACAGCAAAGTCTTGGTACTCAAAGTTTAGCATTGCATCTTTGGTATCATCCACC
AGACACAGCTACATAAGTAAGGGCATGAAATACCTTCGCTACACAGATGAACA
ATGAATAGATGAGTGAATAGCACCACTCCATGGTGGCTCTATTCCTTTTTACCT
GTGTGGACTTTGGCACCTTATTTTTCCTTCTTGTGTACTGAATTCTCAGAGCATT
CTCTAGCTATAGAAGCAGATGTGTGGATGTGTGGGGTGCAGGGGTGGCAGTGA
ACCTCACTGGATGAGGACCGCGCCTCTCCCTACACAAGTTCAGTCAGCAAACT
CCAGTGCTTGATTTGAAGTCCCTGTACCCCCTCACTGCGCCCCTTCTGCACTGG
TCAAGCCAGCAGGCCGCTGCAGCCCTGATCGAGTTAAGGCTGCTGGAGGAAG
GATGCCAGACAGGGCCAGTGACTCGTAGTGAATCCCTCCGCGCAGGCCAGGC
CAGCGCTTCTTCCTGGGGAAACTGAAATTTTGGGCGGCGGGGCCAAAAGGCC
CTTGGCGACAATGAACGAACACACGCACGTTGGGAGGAGCGGCTGGGAGCG
GAAGAACACACAGACTGCTTCGCGCTGCACCCGAAAGTTCAATAATGAAACT
GGGCGGCGGCTGGGGGCGGCGGCGAGGCAGCAGGCTCCGGGGCGCGGGGAG
TCGAGGGCATTTGCGCCTGTGCTTCGGACCGTGGCGCGCCAGGGCTGAGCCTT
TATGAAGCAGGAGGAGGGAGGAGAGTCGGGCTCCTCTATCGGGACCCCTCCC
GTATGGATCTGCCGGGCGGCGGCGGCGGCGCGGCGGCGGCGGCGGCGGCAGC
GGCGGCGGCGGCGGCGGCGGCAACAACGGCAGCAGCAGCGGCGGCGGCGGC
GCGGCGGCAGCGGCAGCGGCAGCGGCAGCAGCGGCAGCGGCAGCGGCGGCA
ACGGCAATGGCACAGCAGCAGCAGCAGCGGCAGCGGCGGCGGCGGCAGCGG
CGGCGGCGGCGCGGCGGCAGCGGCGGCGGCGGCGGCGGCGGCAGCGGCAGC
GGCGGCGGCGAAGCGGCGAAGCGGCAGCAGCGGCAGCGGCAGCGGCAGCG
GCAGCAGCGGCGGCGGCAGCAGCGGCGGCGCGTGGCGGCTGGCGGCAGCGG
CGGCAGCGGCGGCGGCAGCGGCGGCAGCGGCGGCGGCGGCGGCGGCGGAGG
AAGGCGGC

<u>F9-II:6-read 4</u>

GGGAGGAAGGGACTGAGGTCATTCCAAGCAAAGGAACAAGATACAGGGACA
GAAACAAGATATGTTTGGGGAATCTCAAGTTGGCAATACAACTGACTTGTGTG
AAGTTCACAACAAGGCAGGGAGGATGGGAGCCCAATGAAGGCTTATGTGTAC
CATGTTTGAGAAGTTTGAATTTATCCTGAGTCAGGAAGGGATTCTATGGGTGG

GGTTTTCATTAACAGGATTGTAAACTCCCACATCCAATTAAATTACACCTCCATT
TTTCTGTTTCTTTAAGCAACCTTCTCAGATTCATTCCTTCAAATCTATGTTGATA
GTTCAAGTCTTCTTAAATTTGTACAGATGTTCTGGCTCCCAGCTAGTCCTGGGC
TCAGTTGCCCTCTTGCTCCCAACAGTGAATTTTCCTACAGACTTCTCAGAGCA
ATCTTCTTTTATCAAATATCAACTCTGTTCAAAAACTTTTAATTAGTTTATTCTTG
TTGTTCAAGGGTAGCTGCCTCACCCTTTGTGCCATTCAGGCTGTAATTTGGTCC
CCAACCCCTTTCAAGTCCTTGGCTCCTACCATACTAACAGAGACAGAACTCAT
TGCCTCTTTTGACTGTACCTTTGCTTTCACAGGGCTCTTTCCTGAGATAATTATC
CATAAGCTATTGTTGTTGTTCTTACTTTAGCTCTTTTCTTGCACTTGCAGTTTGA
TCACCCCCTACTCTCACACCCCTGGGAAGTAACTTTTCTTCTAATTTATAGTCTC
TACCATGTCTATCCTCATATATATATGCATCCCACAGATAATGTGTAGTATTGTCT
TGAAAAAATATACATAAATGGTATACTTCCTTCTCCCTCTACGATTAGTTTTTCC
TATCATTTAGTTTTAGATTTATCCTATTGACATAAATGCTTTCTAGTCTTTTCAAT
TTTATTGCATTCATTTTATAAATACTACCATTATTCATCCATTCCTTAAGGGTGAG
CATTTATGTTATTTTCAGGGTTTACTATTACAGTGTTGATGAATATCCCTTAAGCA
GCTGTCTCTAACCTCATATACAAATTTTACCCCAGCCTTTCAAGATCCTGCTTTC
TGTGCCCAGCTCCTCTGAGAAGCCTTTACTGGTCCCAGCCCACAGCAGCAAGT
CTTGGTACTCTACAGTTGTGTCTTTGGTGCTCGTCACTGTAAACACCACAGCTA
TAAAGTAAGGCATGAAATATTCATGGATGAACAATGAATGAATAGACCAGCTTC
CTTCCGTAATTAGCTCTATTCCTTTTTAATGGACTTTGGCCACTTTTCCTTCTTG
ACAGCATAATTACCTCCAGGCATTCCTCTAGCTATGCCGTGATGCCGGATGTGT
GGGTGGGTGGCGATACAGACCTCACTGGATGAGGGACCGCCTCTCCCCTGCA
GTTCACGATCCCGGCGAAGCTCAGGTGCTTGAAGTTCAGGTCCCTACCCCCCC
CTGCCTCCACGCCCTTCTGCATAGTGAGCCAGCGAGCCGCTGCAGCCCTGATC
GAGTTAAGGCTGCTGGAAGGATGCGGACAGGGCAATGATGACTCGTGGTAAG
ATCCCTCGCGCGGGCCTCGGGCCAGCGCTTCTTCTGCGGGAAACCCCTGGGTG
CCCAGGCGCAGCAGGGCCGGGCCGCGGCGACAGTAAAGACAGAGGCCCTGC
GGTGGGAGGGAGGCGGCTGGGCGAGAGGACACACGAGGGCTGCGCTGCACC
AGAAAGTTTCGTATTAAACTTCAGGCGGCGGCTGAGGCGGCAGCCGAGGCCA
CAACAGACTCAGGAGCGCAGGAGTCAGGGCATTGCGCCTGTGGCTGGACCAT

GGCGCAGGGCCTGAGCCGCTGGAAGCAGGAGGAGGGAGAAGTGGGCTCCTG
GGACCCCCCTCCCCATGTGGTCTGCCCAGGCGACGCTGGCGGCGGCAGCAGC
GGCGGCAGCGGCGGCGGCAGCAGCAACGGCAACGCGGCAGCGGCGGCGGCG
GCGGCAGCGGCAGCAGCGGCCAGCAGCAGCAGCAGCAGCAACAGCAGCAAC
AGCAACAACAACAGCAACGGCAGCAGCAGCAGCAGCAGCAGCAGCGGCAGC
AGCAGCAGCAGCAGCAGCAGCGGCAGCAGCCCAGCAGCGGCAGCAACAACA
ACAGCAACAACAGCGCGGCAACAGCAGCAGCGGCAGCAGCAGCAGCAGCAA
GCAGCAACAGCAGCGCTGGCGGCAGCAGCAGCAACAACAGCAGCAGCAGCA
GCAACAGCAGCAACAGCAACAGCAACAACGGCAACAGCAGCAGCAGCAGCA
GCAGCAGCAGCAGCAGCAGCAGCAGCAGCAGCGGCAGCAGCAGCAGCAACA
GCAACAGCAGCAACAGCAACGGCGGC

F9-II:6-read 5

GGAGACGGTGGAGGGACTGTCTGTTGGGGAATTTATTGTGTGTCGGCAAAAG
CCTTCTTTGGACTGAAGGATGGTGCCAGTTAGAAGTTCATTAGAGGAGGAGGA
AGGGACTGAGTCATTCCAAGCCAAAGACAAAAGATACGGGACAGAAACAGC
AAGATATGTTGGGGAATCTGGGATTAGCTAATACTGTTATTGTGCTGAAGTTTC
ACAACAGGGGCAGGGAGGACAGGAGATGGGGCCCCAATGTGAAAAAGGCCT
TGTATGTACCATGTTTAGAAGTTTGAATTTTATCCTATAGGGAGTCAGGAGAGG
TTCTATGGGTGGGGTTCCCATCCCAACAGGATTGTAAACTCCCACATCAATTAG
TCACACCTCCATTTTTTTCTGTTTCTTTTAACATTGTCTCAATTCATTCCTTCTA
GAAATCTACCTTATTAGTTCAGGTCTTCTTAAATTTGTGCTGGACTGTTCACAA
CTCTTGACTGGTCCTGGGGCTCCAGTTCACCTCTTGCTCCAACAATCAATTTTC
TAGAACTATCAGAGCAATCTTCCTTTCCATCAATCTCAACTCATTTCAAAACTT
TCCAATAGTTTTATGTGACACAAGGGTAGCTGCCTCCCCACCCTTTCAGCTTGC
CATTCAAGGCTGCATAATTTGGTCCCAACCCCCTTTCAGGTCGCGGCTCCTCAC
TGCTAACAGAGACAGAACTCATTTACTTACTCTTGGCTGTACCTTTGCTTTCAC
GAGAATGCTTTCCTGGAATGATATCCACTACAATATTGTTGTTGTTCTTACTTTT
TAGCTCTTTTCTTAGCCCACTTGTACCCAGGTTTGATCCACACCCCCTACTCAC
TGCCCTGGAAGTAGCTGCTATTCTGATTTATAGTCTCTTAATATCTATATCTATAT
CTATATATATACCGTCACAGATAATGTGTGGTATTGTCGAAAAAATATACATAAA

TGGTATGCTTTGTCTCCCTCTACGATTAGTTTTTCACTGTCATTTAGTTTTTAGAT
TTATCCCTATTGACATAAATAGCTATAGTCTTTTCAATTACTGTGTTGCATTTCAT
TTTAACAAATATACCATACATTATTCATCCATTCCTCTAAGATTTGGGCATTTATG
TTGTTTTCAGGTTTTGCTATTACAGTGTTACCTTGAATATCCTTGGCTTAGCTGG
TCTCTTCTTGTCTTATACAAATTTTTACTGACCTTTCAAGATCCTGCTTTCATCC
AGCTCCTCTGGGAAAGCCTTCCTGAATCCCCCCAGCCCACAGGCGGCAAAGT
CTTGGTACTCAAGTTTAGCAGCCATCTTTAAGGTATCATCCCACTGCCAAACAC
ACAACTCATAAGCCAGGCACTTAAGAAATATTCCTTTGCGGATGAACAAATGA
ATGAATGAATGGGGCGCAGCACCACTCCGTGATGGCTCTGTTCTTTTTAATGGA
CTTTGGCGCCATGGCCCACTTTCCTTCTTGACAGCACTGGTCCCTGCTTAGAGC
ATTCCTCTAGCTAACAAAGCAGTGTGTGGATGTGTGGGTGTGGGTGGCGATAC
AGACCTCACTGGATGAGGACCGCCTCTCCCTGCAAGTTCACAGTCGGCAAAC
TCCAGTGCTTGAAGTTCAGTCCTACCCACCCCCCTCACCTCACGCCCCACTGC
ACTGGTCAAGCCAGCAGGCCGCTACAGCCTGATCAGGTTAAGGCTGCTGGAG
AGAAGGATGCGGACGGGAGCCAGTGACTCGTAGTGAATCCCCTCCGCGCGGA
GCTGGCCGGCGCTTCTTCCTGCGGGAAACCCCTGGGTGCCCAAGGCGGGGCC
AGGCGGCGCGACAGTGGGGCAGGGCTTGCGGTGGGAGGGAGGCGGCTGAGG
CGGAAGGACACACGAGGGCTGCTTCGCTGCACGCGAAAGTTTCAGCCAAACT
TCGGGCGGCAGCTGGAGCGGCAGCCGAGGCAACAGACTCAGGGCGCAGGGA
GTCGAGCATTTTACTTTTATGCTTCGGACCGTAGCGCCAGGGCCTGAGCGCAC
TGAAGCAGGAGGAGGAGGTGGGGCTCCTCTATCCGGACCCCTCCCATGTGGAT
CTGCCCAGGCGGCGGCGGCGGCGGCAGCAGCGGCGGCAGCGGCGGCGGCAG
CGGCGGCGGCGGCGGCGGCGGCGGCAGCAACGGCAACGGCAGCAGCAGCAG
CGGCGGCGGCGGCGCGGCGGCGGCGGCGGCGGCGGCGGCGCGGCGGCGGCG
GCGGCGGCGGCAGCGGCGGCGGCAGCAGCTGACTACGGCAACGGCAGCGGC
GGCGGCAGCGGCGGCGGCGGCGGCGGCGCTGGCAGCAGCAGCGGCGGCAAC
GGCGGCGGCAGCAGCAGCAGCAGCGGCGGCGGCGGCGGCGGCGGCGGCGGC
AGCAGCGGCAACGGCGGCGGCGGCGGCAGCAGCAGCCGGCGCGGCGGAGGA
GGCGGC

<u>F9-II:6-read 6</u>

GGGGTCATTTCGAAGCCAAAGGAACAAAAGATACAGGGACAGAAACAGCAA
GATATGTTTGAGGTCTCAAGTTGGCCATCACTGTTGTTGGCCTTGAGAAGTTCT
ACAACAGGGGCAGGGAGGACAGAGATGGGGCCCAATGACGAAAAAGAGCCT
TGTATGTACCATGTTTAAAATTTTATCCGAGTCAGGGAGTTTATGGTGGGTTTAT
CCCAACAGAGTTATATTATGACTCCCATCCATTAGTCACACCTCCATTTTTTTC
TGTTTCTTTTAACATTGTCTCAGATTCATTCCTTCAGGAAATCTACCTTATTCGT
TTCAGGAGTCTTCTAGCAAGTTGTGCCTGGACTGTTCACCCAGCCTCTTGTTG
ACTGGTCCTGGGGCTCCAGTTCACCTCTTGCTCCCAACAATCAATTTTTCTACA
GAACTATCAGAGCAATCTTCCTTTCCATCAATCTCAACTCTGTTTAAAAAACTT
TCAATATTTTATCATGACCTAAGTAGCTGCCTCCCCACCCTTTCAGCTTGCCATT
CAGAGGCTGCATAATTTGGTCCAGCACCGTCCTTGACTCCTCTCACTGCTAAC
AGAGACAGAACTCATTTACCTGCTGACTGCCTTTACTTTCCTGGGCTGCTGGA
ATGATAGCATCCATACAACTATTGTTGTTGTTCTTACTTTTAGCTCTTTTCTTAGC
CCACTTGTACCCCGGTTTGATCACCCCTACTCTCTGCCTGGGAAGTAACTACTA
TTCTGTTATAGTCTTAATATCTATATATATATATATCTATATATATGCATCCACAGAT
AATGTGTAGTATTATCTTGAAAAATATACATAAATGGTATACTTTGTCTCCCTCTC
GATTAGTTTTTCACTATCATTTAGTTTTTAAGATTTATCTATTGACATAAATAGCT
ATGGTCTTTTCAATTACTGTGTTGCATTTCATTTATAAATATACCATACATTGTTA
CTAAATCCATTCCTCTAAGGGTGAGCATTTATGTTTTCAAGGTTTTTATTACACA
GTGTTACTTGAATATCATATATGCTCACTTGTCTTATACAAATTTGCTGACCTTTC
AAGATCCTCACTTTCATCCCAGCTCCTCTGGGAAAGCCTTCCCTGAATCCCAG
CCCACAGCGGCAAAGTCTTGGCCTCAAGTTGCTTGTGCCATCTTGGTATCATCC
CACTGCACCAAGCACACAACTACATAAAGTAAGGCACTTAAAATATTAAGCTA
CACGGATGAACAAATGAATGATGAATGAATAGGGCAGCACCACTTGTGATGGC
TATTCCTTTTTGAACTTTGGCGCCTTAGCCCCACTTTCTGCTTCTTTGACAGCA
CTGAATCCTACTCCAGAGCATTCCTCCTTCTTGTAAAACAGTGTGTGAGTGTG
GCGGGTGTGGTGGCGATACAGACCTCACTGGATGAGGACCGCCTCTCTCCCTG
CAAGTTCGGTGACAAACTCCAGTGCTTGAAGTTCAGAGTCCTACCCACCCCCT
CACCTCCACGCCCCTTCACCAGGTCAAGCCAGCGAGCCGCCTGCAGCCCTGA
TCCCAGGTTAGTACTGGAGAAGGATGCGGACAGCCTCCTGCAGGAAGCCTGG

GTGCCAAGGCAGCGAACCCGGGCCGCGAGCAGTGGGGCAGGGCTTGCAGTG
GGAGGAGGCGGCTGAGGCAGGAAGGACACGGCTGCTTCGCTGCACACCCGA
AAGTTCAATGCAGCTTCGGGCGGCGGCGCTGGGCGCGGCCGAGGCAGCAGAC
TCAGGGCGCAGAGAGTCAAGGCGTTGCGCCTGTACTTCGGACCGTAGCGCGG
TTCAGGCGCTGGCAGGAGGGAGGGAGAGAGTGAATCCTCTATCAGGACCCCC
CCTCCCCATGTGGATCTGCCAGGCGGCGGCGGCACGGCGGCGGCAGCGGCGG
CAGCGGCGGCGGCGGCAGCAGCAGCAGCGGCGGCGGCGGCGGCGGCGGCGG
CGGCGGCGGCGGCGGCAGCTGGCGGCAACGGCGCGGCGCAGCAACGGCGGC
AGCAGCGGCGGCGGCGGCGGCGGCGGCGGCAACGGCAGCGGCGGCAGCGGC
GGCAGCAGCGGCGGCACGGCAACGGCGGCAACGGCGGCGGCGGCGGCGGCG
CGGCGCGGCGCAACGGCGGCAACGGCAGCGGCAGCAGCGGCAGCGGCGGCG
GCGACGCGGCGAAGCGGCGGCGGCGGCAGCAGCTGGCGGCAGCGGCGGCGC
GGCGGCGGCGGCGGCGGCGGCGGCGGCGGTAACGGCAGAGGAGCCGGC

<u>F9-II:6-read 7</u>

GCCAAAGGAACAAGATACAGGGACAGAAAACAGATATGTTTGGGGGAATTCC
CTGGTTAGCCAATACTGTTATTGTGAAGTTCACAACAGGGGCAGGGAGGATAA
GAGATAGCTCAATGACTAAAAAGGCCTTGTATGTACCATGTTTAGAAGTTTGAA
TTTATCCTATAGGAGTCAGGGAAGAGGTTCTATGGGTAGGATTTCATCCCAACA
GGATTGTAAACCCCCACATCCAATTAGTCACACCTCCATTTTTTTTTTCTGTTT
CTTAACATTGTCTCAGATTCATTCCTTCGAAATCTACCTTTATTAGTTCAGGTCT
TAAATTTGTGCCTGACTGTTCACCAACTCTGGATTGGTCCTGGGGCTCCAGTTC
ACCCTTGCTCCCCAACAATCAATTTTCTACAGAACTATCAGAGCAATCTTCCTT
CCATCAACAATCTCAACTCTGTCTGCAAAAAATCTCAATAGTTTTATGTGACCT
AAAGAGGTATTGCCTCTAATCTTTCAGCTTGCCATCTGTTGCATACAATTTGTCC
CAACTCTTCCGGCCCTTGACTCCTCTCACTGCTAACAGAGACAGAACTCATTT
ACTTACTCTTTGACTGTACCTTGCTTTCACGAGGGCTCTTCCTGGAATGATATG
TCCATACAACTATTGTTGTTGTTCTTACTTTTTAGCTCTTTTCTTAGCCCACTTGT
ACCCAGGTTTGATCACACCCCTACCTCCTGCCCTGGAAGTAACTACTATTCTGG
TTATAGTCTCTAATATCTATATCTATATCTATATATATATCCACAGATAATGTGTAGT
ATTGTTCTTGAAAAATATACATAAATGGTATACTTGTCTCTGCGATTAGTTTTCA

CTATCATTAGTTTTTAAGATTGTCCTATTGACATAAATAGTTATAGTCTTTTTCAA
TTATTGTGCCAGACATTTCATATTTATAAATATACCATACATTATTCATCCATTCCT
CTAAGGGTGGGCATTTGTGTTGCTTTCAAGGTTTGCTATTACAGTGTTACAACT
AATATCCTTAGCTTAGCTGGTCTCCTTGTCTTATACAAATTTTATTGACCTTTCA
AGATCCTGCTTTATCCCAGCTCCTGGGAAAGCTCTTCCTGAATCCCCCAGCCCA
CAGCGGTAAAGTCTGGTACTCAAAGTTGCTTTGCATCTTTTGGTATCATCCCAC
TGCCAAGCACACAGCTACGCAAAGTAAGGCACTTAAGAAATATTCGCTACACG
ATGAACAAATGAATGAATGAATGGGGCAAAAAGCACCACCTGTGATGCCTTAT
CTCCTTTTAATGGACTTGGCGTGCCTTGGCCCACTTTTCCTTCTTTTGAATAGC
ACTGGTCCTACTCAGGAGCATTCCTCTAGCTAGCAAAGCAGTGTGTGATGTGG
GTGTAGGTGGTGACACAGATCTCACTGGATGAGACCGCCTCCCTGCAAGTTCA
CGATCCTGGCAACCCAGTGCTTGAAGTTCAGGGAGTCCTACCCCACCCCTCAC
CTCCACGCCCTTCTGCACTGGTCAAGCCAGCGGGAGCTGCAGCCCTGATCGA
GTTAAGGCTGCTGGAGAAGCATGCGGACGGGCCAGTGGACTGTAGTAGATCC
CTCCGCGCGGGAGCCTGGGCCGGCGCTTCTTCCTGCGGGAAACCCCTGGTGC
CTGCAAGGCGGTGGGGGAAGGGGAGGCCGCGGCGACAGTGGGGCGGGGCTT
GCGGTGGGAGTGAGGCGGCTGAGGAAGGACACACGAGGTTGCTTGCTGCAC
ACCTGAGAAAGTTTCAGCTAAACTTCGGGCGGCATGAGGCGGCGGCCGAGGA
GCGGCGGACTCTGGGCGCGTGGGGAGTCGAGGCATTTGCGCCTGTGCTGACC
AGCGCTAGGGCCTGTTTTGAAGCAGGAGGAGGGAGGAGAGAGTGGGGTTTTA
TCGGGACTCCCCTGGCGATTTACCAGGCGGTAAGGGTGGTGGTGGCGGTGGT
GGCGGTGGCGGTGGTGGTGGTGGCGGTGGTGGCGGTGGTGGTGGCGGTGGCG
GTGGTGGCGGTGGCGGGTGGGTGGTGGCGGCGGGTGGCGGTGGTGGTGGTG
GTGGTGGCGGTGGTGGTGGCGGCGGGTGGTGGCGGCGGTGGCGGTGGTGGTG
GTGGCGGCGGCGGCGGTGGTGGCGGGTGGCGGCGGCGGCGGCGGCGGCGGG
TGGTGCGGCGGCGGCGGCGGTGGCGGCGGTGGCGGCGGCGGCGGCGTGGCG
GTGGCGGCGGCGGTGGCGGCGGCGGCGGCGGCGGCGGCGGCGGCGGCGGAG
GAGGCGGC

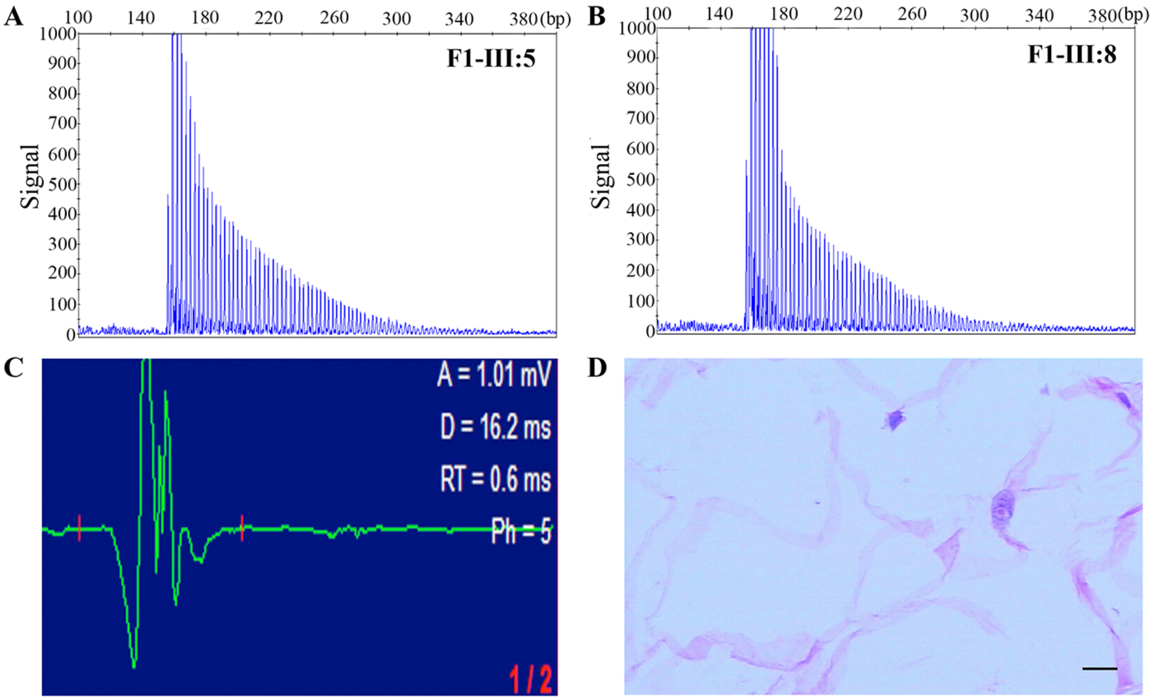

Figure S2. Additional Molecular and Clinical Diagnosis of Family 1. (A-B) RP-PCR assay and GC-PCR assay showed abnormal repeat expansion in subject F1-III:5 **(A)** and F1-III:8 **(B). (C)** Representative motor unit potential (MUP) of right tibialis anterior from electromyogram of subject F1-III:8 indicated neurogenic impairment. **(D)** Skin biopsy sample of subject F1-III:8 displayed typical eosinophilic intranuclear inclusions. Scale bars, 10μm.

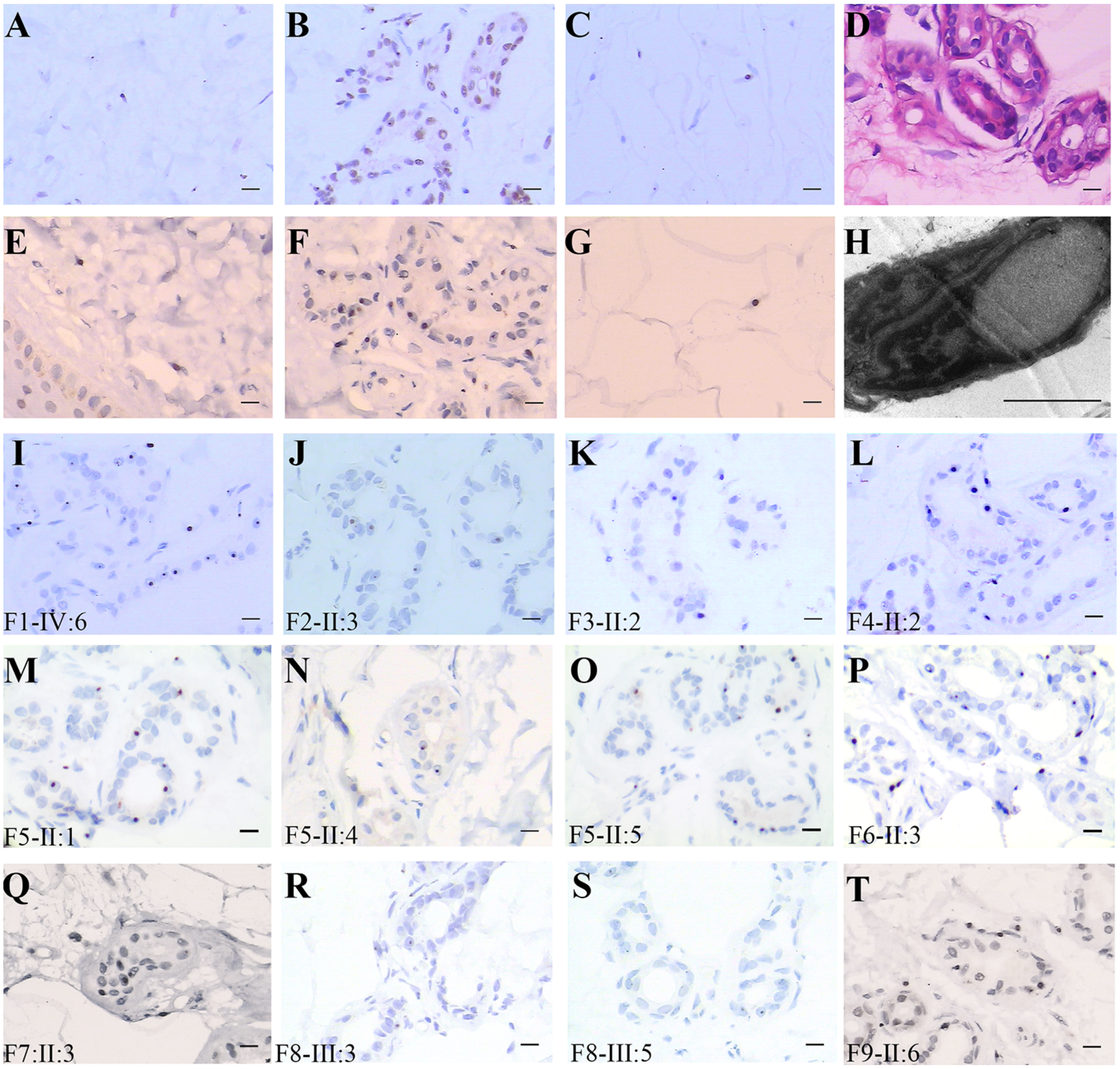

Figure S3. Histopathological Features of NIID. (A-C) Representative skin biopsy samples showing ubiquitin-positive intranuclear inclusions in fibroblasts **(A)**, sweat gland cells **(B)**, and adipocytes **(C)**. Scale bars, 10μm. **(D)** Representative skin biopsy samples revealed eosinophilic intranuclear inclusions in sweat gland cells. Scale bars, 10μm. **(E-G)** Representative skin biopsy samples indicated p62-positive intranuclear inclusions in fibroblasts **(E)**, sweat gland cells **(F)**, and adipocytes **(G)**. Scale bars, 10μm. **(H)** Electron microscopy imaging displaying intranuclear inclusions without membrane. Scale bars, 2μm. **(I-T)** All skin samples showed p62-positive intranuclear inclusions. Scale bars, 10μm.

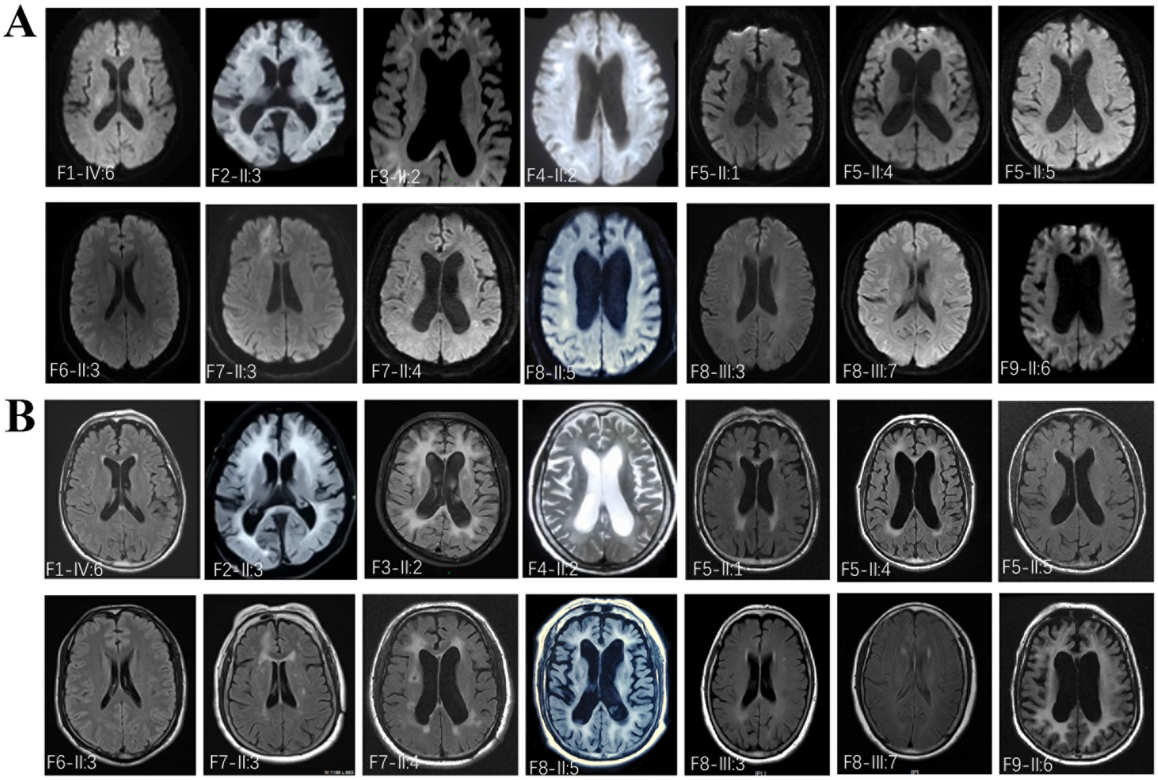

Figure S4. MRI Imaging of NIID. (A) DWI images of some NIID-affected case subjects revealed typical symmetrical high signal in corticomedullary junction. **(B)** Several NIID-affected case subjects showed severe white matter hyperintensity using FLAIR or T2 weighted image.

Table S1. Clinical Features of Muscle Weakness-dominant Type (families 1 and 2).

	Family1												Family2		
	III:5	III:8	III:11	IV:1	IV:4	IV:5	IV:6	IV:7	IV:10	IV:12	IV:15	V:1	II:1	II:2	II:3
Sex	M	M	M	M	M	M	M	M	M	M	F	M	F	F	M
Age at onset	30	/	30	54	31	40	33	32	30	47	34	31	38	33	35
Disease duration	49	/	38	6	18	8	14	9	13	3	14	3	20	20	18
Clinical manifestations															
Dementia	-	-	-	-	-	-	-	-	-	-	-	-	-	-	-
Abnormal behavior	-	-	+	-	-	-	-	-	-	-	-	-	-	++	-
Peripheral neuropathy															
Muscle weakness	-	-	++	+	++	++	++	++	++	+	++	+	+++	++	+++
Sensory disturbance		-	-	-	-	+	+	-	-	-	+	-	/	/	+
Autonomic dysfunction															
Bladder dysfunction	-	+	-	-	+	-	+	-	+++	-	-	-	-	-	++
Miosis	-	-	/	-	/	-	+	-	-	-	-	-	/	/	-
Parkinsonism															
Tremor	+	-	+	+	+	+	++	+	+	+	+	-	-	-	+
Rigidity	-	-	-	-	-	-	-	-	-	-	-	-	-	-	+
Bradykinesia	-	-	-	-	-	-	-	-	-	-	-	-	-	-	+
Ataxia	-	-	-	-	-	-	+	-	-	-	-	-	-	-	+
Neurological attack															
Disturbance of consciousness	+	-	-	-	-	-	-	-	-	-	-	+	-	-	+
Stroke-like episode	-	-	-	-	-	-	-	-	-	-	-	-	-	-	+
encephalitic episode	-	-	-	-	-	-	-	-	-	-	-	-	-	-	+
Brain MRI															
Severe leukoencephalopathy	/	-	/	/	-	-	-	-	/	/	-	/	/	/	+++
DWI U-fiber high signal	/	-	/	/	/	/	-	/	/	/	/	/	/	/	+
Cognitive function test															
MMSE (<education matched average)	/	/	/	/	28	28	29	28	28	/	29	/	/	/	28
MoCA (<education matched average)	/	/	/	/	26	18	21	26	25	/	23	/	/	/	26
Nerve conduction															
Motor															
MCV slowing	/	-	/	/	++	++	++	++	+	/	+	+	/	/	+++
CMAP reduction	/	-	/	/	+	+	+	+	+	/	+	-	/	/	++
Sensory															
SCV slowing	/	-	/	/	++	++	++	++	-	/	+	+	/	/	+++
SNAP reduction	/	-	/	/	+	+	+	+	-	/	+	-	/	/	+
Skin biopsy	/	+	/	/	/	/	+	/	/	/	/	/	/	/	+

Table S2. Clinical Features of Parkinsonism-dominant Type (families 5-7)

	Family 5			Family 6			Family 7		
	II:1	II:4	II:5	I:1	II:2	II:3	I:1	II:3	II:4
Sex	M	M	M	M	M	M	M	F	M
Age at onset	75	58	66	78	37	46	58	66	61
Disease duration	2	11	2	1	15	3	10	6	4
Clinical manifestations									
Dementia	-	-	-	-	-	-	-	-	-
Abnormal behavior	-	-	-	-	-	-	-	-	-
Peripheral neuropathy									
Muscle weakness	-	+	-	-	-	-	-	-	-
Sensory disturbance	+	-	-	+	-	-	-	+	+
Autonomic dysfunction									
Bladder dysfunction	-	++	-	-	-	+	+++	++	+++
Miosis	-	-	-	-	-	-	/	-	-
Parkinsonism									
Tremor	+	++	-	-	+	-	-	+	+
Rigidity	+	++	+	+	+	+	+	++	+
Bradykinesia	+	++	+	+	+	++	+	++	+
Ataxia	-	+	-	-	-	-	++	+	++
Neurological attack							-		
Disturbance of consciousness	-	+	-	-	-	-	-	-	-
Stroke-like episode	-	+	+	-	-	-	-	-	-
encephalitic episode	-	-	-	-	-	-	-	-	-
Brain MRI									
Severe leukoencephalopathy	-	+	-	/	/	-	/	-	+
DWI U-fiber high signal	-	+	-	/	/	-	/	-	-
Cognitive function test									
MMSE (<education matched average)	26	27	26	/	29	30	/	22	25
MoCA (<education matched average)	19	15	19	/	/	25	/	/	23
Nerve conduction									
Motor									
MCV slowing	+	+	+	/	/	+	/	++	+
CMAP reduction	+	-	-	/	/	-	/	-	+
Sensory									
SCV slowing	+	-	-	/	/	-	/	++	+
SNAP reduction	-	-	-	/	/	-	/	+	+
Skin biopsy	+	+	+	/	/	+	/	+	/

Table S3. Clinical Features of Dementia-dominant Type (families 3, 4, 8, 9)

	Family 3			Family 4		Family 8							Family 9			
	I:2	II:2	II:3	II:2	II:5	I:1	II:3	II:5	II:8	III:3	III:5	III:7	I:2	II:2	II:3	II:6
Sex	F	M	F	F	F	M	F	M	M	F	F	F	F	M	F	M
Age at onset	71	42	63	49	31	65	60	60	60	60	58	59	60	60	60	71
Disease duration	20	19	2	18	30	5	8	19	16	7	5	3	15	10	12	10
Clinical manifestations																
Dementia	++	-	+	+	++	+++	+++	+++	+++	++	+	-	++	++	++	+++
Abnormal behavior	++	++	+	++	+++	++	++	++	++	-	-	-	++	++	++	+
Peripheral neuropathy																
Muscle weakness	-	-	+	-	-	/	-	+	-	-	-	+	-	-	-	+
Sensory disturbance	+	-	+	-	-	/	-	+	-	-	+	+	-	-	-	-
Autonomic dysfunction																
Bladder dysfunction	+	-	++	++	-	+	+++	+++	+++	-	++	+++	/	+++	+++	+++
Miosis	-	-	-	+	+	/	/	+	/	-	-	-	/	/	/	+
Parkinsonism																
Tremor	-	-	-	+	-	-	-	+	-	-	-	-	-	-	-	+
Rigidity	-	-	-	++	-	-	-	-	-	-	-	-	-	-	-	+
Bradykinesia	-	-	-	+	-	-	-	-	-	-	-	-	-	-	-	+
Ataxia	+	-	-	-	-	-	-	-	-	-	-	-	-	-	-	-
Neurological attack																
Disturbance of consciousness	-	-	-	+	-	-	-	-	-	-	+	-	-	-	-	+
Stroke-like episode	-	-	-	+	-	-	-	-	-	-	-	-	-	-	-	-
encephalitic episode	-	-	-	-	-	-	-	-	-	-	-	-	-	-	-	+
Brain MRI																
Severe leukoencephalopathy	/	/	+	+	/	/	/	+	/	-	-	-	/	/	/	+
DWI U-fiber high signal	/	/	+	+	/	/	/	+	/	-	-	-	/	/	/	+
Cognitive function test							/									
MMSE (<education matched average)	14	29	/	15	12	/	/	/	/	10	26	29	/	/	/	/
MoCA (<education matched average)	/	28	/	/	/	/	/	/	/	3	20	26	/	/	/	/
Nerve conduction																
Motor																
MCV slowing	/	+	/	-	/	/	/	+	/	-	+	+	/	/	/	++
CMAP reduction	/	+	/	-	/	/	/	+	/	-	+	+	/	/		++
Sensory																
SCV slowing	/	+	/	-	/	/	/	+	/	-	+	+	/	/	/	+
SNAP reduction	/	+	/	-	/	/	/	+	/	-	+	+	/	/	/	+
Skin biopsy	/	+	/	+	/	/	/	/	/	+	+	+	/	/	/	+

Table S4. Clinical Features of Sporadic NIID-affected Case Subjects

	Sporadic Case Subjects				
	Dementia		Paroxysmal disease		
	SD1	SD2	SP1	SP2	SP3
Sex	F	F	M	M	F
Age at onset	65	69	68	51	57
Disease duration	14	2	1	5	6
Clinical manifestations					
Dementia	++	++	-	-	-
Abnormal behavior	++	+	-	-	-
Peripheral neuropathy					
Muscle weakness	-	-	-	-	-
Sensory disturbance	-	-	-	-	-
Autonomic dysfunction					
Bladder dysfunction	+++	+++	-	++	-
Miosis	+	+	-	-	-
Parkinsonism					
Tremor	+	-	-	-	-
Rigidity	+	-	-	-	-
Bradykinesia	+	-	-	-	-
Ataxia	-	-	-	-	-
Neurological attack					
Disturbance of consciousness	+	+	+	+	-
Stroke-like episode	+	+	+	-	+
encephalitic episode	+	+	-	-	+
Brain MRI					
Severe leukoencephalopathy	+	+	+	+	+
DWI U-fiber high signal	+	+	+	+	+
Cognitive function test					
MMSE (<education matched average)	/	/	27	30	/
MoCA (<education matched average)	/	/	16	29	/
Nerve conduction					
Motor					
MCV slowing	+	/	+	+	/
CMAP reduction	+	/	-	-	/
Sensory					
SCV slowing	+	/	+	+	/
SNAP reduction	+	/	-	-	/
Skin biopsy	+	+	+	+	+
