# Supplementary material for: Expansion of Human-Specific GGC Repeat in Neuronal Intranuclear Inclusion Disease-Related Disorders
Source: Am J Hum Genet. 2019 Jun 6;105(1):166–76. doi: 10.1016/j.ajhg.2019.05.013 (PMC6612530; doi:10.1016/j.ajhg.2019.05.013)
Supplement: Document S2. Article plus Supplemental Data [file mmc2.pdf]

ARTICLE

Expansion of Human-Specific GGC Repeat in Neuronal Intranuclear Inclusion Disease-Related Disorders

Yun Tian,[1,3,14] Jun-Ling Wang,[2,14] Wen Huang,[4] Sheng Zeng,[2] Bin Jiao,[2] Zhen Liu,[2] Zhao Chen,[2] Yujing Li,[5] Ying Wang,[6] Hao-Xuan Min,[7] Xue-Jing Wang,[8] Yong You,[9] Ru-Xu Zhang,[10] Xiao-Yu Chen,[11] Fang Yi,[1] Ya-Fang Zhou,[1] Hong-Yu Long,[2] Chao-Jun Zhou,[2] Xuan Hou,[2] Jun-Pu Wang,[6] Bin Xie,[6] Fan Liang,[7] Zhuan-Yi Yang,[11] Qi-Ying Sun,[1] Emily G. Allen,[5] Andrew Mark Shafik,[5] Ha Eun Kong,[5] Ji-Feng Guo,[2] Xin-Xiang Yan,[2] Zheng-Mao Hu,[4,12] Kun Xia,[4,12] Hong Jiang,[2,13] Hong-Wei Xu,[1] Ran-Hui Duan,[4,12] Peng Jin,[5,15,*] Bei-Sha Tang,[1,2,3,15] and Lu Shen[2,3,15,*]

Neuronal intranuclear inclusion disease (NIID) is a slowly progressing neurodegenerative disease characterized by eosinophilic intranuclear inclusions in the nervous system and multiple visceral organs. The clinical manifestation of NIID varies widely, and both familial and sporadic cases have been reported. Here we have performed genetic linkage analysis and mapped the disease locus to 1p13.3-q23.1; however, whole-exome sequencing revealed no potential disease-causing mutations. We then performed long-read genome sequencing and identified a large GGC repeat expansion within human-specific *NOTCH2NLC*. Expanded GGC repeats as the cause of NIID was further confirmed in an additional three NIID-affected families as well as five sporadic NIID-affected case subjects. Moreover, given the clinical heterogeneity of NIID, we examined the size of the GGC repeat among 456 families with a variety of neurological conditions with the known pathogenic genes excluded. Surprisingly, GGC repeat expansion was observed in two Alzheimer disease (AD)-affected families and three parkinsonism-affected families, implicating that the GGC repeat expansions in *NOTCH2NLC* could also contribute to the pathogenesis of both AD and PD. Therefore, we suggest defining a term NIID-related disorders (NIIDRD), which will include NIID and other related neurodegenerative diseases caused by the expanded GGC repeat within human-specific *NOTCH2NLC*.

Introduction

Neuronal intranuclear inclusion disease (NIID [MIM: 603472]) is a rare multisystem neurodegenerative disease characterized by the pathology of eosinophilic intranuclear inclusions in the central, peripheral, and autonomic nervous systems, as well as in the visceral organs.[1,2] The first NIID-affected case subject was reported in 1968.[3] However, until 2011, only about 40 NIID-affected case subjects had been described worldwide, which were diagnosed by post-mortem brain biopsy.[1,4–13] Because eosinophilic intranuclear inclusions also exist in dermal cells of NIID-affected individuals,[14,15] skin biopsy has become a useful tool to confirm NIID diagnosis, and the number of reported cases has increased to more than 100 case subjects.[2]

Both familial and sporadic case subjects have been described.[2] The onset age of NIID varies widely and can be divided into three subgroups according to the age of onset: infant form, juvenile form, and adult form.[1] The clinical manifestation of adult-onset NIID can vary widely, including dementia,[9,16] peripheral neuropathy,[5,17] autonomic dysfunction,[17,18] cerebellar ataxia,[7,8] parkinsonism,[4,19,20] seizure,[21] stroke-like episodes,[22] disturbance of consciousness,[21] and encephalitic episodes.[4] Dementia is the most prominent symptom in sporadic NIID-affected case subjects. In familial NIID-affected case subjects, based on the initial and main symptoms, a subgrouping of adult-onset NIID-affected case subjects was suggested based on dementia-dominant and limb weakness-dominant phenotypes.[2] Considering the wide range in distribution of intranuclear inclusions in the nervous system and other organs, it is possible that there are additional phenotypes of NIID. The typical symmetrical high signal seen in corticomedullary junctions using diffusion weighted imaging (DWI) could be a powerful tool for screening NIID-affected case

[1]Department of Geriatrics, Xiangya Hospital, Central South University, Changsha, Hunan 410008, China; [2]Department of Neurology, Xiangya Hospital, Central South University, Changsha, Hunan 410008, China; [3]National Clinical Research Center for Geriatric Disorders, Xiangya Hospital, Central South University, Changsha, Hunan 410008, China; [4]Center for Medical Genetics, School of Life Sciences, Central South University, Changsha, Hunan 410008, China; [5]Department of Human Genetics, Emory University School of Medicine, Whitehead Research Building, Room 305A, 615 Michael Street, Atlanta, GA 30322, USA; [6]Department of Pathology, Xiangya Hospital, Central South University, Changsha, Hunan 410008, China; [7]GrandOmics Biosciences, Beijing 100000, China; [8]Department of Neurology, The First Affiliated Hospital of Zhengzhou University, Zhengzhou, Henan 450052, China; [9]Department of Neurology, The First Affiliated Hospital of University of South China, Hengyang, Hunan 421000, China; [10]Department of Neurology, The Third Xiangya Hospital, Central South University, Changsha, Hunan 410008, China; [11]Department of Neurosurgery, Xiangya Hospital, Central South University, Changsha, Hunan 410008, China; [12]Hunan Key Laboratory of Medical Genetics, Central South University, Changsha, Hunan 410008, China; [13]Key Laboratory of Hunan Province in Neurodegenerative Disorders, Central South University, Changsha, Hunan 410008, China
[14]These authors contributed equally to this work
[15]These authors contributed equally to this work
*Correspondence: shenlu@csu.edu.cn (L.S.), peng.jin@emory.edu (P.J.)
https://doi.org/10.1016/j.ajhg.2019.05.013.
© 2019 The Author(s). This is an open access article under the CC BY license (http://creativecommons.org/licenses/by/4.0/).

subjects.[2,15,23,24] In addition, almost all dementia-dominant NIID-affected case subjects and about 40% of limb weakness-dominant NIID-affected case subjects have been seen to have remarkable leukoencephalopathy on fluid-attenuated inversion recovery (FLAIR) images and T2-weighted (T2) images, suggesting that severe white matter hyperintensity may also be an indicator for NIID.[2,15]

The pathogenesis of NIID remains unknown. Immunohistochemically, the intranuclear inclusions are positive for ubiquitin and ubiquitin related proteins, including p62, SUMO1, FUS, MYO6, and OPTN-C proteins,[5,12,13,25,26] indicating that the ubiquitin-proteasome system in the nucleus may play a role in NIID. In addition, some intranuclear inclusions were found to stain positive for an anti-polyglutamine antibody 1C2[4,13] and also with an anti-ataxin3 antibody.[11,27] However, this could be the result of a cross-reaction. No CAG repeat expansions have been observed in NIID.[27]

Here we report the identification of an expanded GGC repeat in *NOTCH2NLC* (MIM: 618025) via long-read genome sequencing (LRS) in a five-generation Chinese Han NIID family, and the repeat expansions were validated by repeat-primed PCR (RP-PCR) and GC-rich PCR (GC-PCR) in three additional NIID-affected families as well as five sporadic NIID-affected case subjects. In addition, we found two Alzheimer disease (AD)-affected families (1.43%) and three parkinsonism-affected families (1.46%), with GGC expansions in *NOTCH2NLC* from a cohort that included 140 AD-affected families, 205 parkinsonism-affected families, 51 spinocerebellar ataxia (SCA)-affected families, 16 peripheral neuropathy-affected families, and 44 motor neuron disease (MND)-affected families. Further, skin biopsies confirmed the typical NIID pathology in these two AD-affected families and three parkinsonism-affected families, indicating that GGC repeat expansions could also contribute to AD and PD phenotypes. Thus, we suggest defining a term of NIID-related disorders (NIIDRD), which is a spectrum of diseases including NIID and other diseases caused by the GGC repeat expansions of *NOTCH2NLC*. The prevalence of NIIDRD is not rare, and screening for expanded GGC repeat within *NOTCH2NLC* should be done not only in dementia-affected and peripheral neuropathy-affected individuals, but also in parkinsonism-affected individuals.

Material and Methods

Study Participants

Twenty affected individuals from four NIID-affected families, five sporadic NIID-affected case subjects, and 211 healthy control subjects from mainland China were recruited for this study. All NIID-affected individuals were recruited from the National Clinical Research Center for Geriatric Disorders, Xiangya Hospital, and subjected to a thorough neurological examination by at least two experienced neurologists. We defined familial NIID-affected case subjects as having at least one NIID-affected case subject diagnosed by skin biopsy, and the additional family member(s) had to have either high signal intensity in the corticomedullary junction using DWI or a clinical examination by a neurologist that confirmed the concordance of phenotype with the pathologically diagnosed NIID individual from the same family. All sporadic NIID-affected case subjects presented with typical high intensity in corticomedullary junction in DWI and received a skin biopsy to confirm the diagnosis. All healthy control subjects were recruited from the medical examination center of Xiangya hospital.

Besides NIID cohorts, a cohort of individuals with other neurodegenerative disorders were also recruited, including 140 AD-affected families, 205 parkinsonism-affected families, 51 SCA-affected families, 16 peripheral neuropathy-affected families, and 44 MND-affected families, of which the known pathologic genes had been excluded. All individuals were recruited from the Department of Neurology and National Clinical Research Center for Geriatric Disorders, Xiangya Hospital. All participants were subjected to a thorough neurological examination by at least two experienced neurologists.

This study was approved by the Ethics Committee of Xiangya Hospital of the Central South University in China. Written informed consent was obtained from all individuals.

Skin Biopsy, Immunohistochemistry, and Electron Microscopy

Skin biopsies were performed after local anesthesia. A 5-mm-diameter biopsy specimen was obtained at 10 cm above the lateral malleolus of the affected individual. For immunohistochemistry, all samples were fixed in 10% formalin and then were embedded in paraffin and sectioned at 6 mm thickness. Sections of all samples were stained by hematoxylin & eosin (H&E) and immunohistochemical analysis was performed. Anti-ubiquitin (3936; Cell Signaling) and anti-p62 (610833; BD Biosciences) antibodies were used for ubiquitin and p62 staining. Images were acquired by deconvolution digital microscope (Axioplan 2; Carl Zeiss).

Samples for electron microscopy were sliced into $1 \times 1 \times 3$ mm^3 size and fixed in 2.5% glutaraldehyde solution with Millonig's phosphate buffer (pH 7.3). In the following preparations, the samples were incubated in 1% osmium tetroxide, then dehydrated with graded acetone, and embedded with resin. 50–100 nm ultrathin sections were prepared with an ultramicrotome (Leica Microsystems) and a diamond knife. After 3% uranyl acetate and lead nitrate double staining, prepared sections were examined and photographed on a Hitachi HT-7700 electron microscope.

Linkage Analysis and Whole-Exome Sequencing

Whole-genome SNP scanning on selected members in family 1 (Figure 1A) was performed using Illumina Asian Screening Array with more than 7 million polymorphic loci. Information on SNPs was extracted by the genotyping module of Genomestudio (v.2011.1). Illumina cnvPartition CNV Analysis Plug-in of Genomestudio (v.2011.1) was used for copy number variations (CNVs) analysis. Linkage analysis was performed by the parametric linkage analysis package of MERLIN (v.1.1.2). Whole-exome sequencing (WES) and analysis for the three affected individuals (F1-IV:1, F1-IV:6, and F1-IV:10) and one unaffected individual (F1-IV:3) were used to detect potential causal single nucleotide variants (SNVs) and InDels (insertions and deletions) in the mapping region. Sequencing data were subjected to quality control (QC). This was followed by Burrows-Wheeler Alignment Maximal Exact Matches (BWA-MEM) for read mapping, Samtools for SNV/InDel calling, ANNOVAR for variant annotation, and all

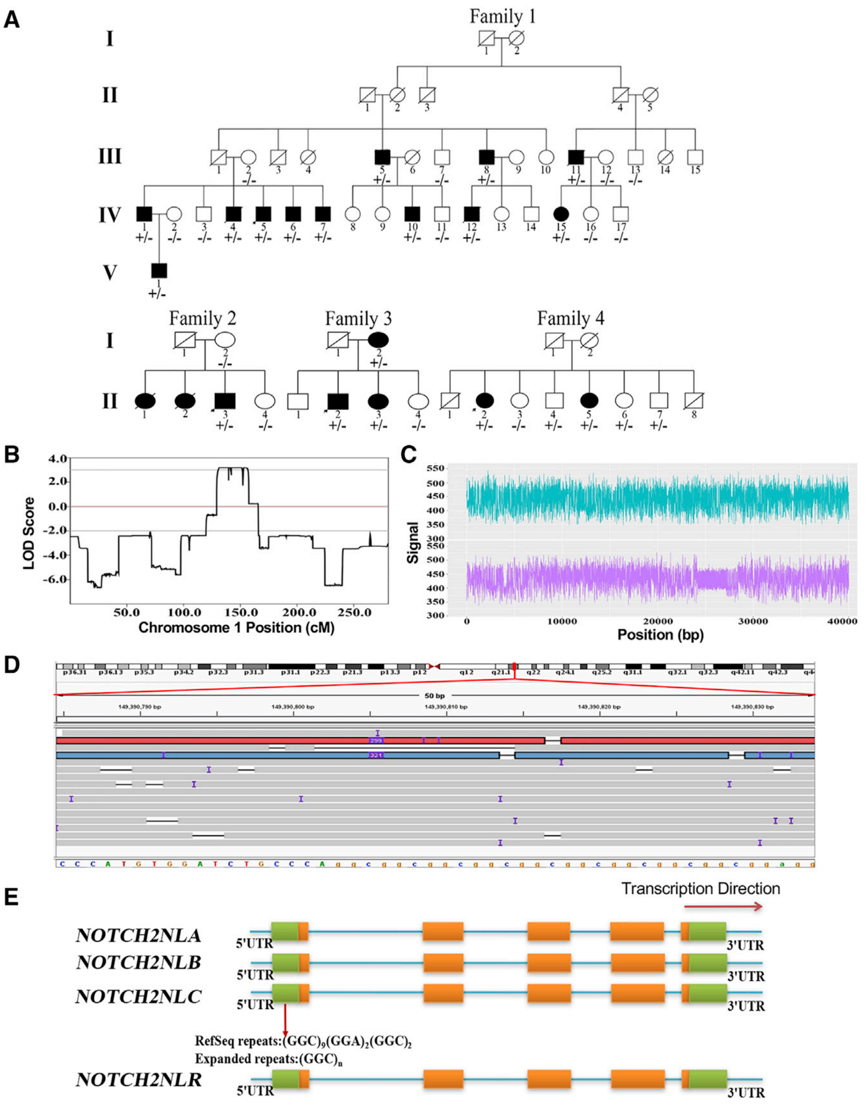

Figure 1. Identification of Expanded GGC Repeat within *NOTCH2NLC* in Neuronal Intranuclear Inclusion Disease

(A) Pedigrees of neuronal intranuclear inclusion disease (NIID)-affected families and corresponding individual genotypes.

(B) Genetic linkage analysis indicated maximum logarithm of odds (LOD) scores 3.184 in chromosome 1, a 49.8-Mb region at 1p13.3-q23.1 (chr1:109260034-159016186).

(C and D) GGC expansions detected by LRS. Nanopore electric signal (C) from subject F1-IV:15 indicated GGC expansions in the lower lane compared to the normal allele in the upper lane. More than ten reads covering the causative region were seen in the Integrative Genomics Viewer for subject F1-IV:15. Two reads were determined to carry the "insertion" variation (chr1:149390803-149390842, hg38 version), corresponding to the GGC triplet expansion in *NOTCH2NLC* (D).

(E) Schematic representation of the causal variant in *NOTCH2NLC*: a certain number of GGC triplets exist in 5′ UTR of *NOTCH2NLC* in healthy individuals, and large expanded GGC triplets are present in affected individuals.

potential disease variants were examined in Integrative Genomics Viewer.

Long-Read Genome Sequencing

Long-read genome sequencing (LRS) on seven affected individuals (F1-IV:7, F1-IV:15, F2-II:3, F4-II:2, F5-II:1, F5-II:4, F9-II:6) and three healthy control subjects was performed on the Oxford Nanopore platform as describe previously.[28] Combined analysis strategy of RepeatHMM and inScan were used for detection in the target region. Minimap2 and nanopolish calls were used for analysis of DNA methylation.

PCR Assays and GGC Repeat Size Determination

For the repeat-primed PCR (RP-PCR) assay, a fluorescein (FAM)-labeled gene-specific primer (5′-CCTCAGCCCGATACTCACCAT-3′) and repeat-containing primers (5′-TACCAATACGCATCCCGC GATTTGTCTTA(CGG)$_5$-3′) were utilized for identifying the CGG repeat expansion. For the GC-rich PCR (GC-PCR) assay, the fluorescein (FAM)-labeled forward primer (5′-AGCGCCAGGGC CTGAGCCTTTGAAGCAG-3′) and reverse primer (5′-TCGCCCC AGGTGGCAGCCCCGGGCGCCGCGGAC-3′) were utilized for repeat size determination. Seven-deaza-2-deoxy guanosine triphosphate (deaza-dGTP) was used in place of dGTP. PCRs were performed using 50 ng of genomic DNA in a 25 μL reaction mix including 10X Expand Long Template Buffer 1, 2.5 mM $MgCl_2$ (Thermo Scientific, Cat#F-510Mg), 2% 2,4-dimethylsulfolane (Sigma, Cat#1003-78-7), 0.2 mM each of deaza-dGTP, dATP, dCTP, and dTTP, 0.2 μM primers (BioSune, Shanghai, China) and 1 U of DNA ploymerase from Expand Long Template PCR System (Roche, Cat#11681842001), using the following thermal conditions: 98°C for 4 min, followed by 30 cycles of 98°C for 45 s, 60°C for 45 s, 72°C for 4 min, and a final extension at 72°C for 7 min. The PCR products were subjected to capillary electrophoresis using the 3500xL Genetic Analyzer for Human Identification (Applied Biosystems). Allele sizes were determined using GeneScan 1000 ROX Size Standard (Applied Biosystems). GGC repeats less than 200 were determined directly by the size of PCR product as determined by ABI 3500xL and compared with the PCR products of known *FMR1* (MIM: 309550) premutation CGG repeat alleles of various sizes. For GGC repeats over 200, the size was determined using an Agilent 2100.

RNA Extraction and Quantitative RT-PCR

Human postmortem dorsolateral prefrontal cortex (DLPFC) tissue from the subjects of different ages (4, 15, 36, and 60 years old) were obtained from the NIH NeuroBioBank. Total RNA was isolated using Trizol (ThermoFisher). Before reverse transcription, total RNA was treated with ezDNase (Millipore) for 5 min at 37°C to remove contaminated genomic DNA, and then incubated at 55°C for 5 min to inactivate the ezDNase. After ezDNase treatment, total RNA was extracted with phenol: chloroform = 25:24 (pH 5.2) and precipitated with glycogen and ethanol. The reverse

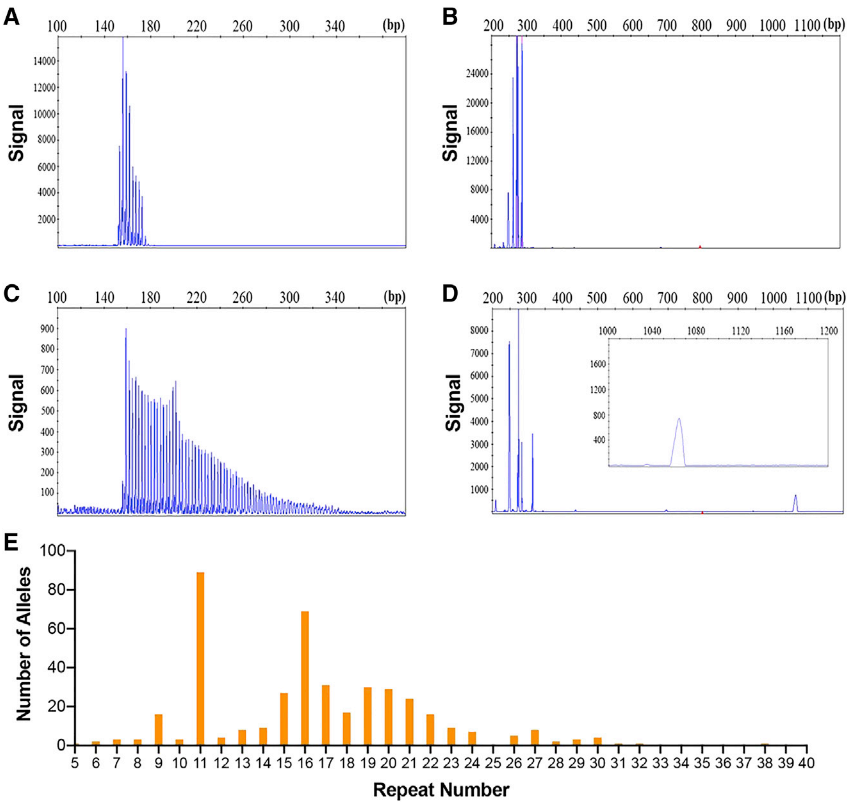

Figure 2. Validation of Expanded GGC Repeats and Variations of GGC Repeat Size among Normal Individuals

(A–D) Representative electropherogram of the RP-PCR assay and the GC-PCR assay showed abnormal repeat expansion in affected individuals (C and D) and negative result in control subjects (A and B).

(E) Size distribution of GGC repeat among healthy control subjects, which are usually less than 40 repeats.

transcription was conducted using SuperScript (II) (ThermoFisher, Cat# Cat. No. 18064-022) to generate first strand cDNA. The first strand cDNA served as the template for real-time PCR using FAST-7500 real time PCR equipment (Thermo Fisher). GAPDH TaqMan Gene Expression Assay (Hs02786624_g1) was used as an internal control. NOTCH2NLB primers: Forward (5′-GGGAGA TATGAAGGGACGCA-3′); Reverse (5′-GGCACACACCTTCCATT CTC-3′). NOTCH2NLC primers: Forward (5′-CTGACCTTTCAA GATCCTGCTTTCATCCCAGCT-3′); Reverse (5′-AAGTGCCTTACT TTGCGTAGCTGTGTGCTTGGCAGT-3′).

Statistical Analyses

For methylation analysis, the dispersion parameters were estimated through a shrinkage estimator based on a Bayesian hierarchical model, then a Wald test was performed as describe previously[29] to identify differentially methylated loci (DML). For the expression data of *NOTCH2NLC* in the blood of both NIID-affected individuals and control subjects, statistical analysis was performed using Student's t test. Differences with $p < 0.05$ were considered as being statistically significant.

Results

Identification of an Expanded GGC Repeat within Human-Specific *NOTCH2NLC* in a NIID-Affected Family

We recruited a five-generation Chinese NIID-affected family (family 1) with muscle weakness-dominant type (Figure 1A) and performed genetic linkage analysis. Our analysis identified a single peak with a maximum logarithm of odds (LOD) score of 3.184 at 1p13.3-q23.1 (chr1:109260034–159016186) (Figure 1B). To determine the genetic etiology of NIID in this family, we performed whole-exome sequencing (WES) in three affected individuals (F1-IV:1, F1-IV:6, and F1-IV:10) and one unaffected individual (F1-IV:3) (Figure 1A), and we were unable to identify any nonsynonymous or potentially causal variants. We went on to perform low-coverage long-read genome sequencing (LRS) on two other affected individuals (F1-IV:7, F1-IV:15) using the Oxford Nanopore platform. Intriguingly, we observed that one allele of *NOTCH2NLC* had an expanded GGC repeat in both affected individuals (Figures 1C and S1), which WES failed to detect.

The expanded GGC repeat is located at the 5′ end of *NOTCH2NLC*. *NOTCH2NLC* is one of the four NOTCH2 paralogs that are present only in the human genome; the other paralogs are *NOTCH2NLA* (MIM: 618023), *NOTCH2NLB* (MIM: 618024), and *NOTCH2NLR* (MIM: 618026). Previous studies have shown that all four NOTCH2NL paralogs contain the first four exons and introns of NOTCH2 as the result of a partial segmental duplication of the NOTCH2 ancestral gene.[30] *NOTCH2NLA*, *B*, and *C* are highly similar to each other, with more than 98% sequence identity over NOTCH2NL exons 1–5.[30] However, only *NOTCH2NLC* contains the GGC repeat in the reference human genome (hg38), $(GGC)_9(GGA)_2(GGC)_2$, which is expanded in the NIID-affected family that we have examined here (Figures 1D and 1E).

Expanded GGC Repeat Is Associated with NIID

To confirm our Nanopore sequencing results, we designed primer sets for both repeat-primed PCR (RP-PCR) and GC-rich PCR (GC-PCR) assays to detect both normal and expanded GGC repeats. Among the 211 healthy control subjects that we examined, the GGC repeat sizes range from 5 to 38, with modes at 11 and 16 repeats (Figure 2E). Our RP-PCR analysis confirmed the expanded GGC repeat among the affected family members in family 1 (Figure 1A). All affected individuals in this family showed similar peak patterns using RP-PCR, indicating the

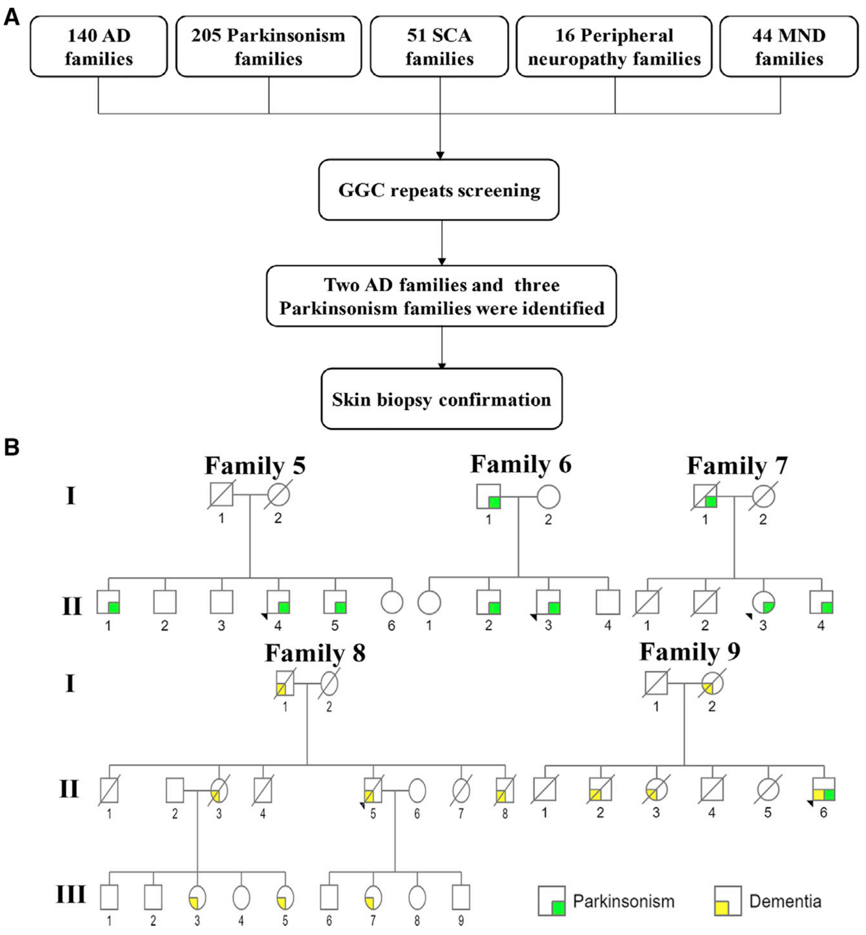

Figure 3. Expanded GGC Repeats Are Associated with Dementia and Parkinsonism
(A) Flow chart of GGC repeat expansion screening in a cohort of families with neurodegenerative disorders.
(B) Pedigrees of AD-affected and parkinsonism-affected families that carry expanded GGC repeats in *NOTCH2NLC*.

presence of expanded GGC repeats (Figure 2C), while unaffected members of the pedigree carried only normal repeats (Figure 2A). We then determined the repeat size using GC-PCR and found that all the affected individuals carry alleles with greater than 100 repeats (Figure 2D). To further confirm the role of GGC repeat expansion in NIID, we examined three additional NIID-affected families (families 2–4) (Figure 1A) and five sporadic NIID-affected case subjects by RP-PCR and GC-PCR assays, some of which were further confirmed by LRS (Figure S1). All affected individuals from both familial and sporadic case subjects carried an expanded GGC repeat larger than 66. Together, these results suggest that GGC repeat expansion is indeed associated with NIID.

However, we did note that the expanded GGC repeat was also observed in a few individuals that were initially thought to be phenotypically normal, such as F1-III:5 and F1-III:8 (Figures S2A and S2B). Upon re-examination of subject F1-III:8, we found that this individual has subclinical peripheral neuropathy and typical eosinophilic intranuclear inclusions (Figures S2C and S2D). Subject F1-III:5 did not complain of limb weakness and refused to receive further examination but presented with tremor and disturbance of consciousness, which are the classic symptoms of NIID. Nevertheless, this indicates the possibility of varying severity for expanded GGC repeat-caused NIID.

Expanded GGC Repeat Is Associated with Dementia and Parkinsonism

Because of the wide range of clinical manifestations associated with NIID, we further determined the size of the GGC repeat in a cohort of 140 families with AD, 205 families with parkinsonism, 51 families with SCA, 16 families with peripheral neuropathy, and 44 families with MND (Figure 3A). Surprisingly, we found affected individuals from three parkinsonism-affected families (families 5–7) and two AD-affected families (families 8 and 9) that carry the expanded GGC repeat in *NOTCH2NLC* (Figure 3B, Tables 1, S2, and S3). LRS was also performed on three affected individuals (F5-II:1, F5-II:4, F9-II:6), confirming the presence of expanded GGC repeat (Figure S1). Upon reexamination, we confirmed that these individuals display classic PD and AD symptoms, respectively. Intriguingly, their skin biopsies revealed typical eosinophilic, p62-positive, and ubiquitin-positive intranuclear inclusions, the pathological feature associated with classic NIID-affected individuals (Figures S3A–S3H and S3M–S3T). These findings suggest that the expanded GGC repeat could also be associated with dementia and parkinsonism.

Expanded GGC Repeat and Phenotypic Variability

A total of 40 affected familial individuals with expanded GGC repeats, ranging from 66 to 517 repeats, were analyzed in this study. Only about 37.5% of affected familial individuals presented with the classical NIID radiological findings of symmetrical hyperintense linear lesions in the corticomedullary junction in DWI (Figure S4A) and severe white matter hyperintensity in FLAIR images/T2 weighted image (Figure S4B). However, all skin biopsies revealed eosinophilic, p62-positive, and ubiquitin-positive intranuclear inclusions in dermal cells (Figure S3). Electron microscopy imaging also revealed intranuclear inclusions lacking membranes (Figure S3H). The main clinical manifestations in these affected familial individuals included bladder dysfunction (56.4%), tremor (47.5%), muscle weakness (46.2%), abnormal behavior (37.5%), sensory disturbance (35.1%), dementia (35.0%), rigidity (30.0%), bradykinesia (30.0%), ataxia (17.5%), disturbance

Table 1. Summary of Clinical Features of NIID

	Sporadic Case Subjects (n = 5)	Familial Case Subjects			
		Total (n = 40)	Muscle Weakness (n = 15)	Parkinsonism (n = 9)	Dementia (n = 16)
Sex ratio (male/female)	2/3	26/14	12/3	8/1	6/10
Average onset age (range)	62.0 (51–69)	50.6 (30–78)	35.6 (30–54)	60.6 (37–78)	58.1 (31–71)
Average disease duration (range)	5.6 (1–14)	12.5 (1–49)	16.6 (3–49)	6.0 (1–15)	12.4 (2–30)
**Clinical Manifestations**					
Dementia	2/5 (40.0%)	14/40 (35.0%)	0/15 (0%)	0/9 (0%)	14/16 (87.5%)
Abnormal behavior	2/5 (40.0%)	15/40 (37.5%)	2/15 (13.3%)	0/9 (0%)	13/16 (81.3%)
Peripheral neuropathy					
Muscle weakness	0/5 (0%)	18/39 (46.2%)	13/15 (86.7%)	1/9 (11.1%)	4/15 (26.7%)
Sensory disturbance	0/5 (0%)	13/37 (35.1%)	4/13 (30.8%)	4/9 (44.4%)	5/15 (33.3%)
Autonomic dysfunction					
Bladder dysfunction	3/5 (60.0%)	22/39 (56.4%)	5/15 (33.3%)	5/9 (55.6%)	12/15 (80.0%)
Miosis	2/5 (40.0%)	5/29 (17.2%)	1/11 (9.1%)	0/8 (0%)	4/10 (40.0%)
Parkinsonism					
Tremor	1/5 (20.0%)	19/40 (47.5%)	11/15 (73.3%)	5/9 (55.6%)	3/16 (18.8%)
Rigidity	1/5 (20.0%)	12/40 (30.0%)	1/15 (6.7%)	9/9 (100.0%)	2/16 (12.5%)
Bradykinesia	1/5 (20.0%)	12/40 (30.0%)	1/15 (6.7%)	9/9 (100.0%)	2/16 (12.5%)
Ataxia	0/5 (0%)	7/40 (17.5%)	2/15 (13.3%)	4/9 (44.4%)	1/16 (6.3%)
Neurological attack					
Disturbance of consciousness	4/5 (80.0%)	7/40 (17.5%)	3/15 (20.0%)	1/9 (11.1%)	3/16 (18.8%)
Stroke-like episode	4/5 (80.0%)	4/40 (10.0%)	1/15 (6.7%)	2/9 (22.2%)	1/16 (6.3%)
Encephalitic episode	3/5 (60.0%)	2/40 (5.0%)	1/15 (6.7%)	0/9 (0%)	1/16 (6.3%)
Brain MRI					
Severe leukoencephalopathy	5/5 (100.0%)	7/20 (35.0%)	1/7 (14.3%)	2/6 (33.3%)	4/7 (57.1%)
DWI U-fiber high signal	5/5 (100.0%)	6/16 (37.5%)	1/3 (33.3%)	1/6 (16.7%)	4/7 (57.1%)
Cognitive function test					
MMSE (<education matched average)	0/2 (0%)	4/21 (19.0%)	0/7 (0%)	0/7 (0%)	4/7 (57.1%)
MoCA (<education matched average)	1/2 (50.0%)	9/16 (56.3%)	3/7 (42.9%)	4/5 (80.0%)	2/4 (50.0%)
Nerve conduction					
MCV slowing	3/3 (100.0%)	19/22 (86.4%)	8/9 (88.9%)	6/6 (100%)	5/7 (71.4%)
CMAP reduction	1/3 (33.3%)	14/22 (63.6%)	7/9 (77.8%)	2/6 (33.3%)	5/7 (71.4%)
SCV slowing	3/3 (100.0%)	15/22 (68.2%)	7/9 (77.8%)	3/6 (50.0%)	5/7 (71.4%)
SNAP reduction	1/3 (33.3%)	13/22 (59.1%)	6/9 (66.7%)	2/6 (33.3%)	5/7 (71.4%)
Skin biopsy	5	14	3	5	6
Average no. of GGC repeats (range)	105 (86–133)	188 (66–517)	272 (118–517)	83 (66–102)	129 (91–268)

of consciousness (17.5%), miosis (17.2%), stroke-like episodes (10.0%), and encephalitic episodes (5.0%) (Table 1). Based on the initial and main clinical manifestations among these affected individuals with expanded repeats, we were able to divide the familial case subjects into three subgroups: muscle weakness-dominant type, parkinsonism-dominant type, and dementia-dominant type (Table 1 and Supplemental Note).

The muscle weakness-dominant type (families 1 and 2) was similar to what has been reported for classic NIID where the main and initial clinical manifestation is muscle weakness. The average onset age was around 36 (Figure 1A,

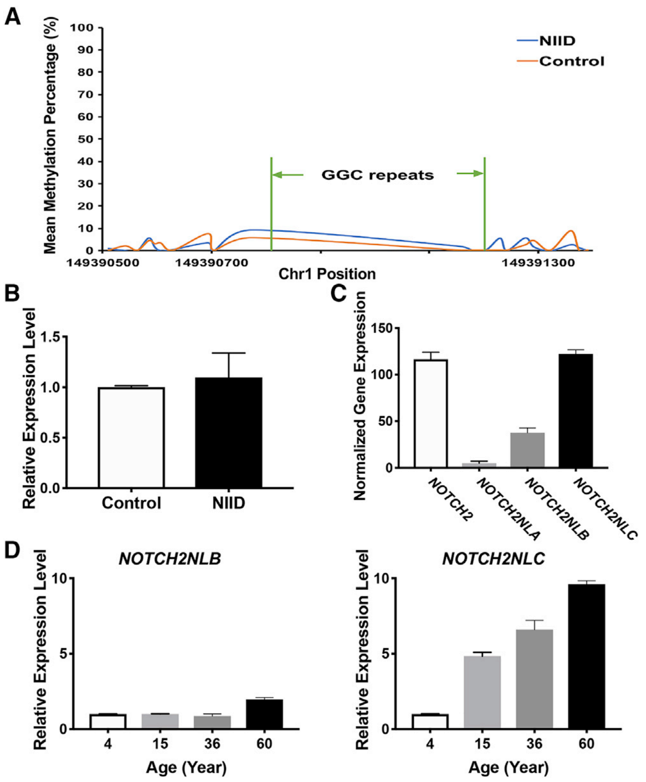

Figure 4. Methylation and Expression at *NOTCH2NLC* Locus
(A) Methylation status across expanded GGC repeats region was determined using LRS data from seven affected individuals (F1-IV:7, F1-IV:15, F2-II:3, F4-II:2, F5-II:1, F5-II:4, F9-II:6) and three healthy control subjects, and no significant methylation difference was detected between NIID-affected case subjects and control subjects. Wald test was performed for statistical analysis; *p < 0.05.
(B) *NOTCH2NLC* expression level in both NIID-affected case subjects (NIID) and normal control subjects (Control). The ezDNase-treated total RNA isolated from the blood of both NIID-affected case subjects and control subjects was reversely transcribed into cDNA followed by quantitative PCR. *GAPDH* was used as internal control. Error bars represent the SD; Student's t test was performed for statistical analysis; *p < 0.05. p $=$ 0.776 (ns).
(C) Expression levels of *NOTCH2* and three *NOTCH2NL* paralogs (*NOTCH2NLA*, *NOTCH2NLB*, and *NOTCH2NLC*) in human adult cortex detected by RNA-seq. Significant differences were observed in the expression levels of these four genes. Shown are the normalized gene expression levels. Error bars represent the SD.
(D) Dynamic change of *NOTCH2NLC* expression in human brain during aging. Relative expression levels of both *NOTCH2NLB* (left) and *NOTCH2NLC* (right) in DLPFC region of human brain during aging are shown. The total RNA isolated from the DLPFC of human postmortem brains from 4-, 15-, 36-, and 60-year-old subjects were used for quantitative RT-PCR with *GAPDH* as internal control. Error bars represent the SD.

Tables 1 and S1). Muscle weakness usually began in the distal part of lower limbs, then moved up to the throat muscles and face. However, the severity and position of muscle weakness could vary significantly between individuals. Tremor was frequently seen in the early stages of almost all of the affected members. The number of GGC repeats in this subgroup ranged from 118 to 517 repeats.

The parkinsonism-dominant type (families 5–7) presented with parkinsonism as the main clinical manifestation. The onset age was typically around 60 years old (Figure 3B, Tables 1 and S2). The severity of symptoms varied greatly, even between siblings with similar repeat sizes. No dementia was observed in either the muscle weakness or parkinsonism groups. A smaller repeat expansion, 66–102 repeats, was observed in this group.

In the dementia-dominant type (families 3, 4, 8, 9), individual characteristics were similar to the previously described dementia-dominant phenotype in NIID.[2] The average onset age was 58. Dementia was the most prominent symptom (Figures 1A and 3B, Tables 1 and S3). Abnormal behavior, autonomic dysfunction, disturbance of consciousness, tremor, rigidity, limb weakness, and sensory disturbance were also seen. This subgroup repeat size ranged from 91 to 268 GGC repeats.

Methylation and Expression at *NOTCH2NLC* Locus

Previous works have found that more than 200 CGG repeats at the *FMR1* locus can lead to hypermethylation of the CGG repeat and adjacent CpG islands, resulting in transcriptional silencing.[31] Because many of the expanded repeats that we discovered at the *NOTCH2NLC* locus in NIID-affected case subjects were greater than 200, we examined the methylation status of GGC repeat and adjacent regions by re-analyzing the signals from Nanopore sequencing. The methylation level around the GGC repeats was very low and no significant methylation difference between NIID and control was detected (Figure 4A), which suggests that the expanded GGC repeat at the *NOTCH2NLC* locus is likely unmethylated. Furthermore, we determined the expression of *NOTCH2NLC* in the blood of both NIID-affected case subjects and control subjects, and did not detect significant changes in expression for the NIID-affected case subjects with expanded GGC repeats (Figure 4B), indicating that the expanded GGC repeat does not alter the expression of *NOTCH2NLC* and that the GGC repeat RNA could potentially play a role in the molecular pathogenesis of NIID.

Previous studies have shown that *NOTCH2NLA*, *NOTCH2NLB*, and *NOTCH2NLC* can be transcribed in human ES cells.[30,32] We analyzed the expression of these three paralogs and *NOTCH2* using the published human brain RNA-seq datasets[33] and found that *NOTCH2NLC* displays the highest expression in the brain among these three NOTCH2 paralogs (Figure 4C). Given that NIID is a neurodegenerative disorder, we designed specific primers that could distinguish the expression of *NOTCH2NLB* and *NOTCH2NLC* by RT-PCR (*NOTCH2NLA* has very low expression in brain; Figure 4C) and determined the expression dynamics of both genes during human brain aging. Interestingly, while the expression of *NOCTH2NLB* displays only modest changes in human prefrontal cortex during aging, the expression of *NOTCH2NLC* increased significantly with age (Figure 4D). Thus, the increased

expression of expanded GGC repeats during aging could contribute to the molecular pathogenesis of NIID.

Discussion

Neuronal intranuclear inclusion disease (NIID) is a slowly progressing neurodegenerative disease characterized by eosinophilic intranuclear inclusions in the nervous system and multiple visceral organs. The clinical manifestation of NIID varies widely, and both familial and sporadic case subjects have been reported.[2] Here we report the identification of a GGC repeat expansion at the 5′ end of *NOTCH2NLC* as the genetic cause of NIID. *NOTCH2NLC* is one of the three human-specific *NOTCH2*-related genes (*NOTCH2NLA*, *NOTCH2NLB*, and *NOTCH2NLC*) in 1q21.1, highly expressed in the brain and thought to be involved in the evolutionary expansion of the human brain.[30,32] Thus, NIID represents a genetic disorder caused by an expanded repeat in a human-specific gene.

Almost 30 years ago, expansion of unstable nucleotide (microsatellite) repeats, notably trinucleotide repeats, was identified as a previously unknown mutational mechanism underlying certain human diseases.[34–37] In recent years the development of next-generation sequencing along with the focus on exome capture provided a massive increase in the catalog of genes with mutations in Mendelian disorders;[38–50] however, in many instances the causal mutations could not be identified even when genome-wide association studies (GWASs) or genetic linkage analysis identified a specific genetic locus. Recent works, including identification of an unstable hexanucleotide repeat within *C9ORF72* (MIM: 614260) as a frequent cause of frontotemporal dementia (FTD)/amyotrophic lateral sclerosis (ALS)[51,52] and CTG repeat expansion as the cause of Fuch's endothelial corneal dystrophy (FECD),[53] suggest that repeat expansions could contribute to the genetic etiology of these disorders, which could be missed by short-read sequencing. In our initial analyses, we failed to detect any causal mutations using WES despite the fact that we mapped the NIID-causing gene to 1p13.3-q23.1. Our findings presented here further highlight the importance of considering the genetic contribution of unstable repeats in disorders where traditional single nucleotide variants (SNVs) or copy number variants (CNVs) cannot be identified.

In addition to GGC repeats, we did observe different repeat units, such as GGT, through our LRS analyses (Figure S1). This is likely due to the high error rate of LRS itself and interference of sequence from paralogs. With the high error rate of Nanopore sequencing, it was challenging to acquire accurate sequence data within the expanded GGC repeat region, which would require further analyses with more accurate methodology. Despite these technical issues, most reads that we obtained were expanded GGC repeats.

The key features associated with the disorders caused by unstable nucleotide (microsatellite) repeats are genetic instability and anticipation.[31] In the NIID-affected families reported in our study, a wide range (66–517) of repeat numbers were observed in affected individuals, while control subjects have fewer than 40 repeats. Anticipation was seen in a few families, while unlike fragile X-related disorders, the pioneer locus with expanded CGG/GGC repeats, increased instability of the GGC repeat size over generations was not observed in all families. Therefore, anticipation was not clearly seen in NIID-affected families, and more NIID-affected families need to be studied to explore the genetic pattern over multiple generations. Nevertheless, the number of affected individuals does seem to increase over multiple generations, suggesting a potential genetic anticipation effect. We also observed variable severity in the clinical manifestation among our affected case subjects; however, there is no apparent association between GGC repeat size and the severity or the onset age in NIID. It is possible that additional genetic or environmental factors could contribute to the pathogenesis of NIID. In addition, extensive works on the *FMR1* locus have shown that there is a threshold for the methylation status of the CGG repeat.[31] Typically, if the CGG repeat is below 200, it is unmethylated, but the majority of expanded CGG repeats over 200 are hypermethylated, leading to transcriptional silencing of *FMR1* and fragile X syndrome.[31] Thus, it has been hypothesized that loci with more than 200 CGG repeats could be recognized by DNA methyltransferase(s) and induce hypermethylation.[54] Similar observations have recently been reported in Baratela-Scott syndrome (BSS), which was found due to an expanded GGC repeat in *XYLT1* (MIM: 608124).[55] However, as reported here, many expanded alleles associated with NIID-affected individuals exceeded 200 repeats but were not methylated. Our finding suggests that in addition to GGC/CGG repeat size, additional factor(s) or sequence(s) could also determine the methylation status of expanded GGC/CGG repeat *in vivo*.

Our analyses of both classic NIID and other individuals with neurological conditions revealed a broadened clinical spectrum of adult-onset NIID. Besides the suggested dementia-dominant type and muscle weakness-dominant type,[2] we have identified the parkinsonism-dominant type as a clinical presentation in NIID. Among sporadic case subjects, we also identified a paroxysmal disease-dominant type (Supplemental Note, Table S4). Furthermore, GGC repeat expansion was also observed in two AD-affected families (1.43%) and three parkinsonism-affected families (1.46%), implicating that GGC repeat expansions in *NOTCH2NLC* could also contribute to the pathogenesis of both AD and PD. Therefore, we suggest defining a term NIID-related disorders (NIIDRD), which include NIID and other related neurodegenerative diseases caused by the expanded GGC repeat. It is likely that the prevalence of NIIDRD could be higher than initially thought. Given that only about 37% of affected individuals showed typical MRI features of NIID, genetic testing

for the expanded GGC repeat could potentially be a more sensitive and accurate screening tool for NIIDRD.

Identification of the GGC repeat expansion as the genetic cause of NIID will help elucidate the molecular pathogenesis in NIID. Our results presented here suggest that the GGC repeat expansion-induced pathogenesis is likely dominant, which suggests that the expression of the expanded GGC repeat could cause the neuronal toxicity associated with NIID and its related disorders. Indeed, a dominant-negative gain-of-function model has been proposed for another neurodegenerative disorder, fragile X-associated tremor/ataxia syndrome (FXTAS [MIM: 300623]), which is also caused by the expanded GGC/CGG repeats.[56] Intriguingly, it was previously discussed that NIID and FXTAS share many clinical and neuropathological features,[27,58] which could be well explained by our genetic findings presented here. Two potential mechanisms have been proposed for FXTAS: RNA toxicity and repeat-associated non-AUG (RAN) translation.[59] The CGG repeat RNA could sequester key RNA binding proteins and prevent them from performing their normal physiological function.[56] Also, repeat-associated non-AUG (RAN) translation of the CGG repeat into polypeptides, the predominant species being poly-Glycine (FMRpolyG peptides), could contribute to FXTAS pathogenesis.[60,61] Both *FMR1* mRNA and FMRpolyG peptide have been found in the human postmortem brain inclusions that are characteristic of FXTAS pathology, suggesting that both mechanisms may contribute to the disease.[60,62] It will be important to explore both mechanisms in NIID pathogenesis in the future. In addition, it could be challenging to establish appropriate model(s) to study NIID since *NOTCH2NLC* is a human-specific gene.

In summary, we have identified an expanded GGC repeat within human-specific *NOTCH2NLC* as the genetic cause of NIIDRD, which could include dementia, parkinsonism, and peripheral neuropathy. Our finding will facilitate more accurate clinical diagnosis in the future and help us better understand the molecular pathogenesis of NIIDRD in general.

Supplemental Data

Supplemental Data can be found online at https://doi.org/10.1016/j.ajhg.2019.05.013.

Acknowledgments

We would like to thank the affected individuals for permitting us to publish this information and NIH NeuroBioBank for providing human brain tissues. This work was funded by National Key R&D Program of China (Grant2018YFC1312003) and National Natural Science Foundation of China (Grant81430023, Grant81701263, Grant81671075, Grant81771366, and Grant81671120).

Declaration of Interests

The authors declare no competing interests.

Received: April 23, 2019
Accepted: May 17, 2019
Published: June 6, 2019

Web Resources

GenBank, https://www.ncbi.nlm.nih.gov/genbank/
Genomestudio (v.2011.1), https://support.illumina.com/array/array_software/genomestudio/downloads.html
gnomAD Browser, https://gnomad.broadinstitute.org/
IGV, http://www.broadinstitute.org/igv/
inScan, https://github.com/Nextomics/inScan
MERLIN (v.1.1.2), http://csg.sph.umich.edu/abecasis/Merlin/download/
Minimap2, https://github.com/lh3/minimap2
Nanopolish call, https://github.com/jts/nanopolish
OMIM, http://www.omim.org/
RepeatHMM, https://github.com/WGLab/RepeatHMM
UCSC Genome Browser, https://genome.ucsc.edu

References

1. Takahashi-Fujigasaki, J. (2003). Neuronal intranuclear hyaline inclusion disease. Neuropathology *23*, 351–359.
2. Sone, J., Mori, K., Inagaki, T., Katsumata, R., Takagi, S., Yokoi, S., Araki, K., Kato, T., Nakamura, T., Koike, H., et al. (2016). Clinicopathological features of adult-onset neuronal intranuclear inclusion disease. Brain *139*, 3170–3186.
3. Lindenberg, R., Rubinstein, L.J., Herman, M.M., and Haydon, G.B. (1968). A light and electron microscopy study of an unusual widespread nuclear inclusion body disease. A possible residuum of an old herpesvirus infection. Acta Neuropathol. *10*, 54–73.
4. Liu, Y., Mimuro, M., Yoshida, M., Hashizume, Y., Niwa, H., Miyao, S., Ujihira, N., and Akatsu, H. (2008). Inclusion-positive cell types in adult-onset intranuclear inclusion body disease: implications for clinical diagnosis. Acta Neuropathol. *116*, 615–623.
5. Kimber, T.E., Blumbergs, P.C., Rice, J.P., Hallpike, J.F., Edis, R., Thompson, P.D., and Suthers, G. (1998). Familial neuronal intranuclear inclusion disease with ubiquitin positive inclusions. J. Neurol. Sci. *160*, 33–40.
6. Schuffler, M.D., Bird, T.D., Sumi, S.M., and Cook, A. (1978). A familial neuronal disease presenting as intestinal pseudoobstruction. Gastroenterology *75*, 889–898.
7. Michaud, J., and Gilbert, J.J. (1981). Multiple system atrophy with neuronal intranuclear hyaline inclusions. Report of a new case with light and electron microscopic studies. Acta Neuropathol. *54*, 113–119.
8. Patel, H., Norman, M.G., Perry, T.L., and Berry, K.E. (1985). Multiple system atrophy with neuronal intranuclear hyaline inclusions. Report of a case and review of the literature. J. Neurol. Sci. *67*, 57–65.
9. Munoz-Garcia, D., and Ludwin, S.K. (1986). Adult-onset neuronal intranuclear hyaline inclusion disease. Neurology *36*, 785–790.
10. Weidenheim, K.M., and Dickson, D.W. (1995). Intranuclear inclusion bodies in an elderly demented woman: a form of intranuclear inclusion body disease. Clin. Neuropathol. *14*, 93–99.
11. Takahashi, J., Tanaka, J., Arai, K., Funata, N., Hattori, T., Fukuda, T., Fujigasaki, H., and Uchihara, T. (2001).

Recruitment of nonexpanded polyglutamine proteins to intranuclear aggregates in neuronal intranuclear hyaline inclusion disease. J. Neuropathol. Exp. Neurol. *60*, 369–376.
12. Pountney, D.L., Huang, Y., Burns, R.J., Haan, E., Thompson, P.D., Blumbergs, P.C., and Gai, W.P. (2003). SUMO-1 marks the nuclear inclusions in familial neuronal intranuclear inclusion disease. Exp. Neurol. *184*, 436–446.
13. Mori, F., Miki, Y., Tanji, K., Ogura, E., Yagihashi, N., Jensen, P.H., and Wakabayashi, K. (2011). Incipient intranuclear inclusion body disease in a 78-year-old woman. Neuropathology *31*, 188–193.
14. Sone, J., Tanaka, F., Koike, H., Inukai, A., Katsuno, M., Yoshida, M., Watanabe, H., and Sobue, G. (2011). Skin biopsy is useful for the antemortem diagnosis of neuronal intranuclear inclusion disease. Neurology *76*, 1372–1376.
15. Sone, J., Kitagawa, N., Sugawara, E., Iguchi, M., Nakamura, R., Koike, H., Iwasaki, Y., Yoshida, M., Takahashi, T., Chiba, S., et al. (2014). Neuronal intranuclear inclusion disease cases with leukoencephalopathy diagnosed via skin biopsy. J. Neurol. Neurosurg. Psychiatry *85*, 354–356.
16. Araki, K., Sone, J., Fujioka, Y., Masuda, M., Ohdake, R., Tanaka, Y., Nakamura, T., Watanabe, H., and Sobue, G. (2016). Memory Loss and Frontal Cognitive Dysfunction in a Patient with Adult-onset Neuronal Intranuclear Inclusion Disease. Intern. Med. *55*, 2281–2284.
17. Sone, J., Hishikawa, N., Koike, H., Hattori, N., Hirayama, M., Nagamatsu, M., Yamamoto, M., Tanaka, F., Yoshida, M., Hashizume, Y., et al. (2005). Neuronal intranuclear hyaline inclusion disease showing motor-sensory and autonomic neuropathy. Neurology *65*, 1538–1543.
18. Nakamura, M., Ueki, S., Kubo, M., Yagi, H., Sasaki, R., Okada, Y., Akiguchi, I., Kusaka, H., and Kondo, T. (2018). Two cases of sporadic adult-onset neuronal intranuclear inclusion disease preceded by urinary disturbance for many years. J. Neurol. Sci. *392*, 89–93.
19. O'Sullivan, J.D., Hanagasi, H.A., Daniel, S.E., Tidswell, P., Davies, S.W., and Lees, A.J. (2000). Neuronal intranuclear inclusion disease and juvenile parkinsonism. Mov. Disord. *15*, 990–995.
20. Motoki, M., Nakajima, H., Sato, T., Tada, M., Kakita, A., and Arawaka, S. (2018). Neuronal intranuclear inclusion disease showing intranuclear inclusions in renal biopsy 12 years earlier. Neurology *91*, 884–886.
21. Toyota, T., Huang, Z., Nohara, S., Okada, K., Kakeda, S., Korogi, Y., Nakayama, T., Sone, J., Sobue, G., and Adachi, H. (2015). Neuronal intranuclear inclusion disease manifesting with new-onset epilepsy in the elderly. Neurol. Clin. Neurosci. *3*, 238–240.
22. Fujita, K., Osaki, Y., Miyamoto, R., Shimatani, Y., Abe, T., Sumikura, H., Murayama, S., Izumi, Y., and Kaji, R. (2017). Neurologic attack and dynamic perfusion abnormality in neuronal intranuclear inclusion disease. Neurol. Clin. Pract. *7*, e39–e42.
23. Abe, K., and Fujita, M. (2017). Over 10 years MRI observation of a patient with neuronal intranuclear inclusion disease. BMJ Case Rep. *2017*, bcr2016218790.
24. Sugiyama, A., Sato, N., Kimura, Y., Maekawa, T., Enokizono, M., Saito, Y., Takahashi, Y., Matsuda, H., and Kuwabara, S. (2017). MR Imaging Features of the Cerebellum in Adult-Onset Neuronal Intranuclear Inclusion Disease: 8 Cases. AJNR Am. J. Neuroradiol. *38*, 2100–2104.
25. Nakamura, M., Murray, M.E., Lin, W.-L., Kusaka, H., and Dickson, D.W. (2014). Optineurin immunoreactivity in neuronal and glial intranuclear inclusions in adult-onset neuronal intranuclear inclusion disease. Am. J. Neurodegener. Dis. *3*, 93–102.
26. Mori, F., Tanji, K., Kon, T., Odagiri, S., Hattori, M., Hoshikawa, Y., Kono, C., Yasui, K., Yokoi, S., Hasegawa, Y., et al. (2012). FUS immunoreactivity of neuronal and glial intranuclear inclusions in intranuclear inclusion body disease. Neuropathol. Appl. Neurobiol. *38*, 322–328.
27. Gelpi, E., Botta-Orfila, T., Bodi, L., Marti, S., Kovacs, G., Grau-Rivera, O., Lozano, M., Sánchez-Valle, R., Muñoz, E., Valldeoriola, F., et al. (2017). Neuronal intranuclear (hyaline) inclusion disease and fragile X-associated tremor/ataxia syndrome: a morphological and molecular dilemma. Brain *140*, e51.
28. Zeng, S., Zhang, M.-y., Wang, X., Hu, Z., Li, J., Li, N., Wang, J., Liang, F., Yang, Q., Liu, Q., et al. (2019). Long-read sequencing identified intronic repeat expansions in *SAMD12* from Chinese pedigrees affected with familial cortical myoclonic tremor with epilepsy. J. Med. Genet. *56*, 265–270.
29. Feng, H., Conneely, K.N., and Wu, H. (2014). A Bayesian hierarchical model to detect differentially methylated loci from single nucleotide resolution sequencing data. Nucleic Acids Res. *42*, e69.
30. Fiddes, I.T., Lodewijk, G.A., Mooring, M., Bosworth, C.M., Ewing, A.D., Mantalas, G.L., Novak, A.M., van den Bout, A., Bishara, A., Rosenkrantz, J.L., et al. (2018). Human-Specific NOTCH2NL Genes Affect Notch Signaling and Cortical Neurogenesis. Cell *173*, 1356–1369.e22.
31. Nelson, D.L., Orr, H.T., and Warren, S.T. (2013). The unstable repeats–three evolving faces of neurological disease. Neuron *77*, 825–843.
32. Suzuki, I.K., Gacquer, D., Van Heurck, R., Kumar, D., Wojno, M., Bilheu, A., Herpoel, A., Lambert, N., Cheron, J., Polleux, F., et al. (2018). Human-Specific NOTCH2NL Genes Expand Cortical Neurogenesis through Delta/Notch Regulation. Cell *173*, 1370–1384.e16.
33. Wang, T., Pan, Q., Lin, L., Szulwach, K.E., Song, C.X., He, C., Wu, H., Warren, S.T., Jin, P., Duan, R., and Li, X. (2012). Genome-wide DNA hydroxymethylation changes are associated with neurodevelopmental genes in the developing human cerebellum. Hum. Mol. Genet. *21*, 5500–5510.
34. Verkerk, A.J.M.H., Pieretti, M., Sutcliffe, J.S., Fu, Y.-H., Kuhl, D.P.A., Pizzuti, A., Reiner, O., Richards, S., Victoria, M.F., Zhang, F.P., et al. (1991). Identification of a gene (FMR-1) containing a CGG repeat coincident with a breakpoint cluster region exhibiting length variation in fragile X syndrome. Cell *65*, 905–914.
35. Vincent, A., Heitz, D., Petit, C., Kretz, C., Oberlé, I., and Mandel, J.-L. (1991). Abnormal pattern detected in fragile-X patients by pulsed-field gel electrophoresis. Nature *349*, 624–626.
36. La Spada, A.R., Wilson, E.M., Lubahn, D.B., Harding, A.E., and Fischbeck, K.H. (1991). Androgen receptor gene mutations in X-linked spinal and bulbar muscular atrophy. Nature *352*, 77–79.
37. Orr, H.T., Chung, M.Y., Banfi, S., Kwiatkowski, T.J., Jr., Servadio, A., Beaudet, A.L., McCall, A.E., Duvick, L.A., Ranum, L.P.W., and Zoghbi, H.Y. (1993). Expansion of an unstable trinucleotide CAG repeat in spinocerebellar ataxia type 1. Nat. Genet. *4*, 221–226.
38. Brook, J.D., McCurrach, M.E., Harley, H.G., Buckler, A.J., Church, D., Aburatani, H., Hunter, K., Stanton, V.P., Thirion, J.-P., Hudson, T., et al. (1992). Molecular basis of myotonic

dystrophy: expansion of a trinucleotide (CTG) repeat at the 3′ end of a transcript encoding a protein kinase family member. Cell *68*, 799–808.

39. MacDonald, M.E., Ambrose, C.M., Duyao, M.P., Myers, R.H., Lin, C., Srinidhi, L., Barnes, G., Taylor, S.A., James, M., Groot, N., et al.; The Huntington's Disease Collaborative Research Group (1993). A novel gene containing a trinucleotide repeat that is expanded and unstable on Huntington's disease chromosomes. Cell *72*, 971–983.
40. Knight, S.J.L., Flannery, A.V., Hirst, M.C., Campbell, L., Christodoulou, Z., Phelps, S.R., Pointon, J., Middleton-Price, H.R., Barnicoat, A., Pembrey, M.E., et al. (1993). Trinucleotide repeat amplification and hypermethylation of a CpG island in FRAXE mental retardation. Cell *74*, 127–134.
41. Nagafuchi, S., Yanagisawa, H., Sato, K., Shirayama, T., Ohsaki, E., Bundo, M., Takeda, T., Tadokoro, K., Kondo, I., Murayama, N., et al. (1994). Dentatorubral and pallidoluysian atrophy expansion of an unstable CAG trinucleotide on chromosome 12p. Nat. Genet. *6*, 14–18.
42. Kawaguchi, Y., Okamoto, T., Taniwaki, M., Aizawa, M., Inoue, M., Katayama, S., Kawakami, H., Nakamura, S., Nishimura, M., Akiguchi, I., et al. (1994). CAG expansions in a novel gene for Machado-Joseph disease at chromosome 14q32.1. Nat. Genet. *8*, 221–228.
43. Lindblad, K., Savontaus, M.L., Stevanin, G., Holmberg, M., Digre, K., Zander, C., Ehrsson, H., David, G., Benomar, A., Nikoskelainen, E., et al. (1996). An expanded CAG repeat sequence in spinocerebellar ataxia type 7. Genome Res. *6*, 965–971.
44. Pulst, S.-M., Nechiporuk, A., Nechiporuk, T., Gispert, S., Chen, X.-N., Lopes-Cendes, I., Pearlman, S., Starkman, S., Orozco-Diaz, G., Lunkes, A., et al. (1996). Moderate expansion of a normally biallelic trinucleotide repeat in spinocerebellar ataxia type 2. Nat. Genet. *14*, 269–276.
45. Ishikawa, K., Tanaka, H., Saito, M., Ohkoshi, N., Fujita, T., Yoshizawa, K., Ikeuchi, T., Watanabe, M., Hayashi, A., Takiyama, Y., et al. (1997). Japanese families with autosomal dominant pure cerebellar ataxia map to chromosome 19p13.1-p13.2 and are strongly associated with mild CAG expansions in the spinocerebellar ataxia type 6 gene in chromosome 19p13.1. Am. J. Hum. Genet. *61*, 336–346.
46. Campuzano, V., Montermini, L., Moltò, M.D., Pianese, L., Cossée, M., Cavalcanti, F., Monros, E., Rodius, F., Duclos, F., Monticelli, A., et al. (1996). Friedreich's ataxia: autosomal recessive disease caused by an intronic GAA triplet repeat expansion. Science *271*, 1423–1427.
47. Holmes, S.E., O'Hearn, E.E., McInnis, M.G., Gorelick-Feldman, D.A., Kleiderlein, J.J., Callahan, C., Kwak, N.G., Ingersoll-Ashworth, R.G., Sherr, M., Sumner, A.J., et al. (1999). Expansion of a novel CAG trinucleotide repeat in the 5′ region of PPP2R2B is associated with SCA12. Nat. Genet. *23*, 391–392.
48. Koob, M.D., Moseley, M.L., Schut, L.J., Benzow, K.A., Bird, T.D., Day, J.W., and Ranum, L.P.W. (1999). An untranslated CTG expansion causes a novel form of spinocerebellar ataxia (SCA8). Nat. Genet. *21*, 379–384.
49. Nakamura, K., Jeong, S.Y., Uchihara, T., Anno, M., Nagashima, K., Nagashima, T., Ikeda, S., Tsuji, S., and Kanazawa, I. (2001). SCA17, a novel autosomal dominant cerebellar ataxia caused by an expanded polyglutamine in TATA-binding protein. Hum. Mol. Genet. *10*, 1441–1448.
50. Jacquemont, S., Hagerman, R.J., Leehey, M.A., Hall, D.A., Levine, R.A., Brunberg, J.A., Zhang, L., Jardini, T., Gane, L.W., Harris, S.W., et al. (2004). Penetrance of the fragile X-associated tremor/ataxia syndrome in a premutation carrier population. JAMA *291*, 460–469.
51. DeJesus-Hernandez, M., Mackenzie, I.R., Boeve, B.F., Boxer, A.L., Baker, M., Rutherford, N.J., Nicholson, A.M., Finch, N.A., Flynn, H., Adamson, J., et al. (2011). Expanded GGGGCC hexanucleotide repeat in noncoding region of C9ORF72 causes chromosome 9p-linked FTD and ALS. Neuron *72*, 245–256.
52. Renton, A.E., Majounie, E., Waite, A., Simón-Sánchez, J., Rollinson, S., Gibbs, J.R., Schymick, J.C., Laaksovirta, H., van Swieten, J.C., Myllykangas, L., et al.; ITALSGEN Consortium (2011). A hexanucleotide repeat expansion in C9ORF72 is the cause of chromosome 9p21-linked ALS-FTD. Neuron *72*, 257–268.
53. Mootha, V.V., Gong, X., Ku, H.-C., and Xing, C. (2014). Association and familial segregation of CTG18.1 trinucleotide repeat expansion of TCF4 gene in Fuchs' endothelial corneal dystrophy. Invest. Ophthalmol. Vis. Sci. *55*, 33–42.
54. Jin, P., Alisch, R.S., and Warren, S.T. (2004). RNA and microRNAs in fragile X mental retardation. Nat. Cell Biol. *6*, 1048–1053.
55. LaCroix, A.J., Stabley, D., Sahraoui, R., Adam, M.P., Mehaffey, M., Kernan, K., Myers, C.T., Fagerstrom, C., Anadiotis, G., Akkari, Y.M., et al.; University of Washington Center for Mendelian Genomics (2019). GGC Repeat Expansion and Exon 1 Methylation of XYLT1 Is a Common Pathogenic Variant in Baratela-Scott Syndrome. Am. J. Hum. Genet. *104*, 35–44.
56. Kong, H.E., Zhao, J., Xu, S., Jin, P., and Jin, Y. (2017). Fragile X-Associated Tremor/Ataxia Syndrome: From Molecular Pathogenesis to Development of Therapeutics. Front. Cell. Neurosci. *11*, 128.
58. Sone, J., Nakamura, T., Koike, H., Katsuno, M., Tanaka, F., Iwasaki, Y., Yoshida, M., and Sobue, G. (2017). Reply: Neuronal intranuclear (hyaline) inclusion disease and fragile X-associated tremor/ataxia syndrome: a morphological and molecular dilemma. Brain *140*, e52.
59. Glineburg, M.R., Todd, P.K., Charlet-Berguerand, N., and Sellier, C. (2018). Repeat-associated non-AUG (RAN) translation and other molecular mechanisms in Fragile X Tremor Ataxia Syndrome. Brain Res. *1693* (Pt A), 43–54.
60. Todd, P.K., Oh, S.Y., Krans, A., He, F., Sellier, C., Frazer, M., Renoux, A.J., Chen, K.C., Scaglione, K.M., Basrur, V., et al. (2013). CGG repeat-associated translation mediates neurodegeneration in fragile X tremor ataxia syndrome. Neuron *78*, 440–455.
61. Sellier, C., Buijsen, R.A.M., He, F., Natla, S., Jung, L., Tropel, P., Gaucherot, A., Jacobs, H., Meziane, H., Vincent, A., et al. (2017). Translation of Expanded CGG Repeats into FMRpolyG Is Pathogenic and May Contribute to Fragile X Tremor Ataxia Syndrome. Neuron *93*, 331–347.
62. Tassone, F., Iwahashi, C., and Hagerman, P.J. (2004). FMR1 RNA within the intranuclear inclusions of fragile X-associated tremor/ataxia syndrome (FXTAS). RNA Biol. *1*, 103–105.

The American Journal of Human Genetics, Volume *105*

Supplemental Data

Expansion of Human-Specific GGC Repeat in Neuronal Intranuclear Inclusion Disease-Related Disorders

Yun Tian, Jun-Ling Wang, Wen Huang, Sheng Zeng, Bin Jiao, Zhen Liu, Zhao Chen, Yujing Li, Ying Wang, Hao-Xuan Min, Xue-Jing Wang, Yong You, Ru-Xu Zhang, Xiao-Yu Chen, Fang Yi, Ya-Fang Zhou, Hong-Yu Long, Chao-Jun Zhou, Xuan Hou, Jun-Pu Wang, Bin Xie, Fan Liang, Zhuan-Yi Yang, Qi-Ying Sun, Emily G. Allen, Andrew Mark Shafik, Ha Eun Kong, Ji-Feng Guo, Xin-Xiang Yan, Zheng-Mao Hu, Kun Xia, Hong Jiang, Hong-Wei Xu, Ran-Hui Duan, Peng Jin, Bei-Sha Tang, and Lu Shen

Supplemental Note

Case Report

Case F1-IV: 6

A 47-year-old male presented with limb weakness for 14 years. His four brothers also presented with muscle weakness in their early 30s. Muscle weakness began in the distal portion of the lower limbs, then moved up to the throat muscles and face. He routinely felt numbness in his limbs. The neurological examination revealed miosis, muscle atrophy, bilateral hand resting tremor, postural tremor, intention tremor, ataxia and weakness of the four limbs. Mini-Mental State Examination (MMSE) score was 29. Nerve conduction studies showed slowed motor and sensory nerve conduction velocity. Eosinophilic intranuclear inclusions were found in his skin samples, and the diagnosis of NIID was made.

Case F6-II: 3

A 49-year-old male was admitted to the hospital because of progressive slow walking over the last 3 years. The man was diagnosed with Parkinson's disease in 2017, and showed good response with dopaminergic therapy. He also complained about sleep disorders, constipation, and urgency of urination. The neurological examination revealed muscle rigidity and bradykinesia of right upper and lower limb. Unified Parkinson's disease rating scale (UPDRS) motor score was 5. Memory, orientation, and calculation were normal. The Mini-Mental State Examination (MMSE) score was 30, and the Montreal cognitive assessment scale (MoCA) score was 25. Blood chemistry was normal. Further skin biopsy confirmed the diagnosis of NIID.

Case F9-II: 6

An 81-year-old male presented with progressive cognitive decline for 10 years, and was diagnosed with Alzheimer's disease in 2015. His medical history included hypertension, diabetes, and he had to wear a catheter at all times due to urine retention over the past 10 years. His siblings had also been diagnosed with Alzheimer's disease. He had experienced several loss of consciousness episodes that lasted a few minutes each. The neurological examination revealed cognitive impairment, miosis, bilateral hand resting tremor, muscle rigidity, and bradykinesia. His brain DWI images showed typical high signal in the corticomedullary junction, Fluid-attenuated inversion

recovery (FLAIR) images showed severe white matter hyperintensity. CSF examination was normal, including the protein level and cytology. Electroencephalogram showed an increase in slow waves and no epileptiform discharges. He further received a skin biopsy which confirmed the diagnosis of NIID.

Case SP1

A 69-year-old male with left hemiplegia was admitted to the hospital. There was no medical history or family history for him. The neurological examination revealed complete recovery from the left hemiplegia. MMSE score was 27. No typical high signal in acute cerebral infarction was observed in DWI, while symmetrical hyperintense linear lesions was presented in the corticomedullary junction. Nerve conduction studies showed slowed motor and sensory nerve conduction velocity. His skin samples indicated typical NIID pathology.

Figure S1. Summary of Reads Covering Expanded Repeat Region from Oxford Nanopore Platform.

ID	Reads	Insertion Size	Repeat Number	Repeat Pattern	Match Ratio (observed read/pure repeat)
F1-IV:7	read 1	666	222	GGT	77%
	read 2	645	215	GGT	74%
F1-IV:15	read 1	344	114	GGT	88%
	read 2	340	113	GGC	78%
	read 3	295	98	GGC	86%
	read 4	370	123	GGT/GGC	81%/78%
F2-II:3	read 1	561	187	GGC	74%
	read 2	568	189	GGC	71%
	read 3	625	208	GGC	73%
	read 4	628	209	GGC	83%
	read 5	635	211	GGC	76%
F4-II:2	read 1	322	107	GGT/GGC	80%
	read 2	327	109	GGT/GGC	79%/77%
	read 3	384	128	GGC/AGC	87%/83%
	read 4	410	136	GGC	76%
	read 5	357	119	GGC	83%
	read 6	342	114	GGC	84%
F5-II:1	read 1	342	114	GGC	84%
	read 2	263	87	GGC	84%
	read 3	248	82	GGC	87%
F5-II:4	read 1	311	103	GGT/GGC	86%/84%
	read 2	348	116	GGT	88%
F9-II:6	read 1	345	115	GGC	78%
	read 2	396	132	GGC	85%
	read 3	403	134	GGC	78%
	read 4	449	149	AGC	81%
	read 5	356	118	GGC	82%
	read 6	385	128	GGC	77%
	read 7	343	114	GGT/GGC	77%/84%

Raw sequence data of expanded repeat region from Oxford Nanopore platform.

F1-IV:7-read 1

ACCATAAGTGTTTAGAAGTTTGAATTTTATCCTAGGGAGCTAGGGAGAGGTTCT
ATGGGTAGATTTCCTCATCCCAACAGATTGTAAAACTCCCACATCCAATTAGTC
ACACCTCCATTTTTTTTCTGTTTCTTTAACATTGTCTCAGATTCATTCCTTCTGA
AATCTACCTTATTAGTTCAGGTCTTCTTAAATTTGTGCTCTGGACTGTTCACTCA
ACTCTTTGACTGGTCCTGGGCTCCAGTTCACCTCTTGCTCTAAATAATCAATTT
TCTACAGAACTACTGAGCAATCTCCTTCCATCAATCTCAACTCTGTTCAAAAAA
CTTGTTGTGTTTTATAGACCCAAGAGGTAGCTGCTCCCACCCTTTCAGCTTGCT
AGTCTGGCTGTACACTGTCCCAACCCCTTTTCCTATTCTTGACTCCTCCTCTCA
CTGCTAACAGAGACAGAACTCATTTACTTACTCTTTGACTGTAATTTTCGAGCC
ACGAGGGCTCTTTCCGAATGATATGTCCATACAACTATTGTTGTTGTTCTTACTT
TTAGCCTCTTTTTCTTAGCCCACTTGTACCCAGGTTTGACTACACCCCTACTCTC
CTGCCCTGAAGTAACTAATATTCTGGTTATAGTTCTTAATATCTATATCTATACAT
TCTATATATATGCATCCACAGATAATGTAGTATTGTCTTTAAAAAAATATACATGT
ACCGTATACTTTTGTCTCCTCTCTGCATTAGCTTTTTTTCACTATCATTTATTTTT
AAGATTTATCCCTATTGGACATAAATAGCTATAGTCTTTTCAATTACTGTGTTGC
ATTTTACCTTATAAATATGCTCATACATTGTCTATCCATTCCTCTAAGGTGTGGCA
TTTATGTTGTTTTCAAGGTTTTGCTATTACAGTGTTAGATAAATATCCTTAGCTTA
GCTGGTCTCCTTGTCTTATACAAATTTTACGACCTTTCAAGATCCTGCTTTCATC
CCAGCCTCTGGGAAAGCTCTTCCTGAACTTTCAGCCTTGGCTGTGCCAGCAAA
GTCTGGCATCAAAGTTGCTTGCATCTTTGGTATCATCCCACTGCTAAAGCACAA
CAGCTACTAAAGTAAGGCACTTTAAGAAATATCTGCCACACGGATGAATGTGA
ATGAATGAATGGGGCAGTACCACTCGTGATGGCTCTATTCCTTTTAATGGACTA
TGCCTGGGCCTACTTCCTTCTTTGGATGTTAATCATCCACTCCAGAGCATTCTCT
AGCTAGCAAAGCAGTGTGGATGTGGGTGTAGGTGGCGATGCCAGACTCACTG
GATGAGGACCGCCTCCCTGCAAGTCACGATCCCGGCAACTCCAGTGCTTGAA
GTTCAGAGTCCTACCCCACCCCCCTCACCTCCACGCCCTTCTGCACTGGTCGT
TTTTGAAGGAGCCGCTTGGCCCTGATCGAGTTAAGGCTGCTGGAGAAGGATGC
GGACGGGCCAGTGACTCGTAGTAGATCCTCCGCGCGAGCTCGGGTCGGCGCT

TCTTCCTGCGGGAAACCCTGGTGCCCAAGGTGGCGGGGGCTGAGGCTGCGGC
GACAGTGGGTAACAGGTGGGAGGAGTGGCTGAGGGAAGGATTGGTCGAGGA
TGCTTCTGCTGCACACCTGAGAAAGTTTCACAAAACTTCGGGCGGCGGCTGG
AGGCGGCGGCCGAGGAGCGCGGACCTGGGGCGCGGGGAGTCGAGGCATTTG
CGCTTCTGTAAGCTGACTGTGTTAGGGCTTGAGTTTTTGAAGCAGGAGGAGGG
GAGGAGAGAGGGGTTTCACGGGGACCTTCCCATGTGGATTCAAGCCCAGGTG
GCGGTGGCGGGGCAGTGGCGGTGGTGGCGGTGGTGGTGGCGGTGGCGGTGG
CGGTGGTGGTGGTGGTGGTGGTGGTGGTGGTGGTGGTGGTGGGTGGTGGTGG
TGTGGGTGGTGGCGGGTGGTGGTGGCGGTGGTGGTGGCGGTGGTGGTGGCGG
TGGCGGTGGCGGCGGCAGGTGGTGGTGGCGGGTGGGTGGTGGTGGTGGTGG
GTGGTGGTGGTGGTGGTGGTGGTGGTGGTGGTGGTGGAGGTGGTGGGTGAGG
GAGAGGTGGTGGTGGAGGGAGGTGGGTGGTGGAGGGAGGTGGTGGTGGAGG
AGTGTGGTGGTGGAGGAGGTGGGTGGTGGAGGGAGGTGGTGGTGGAGGGAG
GTGGTGGTGGAGGGAGGTGGTGGTGGAGGAGGTGGTGGGTGAGGAGGTGGG
TGGTGGGAGGGAGGTGGTGGTGGAGGAGGTGGTGGTGGTGAGGTGGTGGTG
AGAGGGAGGTGGTGGTGGAGGGAGGTGGTGGTGGAGGAGGTGGTGGTGGAG
GAGGTGGCGGTGGAGGAGGTGGTGGTGAGGGAGGTGGTGGTGGAGGAGGTG
GTGGTGGAGGAGGTGGTGGTGGAGAGGTGGGTGGTGGAGGAGGGTGGTGGC
GGGAGGAGGTGGCGGCGGAGGAGGCGGCGGCGGGAGGAGGCGGCGGC

F1-IV:7-read 2

ATTTTGACTACGTTTATTTTTGAAAAGGGTTTCCAGCATTTCAATGTGACTATCA
CTAATGATACTTGCTTTTGCCTAAAGTGCTTGTTAATATGCAGTTTTGGAATTCT
GGCAGGTCTATGCCCATACTGACTCTTCAATTTCTGCTTAGTGTTGTTTACATCT
CTAGTTCAACACCTATTATGTGATAGACACTGGGATTAATAGGCTGTGGTCTTT
GCCCATGAGGACCCTCAAAACTTAGTTGAGAACACAGGAAATACAAAGAGTC
CTGTGTGAAAAATAAGCATTGGTGTTAAGATAAAGAAGCGGGGCAGTGGAAG
ACAGTGGAGGGATCTCTGTCTGTTAGAGGGAGGAATTTATTGCAGTGTCGGCA
AAGCCTTCTTTGGACTGAAGGATGAATTATGATTTAAAGTTCATTAAGAGGGA
GGAGGGAAGGGACTACAGGAGTCATTCCAAGCCAAAGGAACAAAAGATACA
GGGACAGAAACAGCAAGATATGTTTGGGGAATCTCTGGGTTAGCTAATACTGT

TATTGTGTAAGTTCACAACAGGGGCAGGGAGGACAGGAGATGGGGCCCAATG
ACTAGCCTTTGTATGTGCCCATGTTTAGGTTTGAATTTATCCTATAGGAGTCAGG
AGAGGTTCTATGGGTAGGATTCCCATCCCAACAGGATTGTAAACTCCTGGCAC
ATCCAATTAGTTTACACCTCCATTTTTTTTCTCATTTCTTTAACATTGTCTCAGA
TTCATCTTCTGAAATTCACCTTATTAGTTCAGGTCTTTAAATTTGTGCCTGGACT
GTTCACCAACTCGATCGGTCCTGGGGCTCCAGTTCACCTTGCCCCAACAATCA
ATTTTTCTACAGAACTATCAGGGACAATCTTCCTTTCCATCAATCTCAACTCTGT
TCAAAAACTTTCAATAGTTCTTATGTGACCTAAGAGGTAGGCTGCCTCCCACCC
TTTCAGCTTGGGCTATCTGTTGCCGTATAATTTGTCCCAAATTTCTTTCCGGTTC
TTGACTCCTCTTCTCACTGCTAACAGAGACAGAACTATTTACTTACTCTTTGAC
TGTACCTGCTTTCACTTAGGCTCTTCCTGAATGATATGTCCATACAACTATTGTT
GTTGTTCCATTTTTAGCTCTTTTCTTAGCCCACTGTACCCAGGTTTGATCACACC
CCCTACTCTGCCCTGAAGTAACCACATTCTGGTTATAGTCTTTAATATCTATATTT
ATCTATATATGCATCCACATGGCTAATGTGTAGTATTGTCTTGAAAAAATATACAT
AAATGGTATAATTTTTGTCTCCCTTACATTAGTTTTCACTATCATTTAGTTTTAAG
ATTTATCCTATTGACATAAATAGCTATAGTCTTTTCAATTACTGTGTTGCATTTCA
TTTTATAAATATACCACATTGTCTATCCATTCCTCTAAGGGTGGGTATTTATGTTG
TTTTTCAAGGTTTTACATGGTGTTACAATCTAATATCCTTAGCTTAGCTGGTCTC
CTTGTCTTATGCTACAAATTTTACTGACCTTTCAAGATCCTGCTTTTCATCCCAG
CTCCTTCGGGGAAAGCCTTCCTGAATCCCAGCCCACAGCGCAAAAGTCTTGGT
ACTAAAGCTGCTTGTGCATCTTGGTATCATCCTACTGCTACAATAACAGCTACT
TAGCAAGGCACTTAAGAAATATTCGCCACACGGATGAACAAATGAATGAATGA
ATGGGGCAGCACCACCCGTGATGGCTCTCTATTCCTTTTAATGGACTTTATGCC
TTGGCCCACTTCTTTCCTTCTTTGACAGCACTGGTCCTGTACTCCAGAGCATTC
CTCTAGCTAGCAAAGCAGTGTGTGGATGTGTGGGGTGTAGGTGGCGATACAGA
CCTCACTGGATGAGGGACCGCCTCTCCTCTGCAAGTTCACGATCCCGGCAACC
CCAGTGCTTCAGTTCAGAGTCCCACCTCACCCCTCACCTCCACGCCCCTTTCT
GCACTGGTCAAGCCAGAGCCGCTGCAGCCCCATCGAGTTAAGGCCATGGAGA
AGGATGCGACGGGGCCAGTGACCTGTAGATCCCTCCGCGCGGAGCCTGGGCT
GGCGCTTCTTCCTGCGGGAAACCCTGGTGCCCAAGGCGGCGGGGCCGAGCCG

CGGCGACAGGCGGGGCTTGCGGTGGAGGAGGCGGCTGAGGTGAAGGACACA
CGAGGCTGCTGCACACCTGAGAAAGCCAGCCAAACTTCGGGCGGCGGTCGGT
GGCGGCTGAGAGGGCGGACCGGGGCGCGGGGAGTCGAGGCATTTGCGCTTTC
CAAAGCCTGGACCGTAGCCAGGCCTGAGCTTGAAGCAGGAGAGGGAGAGAG
AGTGGCCTTTCTATCGGCCTTCCTATGTGGATTCGTCGGTGGTGGCGGCGGGTG
GCGGCGGTGGCGGTGGCGGCGGTGGCGGCGGTGGCGGTGGTGGCGGTGGTG
GTGGTGGTGGCGGTGGTGGTGGTGGTGGGTGGTGGTGGTGGCGTGGCAG
GCGTGGTGGTGGTGGTGGTGGGTGGTGGCGGCGGGTGGTGGTGGTGGCGGGT
GGCGGTGGTGGTGGTGGTGTGGTGGTGGCGGTGGTGTGGCGGCGGTGGAGGG
GCGGGTGGTGTGGAGGTGGGTGTGGTGGAGGAGGTGGTGGTGGAGGTGGTG
GTGGAGGAGGGTGGTGGAGGGGTGGTGGAGGGAGGTGGTGGTGGGAGGGGA
GGTGGTGGTGGGAGGGAGGTGGGTGGTGGAGGAGGTGGTGGTGGAGGAGGT
GGTGGTGGAGAGGTGGTGGTGGAGGGAGGTGGTGGTGGAGGGAGGGGTGGT
GGTGGAGGAGGTGGCGGGTGGAGGGAGTGGTGGTGAGGGAGGTGGTGGTGG
AGGGAGGTGGCGGGTGGAGGAGGTGGTGGCGGGAGGAGGTGGCGGTGGAGG
AGGTGGCGGCGGGAGGGAGGTGGCGGGTGGTGAGGCGGTGGCCGGAGGAGG
TGGCGGCGGAGGAGGCGGCGGCGGAGGAGGGCGCGGCGGAGGAGGCGGCG
GCGGAGGAGGCGGC

<u>F1-IV:15-read 1</u>

TGATAGACACTGGGGATTAATAGGCTGTGGTCTTTGCTCCATGAGGACCCCTTA
AACTTAGTTGAGAACACAGAAATGCCAAAGAGTCCTGTGTGAAAAATAAGTAT
TGGTGTTAAGATGGAAGAAGTTTGGTGGAAGACAGTGGAGGGACTGTCTGTT
GGAGGGAGGAATTTATTGTGTCGGCAAAGCCTTCTTTTGGACTGAAGGATGAA
TATGATTAGAAGTTCATTAAGAGGAGGAGGGAGGGGACTTACAGACATTCAAG
CCAAAGGAACAAAAGATACAGGACAGAAACAGCGCAAGATATGTTTGGGGGA
ATCTCGTTAGCCAATACTGTTACGTGAAGTTCACAACAGGGTAGGAGGACAGG
AGATGGGGCCCAATGACGCAAAAAAGGCCTTGTATGTACCATGTTTGAAGTTT
GAATTTATCCATAGAGTCAGGGGAGAGACTACAGTAGGATTTCCATCCCAATGT
AGGATTGAAACCCCACATCCAGTCACACCTCCATTTTTTTTCTTTCTGCTTTTT
AACATTGTCTCAGATTCATTCTCCTTCTGGAAATCTACTCATAGTTCAGGTCTTC

TTAAATTTGTGCCTGACTGTTCACCAACTTGACTGGTCCTTAGCTCCAGTTCAC
CTCTTGCTCCCCAACAATCAATTTTCACAGAACTATCAGAGCAATCTTCCCTTT
CCATCAATCAACTCTGTTCAAAAACTTTCAATAGTTTTATGACCTAAGAGGCTT
CCTCACCCTTTCAGCTTCTGCCATTCAAGGCCGTGTACTTTGTCCCAACCCCTT
CCGTCCTTGGACTCCTCTCACTGCTAATGAGATACAGAACTCATTTACTTACTC
TTTGACTGTACCTTTGCTTTCACGAGGGCTCTTTCCTGGAATGATATGTCCATAC
ACAACTATTGTTGTTGTTCTTAGCTTTCAGTCTTTTCTTAGCCTCACTGTACCCA
GGTTTGACCCACACCCCGCCTCCTGCCCTGAAGTAATTACTTATTCCAAGTTAT
AGTCTCTAATATCTATATTCATATTCATATATATAACCAAATAGAGTACGTAGTATT
GTCTTGAAAAAATATACATAAATGGTATACTTTGTCTCCCTCAAGCATTAGTTTT
TTCACTATCATTTAGTTTTTAAGATTTATCTTATTGAGGCTAAATAGCTATAGTCT
TTTCACTCATTGTGTGTTGTGTATTTCATTTTATAAATATACCATGTTAATATTCAT
CCATTCCTCTAAGGGTGGGTAGTACTTGTTACTTTTCAAGGTTTTGCTATTACA
GTAACCAAATATCCTTTAGCTTAGCTGGTCTCCTTGTCTTATACAAATTTTTTAC
TGACCTTTCAAGATCCTGCTTATCCCAGCTCCTCTGGGAAAGCCTTCCCTGAAT
CCCCAGCCCACAGCGGCAAAGTCTGGTACTCAAAGTTGCTTGTGCACTTTGGT
GTCATCCCACTGCTACACACAGCTACGCAAAGCAAGGCACTTACATGATGATA
GCCCAAATGAATGAATGAATGGGGCAGCACTACTCCGTGATGGCTCTATTCCTT
TTTAATGGACTTGGCGCCTTGGCCCACTTTCCTTCTTTGACAGCACTGGTCCTA
CCAGAGCACATTCTCTAGCTAGCAAAGGTGGATGTGTGGTGTAGGTGGCGATA
CAGACCTCACTGGATGAGAACGCCTCCCTGCAAGTTCACGATCCCGGCAACCT
GTGCTTGAAGTTCAGGGAGTCCTACCCCACCCCTCACCTCCACGCCCTTCTGC
ACTGGTCATTAAAGGAGCCGCTGCAGCCCTGATCGAGTTAAGGCTGCTGGAG
AAGGATGCGGACGGGCCAGTGACTGTAGTAGATCCCTCTGCGCGGAGCCTGG
CCGCGCTTTCTTCCTGCGGGAAACCCCAGGCTCAAGGCAGCTGAGGCTCGCG
GCGCGACAGGGCGGGGGCTTATGGGAGGAGGCGGCTGAGGCGGAAAGACAC
GAGGTTGCTTGCTGCACACTGAGAAAGTTTCAGCCAAATCTCATGGCGGGTTG
AGGCGGCGGCCGAGGAGCGGCGGGACTCTGGGGCGCGGGGGGAGTTGAGGG
CATTTGCGCTTGCGTTGGACCGTAGCGCCAGGGCTCTGAGCTTTGAAGCAGGG
GAGGGAGGGGAGAGAGGCCTTTCTTATCGGGACCCTTCCCATGTGGGATCTGC

CCAGGCGGCGGGCGGTGGCAGTGGGTGGTGGCGGGTGGTGGTGGTGGT
GGTGGTGGTGGTGGTGGTGGGTGGTGGTGGTGGTGGTGGTGGTGGTGGT
GGTGGTGGTGGTGGTGGTGGTGGTGGTGTGGTGGTGGTGGTGGTGGTGG
TGGTGGTGGGTGGTGGTGGGTGGTGGTGGTGGTGGTGGTGGTGGTGGTG
GTGGTGGTGGTGGTGGTGGTGTGGTGGTGGTGGTGGTGGTGGTGGTGGG
TGGTGAGGGAGGTGGTGGTGGAGAGGTGTGGTGGTGGTGGTGGGTGGTGGTG
GAGGGAGGTGGTGGTGGAGGGAGGTGGTGTGGAGGAGGC

F1-IV:15-read 2

TTAGTGAGTAAAGAGGAAGATCAGACTGGTAACACTGCTTGGTGAAACAACT
GAAAAATAATGTTATCTTTCTCTGAACATACTTACAAGACAAATTTAAAAATATT
TTGACTACGTTATTTTTTGAAAAAGGGTTTCCAGTATTTCAATGTGACTACTTTC
ACTAATGATGCCTTTTCAAAGTTAACATTAACATGCAGTTTTGGAATTTTCATAG
ATCTACCTTTATATACTGACTTGTACTTGCTTAGTGTTGTTTTACATTCTAGTTCA
ACATGTTATGTGATGAACACTGAGGTTAATGGCTGTGGTCTTTGCCCATGAGGG
ACCTCAAAACTTAGTTGAGAACACAGAAATACAAAAGAGTCCTGTGTGAAAA
TAAGTATTGGTGTTAAGATAAAGAAGCTGACAGTGGAAGACAGTGGAGGGAC
TGTCTGTTAGAGGGAGAATTGTTGGGTGTCGGCAAAGCCCTTCTTTGGACAGA
GATGGGAGTGTGATTAGAAGTTCATTAAGAGGAGGAGGAAGGGACTACAGTC
ATTTCAAGCCAAAAGGAACAAAAGATACAGGGACAGAAACAGCAAAGATATG
TTTGGGGAATCTCAAGTTAATACTGTTGTTGTGTGGGAAGTTCACAACAGGGG
CAGGAGGAGGACAGGAGATGGGGCCCAATGACGAAAAGGCCTTGTATGTACC
ATGTTTGGGGGTTGAATTTTATCCTGGAGTCAGGGAGGTTCTGGGTGGGATTTC
CCATCCCAACAGGATTGTAAACTCCCACATCCAATTAGTCCCTCATTTTTTCTG
TTCCTTTAACGTGTCTCAGATTCATCCCTTCTAAATCTACCTTATTGAGTTTCAA
GGTCTTCTTAAATTTATTGCCTGGACTGTTCACCGCCTCTTGACTGGTCCTGGG
GCTCCAGTTCACCTCTTGCTCCCAACAATCAATTTTCTACAGAACTATCAGAGC
AATCTTCCTTTCCATCAATCTCAACTCTGTTCAAAACTTTCCAATAGTTTTTTAT
GCTTGAGCTAAGAGGTAGCTTCTCCCACCCTTTCAGCTTGCCATTCGAGGCTG
CATAATTTAGTCCCAACCCCTTTCGTCAGCAGCTCCTCTCCTGCTAACAGAGAC
CATTCATTACTTCTTTTGACTGTACCTTTGCTTTCCACGAGGGCTCTTTCTGGA

ATGATATGTCCATACAACTATTGTTGTTGTTCTTACTTTTAGCTCTTTTTCTGCTT
AGCCCACTTGTGCCCAGGTTCAGTCACCCCCCTCTCCCTGCCTGGAAGTAACT
ACTATTCTGGTTATAGTCTACTTAATATCTATATCTATATCTATATATATGCATCCAC
AGATAATGTGTGATATTGTCTTGAAAAAAATATACATAAATGGTATACTTTGTCT
CCTGCGATTAGTTTTTCACTATCTTTAGTTTTAAGATTTATCCCTATTGACATAAA
TAGCTATAGTCTTTTCAATTACTGTGTTGCATTTCATTTATAAATATACCACATAC
ATTATTCATCCATTCCTCTAAAGGTGGGCATTTATGTTGTTTTCAGGTTTTGCTAT
TACAATATTTGTGAATATCCTTAGCAGCTGGTCTCCCTTGTCTTATACAAATTTT
ACTGACCTTTCAAGATCCTGCTTTCATCAACTCCTCTGGGAAAGCCTTCCCTG
AATCCCCCAGCCCACAGCAAGTCTTGGTACATCAAAGTGCTTGTGCATCTTTG
GGTATCATCACTGCCAAGCACACAGCTCGCAAAGTAAGAGCATAGAAATATTC
GCTTCACGGATGAACAAATGAATGAATGAATGGGGCCTTCCCACTCCATCGTG
GCTTATTCCTTTTGCAATGGACTTTGGCGCACCTTGGCCCCACTTTCCTTCTTT
GACAGCACTGGTCCTACTCAGAGCATTCTCCTTTAGCAAAGCAGTGTGTGGAT
GTGTGGGTGTGGGTGGCGATACAGACCTCTTTGGATGAGGACCGCCTCTCCCC
TGCAAGTTCACGATCAGCAAACTCTCAGTGCTTGAAGTTCAGAGTCCTGCCCA
CCCCTCACCTCCACGCCCTTCTGCACTGGTCAAGCCCAGCGAGCCGCTGCAGC
CTGATCGAGTTAAGGCTGCTGGAGAAAGGGTGCGGACGGGGGCCAGAGAGA
GTGGCTCGTAATGGGTCCCTCCGCGCGGAGCTCCGGGCCGGCGCTTCTTCCTG
CTGAAACCCTGGGTGCTGCCCAAGGCGGCAGGGGCCGAGGCCGCGGCGACT
GTGGAGGGCAGGGGCTTGCGGTGGGAGGAGGCGGCTGAGGCGGAAGGACAC
ACGAGGCTGCTTCCTTTGCACACCGAAAGTTTCAAGCCAAACTGGGCGGCGG
CTGGGGCGGCAGCCGAGGGCGGCAGCTCGGGGGCGCGGGGAGTCAGGCATT
GCGCCTGTGCTTCGGACCGTAGCGCCAGGCCTGAGCCTTTGAAAGCATGGGA
GGAGGGAGGAGGTGGGAATCCTCTATCAGGACCCCCTCCCATGTGGATCTGCC
CAGGCAAAGGCAAAGGCGGCGGCGGCGGCGGCGGCGGCGGCAGCGGCGGC
GGCGGCGGCGGCAACTGGCGGCTGGCAACAACGGCAACAGCGGCAGCGGCG
GCAGCGGCGGCGGCGGCGGCGGCGGCGGCGGCGGCGGCGGCGGCGGCGGCG
GCGGCACGGCGACGCTGGCAACGGCACGGCTGGCTGGCAACGGCGGCGGCA
GCAGCACGGCTGGCGGCGGCGGCGGCGGCGGCGGCGGCGGCGGCGGCAAGG

AGGAGGCGGCAGCGGAGGCAGCGGAGGAGGCGGCAGCGGAGGAGGCGGCA
GCGGAGGGCAGCAGCGGAGGGGAGGCAGCGGCGGAGGAGGCGGC

F1-IV:15-read 3

CTGGGATTATGACGCAAAGCGCCCGCCTGGCCTACATTTATTTAATAATGTATGC
TTCTAGTCGCTTCTAGGTTGCTCTCTTTGGGGATAACTATCTTATATTCTTCTACA
GATATGTTTTAATAGTGACAAAACTGCTTTACGGTGATTTTAAGAAAATTTGTAT
GCAGCCAGTACTATATGAATTATCTCTGGTGAGTAAAGAGGAAGATCGAACTG
GTAACACTGTGGGTAACAACTGAAAAATGTTATCTTTCTCAAGAACATACTTG
CTGAACAAATTCAAAAAATATTTTGACTCGTTATTTTTGAAAAAGGGTTTCTGA
CAATATATTTCAAATGCGACTACTTTCACTAATGATGCCCCTTTTAAAGTGCTTG
GTAACATGCGGTTTTGGAATTTCCTTAGGTCTATGCCCTTGACTTGTGTACTTG
CTTTTGATGTTGTTTACATTCTAGTTCAACATCTATTATGTGATAGACACTGGGA
TTAATAGGCTGGCGATTCTTTTACCCATGAGGACCTCAAACTTGATTGAGAACA
CAGAAATACAAAAGGAATCCTGGCCGAAAATAAGTATTATTGTTGGAATAAAG
CGGCAATGGAAGACAGTGGAGGGACTGTCTGTTAGAGGAATTATTGTGGTGTC
GGCAAGCCTTCTTTGATGAGGATAATTCGTTTAGAAGTTCTGTTAAGGGGAGG
AGGGAAGGGGACAAGGTCATTGAAGCCAAAGGAATAAAAGATACAGGGACA
GAAACAACAGCAAGATATGTTTAGGGGGAATCTCAGTGTAATACTGTTATTGTG
TGAAGTTCACAACAGGGGCAGGGAGATGGGGCCTTAATGTGAAAAGGCCTTG
TATGTACCATGTTTAAGTTTGAATTTATCTAGGGGAGTCAGGAGAGGAGTTCTA
TGGTGGATTTCCCATCCAACAGGATTAGCAAACTCCCCACATCCAATTAGTCCA
CCTCCTTTTTTTCTGTTTCTTTAACATTGTCTCAGATTCATTCCTTCTGAAATCTA
CCTTATTAGTTCAGGTCTTCTTAAATTTGTACTGGACTGTTCTGTACTCTTGGCT
TTGGTCCTGGGGCTCCAGTTCACCTCTTGCTCCCAACAATCAGTACAGAGACT
ATCAGAGCATCTTCCTTTCCGTAATCTCAACTCTGTTTTCAAAACTTTCCAATA
GTTTATGTGACCTAAGAGGTAGCTGCCTCCCCACCCTTTCAACGCATTCAAGG
CTGCATAATTTAGTCCCAACCCCTTTCAGGTCCTTGACTCACTGTAACAGAGAC
AGAACTCCCATTTGCCTCTTTTGACTGCTTTCTTTGCTTTCGAGGGGCTCTTTC
CTGGAATGATATGTCCATACAAGCTACTGCTGTTGTTCTTACTTTTAGCTCTTTC
TTAGCCCACTGCCAGGTTTGATCGTCACTACTCACCCTGGAAGTAGCCTATTAT

TCCTGGGTTATAGTCTCTTAGCCTTCTATATCTATATCTATATATATATGCATCCCC
AGATAATGTATTGTCTTGAAAAATATACATAAATGGTATACTTTGTCTCCCTCTAT
AAGTTAGTTTTTCCACTGTCATTTAGTTTTAAGATTTATCCTATTGACATAAATA
GCTATAGTCTTTTCAATTACTGTGTTGCATTTCATTTTATAAATATACATGCAGTA
TTCATCCATTCCTCTAGGGTGCTGAGGCGTATGTATTTGTTTTCAAGGGTTTTGC
TGTACAGTGTTAGCGAATATCCTTAACTAGCTAGTCCTCCTTGTCTTATACAAAT
TTGCAACTTTCAAGATCTGCTTTCATCAACGCTCTATGGAAAGCCTTCCCTGAA
TCCCCAGCCCGGCGGCAAGTCTTGGTACTCAAAGTTGCTTATGCATCTTTGGTA
TCATCCACTACCAAGCACACAGCTGCGCGAAATGTAAAATATTCGCTACGGAT
GAATTGAAATGAATGAATGAATGGGGCAGCACCGCCGTGATGGCTCTATTATTC
CTTTTTAATGGACTTGCGTACTGTCTTTGACAGCTTCTGGTCCTACTCAGAGCA
TTCCTCTAGCTAACAAAGCAGTGTGTGTGGATGTGTGTGTGCCGTTAGCGGGT
GGCGTCTGAACCTCACTGGACCGCCTCCTGCAGTTCGATCCCGGCAAACTCCA
GTGGCAGAGTTCGAGTCCTACCCCCCTCACCTCCACGCCCCTTCTGCACTGGT
CAGGCAGCGAGCCGCTGCAGCCCTGATCCAGTTAGGCTGCCTGCTGGAAGGA
CGGACAGGCCAGTGACTCGTGATCCCTCGCGCGCGCGGAGCTCGGGCCCGGC
ATATTTCCTGCGGGAAACCCCTGGGTGCCTGCCCAAGGCGGCGGCGGGGCCG
AGGCCGCAACATTGCCAGTAAGGCAGGGGCTTTGCGGTGGGAGGCGGCTGGC
CTTGGGTTACGAGGGCTGCTAAGCTGCACACGCGAAAGTTTCAGCAGGCGGC
GGCTGGAGGCGGCGACCGAGGGAGCGGCAGACTCAGGGGCGCAGGGGAGTC
GAGGGCATTTGCGCCTGTGCTGGACCATGGCGCGCAGGACTGGGCAACGCCA
GGAGGAGGAGAGTGGGGCTCTATCAGGTTATGGATCTGCCAGGCGGCGGCGC
GGCGGCGCGCTGACGGCGGCGGCGGCGGCGGCGGCGGCGGCGGCGGCGGCG
GCGGCGGCGGCGGCGGCGGCGGCGGCGGCGGCGGCGGCGGCGGCGGCGGCG
GCGGCGGCGGCGGCGGCGGCGGCGGCGGCAGCGGCGGCGGCAACGCGGCGG
CGGCGGCAGCGGCTGGCTGGCGGCAGCAACGGCGGCAGCGGCGGCGGCGGC
GGCGGAGGAGGCGGCGGCGGAGGGCGGCGGCGGAGGCGGCGGCGGCGGAG
GCGGCGGCGGAGGCGGCGGCAGGTGGCGGC

F1-IV:15-read 4

AGTTACGTATTGGTTATTTTATCCTATAGGAGTCAGAGAGGTTCTATGGGTAGG

ATTTCCCATCCCAACAGGATTGTAAACTCCACATCCAATTAGTCACACCTCCAT
TTTTTTTTTTCTGTTTCTTTAACATTGTCTCAGATTCATTCCTTCTGAAATCTACC
TTATTAGTTCAGGTCTTTCAATTTGTGCCTGGACTGTTCACCAATTTGACTGGT
CCTGGGGCTCCAGTTCACCTCTGCTCCAACAATCAATTTTCTACAGAACCATCA
GAGCAATCTTCCTTTCCATCAATCTCAACTCTGTCTAAAAACTTTCAATAGTTT
TATGTGACCTAAGAGGTAGAAGCTGCCTCCCACCCTTTCAGCTTGCCATTCAA
GGCTGCACACTCTGTCCCAACTCTTTCCATTCCTTGACCTCTCACTGCCACCAG
AGACAGAACTCCATTTACTTACTCTTTTGACTGTACCTTTTGCTTTCACGGTGG
CTCTTCGAATGATATATTCATACAACTATTATGCTGTTGTTCTTACTTTTACTCTT
TTCAGCCTCCTAATTTGTACCTATAGGTTTGATCACAATTTTACCTCCTGCCCTA
GAAGTAACTAATGTTCTGCTATAGTCTCTTAACATTCTATATCTATATCTATATATG
CTTAATTCACAGATAATGGTATTGTCTTGAAAAAATATACATAAATTTGTATACT
TTTTAGTTCCCTCAAGGACATTAGTTTTTTTCATCATCATTTAGTTTTTAAGATT
TATCCTTATTGACATAAATAGCTATAGTCTTTTTCAATTACGTTGCATTTCATTTC
CGAGTAAATATACTCCACATTATTCATCCATTCCTCTATGTGGCATTTATGTTGTT
TTCAAGTGTTTTTGCTATTACAGTGTTACAAACGAATATCCTTAGCTTAGCTGGT
CTCTTGTTCTATACAAATTTTACTGACCTTTCAAGATCCTGCTTTCATCCCAGCT
CCTCTGGGAAAGCTTTCCCTGAATCCCCCAGCCCACAGCGCAAAGTTCACTCA
AAGTTGCTTGTGCAATCTTTGCATCATCCCACTGCTAAGCACACAGCCACGCC
AGCAAGGCACTTAAGAAATATCGCCAATGGATGAAATCAAAAATGAATGAATG
AATGGGCAGCACCACCCGTGATGGCTCTATTCCTTTTTAATGGACTTTCATGTT
TTGGGCTCACTCTTCTCTCTTGACAGCACTGGTCCTACTCCAGCATTCCTCTAG
CTAGCAAAGCAGTGTGTGATGTGTGGGTGTAGGTGGCGACAAGAGACCACTG
GATGAGAATCGCCTCCTCCTGCAAGTTCACGATCCCGCAACTCCAGTGCTTAG
TTCAGGAGTCCTACCCCACCCCTCACCTCCACGCCCTTCTGCACTGGTCAGCT
AGCGAGCCGCTGCAGCCCTGATCGAGTTAAGGCTGCTGGAGAAGGATGCGGA
TTTGTGCCGCATTCCGTAGACCTCCGCGGAGCTCGGGCCGGCGCTTCTTCCTG
CGGGAAACCCCTGCGCTCCAAGGCGGCGTGGGGGGCTGAGGCTGCGGCGAC
AGTGGGGCGGGGGCCGGTGGTGGGAGGAGGCGGCTGAGGCGGGAAGGGATA
CACGAGGTTGAAAGCTTGCTGCACACCTGAGAAAGTTTAGCTAAATCTGGCG

GTGGCTGAGGTGGCGCGAGGAGCGGCGGACGGGGCGCGGGGAGTCGAGGTG
ATGGTGCCAAAGCTGACTGGGGCGCTGTGCCTGGAGCTTGAAGGCAGAGGAG
AGAGAGAAAGCGGGTTTATCGGGACTTTCTATGGGATTTGCCGCAGGGGTGTG
GTGGTGGTGGTGTGGTGGTGGTGGTGGTGGCGGTGGTGGTGGCGGTGGTGGT
GGTGGCGGTGGTGGTGGTGGTGGTGGCGGGTGGTGGTGGTGGTGGTGGTGTG
CGGTGGTGGTGGTGGCGGCGGTGGTGGTGGCGGTGGTGGCGGTGGCGTGGTG
GCGGTGGTGGTGCGGTGGCGGTGGTGGTGGTGGTGGTGGTGGTGGTGGTGGT
GGTGGTGGCGGTGGCGGGTGGCGGTGGCGGTGGCGGTGGCGGTGGCGGCGG
CGGCGGTGGAGGAGGCGGCGGTGGAGGAGGTGGTGGCGGAGGAGGCGGTGG
AGGAGGCGGCGGCGGAGGAGGCGGCGGCGGTGAGGCGGCGGCGGAGGAGG
CGGC

F2-II:3-read 1

CAGTGATTTTTAAAATTGTATGCAGCCAAGTCACCCTCGTTATCTTTAGTGAGT
AAGGAAGATCGAACTGAAGTAGCTGCTGTGGGTAACTGAAGAAGCAATGTTA
TCTTTCTCTGGTATACTTGCACAGACAAATTCAAAAATATTTGACTACGTTATTT
TGAAAAGGGTTTCCAGTATTTCAATGTGACTACTTTCACTAATGATACTTGCTT
ACCTAAAGTGCTTGTTAACATGCAGTTTTGGAATTTCATGATCTGGCTTACATA
CAACATACTTGCTTAGTGTTGTTTTTACATTCTAGTTCAACATCTATTATGTGATA
GACACTGGGGATTAATAGGCTGTGGTCTTTGCCCATGAGGGACCTCAAAACTT
AGTTGAGAACACAGAAATACAAAGAGTCCTGTGTGAAAATAAGTAGTGGTGT
TAAGATAAAGAAAGCGGCAGTGGAGACGGTGGAGGGACTGTCTGTTGGAGG
AGGAATTTATTGTGGTGTGGCCAAGCCTTCTTTGGACTGAGGATGAATTATGAT
TAGAAGTTCATTAAGAGGAGGAGGGAAGGGGACTACAGAGTCATTCCAAGCC
AAGGAACAAAGATACAGGGACAGAAACAGCAGATATGTTTGGGAGATCTCAA
GTTAGCCAATACTGTTATTGTGTGAGATTCACAACAGGGGCAGGGAGGACAGG
AGATGGGGCCCAATGGCGAAAAGGCCTTGTATGTACCATGTTTAGGAACAGAT
TACATCCTATGAGGTCAGGGAGAGGTTCTATGGGTAGGATTTCCCATCCCAACA
GTTGTAAACTCCCATCCAATTAGTCACCTCCCATTTTTTCTGTTTCTTTTTAACA
TCATAATTCATTCCTTCAAATCTACCTTGGCTGATTCAGGTCTTCCAAATTTGTT
ACTGGACTGTTCACCAACTCTTGACTGGTCCTGGGGCTCCAGTTCACCTCTTG

CTCCCAACAATCAATTTTCTGCAAGAACTATCAGAGCAATCTTCTTTCCATCAA
TCTCAACTCTGTTCAAAACTTTCAATAGTTTTATGTGACCTAAAGAGTGGCACA
CGCCCACCCTTTCAGCTTGCCATTCAAGGCTGCATAATTTGGTCCCAACCCTTT
CCGTCCTTGACTCCTCACTGCTAACGAGACCAGAACTCATTTACTTACTCTTTT
GACTGTGCCTTTAGCTTTCGAGGCTCTTTCCTGGATGATGTATCCATACAACTAT
TGTTGTTGTTCTTACCAGCTCTTTTCTTAGCCCCGCCTTTTAGGTTGGTCTTTTC
TCCTGCCTGGAAGTAACTACTATTCTGGTTATAGTCTCTTAATATCTATATCTATA
TCTATATATATGCATCCACAGATAATGTAGTATTGTCCTTGAAAAAAAAACATAA
ATGGTATGCTTTGTCTCCTCTGCGATTGGTTTTTTCCTATCATTGGTTTTAAGATT
TATCCTAATAGCGTAAATAACATAGTCTTTTTCAATTGCGTGATTTGCATTTCAA
AACTTGCAATACTACATATTATTCATCCATTCCTCTAAGGGTGGTATTTATGTTGT
TTTTCAAGGTTTTACTGTACAATGTTACAACGAATATCCTTAAGCAAGCTGGTC
TCCTTGTCTTATACAAATTTTGCAGCCTTTCAAGATCCTGCTTTCTGTTAGCTTC
TACAGGAAGCCTTCCTGAATCCCCAGCCCACAGCAGCAAAGTCTTGGCCTCA
GGTGCTTGTGCCTCTTTGGTATAATCCTGTAAACACACAGCTACATAAAGTAAG
GCACTTAAAATATTCCTTCCACGGATGAACAAATGAATGAATGAATGGGGCAG
CACCACTCCGTGATGGCTCTATTCCTTTTAATGGACTTTGGCGCCTTGGCCCAC
TTTCCTTCTTTGTGCTGGTCCTACTCAGAGCATTCCTCTAGCTAGCAAAGCAGT
GTGTGGATGGTGTAGGTGGCGATACAGACCTCACTGGATGAGGACCGCCTCTC
CCTGCAAGTTCACGATCGGCAACTCCAGTGCTTGAAGTTCAGGTCTACCCCAC
CCCTCACCTCCACCCCTTCTCTGCACTGGTCAAGCCAGCGAGCCGCTGCAGCC
TGATCGAGTTAAGGCTGCTGGAAAGGATGCGGACAGGGCCGGTAATCATGTCG
ATCCTCCGCATGGGCTGGGCCGGCGCTTCTTCCTGCAGAAACCCCTGGGTGCC
CAGGCGGCGGGGCCGAGGCCGCGGCGACAATGAGCAGGGCTTGCGGTGGGA
GGAGGCGGCTGAGGCGGAAGGACACACGAGGGGCTGCTTGCTGCACCCGAG
AAAGTTTCAGCCAAACTTCGGGCGGCGCAGGGCGGCGGCCGAGGGCGGCAC
GGACTCCGGGGCGCGGGGGTCAGGGCAGCTGCGCCTGTGCTTCGGACCGTAG
CGCCAGGGCCTGAGCACAAACGGGAGGGAGGAAGGTGGGGCTCCTCTATCAG
GACCCCACCTCCCATGTGGATCTGCCCAGGCGGCGGCGGCTTTGGCGGCAGC
GGCGGCGGCGGCGGCGGCGGCAGCGGCGGCGGCGGCGGCGGCGGCAGCGGC

AACGGCGGCGGCGGCAGCGGCACGGCAGCGGCGGCGGCGGCGGCGGCGGCG
GCGGCGGCAGCGGCGGCGGCGGCGGCGGCGGCGGCGGCGGCAGCGGCGGCG
GCGGCGGCAACGGCAGCAGCGGCGGCAACGGCAGAGAAGGCAACGGCG
GAGGAGGCGGCGGCGGAGGCGGCGGCGGAGGGCGGCGGCGGAGGCGGCTT
GGCGGAGGAGGCGGCAGCGGAGGAGGCGGCGGCGGAGGAGGCGCGGCGGC
GGGAGGCGGCGGCGGAGGGCGGCAGCGGAGGGAGGCGGCGGCGGAGGCCC
CGGCAGCGGAGAGGCTGGCGGCGGAGGAGGCGGCGGCGGAGGAGGCGGCG
GCGGAGGAGGCGGCGGCGGAGGGGCGGCAGCGGAGGAGGCAACGGCGGAG
GAGCGGCAGCGGAGGAGGGGCGGCGGCGGAGGAGGAGCGGCGGCGGAGGG
AGGCGGCGGCGGAGGCGGCGGCGGAGGAGGCGGC

<u>F2-II:3-read 2</u>

GTTAAGATAAAGCGGCAGTGGAAGACAGTGGAGGGACTGTCTGTTAGAGGAG
AATTTATTGTGGTGTCGGCCAAAGCCTTCCTTTGGACTGAAGGATGAATTATGA
TTAGAGTTCATTAAGAGGAGGAGGGACTGGAGGTGTTCAAGCAAAGGAACAA
AGATACAGGGACAGAAACAGCAAAGATATGTTTTGGGGGAATCTCAGTTAGCC
AATACTGTTGTGTGTGAAGTTCACAACAGGGGCAGGGAGGACAGAGATGGGG
CCCAATAGTGAAAAGAGCCTTGTATGTACCATGTTGAAGTTTGAATTTATCCTA
TAGGAGTCAGGAAGTTCTATGGGTAGGATTTCCCATCCCATGTGGGTTATAGCT
CCATCCAATTAATTCACCTCCATTTTTTCTGTTTCTTTAACATTCTCCAGATTCC
ATTCCTTCAAATCTACCTTATTAGTTCAGGTCTTCTTAAATTTGTGCCTGAGACT
GTTCACCCAACTCTTGACTGGTCCTGGGGCTCCAGTTCACCTCTTGCTCCCAA
CAATCCAATTTTCTACAGAACTATCAAGAGCAATCTTCCTTTCCATCGTCAACT
CTGTTCAAAAACTTTCCAATAGTTTTATGTGACCTAAGAGGTGTGCCTCCCCAC
CCTTTCAGCTTGCCATTCAAGGCTGCAATTTTAGTCCCAACCCCTTTCAGTCTT
GACTCCTCTCACTAACAGAGACAGAACTCATTTCACCTCTTTTGACTGTACCTT
TGCTTTCACGAGGCTCTTTCCTGGAATGATATGTCCATACAATATTTTGTTGTTC
TTACTTTTAGCTCTTTTAGCCCACTTATTGCGGACTTTGATCCACACCCCCTAC
TCTCCTGCCCTAGAGTAACTACTATTCTGGTTATAGTCTCTTTAATATCTATATCT
ATATCTATATGTAGCCATCCACAGATAATGTGTAGTATTATCTTGAAAAAAATATA
CATAAATGGTATACTTTGTCCCCTCTGCGATTAGTTTTCACTTATCATTTAGTTTT

AAATTTATCCCTATTGACATAAATAGCTATAGTCTTTTCAATTGCTGTGTTGCATT
TTATTTATAAATATACCATACATTATTCATCCATTCCTCTAAGGGTGAGCATTATAT
TTTCAAAGGTTTTTATATTACAGTGTTACAACGTTATCCTTAGCTTAGCTGGTCT
CCTTGTCTTACTGAATTTTACTGACCTTTCAAGAGTCTGCTTTCATCCCAGCTC
CTCTGGAAGCTTCCCTGAATCCCCCACAGCCTGACGGCAAAAGTCTTGGTACT
CAAAGTTGCTTGACCGTCTTTGTTGTCATCCACTGCCAAGCACACAGCTTTCAT
AAAGTAAGGCACTTAAAATATTCGCTACACGGATGAACAAATGAATGAATAGT
GAACTGCATTTTTCCTCCGTGATGACTCCTATTATTCCTTTTAATGGACTTTGGC
ATGTGGCCCCACTTTCCTTCTTGAGCACTGGTCCCTACTCCAGAGCATTTGTTC
CTCCTAGCTAGCAAAGCAGTGTGTGGATGTGTGTGTGGGGTGTAGGTGGCAAT
CCTGAACCTCACTGGATGAGGACCGCCTCTCCTGCAAGTTCACGATCTGGCAA
ACTCCAGTGCTTGAGTTCAGGTCTACCCCACCCCTCACCTCCACGCCCCTTCT
GCACAGGTCCAGCAGGCCGCTGCAGCCCCTGATCAGAGTTAAGGCTGCTGGA
GAAGGATGCGGACAGGGGCCAGTGACTCGTAGTAGATCCCTCCGCGCGGAGC
TCGGGCCGGCGCTTCTTCCTGCAGGAAGCCCCTGGGTGCCCAAGGCGGCGGG
GCCGAGGGCCACTGCAACGACGAGTGGGGCAGGGCTTGCGGTGGGAGGAGG
CGGCTGAGCGGAAGGACACACGAGGGCTGCTTCGCTGCACACCGAGAAAGT
TCCGGCTGGAAACTTGGGCGGCGGCTGGGCGGCGGCCAAGACGGCGGACTC
AGGGGCGCAGGGAGTCGAGGCATTTGCGCCTGTGCTTTGGACCCGCCGCGCC
AGGGCCTGAGCCTTTGAAGCAGGAGGAGGAGAGAGTGGGGCTCCTCTTCGGG
CCCCTCCCCATGTGATCTGCCCAGGCGGCGGCGGCTGGCAGCGGCAACTGGC
AGCGGCGGCGGCAGCGGCAGCAGCAACGGCAACGGCACGGCAGCAGCGGCG
GCGGCGGCGGCGGCAGCAGCAGCAGCAGCAGCAGCGGCAGCGGCACCGGCA
ACGGCAGCAACAACGGCAACAACAGCAACGGCAACGGCAGCGGCGGCAGCA
GCAACAGCAACGGCAGCAGCAGCAGCGGCAGCAGCAGCAGCGGGAGAGGC
AACGGCGGAGGAGCAACGTGGAGGAGGCGGCGGCGGGAGGAGGCAACAGC
GGAGGAGGCGGCGGCGGAGGGCGGCGGCGGAGGAGGCGGCGGCGGAGGAG
GCGGCGGCGGAGGTGGCGGCGGAGGAAAGCGGCGGCGGAGAGGCGGCGGA
GGGAGCCGGCGGCGGAGGAGGCGGCGGCGGAGGAACGGCAGCGGAGGAGG
CGGCGGCGGAGGAGGGCTGACGGCGGAGGGCTGGCAGCGGAGGAGGCGGC

GGCGGAGGAGGCGGCAGCGGAGGAGGCGGCAGCGGAGGAGGCGGCAGCGG
AGAGCAGCAGCGGAAGGAGGCGCGAGCGGAGGCGGC

F2-II:3-read 3

GTTATCTTTAGTAGTGAAGAGGAAGATCGAACTGTGGTAACACTGTGCCCAGG
TGAAACATGAAAAATAATATTATCTTTTCTCTAAGACATACTTTTGAGACAAAA
TTCCAAAAAATATTTTGACTACGTTGTTTTGAAAGGGTTCCAAGTATTTTCAAG
TGACTACTTTCCTATGATACTTGCCCACTAAAAAGTACTTAGCATTAACATGCA
GTTTTGGAATTTCCTTAGATTCTACCCATGCAAGCTTGCCTTGAGTGTTTGTTTT
CTGGTTCCTTTATCTATTATGTGAAGTGAACACTAAGTTATCGACTGCATGGTCT
TCCTTGTAGGGACCTCAAAAACTTAGTTGAACACAGAAATACAAAGAAGTCCT
GTGAAAAATAAGTAGCCAATTTTAAGATAAAAAAGCGGCAGTAGAAGACAGT
GGAAGGGACTATCTGTTAGAGGAGAATTTGTTGTGGTGTCATGCACTTCTTTG
GACTGAGGATGAATTATGATTAGAAATTCATTAAGAGGAGGAGGAAGGGGACT
ACAGGGGTCATTTCCAAGCCAAAGACAAAAGATGAGGGACAGAAGCAACAA
GACAGATATGTTTTGGGGGAATCTCAAGTTAATAATACTGTTGTGTGAAGTTCA
CAACAGAGCAGGAGGGACAGGAGATGGAGACTAATAAGCGAAAAAGGCCTT
GTATGTTACCATGTTTAAGTTTAGATTTTATCTATAGGAGTCAGGGAGGGAGTTC
TATGGGTGGGATTTCCATCCCAACAGGTTGTAAACTCCCACGTCAATTAGTCCA
CCTCCATTTTTTTCTGTTTTACTTTAACATTGTCTCAGATTTGTTCCAGAATCTAT
TGGTTCAGACTTTGCTTGGTTTGTGCTGGACTGTTCCACTAACCTCTTGACTGG
TCCTGGGGCTCCAGTTTACCTCTTGCTCCCAACAATCAATTTTTCTACAGAACT
ATGAGCATTCTTTGCTTTCCTCAATCTCAACTCTGTTCAAAACTTTCGGACAGA
TTTTATGTGACCTAAAGAGGTAGCTGCCTCCCACCCTTTCAACACCATTCAAGG
CTGCATAATTTGAATTCTTACTTTCAGTCACCAGCTCCTCTCACTGTAACAGAG
ACAGAACTCATTTACTTACTCTTTGGCCTTTACTTTCAGGGGCTACTCCTGGGA
ATGAAATATATATTTGACTGACGACTGTGTTCTGCACTAGCTCTGCCGCCCACC
CAGCCACCCAGGTTTGATCACACCCCCTCTCACCCCTGGAAGTAGCTTGCAAC
CTGGTTATAGTCTCTTAATATCTATCTATATCTCTCCTACTTGCATCACAGATGAC
ATATAAATGTGTCTTGAAAAAAATATACATAAATGGTATACTTTGGCTCTCCCTC
TAGATTAGTTTTCTATCATTTGGATTTTTTAAGATTTATCCCTATGACATAAATAA

GCTATAGTCTTTTCCAATTACTATATTTACATTCATTTATAAATATACATACATTAT
TCATCCATTCCTCTAAGGGTGCAGGCATTTATGTTACTTTCAGGTTTTGCTATTA
CAGTGTTACAAGATATCCTTAAGCAAGCTGGTCTCTCCTTGTCTTATACAAATTT
TACTGACATTTCAAGACCACACTTTCAGCCCGGCTCCTGGGGAAAGCCTTCCC
TAATCCCCAGCCCACAGCCAGCCAGTGCGACCAGGGTTGCTTGCCACATCTGG
TATCATCCCTACCAAGCACACAGCCTCTTGTAAAGTAAGGCACTTAAGAAATAT
TTCGCTTCACGGATGAACAAATGAATGAATGAATGGGGAATGGCTACTCCGTG
ATGGCTCTGTTCATACTTGTCTGCAGACTTTGGCGCCTTATTTTCTCCTTCTTTG
ACCTTTGGTCCTACTCAGAGCATTCCTCTAGCCCTAGCAAAGCAGTGTGTGGA
TGTGTGGGTGTGGGTGGCAGTGAACCTCACTGGATGAGGACCGCCTCTCCCTG
CAAGTTCTACAGTCAGCAAACTCCAGTGCTTGAAGTTCAGAGTCTACCCCACC
CCCTCCACCTCCACGCCCCTTCTGCTGAGTCAACCCAACCCAGCCACTGCAGC
CTGATCAGTTAGGCTGCTGGAGAAGGATGCCAGACAGGGCCAGTGACTCATG
TCTAGATCCCTCCACCGACCAGGCCCACTTCTTCCTACAGGGAAGCTTCGATTT
TTAAAGCCATGGGGCCGAGGCCACCCGGCGACAGTGGGGGCAGGGCTTGCAG
TGGGAGGAGGCGGCTGAGGCGGAAGGACACACGAAGACTTCGCTGCACCGA
GAAAGTTCAGCCAAACTGGGCGGCAGCTAGAATAGCAGCCGGGGAACTGGCG
GACTCCGGGACGCAGGGGTCAGGCATTTTGCGCCTGTACCTTTGGACCATGCG
CCAGGGCCTGGCCTTTAGAAACCAGGAGGAGGGGGAGGGGAGGAAATCGGG
CTCCTATCGGGACCCCTCCCCCATGTGGATCTGCCCAGCAGGCGGCGGCGGCG
GCGGCGGCGGCGGCGGCGGCTTTGACGGCAGCAGCGGCAGCGGCGGCGGCG
GCGGCGGCTGACCCGGCAACGGCAACGGCAGCAGCGGCGGCGAAAAGCGGC
GGCGGCAACGGCAACACGACGGCGGCGGCGGCGGCGGCAGCGGCGGCGGCG
GCGGCGGCGGCAACGGCAGCAGCGGCAACGGCGGCAGCAGCTGCTAACAAC
CCAACAGCAGCAACAGCGGCGGCGGCGAAGCAGCGGCAGCGGAGGAAGGG
GCGGCAACGGAGGGCGGCGGCGGAGGAGAGCGGCAGCAGAGGAAGCCTGG
CGGCGGAGGAGGGCGGCGGCGGAGAGCGGCTGTAGAGAGGCAGCAGCGGAG
GAGGCAGCAGCGGAGGAGAAACTGGCATGGCGGCGACGGAGGGCAGCTTGA
CGGAGGAGGCAGCGACGGAAGGCAGCAGCAGAGGAGAGCAGCAGCAGGAG
GTAACGAGGAGGCGAAAGCGGCGGAAGGCGGCGGCGGAGGAGGCGGCGGC

AGAGGAGAGCGGCAGCGGAGGCGGCGGCGGAGGAAGGGACGGCAGCCGGA
GGAGGCGGCAGCGGAGGAATGGCAACGGCGGAGGAGGCGGCAGCAGAAGA
GGCGGC

F2-II:3-read 4

GTCACACCTCCATTTTTTTTTTTCTGTTTCTTTAACATTGTCTCAGATTCATTCCT
TCTGAAATCTACCTTATTAGTTCAGGGTCTTCTTAAATTTCCAGTTTGGACTGTT
CACTAACTCTGACTGGTCCTAGCTCCAGTTCACCCTCTTGCCCCAACAATCAAT
TTTCTACAGAACTATCAGAGCAATCTTCTTTCCCCATCAATCTCAACCTGTTCA
AAAACTTTCAATAGTTTTATGACCTAAGAGGTAGCTGCCTCCCCCACCCTTCAG
CTTGCCATTCAAGGCCGTATAATTTGGTCCCAACCCCTTTCCGTCATTCTTGAC
TCTCCTCACTGCTAACAGAGACAGAACTCATTTACTTACCTTTGGATCGTACCT
TTGCTTTCACGTAGGGCTCTTTCCCTGGAATGATATGTCCATACAACTATTGTTG
CTGTTGTTCTTACTTTTAGCCTTTCAGCCCACTTGTACCCAGGTTTGATCACAC
CCCTACCTCCTGCCTCCCAAAGCAATCACTATTCTGGTTATAGTCTCTTAATATC
TATATCTATATCTATATATATGCATCCACAGATAATGTGTAGTATTGTCTTGAAAA
ATATACACAAATGGTATACTTCTGTTCTCTCATGTATTAGTTTTTCACTATCATT
TAGTTTTATGATTTATCCTTATTGACATACAAATAGCTATAGTCTTTTTCAATTAC
TGTGTTGCATTTCATTTTATAAAGGAGTACTATACATTATTCATCCATTCTCTAAG
GGTGGGTATTTATGTTGTTTTCAAGGTTTTGCTATTACAGTGTTACAAATTTAAT
ATCCTTAGCTTAGCTGGTCTCCTGTCTTATGTTAAATTTTGCATTGACCTTTCAA
GATCCTGCTTTCATCCCAGCTCCTCTGGGAAAGCCTTCCCTGAATCCCCCAGCC
CACAGCGCAAAGTCTTTGGTACTAAAGTTGCTTGTGCATCTTTGGTATCATCCC
ACTGCTGCCAACACAGCCACGCAGTAAGGCACTTAAGAAATATTCGCCACACG
GATGAACAAATGAATGAATGAATGGGGTGCACCACCGTGATGGCTCTATTCCT
TTTTAATGGACTTATGCCTTGGCCCACTTCTTCTTTGACAGCACTGTCCTACCC
AGAGCATTCCTCTAGCTAGCAAAGCAGTGTGTGGATGTGTGGGTGTAGGTGGC
GATACAGACTCACTGGATATGAGGGACCGCCTCTCCCGGTAAGTTCACGATCC
CGGCAACCCAGTGCTTGAAGTCTAGAGTCCTATCTTCACCTCACCTCCACGCC
ACCTTCTGCACTGGCTGGGTTAGGTGAGCCGCTGCAGCCCTGATCGAGTTAAG
GCTGCTGGAGAAGGGATGCGGATTGCAGCCAGTGACTCGTAGTAGATCCCTCC

GCGAGCCTGGGCCGGCGCTTTCTTCCCGTGGGAACCCCTGGTGCCCAAGGCG
GCGGGGCTGAGGCCGCGGCGACAGTGTGTGGGCGGGCTTGCGGTGGAGGAG
GCGGCTGAGGCGGAAGGATACGAGGTTGCTTCGCTGCACACCTGAGAAAGTT
TCAGCCAAACTTCTGGGCGGCGGCTGAGGCGGCGGCTGAGGAGCGGCGTGG
ACTGGGGCGGGGAGTCGAGGCATTTGCGCCTGTGCTTCGGACCGTAGCGCCA
GGGCCTGGAGCCTTTGGGGCAGGAGGTGAGGGGAGGGAGAGGCCTCTATCGG
GACCCTCCCCATGTGGATCTGCCCAGGCGGCGGCGGCGGCGGCGGCGGC
GGCGGCGGCGGCGGCGGCGGCGGCGGCGGCGGTGGCGTGGCGGCGGCG
GCGGCGGCGGCGGCGGCGGCGGCGGCGGCGGCGGCGGCGCAAGGGGCA
AGGCGGCGGTGGCGGCGGTGGCGGCGGGCGGCGGCGGGTGGCGGCGGCGGC
GGCGGCGGTGGCGGCGGCGGCGGCGGCGGCGGCGGTGGCATGGCGGGTGGC
GGGGTGGCGGCGGCGGCGGTGGCGGCGGGTGGAGGAGGTGGCGGTGGAGGA
GGGGCGGCGGCGGGAGGGAGGTGGCGGTGGAGGAGGGTGGTGGTGGAGGA
GGTGGTGGCGAGGGAGGCGGTGGCGGAGGAGGTGGCGGTGGAGGAGGTGGG
TGGTGGAGGAGGGTGGTGGCGGAGGAGGCGGTGGCGGAGGAGGTGGCGGTG
GAGGGAGGTGGCGGTGGAGGAGGCGGCGGAGGAGGTGGCGGGCGGAGGAG
GTGGCGGTGGAGGAGGGGTGGCGGGTGAGGAGGCGGTGGTGGAGGAGGCGG
CGGCGGAGGAGGCGGCGGCGGAGGAGGCGGCGGGTGGAGGAGGCGGCGGC
GGAGGAGGTGGTGAAGTGAGGAGGCGGCGGCGGAGGAGGCGGC

<u>F2-II:3-read 5</u>

GGAAGGACTACAGGAGTCATTCCAAGCCAAAGGAACAAAAAGATACAGGGA
CAGAAACAGCAAGATATGTTTGGGGGAATCTCAAGTTAGCTAATACTGTTATTG
TGTGAAGTCTACAACAGGCAGGGAGGACAGAGATGGGCCCAATGACTAAAAG
GCCTTGTGGCTGTACCAGTTTAGAAGTTTGAATTTTATCCTATAGGAGTCAGGG
AGAGGTTCTATGGGTAGGATTTCCCATCCCAACAGGATTGTAAACTCCTCACAT
CCAATTAGTCACACTCATTTTTTTTTTTTTTCTGTTTCTTTAACATTGTCTCAGAT
TCATTCTTCTGAAATCTACCTTATTAGTTCAGGTCTTCAAATTTGTGCCTGACTG
TTCACCAACTCTTGACTGGTCCTGGGCTCCAGTTCACCTCTTGCTCCTAACAAT
CAATTTCTACAGAACTATCAGAGCAATCTTTCCTTTCCATCAATCTCAACTCTG
TTCAAAAAAATCTCACAGTTTTATGACCTAAGAGCAGTAGCTGCCTCCCACCC

TTTTCAGCTTGCCATTCATACGTATAATTTGTCCCAACCCCTTCATTCTTGACTC
TCCTCACTGCCAATAGAGACAGAACTCATTTACTTACTCTTTGACTGTACCTTT
GCTTTCACGAGGGCTCTTTCCGGAATGATATGTCCATACAACTATTGTTGTTGTT
CTTACTTTATTTTTCAGCCCACTTGTACCCAGGTTTGATCACACCCCTACTCTCC
TGCCCCCAAAGAATTGCTACTTATTCTGGTTATAGTCTCTTAATATCTATATCTAT
ATCTATATATATGCAATTCACAGATAATGTGTAGTATTGTCTTGAAAAATATACAC
AAATGGTATACTTTGTCTCCCTCTCATGTATTAGTTTTTCACTATCATTTAGTTTT
TAAGATTTATCCCTTGGCTGACATAAATAGCTATAGTCTTTTTCAATTACTGTGT
TGCATTTTCATTTTATAAATATACCACACAGGTCTATCCATTCCTCTAAGGGTGG
GCATTTATGTTGTTTTCAAGGTTTTACATACAGTGTTACAACGTAATATCCTTAG
CTTAGCTGGTCTCTGTCTTATACAAATTTTACTGACCTTTCAAGATCCTGCTTTC
ATCCCAGCTCTGGGAAAGCCTTCCCTGAATCCCCAGCCCACAGCAAGTCTTGG
TACTCAAAGTTGCTTGTGCATCTTGGTATCATCCCCACTGCTACACACAGCTAC
GCAAAGTAAGGCACTTAAGAAATATTCGCTACACGGATGAACAAATGAATGAA
TGAATGGGGCAGTACCACCTGTGACATTTATCTTTTAATGGACTTTAAGTGCCT
TGGCTCTTTGACAGCACTGGTCTCACTCCAGAGCATTCCTCTAGCTAGCAAAG
CAGTGTGTGGATGTGTGGGTGTAGGTGGCGATACAGACCTCACTGGATGAGGA
CCGCCTCCTGCAAGCTCCACGATCCCGGCAACCCAGTGCTTATAGTTCAGATC
CTACCCCACCCCCTCACCTCCACGCCCCCTTCTGCACTGGCCGGGTTTAGCGA
GCCGCTGCAGCCCTGATCGAGTTAAGGCTGCTGGAGGCGATGCGGACGGGGC
CAGTGACTCGTAGTAGATCCTTCCGCGCGGAGCTCGGGCTGGCGCTTCTTCCT
GCGGGAAACCCCCTGCGCCCAAGGCGGCGGGGCTGAGGCCGCGGCGACAGT
GGGGCGGGGCTTGCGGTGGAGGAGGCGGCTGAGGCGGAAGGACACGAGGTT
GCTTCGCTGCACACCTGAGAAAGTTTCAGCCAAACTTCGGGCGGCGGCTGAG
GCGCGGCGGCCGAGGAGCGGCGGACTCAGCGCGGGGAGTCGAGGCATTTGC
GCCTGTGCTTCGACCGTAGCGCCAGGGCCTGGAGCCTTGAAGCAGGAGGAGG
GGAGGAGAGAGGGGCCTCTATCGGGACCCTCCCCATGTGGATCTGCCCAGGC
GGCGGCGGCGGCGGCGGCGGCGGCGGCGGCGGCGGTGGTGGCGGT
GCGGGCGGGGTAGTAGTGCAGGTGGCGGCGGCGGCGGCGGGCGGGTGG
TGGCGGCGGCGGCGGTGGCGGCGGCGGTGGCGGCGGTGGGTGGTGCGG

CGGCGGCGGTGGCGGGTGGCGGTGGCGGTGGCGGCGGTGGCGGGCGGTGGC
GGCGGTGGTGGCGGCGGCGGCGGCGGCGGTGGCGGTGGCGGTGGCGGTGGC
GGTGGTGGCGGCGGGAGGAGGTGGCGGCGGGAGGAGGCGGGCGTGGCGGAG
GGGAGGGTGGCGGCGGAGGGAGGTGGCGGCGGAGGTGGCGGTGGAGGGAG
GGTGGCGGCGGGAGGAGGCGGCGGCGGAGGAGGCGGCGGGTGGAGGAGGC
GGCGGCGGAGGGAGGGCGGCGGCGGAGGAGGCGGCGGCGGAGGAGGCGGT
GGCGGAGGAGGCGGCGGCGGAGGAGGCGGCGGTGGAGGAGGCGGCGGCGG
AGGAGGCGGCGGCGGAGGAGGCGGCGGCGGAGGAGGCGGTGGCGGCGGAG
GAGGCGGCGGTGGAGGAGGTGGCGGCGGAGGAGGCGGCGGCGGAGGAAGG
AGGAGGCGGCGGGCGGAGGAGGCGGC

F4-II:2-read 1

GTGGTGTCGGGCAAAGCCCTCTCTTTGACTGGAAGGGATGAATTATGATTTTTC
AGGAAGTTCATTAAGAGGAGGAGGAAGGGGACTACAGGAGTCATTCCAAGCC
AAAGGAACAAAAGATACAGGACAGAAACAGCAAGATATGTTTGGGGGGGAA
CTCAAGTTAGCTAATACTGTTATTAAAAAGAAGTCTACAACAGGGGCAGGGAG
GACAGGAGATGGGGCCCAATGACTAAAGCGGCCTTGTATGTACCATGTTTAGA
AGTTTGAATTTTATCCTATAGAGTCAGAGAGGTTCATGGGTGGATTTCCCATCC
CAACAGGATTGTAAACTCCCACATCCAATTAGTCACACCTCCATTTTTTTTTTC
TGTTTCTTAACATTGTCTCAGATTCATTCCCTTCTGAAATCTACCTTATTAGTTC
AGGTCTTCTTAAATTTGTGCCTGGACTGTTCACTCAACTCTTGACTGGTCCTAA
ATCTAGTTCACCCCTTGCTCCCAACAATCAATTTTCTACAGAACTATCAGAGCA
ATCTTCCCTTTCCATCAATCTCAACTCTGTCTAAAAAACTTTCAATAGTTTTATG
TGACTTAAGAGGTAGCTGCCTCCCCACCCTTTCAGCTTCTTGCCATTCAAGGC
CGTGAAGCAATTTGGTCCCAACCCCTTCTTCCGGCCCTTGACTCTCTCACTGCT
AACAGAGACAGAACTCATATTTACTTACTCTTTTGACTTTCCGTACCTTTGCTT
TCACGAGGCCCTTCCTTGGAATGATATGTCCATACAACCATTGTTGTTGTTCTTA
CTTTAGCTCTTTTCTTAGCCCACTTGTACCCAGGTCGCGATCACACCCCTACTC
TCCTGCTCTGAAGTAACTACTATTCTGGCGATAGTCTCAATATCTATATCTATATC
TATATATGCATCCACAGATAATGTGTAGTATTGTCTTGAAAAAAATATACATAAA
TGGTATACTTGTCTCCCTTCACATTAGTTTTTTTCATCACTATCATTTGTTTTTTA

AGATTTATCCTTATTGACATAAATAGCTATAGTCTTTTTCAATTACTGTGTGTTGC
ATTTCATTTTATAAATATACTATACATTATTCATCCATTCCTCTCATATAAGTGGGC
ATTTATGTTGTTTTCAAGGTTTTGCTAGCTACAGTAAGTTACAACTAATATCCTT
AGCTTAGCTGGTCTCCTTTTGTCTTATACAAATTTTACTGACCTTCAAGATCCTG
CTTTCAGTCCTGGCTCCTCTGGAAAGCCTTCCCCTGAATCCCCCAGCCCACAA
AGTTCAGTACCAAAGTTGCTTGTGCATCTGGTATCATCCCACCGCGTTGCCATT
AATAGCCACGCAAAGTAAGGCATTTAAGAAATATTCTCACATGGATGAACAAA
TGAATGAATGAATGGGGGCAGCACCACCCGTGATGAAGCTCTATTCCCTTTTTA
ATGGACTTTATGCCTTGGCCCACTTTCCTCTTTGACAGCACTGGTCCTACTCAG
GAGCATTCTCTCTAGCTAGCAAAGCAGTGTGTGGATGTGTGTGTAGGTGGCGA
TACAGACCACTGGATGAGGACCGCCTCCCCTGCAAGTTCACGATCCTGGCAAC
CCAGTGCTTCAGTTCAGAGTCCTACCCACCCCCCCACCTCCACGCCCCTTCTG
CACCGGTCGCTTGCGGAGCCGCTGCAGCCTCTGATCGAGTTAAGGCTGCTGAG
AAGGATGCGGACGGCCAGTGACTCGTAGCAGATCCCTCCGCGCGGAGCTCAC
GGCGCTCTTTCCTGCGGGGAAACCCCTGGGTGCCCAAGGGCGGCGGCTGAGG
CCGCGGCGACAGTGGGCGGGGCTTGCGGTGGGAGGAGGCGGCTGAGGCGGA
AGGACACACGAGGCTGCTTCGCTGCACACCTGAGAAAGTCAGAAACTGGCG
GCGGCGAGGTGGCGTGCCGGTGGAGCGTGGACTTTGTGCGGGGGAGTCGAG
GTATTTGCCTGTGCTTTCGGGAAGTGCTGTGGCTTGAGCTTGAAGCAGGAGGA
GGGGAGGAGGGTTTTCTATCGGGACCCCTATGTGGATCGCCCAGGTGGTGGTG
CGGTGGCGGTGGTGGCGGCGGTGGCGGCGGCGGGTGGTGGCGGGTGGTGGT
GGTGGTGGTGGTGGTGGTGGTGGTGGTGGTGGTGGTGGTGGTGGTGGTGGTG
GTGGTGGTGGCGGTGGTGGTGTGTGGTGGGTGGTGGTGGTGGTGGTGGTGGT
GGTGGTGGTGGTGGTGGTGGCGGGTGGCGGTGGTGGTGGTGGCAGGCGGGTG
TGGTGGTGGTGTGGTGCGGTGGTGGTGGCGGGGCGGTGGCGGTGGCGGTGGC
GGTGGTAGTGGCGGCGGCGGCGGGTGGCGGTGGCGGCGGGCGGCGGCGGCG
GC

F4-II:2-read 2

GGGCAGATTCTATCCCAACAGGATTGTAAACTCCCACATCCACCCTAGTCACA
CCTCCATTTTTTTCTGTTTCTTTAACATTGTCTCAGATTCATTCCTTCTGAAATCT

ACCTTATTAGTTCAGGTCTTTAAATTTGTGCCTGACTGTTCACCAACTTGACTG
GTCCTTGGGGCTCCAAAGTTCACCTCTGTTGCTCCCCAACAATCAACTCTTTCT
ACAGAACTATCAGAGCAATCTTCTCCTTCCAGCATCAATCTCAACTCTGTTTCA
AAACTTTCATTAGTTTTATGTGACCTAAGAGGTAGCTGCCTCACCCTTTCAGCT
TCGCTATCCAAGGTTGCATAATTTGGTCCCCAACCCCTTCCGCCCTTGACTCCT
CACTGCTAACAGAGACAGAACTCATTTACTTACTCTTTGACTGTACCTTTGTTT
ACGAGGCTCTTTTCCTGAATGATATGTCCATACAACTATTGTTGTTGTTCTTACT
TTTAGCCTTTTCTGAAATTACTTGTACCCAGGTTTGATCACACCCCCTACCTCCT
GCTCCTGGAAGTAACTACTATTCTGGTTATAGTTCTTACTATCTATATCTATATCT
ATATATGCATCCACAGATAATGTGTAGTATTGTCTTGAAAAAATATACATAAATG
GTATACTTTATTCCCTCTGTATTAGTTTTTTCACTATCATTTAGTTTTTTAAGATTT
ATCCCTATTGGATATAAATAGCTATAGTCTTTTCAATTACTGTGTTGCATTTCATT
TCATAAATATACCATACATTATTCATCCATTCCTCTAAGGGTGGGCATTTATGTTG
TTTTCTAAGGTTTTGCTATTACAGTTACAACTAATGTTCCTTAGCTTAGCTGGTC
TCCTTGTCTTATACAAATTTTACTGACCTTTCAAGATCCTGCTTTCATCCCAGCT
CCTCTGGGAAAGCCTTCCTGAATCCCAGCCCACAGCGGCAGTAAAAGTCTTG
GTACTGTGCTGCTTGCATCTTTTGGTATCATCCTAATGTTAAGCAATACGCTACG
CAAAGTAAGCGTTAATTTAAGAAATATTCACACATCGGATGAACAAATGAAGA
ATGAATGGGGGCAGCACCACCCGTGATGGCTCTATCTTTTAATGACTTTATGCC
TTGGCCCACTTTCCTTTTGACAGTACTGTCCTACTCCAGAGTATCTCTAGCTAG
CAAAGCAGTGTGTGTGGATGTGTGGGTGAGGTATATACAGACCTCATTACGAG
GACCGCCTCCCTGTAAGTTCACGATCCCGGCAACCCAGTGCTTGAAGTTCAGA
GTCCACCCCACCCCTCACCTCCACGCCCTTCTGTACCATCAAGCAGAGCCGCT
GCAGCCCTGATCGAGTTAAGGCTGCTGGAGAAGGATGCGGACGGGCCAGTGA
CTGTAGTAGATCCCTCTGCGCGGAGCTCGCCGCGCTTTCTTCCCGCAGGGAAA
CCCCTTGGTGCCCAAGGTAAGGGGCTGGAGCGTGGCGACAGGGAAGGGGCTT
GCGGTGGGAGGAGGCGGCTGGAGGCGGAAGGACATGAGGTTGCCGCTGCAC
CCGAGAAAGTTTCAGCCAAACTTCGGGCGGTGGTTGAGGCGGCGGGCCGAGG
AGTGCGACTTCGGGGCGCGGGAGTCGAGGATGGTGCCTGTGGGCTGGACTGT
AGCGCCAGGGCCTGGAGCTTTTGAAGCAGGAGGAGGGGAGGAGAGAGGGTT

TCTATCGGGACCCTTCCCTATGGATTCGTTTCAGGTGGCGGCGGTGGCGGTGC
GGTGGTGGCGGTGGTGGTGGCGGTGGTGGCGGCGGTGGTGGCG
GTGGTGGCGGGTGGTGGCGGCGGTGGTGGCGGCGGTGCGGCGGCG
GCGGTGGCGGCGGCGGTGGTGGTGGCGGTGTGGCGGGTGGCGGTGGCGGCG
GTGGTGGTGGTGGCGGGCGGTGGCGGCGGCGGCGGCGGTATGGTGCGGC
GGCGGCGGCGGCGGTGGCGGCGGCGGCGGGTGGTAAGTGCGCGCGCGC
GGTGGCGCGGCGGCGGCGGCGGCGGCGGCGGCGGCGCAGGCGGCGGC

<u>F4-II:2-read 3</u>

GACTACAGGGGTGTTCCAACCAAAGAGACAAAGATGCAAGACAGAAACAAC
CAGTTATGTTTGGGGAATCTCAGTTAGCTGATACTGTTGTTGACAGTTAAACAG
GGCAAGGAGGACAGGAGATGGGCCCCAATGAAACCTTATATATGCCATGTTTA
GAAGTTTAGATTTTATCCTGTAGAGGTCGAGGGAGAGGGTTGTGGTTCCCACA
TCCCAACAGGTTATAAGCTCCACGTCAATTGATCACACCTCCATTTTTTTCTATT
TCTTAACATTGCTCCTCAGATTCATTCCTTCTAAAATCCCTACCTTATTAGTTCA
GATCTTCTTAAATTTATTGCCTGGACTGTTCACCCAACTCTTCAGCTGGTCCCT
GGGGGCTCCAGTTCCTGCCTCCCCCAACAATCAATTTCTACAGAACTATCAGA
GCAAATCTTCCACTCCATCAATCTCAACTCTGTTCAAAAACTTTCCAATGGTTT
ATACTTGACCTAAGAGTGGCTACCTCCCACCTTTCAGCTTTGCCATTCAAGGCT
GCATAATTTTGGTCCCAACCCCTTTCCGTCACTTGACTCCTCCTGCTAACAGAG
ACAGAACTCATTTCGCCTCTTTTGAGCTGTACCTTTGCTTTCTGGGGCTCTTTC
CTGGAATGATCATGTCCTTCAACTATTTATTTGTTGTTCTTACTTTTTAGCTCTTT
TCTTGGCTGCTTGCTGATTTTAATCACCCCCTCTCCTGCCCTGGGAGTAACTGC
TATTCTAGTTATAGTCTCTTTTAATATCTATATCTATATCTATATATAGCCGTCCAC
AGATAATGTGTAGTATTATCTTGAAAAAAATATACATAAATACTTGTATACTTTGT
CTCCCTCTAGATTAGTTTTTTCCATATCATTTTGTAGTTTTTTAAGATTTATCCTAT
TATTGACATGAAATAGCTATAGTCTTTTTCAATTCTGTGTGCATTTCATTTTATAA
ATATACACAGCATTATTCATCCATTCCTCTAAGGGGTGAGGCATTTATGTTATTT
CAAAGTTTTGCTGACTTACAGTGTTACAAAAATATCTTCCAACAACTGGTCTCC
TTGTCTTATGCAAATTTTACTGTTATACCAAAGGATCCTGCTTTCATCCAAGCTC
CTCTAGGAAAGTAACCCTAAGATCCCCCAATAATGACAAGTCTTAGTATAAAGT

TGGCAGCCATCTTTAGTATCCATCACTACCAAACACAGCTACACCAAATGAGC
ACTTAAGAAATATTCACTACAGATGAACAAATAGATGAATAGATGGGGCAGCA
CCATCCGTGATAAATATTCCTTTTTAATGGACTTTGGCGCCTTGGCCCTTTCATG
TGACAGCACTGGTCCTACTCAGACATTCCTCTAGCTAACAAATGGTGGCCAAC
TGGATGTGTGGGTGTAGTGGCGTCTGAACCTCACTGGATGAGGACCATACCTC
TCCTGCAAGTTCCACGATCCAGCAGCTCAGTGGCCGGGATTCAGAGTCCTGCC
CACCCTGCCTCCACGCCCCTTCTGCACTGGTCAAGCCCAGCAGGCCACTGCA
GCCTGATCGAGTTAAGGCTGTACTGGAAAGGATGCGGACAGGGCCAGTGACT
CGTAGTAGATCCTCACTTTGGAGCTCAGGCAGCCTTCTTCCTGCAGGGAAACC
CCTGGGTGCCCAAGGAGGCAGCGGGGCCGGGCCACGCAACGACAATCAGGA
AGACAGGGCTTGCAGTGGGAGGGAGGCGGCTGGGGCGGGGAAGGGACTACA
CGGGCTGCTTCGCTGCACACCGAGAGAGATTTCAGCCAAACTTCCGGGCGGC
AGCTGGGGCAGCAGCCGAGGCCGGCACAGACTCAGGGCATGAGGAGTCAAG
GGCATTGCCTGGCTGGTTCCCGCCATGGCGCAGGGCCTGAGCCTTTGAAGTAG
GGAGGAGGAGATGGGGCTCCTCTATCAGGACCCCACCATGTGGATCTGCCCAG
GCGGCGGCGGCGGCGGCAGCAGCGGCAGCAGCGGCGGCTGGCGGCGGCGGC
AGCGGCGGCGGCTAACGGCAACAGCACCAGCAACAACGGCGGCGGCGGCGG
CGGCAGCAGCAGCAGCAGCAGCGAGCAGCAGCAACGGCAGCAACAGCAGCA
GCAGCAGCGGCGGCAGCAGCAACAGCAACAGCAGCAACAGCAACAACAGCA
ACCGGCAACAGCAACAGCAACAACAACAGCAACAGCAGCAGCAGCAGCAAG
CAGCAGCAGCAGCGGCAGCAGCGGCGGCAGCAGCAGCAGCAGCAGCAGCAG
CAGCAGCAGCTTGTGGCAGCAGCGGCGGCGGCAGCAGCAGCAGCAGCAGCA
GCAGCAGCAGCAGCAGCAGCAGCGGC

F4-II:2-read 4

GAAGACAGTGGAGGACTATATATTAGAGGAGAATTTATTGTGGTGTGACTGAC
CTTCTCTTTGGACTGAGAAAAATTGAATTATGATTAAGTTCATTAGAGGAGGAG
GGAAGGGGACTACAGAGTCATTCAAGCCAAAGACAAAAGATACAGGGACAG
AAACAGCAAGATATGTTTGGGGGAATCTCAGGATTGGCAATACTACATTATTGT
GAAGTTCACAACAGGGGCAGGAGGACGGAGATGCAGAATCTCAGTTCTATGG
GTGAAGTTTCCATCCCAACAGGATGTAAACTCCCATCCAATTAGTCACCTCATT

TTTTTCTGTTTCTTTAACATTGTCTAGATTCATTCCTTCCCAAAATCTGTAATAGT
TCAGGTCTTCTTAAAATTTTGCCTGGACATTCACCAACTCTTGACGCCTGATTT
TCCAAGACCAGTTGCTCTTGCTCCCAGCAATCAATTTTCTACAGAACTATCAGA
GCAATCTTCTTTATCAAGTCTCAACTCTGTTCAACTTTCAATAGTTTTATGTGAC
CTAAGTGGCTGCCTCCCCCACCCTTTCACGCTTTGCAATTCAAGGCTGCATGA
ATTTGATTATTGCCTAGGTCCTTGACTCCTCTCACTGCTAACAGAGACAGAAGC
TCATTTGCCTCTTGACTGTTACCTTTTTGCTTTCCACGAGGCTCTTTCCTGGAAT
GATATGTCCATACAAGCATTATTTATTTCTTGCCTGCTCTTTTCTTATTTTTGTAC
CCAGGTTTGATCCACCCCCTACTCTACATGGAGTAACACTATTCTGGTTATAGT
CTCTTATTTATCATATCATATCTATATATATATACATCCACAGATAATGTAGTATTAT
CTTGAAAAAAAATATACATAAATGGTATCTTGTCTCACTATAGATTAGTTTTCAC
TTCTATAAATTTAGTTTTAAGATTTGTCTAATACAGAGCATAAATAGCTATAGTCT
TTCTCAATTACTGTGTTGCATTCATTTATAATATACCATACATTGTTAATGCATTC
CTCTAGAGGTGGGGCATTTATGTTGTTTTCAAGTTTTGCTATTACAGTGTTACA
ACGAATATCCCTTATAAATAGCTGGTCTCCTTGTCTTATACAAATTTTACCCCGA
CTTTCGAGATCCTGCTTTCATCCCAGCTCCTCTGGGGAAAGCCTTCCCTAGATC
CCTGACTGGCAGCAAAGTCTCCTCAAAAATTTGCTTGCATGCATCTTTGGTATC
ATCCACTATAATACAGCTGCATAAAGTGAGCCACTTAGAAATATTCTTCCACGG
ATGAACAAATGAATAGATGAATGGGCCAGCACCACTCCGTGATGGCTCTATTC
CTTTTTAATGGACTTTGGCGCCTTGGCCCACTTTCCTTCTTTGACAACTGGTCC
TACTCCAGGCATTCCTCTAGCTAGCAAAGCAGTGTGGATGTGTGTGGGTGTGG
GTGGCGATACGAACCTCACTGGATGAGGGACCGCCTCCTGCAGGTTCACGATC
CCAAACTCCAGTGCTTGAAGTTCAGGGTCCTACCCCACCCCTCTCCGCCCCTT
CTACTTTGTGGTCAAGCCAGCAGGCCGCTGCAGCCCTGATCGAGTTAAGGCTG
CTGGAGAAGGATGTTGGACAGGGCAATGACTCGTAGTAGATCCCTCGCGCGG
GCTCGGGCCGGCGCTTCTTCCTGCAGGAAACCCCCTGGGTGCCCAAGGCAGG
GGCCGAGGGCCACTTGACGACAGTGGGAAGACAGGCTTACCTGCAGTGGGA
GGGAGGCGGCTGAGCTGAAGGACACACAGGAAAGCTGCTTCGCTACACTGA
GAAAGTTTCAGCCAAACTTCGGGCGGCGGCTGGGGCGGCAGCCGAAGACGG
CGGACTCAGGGCGCAGGGGAGTCGAGGCATTGCGCCTGTCGCTTCGGACCGT

AGCGCGGGGCTGGGCGCTGGAAGGCAGGAGGAGGAGGAGAGTGGGGCTCCT
TATCGGGACCCCCTCCCCGTGGATCTGCCCAGGCGGCGGCGGCGGCGGCGGC
GGCGGCGGCGGCAGCGGCGGCGGCGGCGGCGGCGGCGGCGGCAGCGGCGGC
GGCGGCAGCGGCAACGGCAGCAGCTGGCCCGGCAGCGGCAGCGGCGGCGGC
GGCGGCAGCGGTGGCTGGCGGTGGTGGCGCGGCGGCCCGGCAACGGCAACG
GCGGCAGCGAAGGCGGCGGCGGCAGCAGCGGCGGCGGCGGCGGCGGCA
ACGGCAGCAGCGGCGGCAGCGGCGGCGGCGGCTGCGGCGGCGGCGGCGGCG
GCAACGACAGCAGCGGCGGCGGCGGCAACGGCAGCGGCGGCGGCAGCGGCA
GCGGCAACGGCAGCAACAGCAACAGCAGCAGCAGCAGCGGCAGCGGCAGCG
GCAGCGGCAGCGGCGGCGGCAGCGGCGGCGGC

F4-II:2-read 5

GAGTCAGGGAGAGGGTTCTATGGGTTGCAGTTCCCATCCCAACAGGATTATAC
AAGCTCCCACATCAATTAGTCCACCTCCATTTTTTTTTCTGTTTCTTTAACATT
GTCTCAGATTCATTCAACTTCTAAATCTACCTTATTAGTTCAAGTCTTCTTAAAT
TTGTACTGGACTGTTCACCAACTCTTGACTGGTCTGGGGCTCCAGTTCACCTC
TTGTAACAATCAATTTTCTACAGAACTATCAGGTAGTCTTTGCTTTCCATCAATC
TCAGCTCTGTTCAAAAACTTTCCAATAGTTTATGTGACCTAAGAGGTAGCTGCC
TCCCCACTTACTTTCAGCTTGCCATTCAAAGGCTTGCATAATTTGTGATCCCAC
AACCCCTTTTCCGTCCTTGACTCCTCTCCTGCTAACAGAGACAGAACTCATTTT
ACTTACTCTTTTGACTGTACCTTTGCTTTCCCGAGGCTCTTTCCTGGAATGATAT
GTCCAAAAGCCCCCAACTGTATTGTTATTTCTTACTTTAGCTCTTTTCTTAGCCC
ACTTGTACCCAGGGTTTGATCCACCCCTACTCTCCTGCCTGGGAGTAACTACTA
TTCTGGTTATAGTCTCTTAGCCTCTATATCTATGTCTATATGTGCATCCACAGATA
ATGTGTAGTATTGTCTTGAAAAAAATATACATAAATGGTATGCTGTCTCCTAGAT
TAGTTTTTCACTATCATTTTGGTTTTTAAGATTTATCCTATTGACATAAATAAGCT
ATAGTCTTTTCAATTACTGTGTTGCATTTCATTTTACTAAATATACCATACATTAT
TCATCCATTCCTCTAAGGGTGGGCGTTGCAGCAACATTTTCAAGGTTTTTACTA
TTACAGTGTTGTGAATATCCTTAAAAACAGCTTTTGAGTCTCCTTGTCTTATACA
AATTTGCCCCAGCCTTTCGAATCCTGCTTTCATCCCAACTCCTCTGGAGAAGCG
CTTCCTGGTCCCCCAGCCCACAATGACAAAATCTTGGTACTCCAAAAGTGTAT

GCATCTTTGGTATCATCACTGCCAAGCTTACACAGCTACATAAGTAGGCAGCAA
AATATTCGCTACACGGATGAACAAATGAATGAATGAATGGGCAGCACCACTCA
CGTGATGGCTCTATTCCTTTTAATGGACTTTGGCGCCTTGGCCCACTTTCCTTCT
TGACAGCACTGGTCCTACTCCAGAGCATTCCTCTAGCTAACTTCGAAAGCAGT
GTGTGGATGTGTGGCGAATTGTAGGTGGCGATACAGACCTCACTGGATGGGGA
CCGCCTCTCCTGCAAGTTCACGATCGAAGAACTCCAGTGGCAGGTTCGAGTCC
TCCACCCCTCACCTCCGCCCCTTCTGCACTGGGTGAAGCTGGCGAAAGGCTGC
TGCAGCCTGATCCGATTTAAGGCTGCTGGGAGGAAGGGATGCGGACGGGGCC
AGTGACTCGTAGTAGATCCCTCCGCGCGGAGTCGGGCCAGCGCTTCTTCCTGT
GAGAAACCCCTGGGTGCCCAAAGGCAGCAAAGACCCGAGGGCCGCGGCTCA
ATGGGACAGGGCTTGCGGTGGGAGGGAAGCGGCTGAGACGGAAGGGACCCC
ACACGAGGCTGGCCTTCACTGCCGGAAGGTTCAATGAAACTTCCGGGCGGCG
GCTGACGGCAGCCGAGGGCGGCGGCGCGGACTCAGGGGCGCGAGGTCGGGC
ATTTGCGCCTGTGCTTCGGACCGTAGCGCCAGGGCTTCTGAGCCTTTGGAAGC
AGGAGGAGGAGAGAGTGAGAGGGCTCCTCTATCAGGACCCCCCTCCCCATGT
GGATCACAGGCGGCGGCGGCGGCATGCAGCAGCGTGGCAGCAGCAGCAGCG
CGGCAGCCAGCGGCAGCAGCAGCGGCGGCGGCAGCTGGCAGCGGCAGC
GGCGGCGGCGGCAGCGGCGGCGGCGGCGGCGGCGGCGGCGGCAGCAGC
GGCGGCGGCGGCGGCGGCGGCAACGGCGGCAACGGCAGCGGCGGCAGCAGC
GGCGGCGCGGCGGCGGCGGCGACGGCGGCGGCGGCGGCGGCGGCGGCG
GGCGGCTGGCGGCGGCACGGCGGCGGCACGGCGGCAACGGCAGCGGCAGCA
GCAGCGGCAGCAGCAGCAACGGCAGCAACAGCAACGGCAGCAGCAGCAGCA
GCGGCAGC

F4-II:2-read 6

GGCAGGTCTATGCCTATACTGACTTGTACTTGCTTAGTGTTGTTTACATTCTAGT
TCAACATCTATTGCGGATAGGACACTCGGGGGATTAATAGGCTGTGGTCTCTTG
CCCACAGAGACCCCGTTAAAACTTAGTTGAGAAACACAGGAAACAAAGAGTC
CTGTGTGAAAAATAAGTATTGGTGTTAAGATGCAGTGAAGACAGTGGAGGGA
CTGTCTGTTAGAGGAGGAATTTATTGTGGTGTCGGCAAAGCTCTTGGATTGAA
GGATGAATTATGATTAGAAGTTCATTACAGAGGAGGAGGGAAGGGGACTACAG

GAGTCATTCTGAAGCCAAAGGAACAAAAGATACAGGGACAGAAATAGCAAGA
TGTTTGGGGGAATCTCGTTACTACTGTTATTGTGTGAAGTTCACAACAGGGGTA
TAGGGAGGACAGGATGGGGTCCAATGACTAAAAGGTCTTGTATGTACCATGTT
TAGAAGTTTGAATTTTATCCTATAGGGAGCTAGGAGAGGTTCTATGGGTAGGAT
TTCCATCCCAACAGGATTGTAAACCCCACATCCAATTAGTTTCACCTCCATTTT
TTTTTCTGTTTCTTTAACATTGTCTCAGATTCATTCCTTCTGAAATCTACCTTAC
GAATTCAGGGTCTTCTTAAATTTGTGCCTGGACTTCACTAACTCTTGACTGGTC
CTGGGGGGCTCCAGTTCACCTCTTGCTCCCAACAATCAATTTTCACAGAACTAT
CAGAGCAATCTTCCTTCCATCAATCTCAACTCTGTTCAAAATCTCAATAGTTTT
ATGTGACCTAAGAGGTAGCTGCCTCCCACCCTTTCAGCTCTGCCATTCAAGGC
TGCATAATTTGGTCCCACCCCTTCCGGCCCTGACTCACTGCTAATAGAGACAGA
ACTTCATTTACTTACTCTTTTGACTGTACCTTTGCTTTTACTTAGGGCTCTTCCT
GGAATGATATGTCCATACAACTATTGTTACATTTTACTTTAGCTCTTTTCTTAGCT
CATTTGTACCCAGGTTTGATCACAATTCCCCCTACTCTCCTGCTCCTGAAGTAA
CTACATTATTCTTATTATAGCTTTTAGTTATTATATTAGGTTATAGGTGTACCATGG
AGCATGTGTAGTATTGTCTTGAAAAAAATATACATAAATGGTATACTTTTGTCTC
CCTCACATTAGTTTTTTCTTACTATCATTTAGTTTTTTAAGATTTATCCCCATTGG
AGTGTAAATAGCTATAGTCTTTTCAATTACTGTGTTGTACTTCCTCATTTTTATAA
ATATACCATACATTATTATCCATTCCTCTAAGGGTGGGCATTTATGTTGTTTCTCA
AGGTTTTGCTATTACAGTGTGTTACAACGACATCCTTAGCTTAGCTGGTCTCCT
TGTCTTATACAAATTTTACTGACCTTTCAAGATCCTGCTTATCCCAGCCCTCTGG
AAAGCCTTCCCTGAATCCCCCAGCCCACAGCGCCAGCGGGTAAAGTCTGGTA
CTCTGTGCTGCTTGTGCATCTTTGGTATCATCCCACTGCTAAGCACACACAGCT
ACGCAAAGTAAGGCACTTAAGAAAATATTCGCTACACGGATGAACAAATGAAT
GAATGAATGAAGCAGTACCACTCCGTGATGGCTCTATCTTTAATGGACTTTGGC
GCCTTTGGCCCACTTTCTCCTTCTTGACAGCACTGGTCCTACTCCAGAGCATTC
CTCTAGCTAGCAAAAGCAGTGTGTGGATGTGTGGGTGTAGGGTGGCGATACAG
ACCTCACTTCGGATGAGGACCGCCTCCCTGCAAGTTCACGATCCCGGCAACTC
CAGCGCTTGAAGTTCAGGAGGTTCCACCCCCACCCCCTCACCCACGCCCCTTC
TGCACTGGTCAAGCCATGAGCCGCTGCAGGCCCCTGATCGAGTTAAGGCTGCT

GGAGAAGGATGCGGACGGGGCCGCGACTTCGTAGTAGATCCCTTCCGCGCGA
GCTCGGGCCGGCGCTCTTCCCGTGGAAACCCCTGGGTGCCCAAGGCGGCGTG
CCGAGGCTGCGGTGGCGACAGTGGGGCGGGGCTTGCGGTGGGAGGAGGTGG
CTGAGGCGAAGGACACACGAGGTTGCTTCGCTGCACACCTGAGAAAGTTTCA
GCCAAACTTGGGCGGTGGTGGCGGCTGAGGCGGCGGCTGAGGAGCGGCGGG
ACTCAAGGGGGAGTCGAGGTATTTGCGCCTCGTGCTTCGGACCGTAGCGCTAG
GCCTGAGCCTTTGAAGCAGGAGGGAGGGGAGGAGAGAGTGGGGCTCTCTATC
GGGACTTTCTCATGTGGATTCGCCCAGGTGGCGGTGGCGGGTGGGCGGCGGC
GGTGGCGGCGGCGGGTGGTGGCGGTGAAAAGTGGCGGCGGCGGCGGTGGCG
TGGCGGCGGCGGCGGCGGCGGTGGCGGCGGCGGCGGCGGCGGCGGTGGTGG
CGGCGGTGGCGGTGGCGGCGGCGGTGGTGGCGGTGGCGGCGGGCGGTGGCG
GTGGCGGCGGCGGCGGTGGCGGCGGGCGGCGGCAGTGGTGGCGGCGGTGGC
GGTGGCGGTGGCGGCGGCGGTGGCGGCGGCGGCGGCGGCGGCGGCGGTGGT
GGCGGCGGCGGCGGCGGCGGCGGCGGCGGCGGCGGTGGCGGCGGCGGCGGC
GGGTGGCGGC

<u>F5-II:1-read 1</u>

GGCAGGTCTATGCCTATACTGACTTGTACTTGCTTAGTGTTGTTTACATTCTAGT
TCAACATCTATTGCGGATAGGACACTCGGGGGATTAATAGGCTGTGGTCTCTTG
CCCACAGAGACCCCGTTAAAACTTAGTTGAGAAACACAGGAAACAAAGAGTC
CTGTGTGAAAAATAAGTATTGGTGTTAAGATGCAGTGAAGACAGTGGAGGGA
CTGTCTGTTAGAGGAGGAATTTATTGTGGTGTCGGCAAAGCTCTTGGATTGAA
GGATGAATTATGATTAGAAGTTCATTACAGAGGAGGAGGGAAGGGGACTACAG
GAGTCATTCTGAAGCCAAAGGAACAAAAGATACAGGGACAGAAATAGCAAGA
TGTTTGGGGGAATCTCGTTACTACTGTTATTGTGTGAAGTTCACAACAGGGGTA
TAGGGAGGACAGGATGGGGTCCAATGACTAAAAGGTCTTGTATGTACCATGTT
TAGAAGTTTGAATTTTATCCTATAGGGAGCTAGGAGAGGTTCTATGGGTAGGAT
TTCCATCCCAACAGGATTGTAAACCCCACATCCAATTAGTTTCACCTCCATTTT
TTTTTCTGTTTCTTTAACATTGTCTCAGATTCATTCCTTCTGAAATCTACCTTAC
GAATTCAGGGTCTTCTTAAATTTGTGCCTGGACTTCACTAACTCTTGACTGGTC
CTGGGGGGCTCCAGTTCACCTCTTGCTCCCAACAATCAATTTTCACAGAACTAT

CAGAGCAATCTTCCTTCCATCAATCTCAACTCTGTTCAAAATCTCAATAGTTTT
ATGTGACCTAAGAGGTAGCTGCCTCCCACCCTTTCAGCTCTGCCATTCAAGGC
TGCATAATTTGGTCCCACCCCTTCCGGCCCTGACTCACTGCTAATAGAGACAGA
ACTTCATTTACTTACTCTTTTGACTGTACCTTTGCTTTTACTTAGGGCTCTTCCT
GGAATGATATGTCCATACAACTATTGTTACATTTTACTTTAGCTCTTTTCTTAGCT
CATTTGTACCCAGGTTTGATCACAATTCCCCCTACTCTCCTGCTCCTGAAGTAA
CTACATTATTCTTATTATAGCTTTTAGTTATTATATTAGGTTATAGGTGTACCATGG
AGCATGTGTAGTATTGTCTTGAAAAAAATATACATAAATGGTATACTTTTGTCTC
CCTCACATTAGTTTTTTCTTACTATCATTTAGTTTTTTAAGATTTATCCCCATTGG
AGTGTAAATAGCTATAGTCTTTTCAATTACTGTGTTGTACTTCCTCATTTTTATAA
ATATACCATACATTATTATCCATTCCTCTAAGGGTGGGCATTTATGTTGTTTCTCA
AGGTTTTGCTATTACAGTGTGTTACAACGACATCCTTAGCTTAGCTGGTCTCCT
TGTCTTATACAAATTTTACTGACCTTTCAAGATCCTGCTTATCCCAGCCCTCTGG
AAAGCCTTCCCTGAATCCCCCAGCCCACAGCGCCAGCGGGTAAAGTCTGGTA
CTCTGTGCTGCTTGTGCATCTTTGGTATCATCCCACTGCTAAGCACACACAGCT
ACGCAAAGTAAGGCACTTAAGAAAATATTCGCTACACGGATGAACAAATGAAT
GAATGAATGAAGCAGTACCACTCCGTGATGGCTCTATCTTTAATGGACTTTGGC
GCCTTTGGCCCACTTTCTCCTTCTTGACAGCACTGGTCCTACTCCAGAGCATTC
CTCTAGCTAGCAAAAGCAGTGTGTGGATGTGTGGGTGTAGGGTGGCGATACAG
ACCTCACTTCGGATGAGGACCGCCTCCCTGCAAGTTCACGATCCCGGCAACTC
CAGCGCTTGAAGTTCAGGAGGTTCCACCCCCACCCCCTCACCCACGCCCCTTC
TGCACTGGTCAAGCCATGAGCCGCTGCAGGCCCCTGATCGAGTTAAGGCTGCT
GGAGAAGGATGCGGACGGGGCCGCGACTTCGTAGTAGATCCCTTCCGCGCGA
GCTCGGGCCGGCGCTCTTCCCGTGGAAACCCCTGGGTGCCCAAGGCGGCGTG
CCGAGGCTGCGGTGGCGACAGTGGGGCGGGGCTTGCGGTGGGAGGAGGTGG
CTGAGGCGAAGGACACACGAGGTTGCTTCGCTGCACACCTGAGAAAGTTTCA
GCCAAACTTGGGCGGTGGTGGCGGCTGAGGCGGCGGCTGAGGAGCGGCGGG
ACTCAAGGGGGAGTCGAGGTATTTGCGCCTCGTGCTTCGGACCGTAGCGCTAG
GCCTGAGCCTTTGAAGCAGGAGGGAGGGGAGGAGAGAGTGGGGCTCTCTATC
GGGACTTTCTCATGTGGATTCGCCCAGGTGGCGGTGGCGGGTGGGCGGCGGC

GGTGGCGGCGGCGGGTGGTGGCGGTGAAAAGTGGCGGCGGCGGCGGTGGCG
TGGCGGCGGCGGCGGCGGCGGTGGCGGCGGCGGCGGCGGCGGCGGTGGTGG
CGGCGGTGGCGGTGGCGGCGGCGGTGGTGGCGGTGGCGGCGGGCGGTGGCG
GTGGCGGCGGCGGCGGTGGCGGCGGGCGGCGGCAGTGGTGGCGGCGGTGGC
GGTGGCGGTGGCGGCGGCGGTGGCGGCGGCGGCGGCGGCGGCGGCGGTGGT
GGCGGCGGCGGCGGCGGCGGCGGCGGCGGCGGCGGTGGCGGCGGCGGCGGC
GGGTGGCGGC

F5-II:1-read 2

GGGGACTACAGGAGCTGGTCTCCAAGCCAAAGGAAACAAAAAAGATAGGGA
CAGAAACAGCAAGATATGTTTGGGGAATCTCAAGTTAGCTAATACTGTTATTGT
GTGAAGTTCAAATAGGGTAGGGAGCAGGTGAGATGTGGGGCCCAATGACGAA
AAGGCCTTGTATGTACCATGTTTAGAAGTTTTGGAATTTTATCCTATAGGAGTCA
GGGAGAGGTTCTATGGGTAGGATTTCATCCCAACAGGATTGTAAACTCCCAGC
ATCCAATTAGTCACACCTCCATTTTTTTTTTCTGTTTCTTTAACATTGTCTCAGA
CTATTCCTTCTGGAAATCTACCTTATTATTAGTTCAGGTCTTCTTTAAATTTGTGC
CTGGACTGTTCACCAACTTGACTGGTCCTGGGGCTCCAGTTCACCTCAAGTTC
TTAACAATCAATTAATTTTTCTACAGAACTATCAGAGCAATCTTCCTTCCATCAA
TCTCAACTTGTTCAAAAAACTTTCAATAGTTTTATGTGACCTAAGAGTAGCTGC
CTCCTCACCCCTTTCAGCTTGCCATTCAAGGCTGCATAATTTGTCCCAACCCCT
TTCCGTCCTTCTTGACTCCTCTCACTGCTAACAGAGACAGAACTCATTTACTTA
CTTCTTTGACTGTACCCCTTGCTTCTCACGAGGGCTCTCCTTCCTAAATGATATG
TCCATACAACTATTGTTGTTGTTCTTACTTTTAGCTCTTTTCTTAGCTCCACTTGT
ACCCAGGTTTGATCACACCCCTACTCTCTCTGCCCTGAAGCACCATTATTCTGT
TATAGTCTCTTAATATCTATATCTATATTCATATATATGCATCCACAGATAATGTGT
GGTATTGTCTTGAAAAAATACATAAATGGTATACTTTGGTCTCCCTCTTATTAGT
TTTTTCACTATCATTTAGTTTTTAAGATTTATCCCTTATTGGACATAAATAGCTAT
AGTCTTTTTCACTACTGTGTTGCATTTCATTTTATAAATATGTACCATACATTATT
CATTCATTCCTCTAAGGGTGGGCATTTATGTTGTTTCTGCTGTTTCTGTTATTAC
AAAAAGTTACAACTAATATCCTTAGCTAGTTGGTCTCTTGTCTTATGGCCAAA
TTTTTACTGACCTTTCAAGATCCTGCTTTCATCCCAGCAGCTCCTCTGGGAAAA

GCCTTCCCTGAATCCCCAGCCCACAGCTGCAAAAGTCTTGGTACTCAAAGTTG
CTTTGTGCATCTTGTATCATCCCACTGCTACACACAATAGCCACGCAGAAAGTA
AGGCACTTAAGAAATATTCGCCACGGATGAACAAATGAATGAATGAATGGGGC
ATACCACTCCGTGATGGCTCTATTCCTTTTAATGGACTTCATGCCTTGGCCCACT
TCTTCTCCTTCTTTTTGACAGCACTGGTCCTACTCCAGACATTCCTCTAGCTAG
CAAAGCAGTGTGTGGATGGGGTGGGGGTGTAGGGTGATACAGACCTCACTGA
CGAGGACCGCCTCCCTGCAAGTTCACGATCCCGGCAACCCAGTGCTTGAAGC
TCAGAGTCCTACCTCAATTCCCCCTCACCTCCACGCCCTTCTGCACTGGTCGAC
AGTGAGCCGCAGCCCCTGATCGAGTTAAGGCTGCTGGAGAAGGATGCGGACG
GGGCCAGTGACTCGTAGTAGATCCCTCCGCGCGGAGCTCGGGCCGGCGCTTCT
TCCTGCGTGGAAACCCTGGTGCCCAAGGCGGCGGAAGCCGAGGCCGCGGCG
ACAGTGGGGCGGGGCTTGCGGTGGGAGGAGGCGCTGAGGCGGAAGGACACA
CGAGGCTGCTTCTTGCCGCAATACCACCCGAGAAAGTTTCAGCCAAACTTCGG
GCGGCGGCTGAGGCGGCGGCCGAGGAGCGGCGGACTTTTCGGGGCGCGGGG
AGCGAGGCATTTGGTGCCTGTGCTGGACCGTGCGCCTGTGCTCTGAGCTTTGG
GGCAGGAGGGGAGGGAGAGAGTGGGCTTCCTTATCGGGACCCCTCCCTATGT
GGACTCGCTTCCAGGCGGCGGCGGTGGCGGCGGCGGCGGCGGTGGCAAGGT
GGTGGCGGTGGCGGCGGCGGCGGCGGCGGTGGTGGCGGCGGCGGCGGGCGG
CGGTGGCGGCGGTGGCGTGGCGGCGGCGGCGGCGGCGGCGTGGCGGCG
GCGGCGGCGGCGGCGGCGGTGGCGGCGGCGGCGGCGGCGGCGGCGGTG
GCGGCGGTGGCGGTGGCGTGGCGGCGGCGGCGGCGGCGGCGCGGCGGC
GGCGGCGGCGGCGGCGGCGGC

F5-II:1-read 3

GTGGAGGGACTGTCTCTGTTATTAGAGGGAGGAATTTATTATTGTGGTGTCGGC
CGTGGTCTTTGGACTGAAGGATAGTTATGGTAGAAGTTCATTAGAGGAGGAGG
GAAGGGGACTACAGGAGGGTCATTCAGGCAAAGGAACAAGATACAGGGACC
CAGAAACAGCAAGATGTGTGGGGGAATCTCAAGTTAGCCAATACTGTTATTGT
GTGAAGTTCACAACAGGGCAGGGAGGAGAGGACAGGAGATGGGGCCCAATG
TGAAAAGACCTTGTATGTACCATGATTTGAATTTTTATCCTATAGGAGTCAGGG
AGAGGTTCTATGGGTAGGTTTACATCCCAACAGGATTGTAAACTCCCATCCAAT

TATAGTCACACCTCCATTTTTTTTCATTTCTTTAACATTGCATCTCAGATTCATTC
CTTCTGAAATCTACCTTATTAGTTCAGGTCTGTAGGTTGTGCTGGACTATTTCAT
AACTCTTGACTGGTCCTGGGCTCCAGTTCACCTCTTACTCCAACAATCAATTTT
CTACAGAACTATCAGAGCAATCTTCCTTTCCATCAATCTCAACTCTGTTCAAAA
ACTTTCAATAGTTTATGTGACCCTAAGAGGTAGCTGCCTCCCCACCTTTCAGCT
TGCCATTTCAAGGCTGCATAATTTGGTCCCAACCCCCTTTCCGTCCTTGACTCC
TCTCCTGCTAACAGAGACAGAACTCATTTTACTTACTCTTTTGACTGTACACCT
TTGCTTTCCACGAGGGCTCTTTCCTGGAATGATATGTCCATACAACTGTGTGTT
GTTGTTCTTGCCTGGCTCTTTCTTGGCCCACTTGTACCCAGGTTTGATCACCCC
TACTCTCCTGCCCTGGGTAACTGCTATTCAGTTATAGATCTTAGCTATCACCATC
TATATCTCTATATATAACCATCTTTAGATAATGTGTAGTATTGTCTTGAAAAAAAT
ATACATAAATGGTATACTTTGTCTCCCTCTCTGCGATTAGTTTTTTTTCACTATCA
TTTTAGTTTTAAGATTTATCCCTATTGACATAAATAGCTGGTCTTCCAATTACTGT
GTTTTTCATTTTATAAATATACCATACATTGTTACATCCATTCCTCTAAGAGGTGG
GCATTTATATTTATTTTCAAAGGTTTGCTATTACAGTGTTACAACGAATATCCTTA
GCTTAGCTGGTCTCCTTGTCTTGCCTAAATTTTACCCCACTGACCTTTCAGATC
CTGCTTTCATCCCAGCTCCTCTGGGAAAGCCTTCCCTGAATCCCCCAGCCCAC
AGCAGCAAAGTCTCTTGGTGCTGCTCAAGTTGCTTGTGCATCTTTGGTATCATC
CCTGCCAAGCACACAGCTACATAAAGTGTTACTTAAAATATTAGCCGTTGACA
AATGAATGAATGGGGCAGCACCACTCCGTGATGGCTCTATTCCTTTTGGCTTTG
GCGCCTTGGCCCACTTTCCTTCTTTGACAGCACTGGTCCCTACTCCAGAGCATT
CCTCTAGCTAGCAAAGCAGTGTGTGGATGTGTGTGGGTGTGGGTGGCGATATA
CAGACCTCACTGGATGAGGTGCCTCTGCAAGTTCACGATCCAGCCCAAACTCC
AGTGCTTGAGTTCAGATTACACCCACCCCCTCACCTCCACGCCTTCTGCACTG
GTCCAAGCCAGCGAGCCGCTGCAGCCTAGTCGAGTTAAGGCTGCTGGAGAAG
AAGGATGCACGGACAGGGCCAGTGGCTCGTAGTAGTCCGCGCGGGAGCTCGG
GCGGCACTTCTTCCTGCAGGAAACCCTGGGTTGCCCAAGGCGGCGGGGCCGA
GGCCACGCGGCAATGGGGCAGACTTGCGGTGGGAGGAGGCGGCTGAGGCGG
AAGGACACGAGGCTGCTGGCTGCACCCGAAAGTTTCAGCCAAACCGGGCGG
CGGCTGGGCGGCGGCCGAAGACGAGCTCAGGGCGCGGGGAGTCGAGGCATG

CCTGCGCTGCGGACCGTAGCGCCAGGGCCCTGAGCCTTTGAAGCCGCAGGAG

GAGGTGAGACGTCTCGGTTCCTCATGTGGATCTGCCCAGGCGGCGGCGGCGG

CGGCGGCGGCGGCGGCAGCGGCGGCAGCGGCGGCGGCAACGGCGCGGCGGC

GGCAGCGGCGGCGGCGGCAGCGGCGGCGGCGGCGGCGGCGGCGGCGGCGGC

AGCGGCGGCGGCGGCGGCGGCGGCGGCAGCGGCAGCAACGACGGCAACGGC

AGCAGCAGCAGCAGCGGCAGCAGCGGCGGCAGCAGCGGCAACGGCGATGGC

TGCAACGGCAGCAGCGGCGGCGGCGGCGGC

F5-II:4-read 1

GAAGACAGGAGGGACTGGTCTGTTAGAGGAGGAATTTATTGTGGTGTCGGCA

AAGCCTTCTTTGACTGAAGGATGAATTATGATTGTAGTTCATTAAGAGGAGGA

GGGAAGGGGACTACAGGAGTCATTCCAAGCCAAAGGAACAAAAGTATTACAG

GAGAAACAGCAAGATATGTTTGGGGGAACTTGCCAGCCAATACTGTTATTGTG

TGAAGTCTGGCTACCAGGCAGGAGGACACAGAGATGTAGTCTAATGACTTAA

AAGCAAGGCCTTGTATAAAAATTCCATGTTTAGAAGTTTGAATTTTATCCCATA

GGAGCTGTGAGAGGTTCTATGGGTAGGATTTCCCATCCCAACAACAGGATTGT

AAACTCCCACATCCAAGAATTAGTCACACCCCCATTTTTTTTTTTCTTGCTTTT

AACATTGTCTCAGATTCATTCCTTCTGAAATCTACCTTATTAGTTCAGGCTCAAT

TTGTGCCTGGACTGTTCACTCAACTCTTGACTGGTCCTAACGCAGTTCACCTCT

TTGCTCCCAACAATCAATTTTCTACAGAACTATCAGAGCAATCTTCCTTTCCAT

CAATCTCAACTCTGTCTGGGCAACTTTCAATAGTTTTATGTGACCAAGAGGTG

AAGCGTTTCACCCTTTCAGCTTAAGCATTCATATGCTGCATAATTTGTCCCAAC

CCTTCTCGTCCTTGACTCTCATTGCTAACAGAGACAGAACTCATTTATTTACTC

TTGATTGTACCTTTGTTTACGAGGGCTCTTTCCCTGAATGATATGTCCACAACTA

TTGTTGTTGTTCTTACTTTTAGCTCTTAGCCCACTTGTACCCAGGTTTGATCACA

CCCCCCACTCTCCTGCCCTGGAAGTAACTACTATTCTGGTTATAGTCTTCAATAT

CTATATCTATATCTATATATATGCATCCACAGATAATGTGTAGTATTGTCTTGAAA

AACATAAATGGTATACTTTGTCTTCATGTATTAGTTTTTTTCACTATCATTTAGT

TTTTAAGATTTTATCCCTATTGACATAAATAGCGGCGTCTTTTCATAATTACTGTG

TTGCATTTCATTTTATAAACATACCACATTATCTATCCATTCTCTAAGGGTGGCAT

TTATGTTGTTTTCTGGCTGTTTTGCTATTACAGGTGTTAGCAAACGAATATCCAG

TTTAGCTGGTCTCCTTTGTTCATGCCAAATTTTACTAATCTTTCAAGATCCTGCC
ATCCCAGCCTCTGGGAAAGCCTTCCCTGAATCCCAGCCCAGCGCAAAGTCTTG
GTACTCAGTTGCTGGCAGTGCATCTTTTGGTATCATCCCACTTGCTACACAGCG
CACACAAAAGTAAGGCACTTAAAGAAACCACGGATGAACAAATGAAGAATGA
ATGGAAGCAGCACAACGGGTGATGGCTCTTATTCCTTTTAATGGACTTTGGCG
CTCTTGGCCCAATTTCCTTCTTTTGACAGCACTGGTCCTACTCCAGAGCACATT
CTCTAGCTAGCAAAGCAGTGTGTGGATGTGTGTAGGTGGCGATACAGACCTCA
CTGATGAGATCTGCCTCTCCCTGCAAGTCTACGATCCAACCCAGTGCCAGTTC
AGAGTCCCACCCACCCCCACCCACGCTCCCTTCTGCACTGGTCGCTTTCAGCG
AGGGGGCGCAGCCCCTGATCGAGTTAAGGCTGCTGGAGAAGGATGCGGACGG
GGCCAGGACTGTAGTAGATCCTCCGCGTGCGGAGCTCGGGCCGGCGCTTTTTC
CTGCGGGAAACCCCTGGTGCCCAAGGCGGAAGCTGAGCGCCGCGGCGACAG
GGCGGGGCTTGCGGTGGAGGAGGCGGCTGAGGCGGAAGGACACACGAGGCT
GCTTCGCTGCACAATTCCGAGAAAAGTTTCAGCCAAACTTCGGGCGGCGGCC
GAGAGGCGGCGGCCGAGGAGCGGCGGACTCTGGGGCGCGGGGGAGTCGAGG
CATTTAGGTGCCTGTAAGCTTGACCGTAGCGCCAGCCTGAGCTTTGAAGCAGG
AGGAGAGTGGGGCCTCTACTGGGACCCCCATGTGGATCTGCCCAGGCGGTGG
TGGTGGTGGTGGTGGGTGGGTGGTGGTGGTGGGTGGTGGTGGTGGTGGTGGT
GGTGGTGGTGGTGGGTGGTGGTGGTGGTGGTGGGTGGGTGGGTGGTGGTGGT
GGTGGTGGTGGTGGGTGGTGGTGGTGGTGGTGGTGGTGGTGGTGTGGCGGGT
GGTGGTGGGGTGGTGGCGGCGGTGGTGGTGTGGCGGTGGCGGTGGCGGCGGT
GGCGGTGGCGGCGGCGGTGGCGGTGGCGGCGGGTGGCGGCGGCGGCGGGCG
GCGGCGGCGGCGGCGGCGGCGGCGGCGGCGGCGGCGGCGGCGGT

<u>F5-II:4-read 2</u>

GGATGGTAGATGGGGGCCCAATGACTTAAAAGGCCTTTGTATGTACTATGTTTA
GAAGTTTGAATTTTATCCTATAGAGTCAGAGAGTTCTATGGGTAGGATTTCCCA
TCCCAACAACAGGATTGTAAACCCCACATCCAATTAGTCACACCTCCATTTTTT
TTTTCTGTTTCTTTAACATTGTCTCAGATTCATTCCTTCTGAAATCTACCTTATTA
GTTCAGGTCTTCTTAAATTTGTGCCTGGACTGTTCACCAACTCTTTGACTGGTC
CTGGGGCTCCAGTTCACCTTGCTCCCAACAATCAATTTTCTACAGAACTATCAG

AGCAATCTTCCTTTCCATCAATCTCAACTCTGTTCAAAAACTTTCAATAGTTTT
ATGCACCAAGAGGTAGCTGCCTCCCCCACCCCTTCAGCTTGGCTATCTCAAGG
CTGCATAATTTGTCCCAACTCCCCTTCCGGCCCTGACTCCTCTCACTGCTAACA
GAGACAGAACTCATTTACTTACTTCTTTTGACTGTACCTTTGCTTTCACGTAGG
GCTCTTTCCTGGAATGATATGTCCATACAACTATTGTTGTTGTTCTTACTTTTAG
CTCTCTTTCTTAGCCCACTTGTACCCAGGTTTGATCACACCCCCTACTCTCCTG
CCCTGGAAGTAACTACTATTCTGGTTATAGTCTCTTTAATATCTATATCTATATCT
ATATATATGCATCCACAGATAATGTGTAGTATTGTTCAAAAAAAACATACTACAA
ATGGTATACTTTTGTCTCCTCTTATGCATTAGTTTCTTCACTATCATTAGTTTTAA
GATTTATCCCTATTGGAGTACAACAGCTATAGTCTTTTCAATTACTGTGTTGCAT
TTCATTTTATAAATATATCTATACATTATTCATCCATTCCTCTAAGGGTGGGCATTT
ATGTTGTTTTCAAGGTTTTGCTATTACAGTGTGTTACAACTAATATCCTTAGCTT
AGCTGGTCTCCTTGTCTTATACAAATTTTACTGACCTTTCAAGATCCTGGCTTAT
CCCAGCTCCTCTGGGAAAGCTCTCTTCCCTGAACTTCAGCCTAAGCGGTAAAG
TCTTTGGTACTCAAAGTTGCTTTGTGCATCTTGCATCATCCCACTGCTACACAC
AGCTACGCAGCAAGGCACTTAAGAAATATTCGCTACACGGGATGAACAAATGA
ATGAATGAATGGGGCAGCACCACCTGTGATGGCTTCTATTCCTTTTAATGGACT
TTTGGCGCCTTGGCCCATTTTCCTCTTTGACAGCACTGTCCTACTCCAGAGCAT
TCCTCTAGCTAGCAAAGCAGTGTGTGGATGTGTGGGTGTAGGTGGCGATACAG
ACTCATCCTCGGATGAGGACCGCCTCTCCCTGCAAGCTCACGATCCCGCAAAC
TCCAGTGCTTGAAGTTCAGAGAGTCCACTCCCCACCCCCTCACCTCCACGCCC
TTCTGCACTGGCTGCTTGCTGTGAGCCGCTGCAGCCCTGATCGAGTTAAGGCT
GCTGGAGAAGGATGTGATCAGCCAGTGACTCGTAGTAGATCCCTCCGCGCGGA
GCTCGCCGGCGCTTCTTCCTGCGGGAAACCCTGTGCCCAAGGCGAGGAAGGA
GGCCGCGGCGACAGGGGCGGGGCTTGCGGTGGAGGAGTAAGGCTGAGGCGG
AAGGACACACGAGGTTGCTTCTGCTGCACCTGAGAAAGTTTCGCGCCAAACT
TCGGGCGGCGGTTGAGGCGGCGGCCGAGGAGCGGCGGACTGGGGCGCGGGG
GAGTTGAGGCATTGCGCCTGTGCTTCGACCAAAGTGCCAGGGCCTGAGCTTTG
AAGCAGGAGGGAGGGGAGGAGAGAGAGGTTTCTCTATTGGACCCTCCCATGT
GGATTCGGGTTTGTGTGGCGGTGGCGGTGGCGGTGGTGGCGGGTGGTGGCGG

TGGTGGTGGTGGTGGTGGTAGTGGTGGGTGTGGTGGTGGTGGTGGTGGTGGT
GGTGGTGGTGTGGTGGTGGTGGTGGTGGTGGTGGTGGTGGTGGTGGTGTGGT
GGTGGTGGTGGTGGTGGTGGTGGTGGGTGGTGGTGGGTGGTGGTGGTGGTGG
TGGTGGGTGGTGGTGGTGGTGGTGGTGGTGGTGGTGGTGGTGGTGGTGGTGG
TGGTGGTGGTGGGTGGTGGTGGTGGTGGTGGTGGGTGGGTGGTGGTGGTGGT
GTGGTGGCGGGTGGGTGGTGGTGGTGGGTGGTGGTGTGGTGGTGGTGGTGGT

F9-II:6-read 1

GGTAATAGTCTTTGCCCATGAGGACCTCAAAACTTAATTGAGAACACAGAAAT
ACCAAAAAGTCCTGTGTGAAAAATAAGTATTGGTGTTAAGATAAAGAAAGCTG
ACAGTGGAAGACAGTGGAGGACATCTGTGGGAGGAGGAATTTATTGTGGTGT
AAGCAAAACCTTCTTTGGACTGAAGGATGAATTATGATTAGAAGTTCCATTAA
GAGGAGGAGGAAGGGGACTACAGTCATTCAAACCAAAGGAACAAAAGATAC
AGGGACAAAAACAGCAAGATATGTTTGGGGAATCTCAAATTAGCCAATACTGT
TATTGTGTAGAAGTTCTAACAGGGGCAGGAGGACAGGAGATAGGACCCCAATA
GCCAGAAAAAAGGCCTTGTATGTACCATGTTTAGAAGTTTGAATTTTATCCTAT
AGGAGTCAGGGAGGTTCTATGGGTGGGATTTTCCCATCCCAACAGGATTGCTT
AACTCCACATCAATTAGTACACCTCCATTTTTTTTGTTTCTTTAACATTGTCTC
AGATTCATTCCTTCTGAAATCTACCTTATTAGTTCAGGTCTTCTTAGAATTTGTA
CCTGGACTGTTCCACCAACTCTTGAGCTGGTCCTGGGGCTCCAGTTTACTCTG
CTCCCAACAATCAATTTTACCTACCAAGAACTATCAGGCAATCTTCCTTCCATC
AATCTCAACTCTGTTCAAAAACTTTCAATGGTTTATGTGACCCTGAAGTGTTGC
CTCCCCACCCTTTCAGCTTACCATTCAAGGCTGCATAATTTGGTCCCAACCCCC
CTTTCCGTCCTTGACTCCTCTCACTGCTAACAGACAGAACTCATTTGCCTCTTT
TGACTGTACCTTTGCTTTCACGAGGGGCTCTTTCTGGAAATGATATGTCAGCAC
AACTATGTTGTTGTTCTTATAGCTCTTTTCTTGTACTTGTACCCAATTTGATCAC
CCTACTCTCCTCACCCTGGAAATGCTACTATTCTGGTTAACAGTCTCTTATATCT
ATATCTATATCATATATACCATCCACAGATAATGTGTAGTATTGTCTTGAAAATAT
ACATAAATAAAAGGCATACTTTTATCTCTCTCCTAGATTTAGTTTTTCACTATCAT
TTGGTTTCAGATTTATCCCTATTGACATAAATAAGCTATAGTCTTTTTCAATTACT
GTGTTGCATTTTCATTTTTCAAATATACCATACATTATTCATCCATTCCTCTAAGG

GTGGGCATTTTATGTTGTTTTCAGGGTTTTGCTATTACAGTGTTACAACTTGAAT
ATCCTTAGCTTAGCTGGTCTCCTTGTCTTATACAAATTTTACTGACCTTTTCAAA
GATCCTGCTTTTCATCCAGCTCCTCTGGGAAGCCCTTCCCTAGATCCCTGACCC
ACAGCTGACAGTACTCAAAGTTGGCACGGCATCTTTGGTATCATCCTGCCAAG
CACACAGCCTACTACGCAAAGTAGGCGCAGAAATATTCCTTCACGGATGAACA
AATGAAGAAATGAATGAATGGGGCAGCACCCTTCCGTGATGGCTCTATTCCTTT
TTAATGGACTTTGGCGCCTTGGCCCACTTTCCTTCTTGACAACTGGTCCTGCTC
AGAGCATTCCTCTAGCTAACAAAGCGGTGTGTGGATGTGTGGGTGTAGGTGGC
GATACAGACCTCACTGGATGAACCGCCTTACTCTCCCTGCAGTTCGATCGGCA
GAGCTCCATTGCGCGGGGACCAGAATTCTACTTGCCCCCTACTCCACCCTTCT
GCACTGGTCAAGCCAGCGAGCCCGCTGCAGCCCACAGTAGTTGAGGCTGCTG
GGAGAAGGATGCGGACAGGGGTTGATGACTCGTGGTAGATCCCTCCGCGCGC
GGAGCTCGGGCAGCGCTTCTTCTGGGAAGCCCCTAGGTGCCCAAAGCGGCGG
GGCCGAGGCCGCGGCATTTCAATCAGGGCAGGGCTTTGCGGTGGGAGGGAGG
CGGCTGAGCGGAAGGACACACAGGGCTGCTTCGCTGCACACCGGTTCAAAGC
CAGAGCTTCAGGCGGCAGCTGAGGCGGCAGCCAGGGGCGTGACTCCAGGGC
GCAGGAGTCGGGCATTGCGCCTGTGCTTCGGACCGTAGCGCCAGGGCTAGGC
CTTTGAAGGCAGGAGGAGGAGAATTAGGGCTCCTATCGGGACCCCCCTCCCAT
GGATCTGCCCAGGCGGCGGCGGCGGCGGCGGCAGCGGCAGCAGCAGCAGCA
GCGGCGGCGGCACGGCAGCAACAGCAGCAACGGCTTGGCGAAGCGGCGGCG
GCAGCGGCGGCGGCGGCGGCGGCGGCGGCGGCGGCGACGGCAACAACGGCA
GCAGCAGCAGCGGCGGCGGCGGCGGCGGCGGCAGCAGCGGCGGCAGCAGCA
ACGGCTGGCAACGGCAACAGCAGCAGCAGCAGCGGCAGCGGCGGCGGCAGC
GGCGGCGGCAGCGGCAGCAGCGGCAGCGGCAGCGGCGGCAGCGGCGGCAGC
GGCAACGGCGGCAGCTGGCAGCGGCGGCAGCGGCGGCAGAGGAAGGCGGC

F9-II:6-read 2

GGCCTTTGTACTATGTTTAAGTTTGAATTTATCCTATGAGTCAGGGAGGTTCTAT
GGGTAGGATTCCCATCCCAACAGGATTGTAAGCTCCCACATCAATTAGTCACAC
CTCCATTTTTTTTCTGTTTCTTTAACATTGTCTCAAGGTTGTTCCTTCTAATCTAC
CTTATTAGTTCAGGTCTTCTTAAATTTGTGCCTGGACTGTTCACCAACCTCTTG

ACGGTCCTGGGAGCCAGTTTCACCTCTTGCTCCCAACAATCCAATTTTCTACA
GAACTATCAGAGCAATCTTCTTTCCATCAATCTCAACTCTGTTCAAAACTTTCA
ATAGTTTATATGACCCTAAGAGGTAGCTGCCTCCCCACCCCTTTCAGCTTGCCA
TTCAGGCTGCATGTAATTTGGTCCCAACCCCCTTTCCGTCCTTGACTCCTCTCA
CTGCTAACCAGAGACAAGAACTCATTGCCTCTTTTGACTGTACCTTTGCTGCTT
TCTGAGGCTCTTTCCTGGAATGATATGTCACGCTGTGTTGTTGTTCACTGGCTC
TTTTACTTAGCCCACTTGCCCAGGTTTGATCACACCCCTACTCTCTCCTGCCCT
GGAAGTAACTACTATTCTAGTTTCAGTCTCTTAATATCTATATCTATGTCTATATAT
ATGCATCTACAGATAATGTGTAGTATTGTCTTAGAAAAAAATATACATAAATGGT
ATACTGTCTCCTGCGATTAGTTTTTTTCACTGTATTTGTAGTTTTTAGATTTATCC
CTGACATAATGTGCAAATTACTTTTTCCATTACTGTTGCATTTCATTTTATAAATA
TACCATACATTATTCATCCATTCTACTCTAAGGGTGGGCATTTATGTTGTTTTCA
GGTTTTATTACAGTGTTATGAATATCCTTAGCTTAAGCTGGTCTCCTTGTCTTATA
CAAATTTTACTGACCTTTCAGATCCTGCTTCATCCAGCTCCTCTGGGGCTTCCC
TAAATCCCCAAACACAGCAGCAAAGTCTTGGTACTCAAAGTTGCTTGCATCTT
TGGTATCATCCACTGCAAGCACACAACTCACATAAAGTAAGGCACTTAAATATT
ATACGGATGAACAAATGAATGAATGAATGGGCAGCACCACTCCGTGATGACCA
TTGCTTTAATGGACTGGCGCCTTGGCCCACTTTCCCCTTGCGACTGGCAGCAC
TGGTCCTACTCCAGAGCAGCCCTCTAGCTAGCAAAGCAGTGTGTGGATGTGTG
GGTGTAGGTGGCGATACAGACCTCACTGGATGAGGACCCACTTACTCTCCCTG
CAAGTTCGATCAGCAGCAGAGAGCTCAGTGCTTGAAGTTCAGGTGCACCCCT
GCCTCCACGCCCCTTCTGCACTGGTCAAGCCAGCAAACCGCTGCAGCCCTGAT
CGAGAGTTAAGGCTGCTGGAGAAGGATGCGGACAGGGCCAGTGACTCAGTAG
TGAATCCCTCCGCGCCCAGACTCGGGCCGGCACTTCTTCCTGCAGGGAAACCC
TGGGTGCCCCAAGGCAGGCAGGGGCCAGGCCGCGGCGACAGTGGGGCGGGA
GCTTGCAGTGGGAGGGAGGCGGCTGAGGCCCGGAAGGACCACAGGCTGCTT
CGCTGCACACCCGAGAAAGTTTCAACCAAACTTCGGGCGGCTGGGCGGCGGC
CGAGGAGCGGCGCTTTCAGACTGGGGGCACGCAGGGGAGTCAAGGCATTGC
GCCTATGCCTTCGGACCGTAAGCGCCAGGCCTGAGCCTTTGAAGCAGGGAGG
AGGGAGGAGAGTGGGGCTCCTCTATCAGGACCCCTCCCATGTGGATCTGCCCA

GGCGGCGGCGGCGGCGGCGGCGGCGGCAGCAGCAGCGGCGGCGGCGGCGGC
AACGGCGCGGCAGCGGCGGCGGCGGCGGCGGCGGCGGCAGCAGCGGCGGCG
GCGGCGGCGGCGGCGGCGGCGGCGGCGGCGGCGGCGGCGGCGGCGGCGGCG
GCGGCAGCGGCAGCGGCGGCGGCGGCGGCGGCAGCAGCGGCGGCGGCGGCG
GCAACGGCAGCAGCGGCGGCGGCGGCCCGGCGGCGGCGGCAACGGCGGCGC
GGCAGCGGCGGCGGCGGCGGCGGCGGCAGCGGCAGCGGCGGCGGCAGCGGC
GGCAGCGGCGGCAGCGGCGGCGGCGGCGGCGGCGGCGGCGGCAACGGCAGC
GGCGGCGGCAGCGGCAACGGCGGCAGCAGAAGAGGCAGC

F9-II:6-read 3

GGATTCAGAGGATGAATTATGATTAAGTTCATTAAGAGGGAGGAGGAAGGGGA
CTACGAGTCATTCCAAGCCGAACAAAAGATACAGGGACAGAAACAGCAAGAT
ATGTTTGGGGGAATCTCAAGTTATGGCCAATACTATTGTGTGTGTGAAGTTCAC
AACAGAACATAGGAGGACAGGAGATACAGAGACCCAATGGCGAAAAGGCTG
TATGTACCATGTTTGATTTGAATTTTTATCTATGGAGTCGGGAGAGAGGTTCTAT
GGGTAGGATTCCCATCCAACAGGTTGTAAACTCCCACATCCAATTAGTCACAC
CTCCATTTTTTTTTCTGTTTTCTTTAACATTGTCTCAATTCATTCTTTAATCTACC
TTATGGATTTCAGGTCTTCTTTAAATTTGTTACCTGGACTGTTCTGCCTTTCCAG
CTAGTCACAAACCAGTTTCTTTCCCTCTTGCTCTAACAATCAATTTTTCTACAG
AACTATCAGAACAATCTTCCTTTCCATCAATCTCTCGCTCTGTTCAAAGCTTTC
AATAGTTTTATGTGACCCTAAAATTTAGCTGCCTCCCCACCTTTCAGCTTGCTG
CCATTCAAGCTGTAATTTAGTGCAACCTTTTACCATGTAGCTCTCTCTCTTAACA
GAGACAGAGACTCATTTACTTTTACTCACGGCTGTACCTTTGCTTTCAGGGCTC
TTTCTGGAATGATATGTCCATAACTATTGTTGTTTATTTCTTACTTTTAGCTCTTT
TCTTAGCCACTTGTACCCAGGTTTGATCCACCCCTACTCTCCTGCCTGGAAGTA
ACTACTATTCTGACATAGTCTTAATATCTATATCTATATCTATATATGCATCCACAG
ATAAATGTGTGATATTGTCTTGAAAAAATATACATAAATGGTATGCTTTGTCTCC
CTCTCGTTAGTTTTTCACTTTATCATTTGGTTTTAAGTTTATCTAACAATATAAAT
AATATAGTCTTTCAATTACTGTGTTACATTTCATTTTTATAAATACCATACATTATT
CATCCATTCCTCTAAGTGTGACATTTATGTTGTTTCAGGTTTGCTATTACAATTAT
TTGTGAATATAGCTTAAGCGGTCTCATGTCTTGCTCTAAATTTACTAGCCTTTCA

GGGTCCTGTTTCATCCAGCTCCTCTGGGAAAGCCTTCCTGAATCCCCCAGCCC
ACAGCAAAGTCTTGGTACTCAAAGTTTAGCATTGCATCTTTGGTATCATCCACC
AGACACAGCTACATAAGTAAGGGCATGAAATACCTTCGCTACACAGATGAACA
ATGAATAGATGAGTGAATAGCACCACTCCATGGTGGCTCTATTCCTTTTTACCT
GTGTGGACTTTGGCACCTTATTTTTCCTTCTTGTGTACTGAATTCTCAGAGCATT
CTCTAGCTATAGAAGCAGATGTGTGGATGTGTGGGGTGCAGGGGTGGCAGTGA
ACCTCACTGGATGAGGACCGCGCCTCTCCCTACACAAGTTCAGTCAGCAAACT
CCAGTGCTTGATTTGAAGTCCCTGTACCCCCTCACTGCGCCCCTTCTGCACTGG
TCAAGCCAGCAGGCCGCTGCAGCCCTGATCGAGTTAAGGCTGCTGGAGGAAG
GATGCCAGACAGGGCCAGTGACTCGTAGTGAATCCCTCCGCGCAGGCCAGGC
CAGCGCTTCTTCCTGGGGAAACTGAAATTTTGGGCGGCGGGGCCAAAAGGCC
CTTGGCGACAATGAACGAACACACGCACGTTGGGAGGAGCGGCTGGGAGCG
GAAGAACACACAGACTGCTTCGCGCTGCACCCGAAAGTTCAATAATGAAACT
GGGCGGCGGCTGGGGGCGGCGGCGAGGCAGCAGGCTCCGGGGCGCGGGGAG
TCGAGGGCATTTGCGCCTGTGCTTCGGACCGTGGCGCGCCAGGGCTGAGCCTT
TATGAAGCAGGAGGAGGGAGGAGAGTCGGGCTCCTCTATCGGGACCCCTCCC
GTATGGATCTGCCGGGCGGCGGCGGCGGCGCGGCGGCGGCGGCGGCAGC
GGCGGCGGCGGCGGCGGCGGCAACAACGGCAGCAGCAGCGGCGGCGGCGGC
GCGGCGGCAGCGGCAGCGGCAGCGGCAGCAGCGGCAGCGGCAGCGGCGGCA
ACGGCAATGGCACAGCAGCAGCAGCAGCGGCAGCGGCGGCGGCGGCAGCGG
CGGCGGCGGCGCGGCGGCAGCGGCGGCGGCGGCGGCGGCGGCAGCGGCAGC
GGCGGCGGCGAAGCGGCGAAGCGGCAGCAGCGGCAGCGGCAGCGGCAGCG
GCAGCAGCGGCGGCGGCAGCAGCGGCGGCGCGTGGCGGCTGGCGGCAGCGG
CGGCAGCGGCGGCGGCAGCGGCGGCAGCGGCGGCGGCGGCGGCGGCGGAGG
AAGGCGGC

<u>F9-II:6-read 4</u>

GGGAGGAAGGGACTGAGGTCATTCCAAGCAAAGGAACAAGATACAGGGACA
GAAACAAGATATGTTTGGGGAATCTCAAGTTGGCAATACAACTGACTTGTGTG
AAGTTCACAACAAGGCAGGGAGGATGGGAGCCCAATGAAGGCTTATGTGTAC
CATGTTTGAGAAGTTTGAATTTATCCTGAGTCAGGAAGGGATTCTATGGGTGG

GGTTTTCATTAACAGGATTGTAAACTCCCACATCCAATTAAATTACACCTCCATT
TTTCTGTTTCTTTAAGCAACCTTCTCAGATTCATTCCTTCAAATCTATGTTGATA
GTTCAAGTCTTCTTAAATTTGTACAGATGTTCTGGCTCCCAGCTAGTCCTGGGC
TCAGTTGCCCTCTTGCTCCCAACAGTGAATTTTCCTACAGACTTCTCAGAGCA
ATCTTCTTTTATCAAATATCAACTCTGTTCAAAAACTTTTAATTAGTTTATTCTTG
TTGTTCAAGGGTAGCTGCCTCACCCTTTGTGCCATTCAGGCTGTAATTTGGTCC
CCAACCCCTTTCAAGTCCTTGGCTCCTACCATACTAACAGAGACAGAACTCAT
TGCCTCTTTTGACTGTACCTTTGCTTTCACAGGGCTCTTTCCTGAGATAATTATC
CATAAGCTATTGTTGTTGTTCTTACTTTAGCTCTTTTCTTGCACTTGCAGTTTGA
TCACCCCCTACTCTCACACCCCTGGGAAGTAACTTTTCTTCTAATTTATAGTCTC
TACCATGTCTATCCTCATATATATATGCATCCCACAGATAATGTGTAGTATTGTCT
TGAAAAAATATACATAAATGGTATACTTCCTTCTCCCTCTACGATTAGTTTTTCC
TATCATTTAGTTTTAGATTTATCCTATTGACATAAATGCTTTCTAGTCTTTTCAAT
TTTATTGCATTCATTTTATAAATACTACCATTATTCATCCATTCCTTAAGGGTGAG
CATTTATGTTATTTTCAGGGTTTACTATTACAGTGTTGATGAATATCCCTTAAGCA
GCTGTCTCTAACCTCATATACAAATTTTACCCCAGCCTTTCAAGATCCTGCTTTC
TGTGCCCAGCTCCTCTGAGAAGCCTTTACTGGTCCCAGCCCACAGCAGCAAGT
CTTGGTACTCTACAGTTGTGTCTTTGGTGCTCGTCACTGTAAACACCACAGCTA
TAAAGTAAGGCATGAAATATTCATGGATGAACAATGAATGAATAGACCAGCTTC
CTTCCGTAATTAGCTCTATTCCTTTTTAATGGACTTTGGCCACTTTTCCTTCTTG
ACAGCATAATTACCTCCAGGCATTCCTCTAGCTATGCCGTGATGCCGGATGTGT
GGGTGGGTGGCGATACAGACCTCACTGGATGAGGGACCGCCTCTCCCCTGCA
GTTCACGATCCCGGCGAAGCTCAGGTGCTTGAAGTTCAGGTCCCTACCCCCCC
CTGCCTCCACGCCCTTCTGCATAGTGAGCCAGCGAGCCGCTGCAGCCCTGATC
GAGTTAAGGCTGCTGGAAGGATGCGGACAGGGCAATGATGACTCGTGGTAAG
ATCCCTCGCGCGGGCCTCGGGCCAGCGCTTCTTCTGCGGGAAACCCCTGGGTG
CCCAGGCGCAGCAGGGCCGGGCCGCGGCGACAGTAAAGACAGAGGCCCTGC
GGTGGGAGGGAGGCGGCTGGGCGAGAGGACACACGAGGGCTGCGCTGCACC
AGAAAGTTTCGTATTAAACTTCAGGCGGCGGCTGAGGCGGCAGCCGAGGCCA
CAACAGACTCAGGAGCGCAGGAGTCAGGGCATTGCGCCTGTGGCTGGACCAT

GGCGCAGGGCCTGAGCCGCTGGAAGCAGGAGGAGGGAGAAGTGGGCTCCTG
GGACCCCCCTCCCCATGTGGTCTGCCCAGGCGACGCTGGCGGCGGCAGCAGC
GGCGGCAGCGGCGGCGGCAGCAGCAACGGCAACGCGGCAGCGGCGGCGGCG
GCGGCAGCGGCAGCAGCGGCCAGCAGCAGCAGCAGCAGCAACAGCAGCAAC
AGCAACAACAACAGCAACGGCAGCAGCAGCAGCAGCAGCAGCAGCGGCAGC
AGCAGCAGCAGCAGCAGCAGCGGCAGCAGCCCAGCAGCGGCAGCAACAACA
ACAGCAACAACAGCGCGGCAACAGCAGCAGCGGCAGCAGCAGCAGCAGCAA
GCAGCAACAGCAGCGCTGGCGGCAGCAGCAGCAACAACAGCAGCAGCAGCA
GCAACAGCAGCAACAGCAACAGCAACAACGGCAACAGCAGCAGCAGCAGCA
GCAGCAGCAGCAGCAGCAGCAGCAGCAGCAGCGGCAGCAGCAGCAGCAACA
GCAACAGCAGCAACAGCAACGGCGGC

F9-II:6-read 5

GGAGACGGTGGAGGGACTGTCTGTTGGGGAATTTATTGTGTGTCGGCAAAAG
CCTTCTTTGGACTGAAGGATGGTGCCAGTTAGAAGTTCATTAGAGGAGGAGGA
AGGGACTGAGTCATTCCAAGCCAAAGACAAAAGATACGGGACAGAAACAGC
AAGATATGTTGGGGAATCTGGGATTAGCTAATACTGTTATTGTGCTGAAGTTTC
ACAACAGGGGCAGGGAGGACAGGAGATGGGGCCCCAATGTGAAAAAGGCCT
TGTATGTACCATGTTTAGAAGTTTGAATTTTATCCTATAGGGAGTCAGGAGAGG
TTCTATGGGTGGGGTTCCCATCCCAACAGGATTGTAAACTCCCACATCAATTAG
TCACACCTCCATTTTTTTTCTGTTTCTTTTAACATTGTCTCAATTCATTCCTTCTA
GAAATCTACCTTATTAGTTCAGGTCTTCTTAAATTTGTGCTGGACTGTTCACAA
CTCTTGACTGGTCCTGGGGCTCCAGTTCACCTCTTGCTCCAACAATCAATTTTC
TAGAACTATCAGAGCAATCTTCCTTTCCATCAATCTCAACTCATTTCAAAACTT
TCCAATAGTTTTATGTGACACAAGGGTAGCTGCCTCCCCACCCTTTCAGCTTGC
CATTCAAGGCTGCATAATTTGGTCCCAACCCCCTTTCAGGTCGCGGCTCCTCAC
TGCTAACAGAGACAGAACTCATTTACTTACTCTTGGCTGTACCTTTGCTTTCAC
GAGAATGCTTTCCTGGAATGATATCCACTACAATATTGTTGTTGTTCTTACTTTT
TAGCTCTTTTCTTAGCCCACTTGTACCCAGGTTTGATCCACACCCCCTACTCAC
TGCCCTGGAAGTAGCTGCTATTCTGATTTATAGTCTCTTAATATCTATATCTATAT
CTATATATATACCGTCACAGATAATGTGTGGTATTGTCGAAAAAATATACATAAA

TGGTATGCTTTGTCTCCCTCTACGATTAGTTTTCACTGTCATTTAGTTTTAGAT
TTATCCCTATTGACATAAATAGCTATAGTCTTTTCAATTACTGTGTTGCATTTCAT
TTTAACAAATATACCATACATTATTCATCCATTCCTCTAAGATTTGGGCATTTATG
TTGTTTTCAGGTTTTGCTATTACAGTGTTACCTTGAATATCCTTGGCTTAGCTGG
TCTCTTCTTGTCTTATACAAATTTTTACTGACCTTTCAAGATCCTGCTTTCATCC
AGCTCCTCTGGGAAAGCCTTCCTGAATCCCCCCAGCCCACAGGCGGCAAAGT
CTTGGTACTCAAGTTTAGCAGCCATCTTTAAGGTATCATCCCACTGCCAAACAC
ACAACTCATAAGCCAGGCACTTAAGAAATATTCCTTTGCGGATGAACAAATGA
ATGAATGAATGGGGCGCAGCACCACTCCGTGATGGCTCTGTTCTTTTTAATGGA
CTTTGGCGCCATGGCCCACTTTCCTTCTTGACAGCACTGGTCCCTGCTTAGAGC
ATTCCTCTAGCTAACAAAGCAGTGTGTGGATGTGTGGGTGTGGGTGGCGATAC
AGACCTCACTGGATGAGGACCGCCTCTCCCTGCAAGTTCACAGTCGGCAAAC
TCCAGTGCTTGAAGTTCAGTCCTACCCACCCCCCTCACCTCACGCCCCACTGC
ACTGGTCAAGCCAGCAGGCCGCTACAGCCTGATCAGGTTAAGGCTGCTGGAG
AGAAGGATGCGGACGGGAGCCAGTGACTCGTAGTGAATCCCCTCCGCGCGGA
GCTGGCCGGCGCTTCTTCCTGCGGGAAACCCCTGGGTGCCCAAGGCGGGGCC
AGGCGGCGCGACAGTGGGGCAGGGCTTGCGGTGGGAGGGAGGCGGCTGAGG
CGGAAGGACACACGAGGGCTGCTTCGCTGCACGCGAAAGTTTCAGCCAAACT
TCGGGCGGCAGCTGGAGCGGCAGCCGAGGCAACAGACTCAGGGCGCAGGGA
GTCGAGCATTTTACTTTTATGCTTCGGACCGTAGCGCCAGGGCCTGAGCGCAC
TGAAGCAGGAGGAGGAGGTGGGGCTCCTCTATCCGGACCCCTCCCATGTGGAT
CTGCCCAGGCGGCGGCGGCGGCAGCAGCGGCGGCAGCGGCGGCGGCAG
CGGCGGCGGCGGCGGCGGCGGCGGCAGCAACGGCAACGGCAGCAGCAGCAG
CGGCGGCGGCGGCGCGGCGGCGGCGGCGGCGGCGGCGGCGCGGCGGCGGCG
GCGGCGGCGGCAGCGGCGGCGGCAGCAGCTGACTACGGCAACGGCAGCGGC
GGCGGCAGCGGCGGCGGCGGCGGCGGCGCTGGCAGCAGCAGCGGCGGCAAC
GGCGGCGGCAGCAGCAGCAGCAGCGGCGGCGGCGGCGGCGGCGGCGGCGGC
AGCAGCGGCAACGGCGGCGGCGGCGGCAGCAGCAGCCGGCGCGGCGGAGGA
GGCGGC

F9-II:6-read 6

GGGGTCATTTCGAAGCCAAAGGAACAAAAGATACAGGGACAGAAACAGCAA
GATATGTTTGAGGTCTCAAGTTGGCCATCACTGTTGTTGGCCTTGAGAAGTTCT
ACAACAGGGGCAGGGAGGACAGAGATGGGGCCCAATGACGAAAAAGAGCCT
TGTATGTACCATGTTTAAAATTTTATCCGAGTCAGGGAGTTTATGGTGGGTTTAT
CCCAACAGAGTTATATTATGACTCCCATCCATTAGTCACACCTCCATTTTTTTTC
TGTTTCTTTTAACATTGTCTCAGATTCATTCCTTCAGGAAATCTACCTTATTCGT
TTCAGGAGTCTTCTAGCAAGTTGTGCCTGGACTGTTCACCCAGCCTCTTGTTG
ACTGGTCCTGGGGCTCCAGTTCACCTCTTGCTCCCAACAATCAATTTTTCTACA
GAACTATCAGAGCAATCTTCCTTTCCATCAATCTCAACTCTGTTTAAAAAACTT
TCAATATTTTATCATGACCTAAGTAGCTGCCTCCCCACCCTTTCAGCTTGCCATT
CAGAGGCTGCATAATTTGGTCCAGCACCGTCCTTGACTCCTCTCACTGCTAAC
AGAGACAGAACTCATTTACCTGCTGACTGCCTTTACTTTCCTGGGCTGCTGGA
ATGATAGCATCCATACAACTATTGTTGTTGTTCTTACTTTTAGCTCTTTTCTTAGC
CCACTTGTACCCCGGTTTGATCACCCCTACTCTCTGCCTGGGAAGTAACTACTA
TTCTGTTATAGTCTTAATATCTATATATATATATATCTATATATATGCATCCACAGAT
AATGTGTAGTATTATCTTGAAAAATATACATAAATGGTATACTTTGTCTCCCTCTC
GATTAGTTTTTCACTATCATTTAGTTTTTAAGATTTATCTATTGACATAAATAGCT
ATGGTCTTTTCAATTACTGTGTTGCATTTCATTTATAAATATACCATACATTGTTA
CTAAATCCATTCCTCTAAGGGTGAGCATTTATGTTTTCAAGGTTTTTATTACACA
GTGTTACTTGAATATCATATATGCTCACTTGTCTTATACAAATTTGCTGACCTTTC
AAGATCCTCACTTTCATCCCAGCTCCTCTGGGAAAGCCTTCCCTGAATCCCAG
CCCACAGCGGCAAAGTCTTGGCCTCAAGTTGCTTGTGCCATCTTGGTATCATCC
CACTGCACCAAGCACACAACTACATAAAGTAAGGCACTTAAAATATTAAGCTA
CACGGATGAACAAATGAATGATGAATGAATAGGGCAGCACCACTTGTGATGGC
TATTCCTTTTTGAACTTTGGCGCCTTAGCCCCACTTTCTGCTTCTTTGACAGCA
CTGAATCCTACTCCAGAGCATTCCTCCTTCTTGTAAAACAGTGTGTGAGTGTG
GCGGGTGTGGTGGCGATACAGACCTCACTGGATGAGGACCGCCTCTCTCCCTG
CAAGTTCGGTGACAAACTCCAGTGCTTGAAGTTCAGAGTCCTACCCACCCCCT
CACCTCCACGCCCCTTCACCAGGTCAAGCCAGCGAGCCGCCTGCAGCCCTGA
TCCCAGGTTAGTACTGGAGAAGGATGCGGACAGCCTCCTGCAGGAAGCCTGG

GTGCCAAGGCAGCGAACCCGGGCCGCGAGCAGTGGGGCAGGGCTTGCAGTG
GGAGGAGGCGGCTGAGGCAGGAAGGACACGGCTGCTTCGCTGCACACCCGA
AAGTTCAATGCAGCTTCGGGCGGCGGCGCTGGGCGCGGCCGAGGCAGCAGAC
TCAGGGCGCAGAGAGTCAAGGCGTTGCGCCTGTACTTCGGACCGTAGCGCGG
TTCAGGCGCTGGCAGGAGGGAGGGAGAGAGTGAATCCTCTATCAGGACCCCC
CCTCCCCATGTGGATCTGCCAGGCGGCGGCGGCACGGCGGCGGCAGCGGCGG
CAGCGGCGGCGGCGGCAGCAGCAGCAGCGGCGGCGGCGGCGGCGGCGGCGG
CGGCGGCGGCGGCGGCAGCTGGCGGCAACGGCGCGGCGCAGCAACGGCGGC
AGCAGCGGCGGCGGCGGCGGCGGCGGCGGCAACGGCAGCGGCGGCAGCGGC
GGCAGCAGCGGCGGCACGGCAACGGCGGCAACGGCGGCGGCGGCGGCGGCG
CGGCGCGGCGCAACGGCGGCAACGGCAGCGGCAGCAGCGGCAGCGGCGGCG
GCGACGCGGCGAAGCGGCGGCGGCGGCAGCAGCTGGCGGCAGCGGCGGCGC
GGCGGCGGCGGCGGCGGCGGCGGCGGCGGTAACGGCAGAGGAGCCGGC

F9-II:6-read 7

GCCAAAGGAACAAGATACAGGGACAGAAAACAGATATGTTTGGGGGAATTCC
CTGGTTAGCCAATACTGTTATTGTGAAGTTCACAACAGGGGCAGGGAGGATAA
GAGATAGCTCAATGACTAAAAAGGCCTTGTATGTACCATGTTTAGAAGTTTGAA
TTTATCCTATAGGAGTCAGGGAAGAGGTTCTATGGGTAGGATTTCATCCCAACA
GGATTGTAAACCCCCACATCCAATTAGTCACACCTCCATTTTTTTTTTCTGTTT
CTTAACATTGTCTCAGATTCATTCCTTCGAAATCTACCTTTATTAGTTCAGGTCT
TAAATTTGTGCCTGACTGTTCACCAACTCTGGATTGGTCCTGGGGCTCCAGTTC
ACCCTTGCTCCCCAACAATCAATTTTCTACAGAACTATCAGAGCAATCTTCCTT
CCATCAACAATCTCAACTCTGTCTGCAAAAAATCTCAATAGTTTTATGTGACCT
AAAGAGGTATTGCCTCTAATCTTTCAGCTTGCCATCTGTTGCATACAATTTGTCC
CAACTCTTCCGGCCCTTGACTCCTCTCACTGCTAACAGAGACAGAACTCATTT
ACTTACTCTTTGACTGTACCTTGCTTTCACGAGGGCTCTTCCTGGAATGATATG
TCCATACAACTATTGTTGTTGTTCTTACTTTTTAGCTCTTTTCTTAGCCCACTTGT
ACCCAGGTTTGATCACACCCCTACCTCCTGCCCTGGAAGTAACTACTATTCTGG
TTATAGTCTCTAATATCTATATCTATATCTATATATATATCCACAGATAATGTGTAGT
ATTGTTCTTGAAAAATATACATAAATGGTATACTTGTCTCTGCGATTAGTTTTCA

CTATCATTAGTTTTTAAGATTGTCCTATTGACATAAATAGTTATAGTCTTTTTCAA
TTATTGTGCCAGACATTTCATATTTATAAATATACCATACATTATTCATCCATTCCT
CTAAGGGTGGGCATTTGTGTTGCTTTCAAGGTTTGCTATTACAGTGTTACAACT
AATATCCTTAGCTTAGCTGGTCTCCTTGTCTTATACAAATTTTATTGACCTTTCA
AGATCCTGCTTTATCCCAGCTCCTGGGAAAGCTCTTCCTGAATCCCCCAGCCCA
CAGCGGTAAAGTCTGGTACTCAAAGTTGCTTTGCATCTTTTGGTATCATCCCAC
TGCCAAGCACACAGCTACGCAAAGTAAGGCACTTAAGAAATATTCGCTACACG
ATGAACAAATGAATGAATGAATGGGGCAAAAAGCACCACCTGTGATGCCTTAT
CTCCTTTTAATGGACTTGGCGTGCCTTGGCCCACTTTTCCTTCTTTTGAATAGC
ACTGGTCCTACTCAGGAGCATTCCTCTAGCTAGCAAAGCAGTGTGTGATGTGG
GTGTAGGTGGTGACACAGATCTCACTGGATGAGACCGCCTCCCTGCAAGTTCA
CGATCCTGGCAACCCAGTGCTTGAAGTTCAGGGAGTCCTACCCCACCCCTCAC
CTCCACGCCCTTCTGCACTGGTCAAGCCAGCGGGAGCTGCAGCCCTGATCGA
GTTAAGGCTGCTGGAGAAGCATGCGGACGGGCCAGTGGACTGTAGTAGATCC
CTCCGCGCGGGAGCCTGGGCCGGCGCTTCTTCCTGCGGGAAACCCCTGGTGC
CTGCAAGGCGGTGGGGGAAGGGGAGGCCGCGGCGACAGTGGGGCGGGGCTT
GCGGTGGGAGTGAGGCGGCTGAGGAAGGACACACGAGGTTGCTTGCTGCAC
ACCTGAGAAAGTTTCAGCTAAACTTCGGGCGGCATGAGGCGGCGGCCGAGGA
GCGGCGGACTCTGGGCGCGTGGGGAGTCGAGGCATTTGCGCCTGTGCTGACC
AGCGCTAGGGCCTGTTTTGAAGCAGGAGGAGGGAGGAGAGAGTGGGGTTTTA
TCGGGACTCCCCTGGCGATTTACCAGGCGGTAAGGGTGGTGGTGGCGGTGGT
GGCGGTGGCGGTGGTGGTGGTGGCGGTGGTGGCGGTGGTGGTGGCGGTGGCG
GTGGTGGCGGTGGCGGGTGGGTGGTGGCGGCGGGTGGCGGTGGTGGTGGTG
GTGGTGGCGGTGGTGGTGGCGGCGGGTGGTGGCGGCGGTGGCGGTGGTGGTG
GTGGCGGCGGCGGCGGTGGTGGCGGGTGGCGGCGGCGGCGGCGGCGGCGGG
TGGTGCGGCGGCGGCGGCGGTGGCGGCGGTGGCGGCGGCGGCGGCGTGGCG
GTGGCGGCGGCGGTGGCGGCGGCGGCGGCGGCGGCGGCGGCGGCGGCGGAG
GAGGCGGC

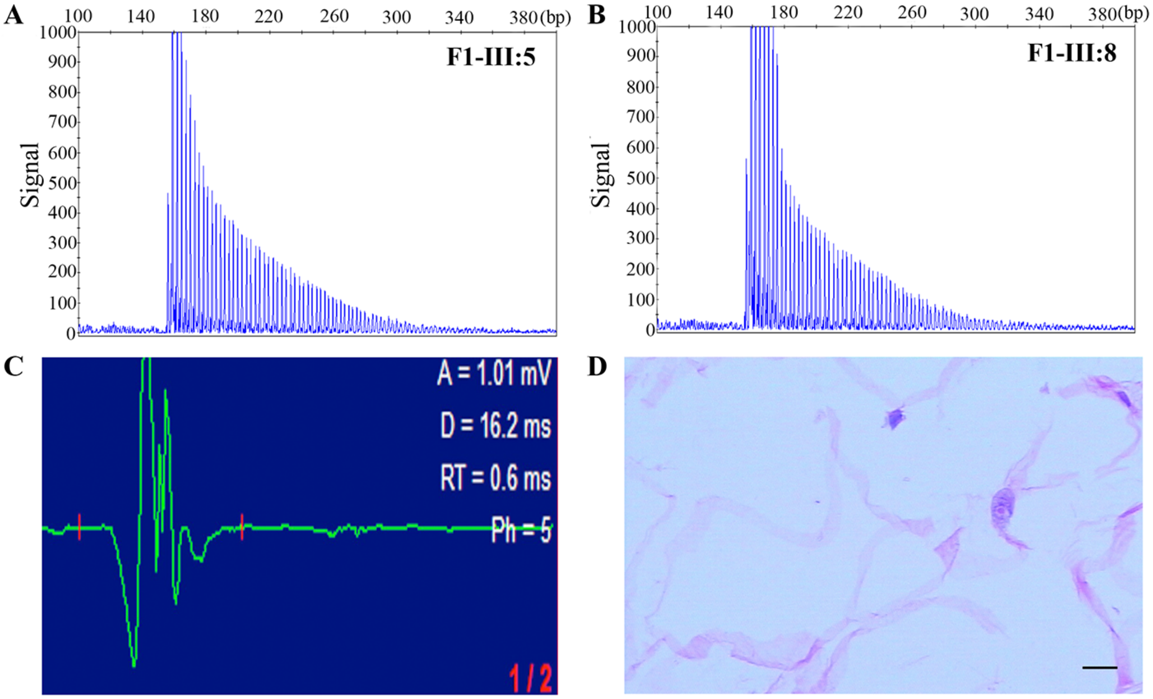

Figure S2. Additional Molecular and Clinical Diagnosis of Family 1. (A-B) RP-PCR assay and GC-PCR assay showed abnormal repeat expansion in subject F1-III:5 **(A)** and F1-III:8 **(B). (C)** Representative motor unit potential (MUP) of right tibialis anterior from electromyogram of subject F1-III:8 indicated neurogenic impairment. **(D)** Skin biopsy sample of subject F1-III:8 displayed typical eosinophilic intranuclear inclusions. Scale bars, 10μm.

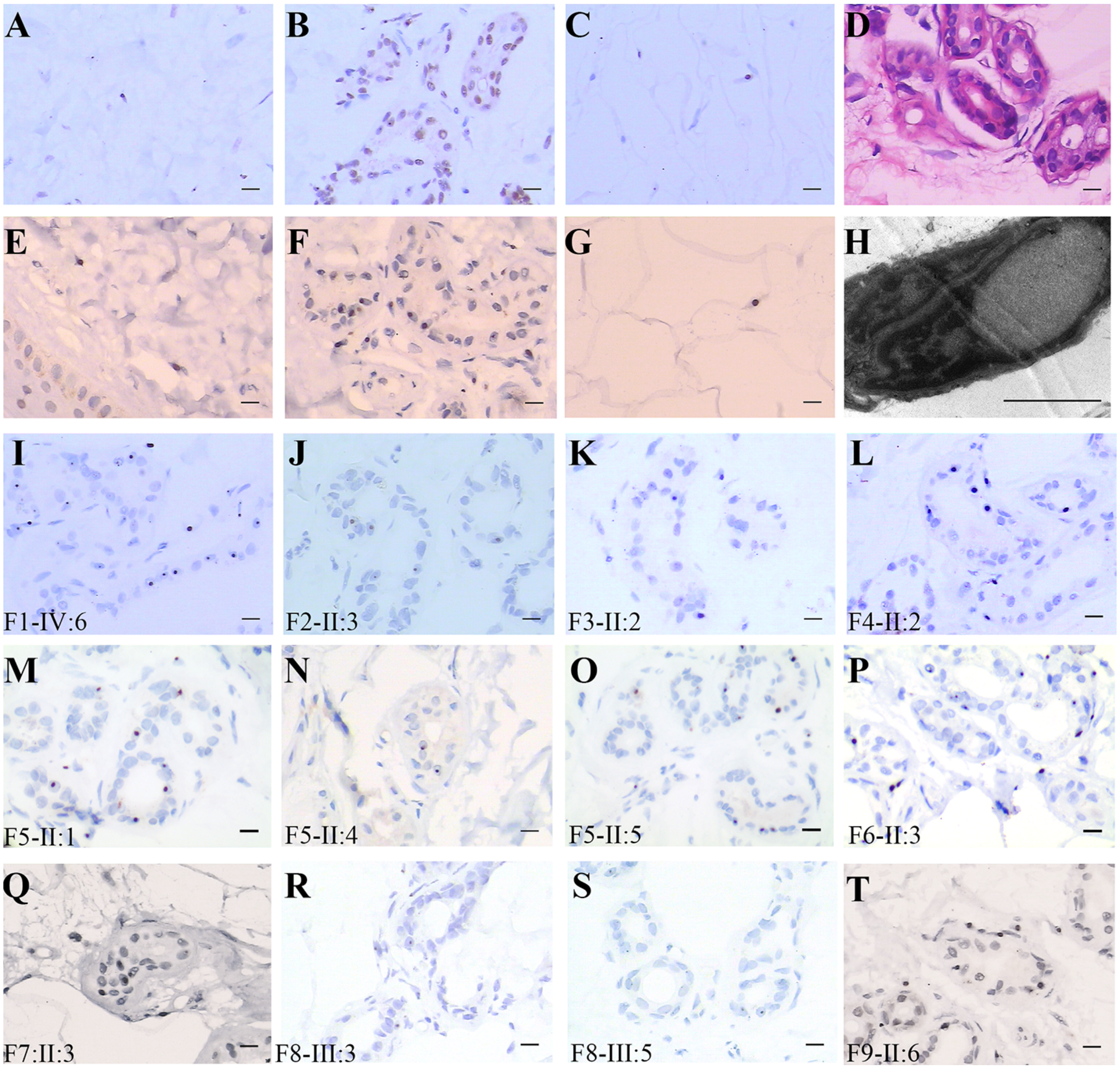

Figure S3. Histopathological Features of NIID. (A-C) Representative skin biopsy samples showing ubiquitin-positive intranuclear inclusions in fibroblasts **(A)**, sweat gland cells **(B)**, and adipocytes **(C)**. Scale bars, 10μm. **(D)** Representative skin biopsy samples revealed eosinophilic intranuclear inclusions in sweat gland cells. Scale bars, 10μm. **(E-G)** Representative skin biopsy samples indicated p62-positive intranuclear inclusions in fibroblasts **(E)**, sweat gland cells **(F)**, and adipocytes **(G)**. Scale bars, 10μm. **(H)** Electron microscopy imaging displaying intranuclear inclusions without membrane. Scale bars, 2μm. **(I-T)** All skin samples showed p62-positive intranuclear inclusions. Scale bars, 10μm.

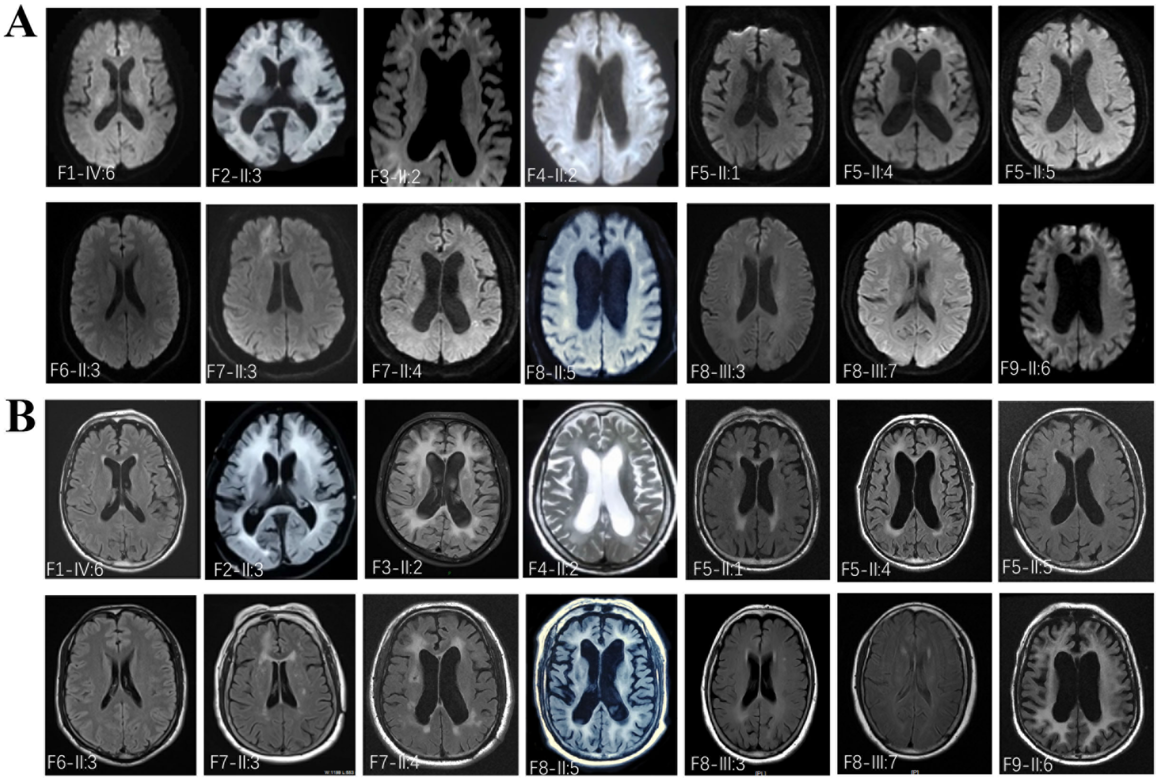

Figure S4. MRI Imaging of NIID. (A) DWI images of some NIID-affected case subjects revealed typical symmetrical high signal in corticomedullary junction. **(B)** Several NIID-affected case subjects showed severe white matter hyperintensity using FLAIR or T2 weighted image.

Table S1. Clinical Features of Muscle Weakness-dominant Type (families 1 and 2).

	Family1												Family2		
	III:5	III:8	III:11	IV:1	IV:4	IV:5	IV:6	IV:7	IV:10	IV:12	IV:15	V:1	II:1	II:2	II:3
Sex	M	M	M	M	M	M	M	M	M	M	F	M	F	F	M
Age at onset	30	/	30	54	31	40	33	32	30	47	34	31	38	33	35
Disease duration	49	/	38	6	18	8	14	9	13	3	14	3	20	20	18
Clinical manifestations															
Dementia	-	-	-	-	-	-	-	-	-	-	-	-	-	-	-
Abnormal behavior	-	-	+	-	-	-	-	-	-	-	-	-	-	++	-
Peripheral neuropathy															
Muscle weakness	-	-	++	+	++	++	++	++	++	+	++	+	+++	++	+++
Sensory disturbance		-	-	-	-	+	+	-	-	-	+	-	/	/	+
Autonomic dysfunction															
Bladder dysfunction	-	+	-	-	+	-	+	-	+++	-	-	-	-	-	++
Miosis	-	-	/	-	/	-	+	-	-	-	-	-	/	/	-
Parkinsonism															
Tremor	+	-	+	+	+	+	++	+	+	+	+	-	-	-	+
Rigidity	-	-	-	-	-	-	-	-	-	-	-	-	-	-	+
Bradykinesia	-	-	-	-	-	-	-	-	-	-	-	-	-	-	+
Ataxia	-	-	-	-	-	-	+	-	-	-	-	-	-	-	+
Neurological attack															
Disturbance of consciousness	+	-	-	-	-	-	-	-	-	-	-	+	-	-	+
Stroke-like episode	-	-	-	-	-	-	-	-	-	-	-	-	-	-	+
encephalitic episode	-	-	-	-	-	-	-	-	-	-	-	-	-	-	+
Brain MRI															
Severe leukoencephalopathy	/	-	/	/	-	-	-	-	/	/	-	/	/	/	+++
DWI U-fiber high signal	/	-	/	/	/	/	-	/	/	/	/	/	/	/	+
Cognitive function test															
MMSE (<education matched average)	/	/	/	/	28	28	29	28	28	/	29	/	/	/	28
MoCA (<education matched average)	/	/	/	/	26	18	21	26	25	/	23	/	/	/	26
Nerve conduction															
Motor															
MCV slowing	/	-	/	/	++	++	++	++	+	/	+	+	/	/	+++
CMAP reduction	/	-	/	/	+	+	+	+	+	/	+	-	/	/	++
Sensory															
SCV slowing	/	-	/	/	++	++	++	++	-	/	+	+	/	/	+++
SNAP reduction	/	-	/	/	+	+	+	+	-	/	+	-	/	/	+
Skin biopsy	/	+	/	/	/	/	+	/	/	/	/	/	/	/	+

Table S2. Clinical Features of Parkinsonism-dominant Type (families 5-7)

	Family 5			Family 6			Family 7		
	II:1	II:4	II:5	I:1	II:2	II:3	I:1	II:3	II:4
Sex	M	M	M	M	M	M	M	F	M
Age at onset	75	58	66	78	37	46	58	66	61
Disease duration	2	11	2	1	15	3	10	6	4
Clinical manifestations									
Dementia	-	-	-	-	-	-	-	-	-
Abnormal behavior	-	-	-	-	-	-	-	-	-
Peripheral neuropathy									
Muscle weakness	-	+	-	-	-	-	-	-	-
Sensory disturbance	+	-	-	+	-	-	-	+	+
Autonomic dysfunction									
Bladder dysfunction	-	++	-	-	-	+	+++	++	+++
Miosis	-	-	-	-	-	-	/	-	-
Parkinsonism									
Tremor	+	++	-	-	+	-	-	+	+
Rigidity	+	++	+	+	+	+	+	++	+
Bradykinesia	+	++	+	+	+	++	+	++	+
Ataxia	-	+	-	-	-	-	++	+	++
Neurological attack							-		
Disturbance of consciousness	-	+	-	-	-	-	-	-	-
Stroke-like episode	-	+	+	-	-	-	-	-	-
encephalitic episode	-	-	-	-	-	-	-	-	-
Brain MRI									
Severe leukoencephalopathy	-	+	-	/	/	-	/	-	+
DWI U-fiber high signal	-	+	-	/	/	-	/	-	-
Cognitive function test									
MMSE (<education matched average)	26	27	26	/	29	30	/	22	25
MoCA (<education matched average)	19	15	19	/	/	25	/	/	23
Nerve conduction									
Motor									
MCV slowing	+	+	+	/	/	+	/	++	+
CMAP reduction	+	-	-	/	/	-	/	-	+
Sensory									
SCV slowing	+	-	-	/	/	-	/	++	+
SNAP reduction	-	-	-	/	/	-	/	+	+
Skin biopsy	+	+	+	/	/	+	/	+	/

Table S3. Clinical Features of Dementia-dominant Type (families 3, 4, 8, 9)

	Family 3			Family 4		Family 8							Family 9			
	I:2	II:2	II:3	II:2	II:5	I:1	II:3	II:5	II:8	III:3	III:5	III:7	I:2	II:2	II:3	II:6
Sex	F	M	F	F	F	M	F	M	M	F	F	F	F	M	F	M
Age at onset	71	42	63	49	31	65	60	60	60	60	58	59	60	60	60	71
Disease duration	20	19	2	18	30	5	8	19	16	7	5	3	15	10	12	10
Clinical manifestations																
Dementia	++	-	+	+	++	+++	+++	+++	+++	++	+	-	++	++	++	+++
Abnormal behavior	++	++	+	++	+++	++	++	++	++	-	-	-	++	++	++	+
Peripheral neuropathy																
Muscle weakness	-	-	+	-	-	/	-	+	-	-	-	+	-	-	-	+
Sensory disturbance	+	-	+	-	-	/	-	+	-	-	+	+	-	-	-	-
Autonomic dysfunction																
Bladder dysfunction	+	-	++	++	-	+	+++	+++	+++	-	++	+++	/	+++	+++	+++
Miosis	-	-	-	+	+	/	/	+	/	-	-	-	/	/	/	+
Parkinsonism																
Tremor	-	-	-	+	-	-	-	+	-	-	-	-	-	-	-	+
Rigidity	-	-	-	++	-	-	-	-	-	-	-	-	-	-	-	+
Bradykinesia	-	-	-	+	-	-	-	-	-	-	-	-	-	-	-	+
Ataxia	+	-	-	-	-	-	-	-	-	-	-	-	-	-	-	-
Neurological attack																
Disturbance of consciousness	-	-	-	+	-	-	-	-	-	-	+	-	-	-	-	+
Stroke-like episode	-	-	-	+	-	-	-	-	-	-	-	-	-	-	-	-
encephalitic episode	-	-	-	-	-	-	-	-	-	-	-	-	-	-	-	+
Brain MRI																
Severe leukoencephalopathy	/	/	+	+	/	/	/	+	/	-	-	-	/	/	/	+
DWI U-fiber high signal	/	/	+	+	/	/	/	+	/	-	-	-	/	/	/	+
Cognitive function test							/									
MMSE (<education matched average)	14	29	/	15	12	/	/	/	/	10	26	29	/	/	/	/
MoCA (<education matched average)	/	28	/	/	/	/	/	/	/	3	20	26	/	/	/	/
Nerve conduction																
Motor																
MCV slowing	/	+	/	-	/	/	/	+	/	-	+	+	/	/	/	++
CMAP reduction	/	+	/	-	/	/	/	+	/	-	+	+	/	/		++
Sensory																
SCV slowing	/	+	/	-	/	/	/	+	/	-	+	+	/	/	/	+
SNAP reduction	/	+	/	-	/	/	/	+	/	-	+	+	/	/	/	+
Skin biopsy	/	+	/	+	/	/	/	/	/	+	+	+	/	/	/	+

Table S4. Clinical Features of Sporadic NIID-affected Case Subjects

	Sporadic Case Subjects				
	Dementia		Paroxysmal disease		
	SD1	SD2	SP1	SP2	SP3
Sex	F	F	M	M	F
Age at onset	65	69	68	51	57
Disease duration	14	2	1	5	6
Clinical manifestations					
Dementia	++	++	-	-	-
Abnormal behavior	++	+	-	-	-
Peripheral neuropathy					
Muscle weakness	-	-	-	-	-
Sensory disturbance	-	-	-	-	-
Autonomic dysfunction					
Bladder dysfunction	+++	+++	-	++	-
Miosis	+	+	-	-	-
Parkinsonism					
Tremor	+	-	-	-	-
Rigidity	+	-	-	-	-
Bradykinesia	+	-	-	-	-
Ataxia	-	-	-	-	-
Neurological attack					
Disturbance of consciousness	+	+	+	+	-
Stroke-like episode	+	+	+	-	+
encephalitic episode	+	+	-	-	+
Brain MRI					
Severe leukoencephalopathy	+	+	+	+	+
DWI U-fiber high signal	+	+	+	+	+
Cognitive function test					
MMSE (<education matched average)	/	/	27	30	/
MoCA (<education matched average)	/	/	16	29	/
Nerve conduction					
Motor					
MCV slowing	+	/	+	+	/
CMAP reduction	+	/	-	-	/
Sensory					
SCV slowing	+	/	+	+	/
SNAP reduction	+	/	-	-	/
Skin biopsy	+	+	+	+	+
